# Severe attenuation of circadian clock output in the heart following sustained augmentation of cardiomyocyte protein O-GlcNAcylation

**DOI:** 10.3389/fcvm.2025.1601407

**Published:** 2025-07-17

**Authors:** Gobinath Shanmugam, Samuel F. Chang, Helen E. Collins, Chae-Myeong Ha, Mariame S. Kane, Luke Potter, Lily Xie, Luyun Zou, Jianhua Zhang, John C. Chatham, Adam R. Wende, Martin E. Young

**Affiliations:** ^1^ Division of Cardiovascular Disease, Department of Medicine, University of Alabama at Birmingham, Birmingham, AL, United States; ^2^ Division of Molecular Cellular Pathology, Department of Pathology, University of Alabama at Birmingham, Birmingham, AL, United States; ^3^ Division of Environmental Medicine, Department of Medicine, Center for Cardiometabolic Science, University of Louisville, Louisville, KY, United States

**Keywords:** chronobiology, fibrosis, gene expression, post-translational modification, glycobiology

## Abstract

**Background:**

Changes in circadian-related behaviors (e.g., the timing of food intake, sleep cycles) and the environment (e.g., light-dark cycles) increase the risk of numerous cardiometabolic diseases, including diabetes mellitus and cardiac disease. Recent studies indicate a close interrelationship between circadian clocks and the posttranslational modification, protein O-GlcNAcylation. The current study was designed to investigate whether a modest elevation of protein O-GlcNAcylation in the adult mouse heart, similar to levels observed during pathologic states, influenced circadian governance of the heart.

**Methods and results:**

Cardiomyocyte-specific expression of a dominant negative O-GlcNAcase (dnOGAh) for a 2-week period resulted in an approximate 1.5-fold increase in cardiac protein O-GlcNAcylation, impacting 70% of core circadian clock components in the heart at the mRNA level. Further interrogation of cardiac mRNA species in dnOGAh hearts at candidate (RT-PCR) and unbiased (RNAseq) levels revealed a 95% loss of circadian governance of the cardiac transcriptome. This was despite persistent/augmented 24 h oscillations of the core circadian clock proteins BMAL1, REVERB*α*, and PER2 in dnOGAh hearts. Direct comparison of dnOGAh hearts with cardiomyocyte-specific BMAL1 knockout (CBK) hearts underscored an apparent uncoupling of the core clock mechanism from clock control of downstream target genes in dnOGAh hearts, and highlighted that loss of circadian governance results in interstitial fibrosis.

**Conclusions:**

Sustained protein O-GlcNAcylation in the heart causes loss of circadian governance, likely downstream of the core circadian clock mechanism. Moreover, interstitial fibrosis appears to be a universal adverse outcome following impaired circadian governance.

## Introduction

Reversible posttranslational modification (PTM) of cellular proteins is an established means by which biological processes are modulated in both acute and chronic manners. Several hundred different PTMs have been identified, although the function of many of these modifications are not known. Most extensively studied PTMs include phosphorylation, acetylation, palmitoylation, SUMOylation, and O-GlcNAcylation (1). The latter involves the reversible, catalytic O-linked addition of β-N-acetyl-glucosamine to serine and threonine residues of target proteins by the enzyme O-GlcNAc transferase (OGT) (2). This PTM affects protein stability, cellular localization, and functions such as protein-protein interactions and intrinsic activity (2). It has been estimated that >5,000 proteins can be O-GlcNAc modified; O-GlcNAcylated proteins mediate/regulate diverse biological processes, ranging from transcription, translation, and protein quality control, to signaling, electrophysiology, and metabolism (3). Given that the carbon required for this PTM is derived from glucose, fatty acids, and amino acids (via the hexosamine biosynthetic pathway), protein O-GlcNAcylation has classically been considered a nutrient sensing pathway (3). However, it has become increasingly clear that alterations in protein O-GlcNAcylation occur in response to a variety of nutrient-independent stimuli/stresses, in both acute and chronic settings (2, 4). This can be exemplified by studies in the heart, for which perturbations in protein O-GlcNAcylation have been causally linked to acute adaptations to physiologic stimuli (e.g., exercise) and chronic pathologic conditions (e.g., diabetes mellitus and cardiac hypertrophy) (2, 4–6). In the latter case, recently published studies indicate that selective augmentation of protein O-GlcNAcylation in the adult murine heart results in interstitial fibrosis and diastolic dysfunction, which is comparable to the pathologic cardiac remodeling observed during diabetes (7).

Consistent with the dynamic and reversible nature of protein O-GlcNAcylation, levels of this PTM fluctuate over the course of the day in multiple organs, including the heart, peaking during the awake period (8, 9). For the heart, these oscillations appear to be driven in large part by the cardiomyocyte circadian clock (8). This cell autonomous timekeeping molecular mechanism is comprised of a series of interconnected transcription-translation feedback loops, which have a free running periodicity of approximately 24 h (10). Numerous core circadian clock components are regulated by PTMs, which are critical for the temporal nature of this timekeeping mechanism (11). One such PTM is protein O-GlcNAcylation. For example, BMAL1 (brain and muscle ARNT-like 1), CLOCK (circadian locomotor output cycles kaput), and PER (period) proteins can be O-GlcNAc modified, which affects the nuclear-cytosolic localization, DNA binding, and/or stability of these core circadian clock components (8, 9, 12, 13). Consistent with these reports, acute (i.e., <24 h) pharmacologic augmentation of protein O-GlcNAcylation cause a rapid phase shift in the heart circadian clock (8).

Considerable overlap exists between the cellular processes regulated by circadian clocks and protein O-GlcNAcylation. Moreover, cardiomyocyte circadian clock disruption and chronic elevation of cardiomyocyte protein O-GlcNAcylation both result in cardiac fibrosis and contractile dysfunction (7, 14–16). Despite these observations, and the aforementioned interrelationship between the circadian clock and protein O-GlcNAcylation, it is unclear whether sustained elevation of O-GlcNAcylation (as might be observed during a disease state) contributes towards adverse cardiac remodeling through impairment of circadian governance (i.e., the ability of the circadian clock to temporally regulate biologic processes). To gain insight into this possibility, we compared the impact of sustained elevation of cardiomyocyte protein O-GlcNAcylation and cardiomyocyte BMAL1 deletion on circadian governance. These studies revealed that a modest (∼1.5-fold) increase in cardiac protein O-GlcNAcylation for 2 weeks is sufficient to severely attenuate the cardiac diurnal transcriptome, including circadian governance of genes involved in the extracellular matrix and collagen deposition.

## Methods

### Animals

The present study utilized inducible, cardiomyocyte-specific dominant negative O-GlcNAcase (dnOGAh) transgenic mice, as described previously (7). Briefly, to generate dnOGAh mice, TRE-EGFP-dnOGA (tetracycline-responsive element, enhanced green fluorescent protein, rat OGA splice variant) mice were bred with αMHC-rtTA (α-myosin heavy chain promoter driven codon optimized reverse tetracycline transactivator) mice. To induce the transgene, 10 week old male dnOGAh mice were injected with 100 μg doxycycline (DOX) (MilliporeSigma, Burlington, MA, USA) in 0.9% NaCl solution and immediately switched to 1 g/kg DOX-supplemented NIH31 diet (Envigo, Madison, WI, USA). αMHC-rtTA mice treated with DOX for the same duration served as controls (CON). This study also utilized male cardiomyocyte-specific BMAL1 knockout (CBK) mice; littermate floxed mice served as controls (CON), as described previously (14). All mice were housed at the Center for Comparative Medicine at the University of Alabama at Birmingham (UAB), under temperature-, humidity-, and light- controlled conditions. A strict 12 h light/12 h dark cycle regime was enforced [lights on at 6 a.m.; zeitgeber time (ZT) 0]; the light/dark cycle was maintained throughout these studies, facilitating investigation of diurnal variations (as opposed to circadian rhythms). Mice were housed either in standard micro-isolator cages or CLAMS (Comprehensive Laboratory Animal Monitoring System) cages, and received food and water in an *ad libitum* fashion. CLAMS cages allowed the non-invasive assessment of energy expenditure, food intake and physical activity in a continuous manner, as described previously (17). All mice were on the C57BL/6 background strain, were male, and were 12 weeks old at the time of assessments and/or tissue collection. All animal experiments were approved by the Institutional Animal Care and Use Committee of the University of Alabama at Birmingham.

### Quantitative RT-PCR

RNA was extracted from biventricular samples using standard procedures. Candidate gene expression analysis was performed by quantitative RT-PCR, using previously described methods (18, 19). For quantitative RT-PCR, custom-designed Taqman assay were utilized; sequences for custom-designed assays have been reported previously (14, 20). All quantitative RT-PCR data are presented as fold change from an indicated control group.

### RNA sequencing

Transcriptomic analysis was performed using RNA sequencing (RNA-seq) in the UAB Genomics Core facility. Following initial testing of RNA samples using an Agilent BioAnalyzer, RNA with RIN values greater than 7.0 were subsequently utilized for library preparation (after DNAse treatment). RNA-Sequencing libraries were next generated using the NEBNext Ultra II RNA kit (NEB, Ipswich MA); resulting libraries were sequenced on the Illumina NextSeq 500 (Illumina, Inc. San Diego CA) using paired end 75 bp sequencing reads, per standard methods. Sequencing data can be accessed in GEO.

### Western blotting

Qualitative analysis of protein expression was performed via standard western blotting procedures, as described previously (8). Briefly, 15–30 μg protein lysate was separated on polyacrylamide gels and transferred to PVDF membranes. Membranes were probed for with anti- BMAL1 (Cell Signaling Tech #14020; 1:2000), REVERBα (Cell Signaling Tech #13418; 1:2000), PER2 (Alpha Diagnostics Inc #PER21-A; 1:2500), and E4BP4 (Cell Signaling Tech #14312; 1:2000) antibodies, or with the CTD110.6 antibody (UAB Epitope Recognition Core) for detection of O-GlcNAcylation. Rabbit and mouse HRP-conjugated secondary antibodies (Cell Signaling 7074 and 7076, respectively; 1:2000–5000) were used for chemiluminescent detection with Luminata Forte Western Blotting substrate (Millipore, WBLUF0100). All densitometry data were normalized to amido black staining. Importantly, to minimize the contribution that position on the gel might have on outcomes, samples were randomized on gels; all original gels are included in Supplemental files.

### Histologic assessment

Cross sections from the middle region of the left ventricle were taken immediately upon removal of the heart, and were fixed in formalin for 24 h (followed by storage in 70% ethanol at 4°C prior to paraffin embedding and sectioning). Assessment of left ventricular interstitial fibrosis employed either Picrosirius Red staining (CBK hearts) or Masson Trichrome staining (dnOGAh hearts), followed by semi-quantitative analysis using Image-J software (NIH), as described previously (21). All original images are included in Supplemental files.

### Statistical analysis

All data are presented as mean ± standard error of the mean (SEM). For non-omics data (i.e*.*, not RNA-seq data), comparisons among groups with two variables were analyzed by two-way analysis of variance (ANOVA) through use of Prism statistical software to investigate main effects of genotype and time. Normality of data was assessed through use of the Shapiro–Wilks test, followed by either parametric (*t*-tests for only two experimental groups or Sidak's *post-hoc* test for multiple pairwise comparisons) or non-parametric (Mann–Whitney for only two experimental groups or Kruskal–Wallis with Dunn's correction for multiple pairwise comparisons). Cosinor analyses were performed to determine whether 24 h time series data significantly fit a cosine curve; if they did, then mesor (daily average value), amplitude (peak-to-mesor difference), and acrophase (timing of the peak) were calculated and compared between experimental groups, as described previously (22). For RNA-seq data, JTK cycle was performed using the online tool NiteCap. For the JTK cycle analysis, transcripts were considered to have a 24 h oscillation if they significantly fit a cosine curve (*q* < 0.05); amplitude and acrophase were calculated within NiteCap, which also determined significant differences between genotypes for these cosine parameters. NiteCap was further utilized for heat map generation and principal component analysis (PCA). In addition, the online tool PANTHER was utilized for pathway enrichment analysis. In all analyses, the null hypothesis of no model effects was rejected at *p* < 0.05.

### Biological variables

The primary outcomes of this study included gene expression, protein expression, and fibrosis; one or more of these outcomes are influenced by time-of-day, sex, age, diet, and genetic background. Consistent with the stated purpose of the study, two main variables were investigated: time-of-day and genotype (i.e., CBK, dnOGAh, and littermate controls). This resulted in a total of 28 experimental groups: 16 experimental groups for the CBK model and 12 experimental groups for the dnOGAh model. To the extent that was possible based on the study design, other biological variables remained constant. Male 12 week old mice on the C57BL/6 background were chosen, allowing direct comparison of RNA-seq data with previously published transcriptomic data obtained from CBK and littermate control hearts (14). Our recent studies investigating male and female dnOGAh mice at this age revealed comparable phenotypes (7). One variable that was not identical between CBK and dnOGAh models was diet; dnOGAh mice were fed a doxycycline containing diet (to induce the transgene), whereas CBK mice were fed a normal rodent chow.

## Results

### Cardiomyocyte-specific BMAL1 deletion impacts both circadian clock gene expression and protein O-GlcNAcylation in the heart

**Figure 1:**
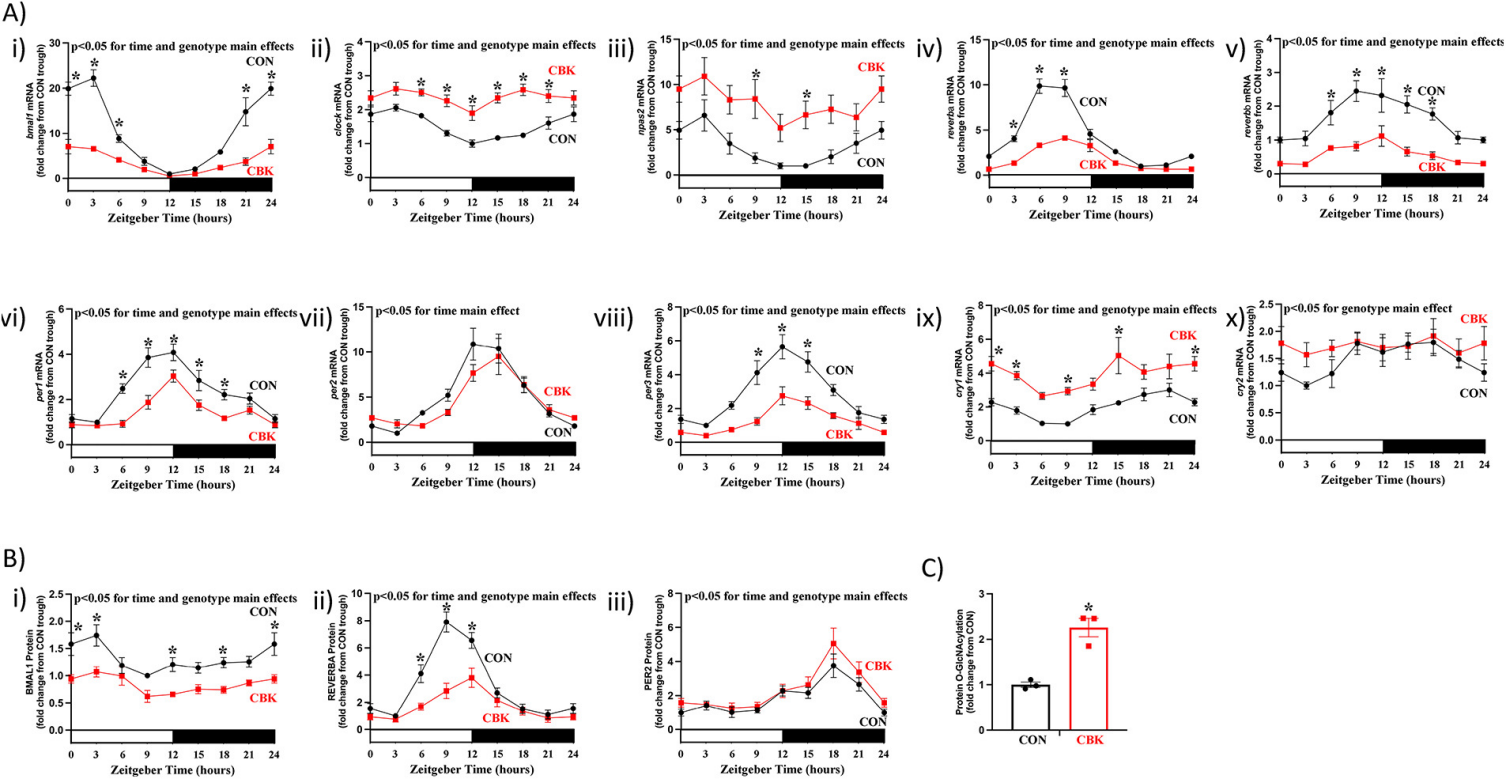
Impact of cardiomyocyte-specific BMAL1 deletion on circadian clock components and protein O-GlcNAcylation in the heart. Hearts were collected from CBK and littermate CON mice at 3 h intervals over a 24 h period, followed by RT-PCR and Western Blot analyses. **(A)** mRNA levels of bmal1 (i), clock (ii), npas2 (iii), reverbα (iv), reverbβ (v), per1 (vi), per2 (vii), per3 (viii), cry1 (ix), and cry2 (x) in CON and CBK hearts (*n* = 5−6). **(B)** Protein levels of BMAL1 (i), REVERB*α* (ii), and PER2 (iii) in CON and CBK hearts (*n* = 5−8). **(C)** Protein O-GlcNAcylation levels in CON and CBK hearts at ZT9 (*n* = 3). In panels A and B, ZT0 and ZT24 are identical (the data are double plotted purely for the sake of presentation). Please see Supplemental files for all original images. Data are presented as mean ± SEM, and have been normalized to the lowest (trough) value in CON hearts. Main effects of time and genotype are reported at the top of the figure panels. *, *p* < 0.05 for CON vs. CBK hearts (at the same time-of-day).

To identify roles of the cardiomyocyte circadian clock, we previously generated a cardiomyocyte-specific BMAL1 knockout (CBK) mouse model (14). To confirm that the cardiac clock is disrupted in CBK mice, hearts were collected from CBK and littermate control mice at 3 h intervals over a 24 h period, followed by assessment of circadian clock components at mRNA and protein levels. At the transcript level, 10 core circadian clock components were investigated; *bmal1*, *clock*, *npas2*, *rever**bα*, *rever**bβ*, *per1*, *per2*, *per3*, *cry1*, and *cry2* mRNA. A 2-way ANOVA revealed significant time main effects for 9 out of 10 core clock component mRNAs (Figure 1A; Supplementary Table 1); only *cry2* mRNA did not exhibit a significant time main effect (*p* = 0.187). Significant genotype main effects were also observed for 9 out of 10 core clock component mRNAs (Figure 1A; Supplementary Table 1); *per2* mRNA did not exhibit a significant genotype main effect (*p* = 0.183). Of those transcripts that exhibited significant genotype main effects, *bmal1*, *rever**bα*, *rever**bβ*, *per1*, and *per3* were repressed, while *clock*, *npas2*, *cry1*, and *cry2* were induced (Figure 1A; Supplementary Table 1). Cosinor analysis indicated that all 10 core clock component mRNAs exhibit 24hr oscillations in CON hearts; these oscillations were either abolished or significantly attenuated in CBK hearts for 8 out of 10 core clock component mRNAs (*cry2* and *per2* mRNA 24 h oscillations persisted in CBK hearts; Supplementary Table 2). At the protein level, 3 core circadian clock components were investigated; BMAL1, REVERBα, and PER2. A 2-way ANOVA revealed significant time and genotype main effects for all 3 core clock proteins; both BMAL1 and REVERBα protein levels were decreased in CBK hearts, while PER2 levels were increased (Figure 1B; Supplementary Table 1). Cosinor analysis indicated that all 3 core clock component proteins exhibit 24 h oscillations in CON hearts; REVERBα oscillations were significantly attenuated in CBK hearts (Supplementary Table 2). Consistent with the cardiomyocyte-specific nature of the murine model, CBK mice exhibited 24 h oscillations in energy expenditure, food intake, and physical activity that were comparable to littermate control mice (Supplementary Figure 1A, as well as Supplementary Tables 1, 2). Similarly, we have previously reported that distinct circadian clock components are not altered in livers of CBK mice (14). Next, we investigated protein O-GlcNAcylation levels in CON and CBK hearts isolated during the light phase (ZT9); protein O-GlcNAcylation levels were significantly increased in CBK hearts (2.26-fold; Figure 1C). Collectively, these data are consistent with the concept that cardiomyocyte BMAL1 deletion disrupts the clock in the heart, and this is associated with augmentation of protein O-GlcNAcylation.

### Impact of inducible dnOGA overexpression on core circadian clock components in the heart

**Figure 2:**
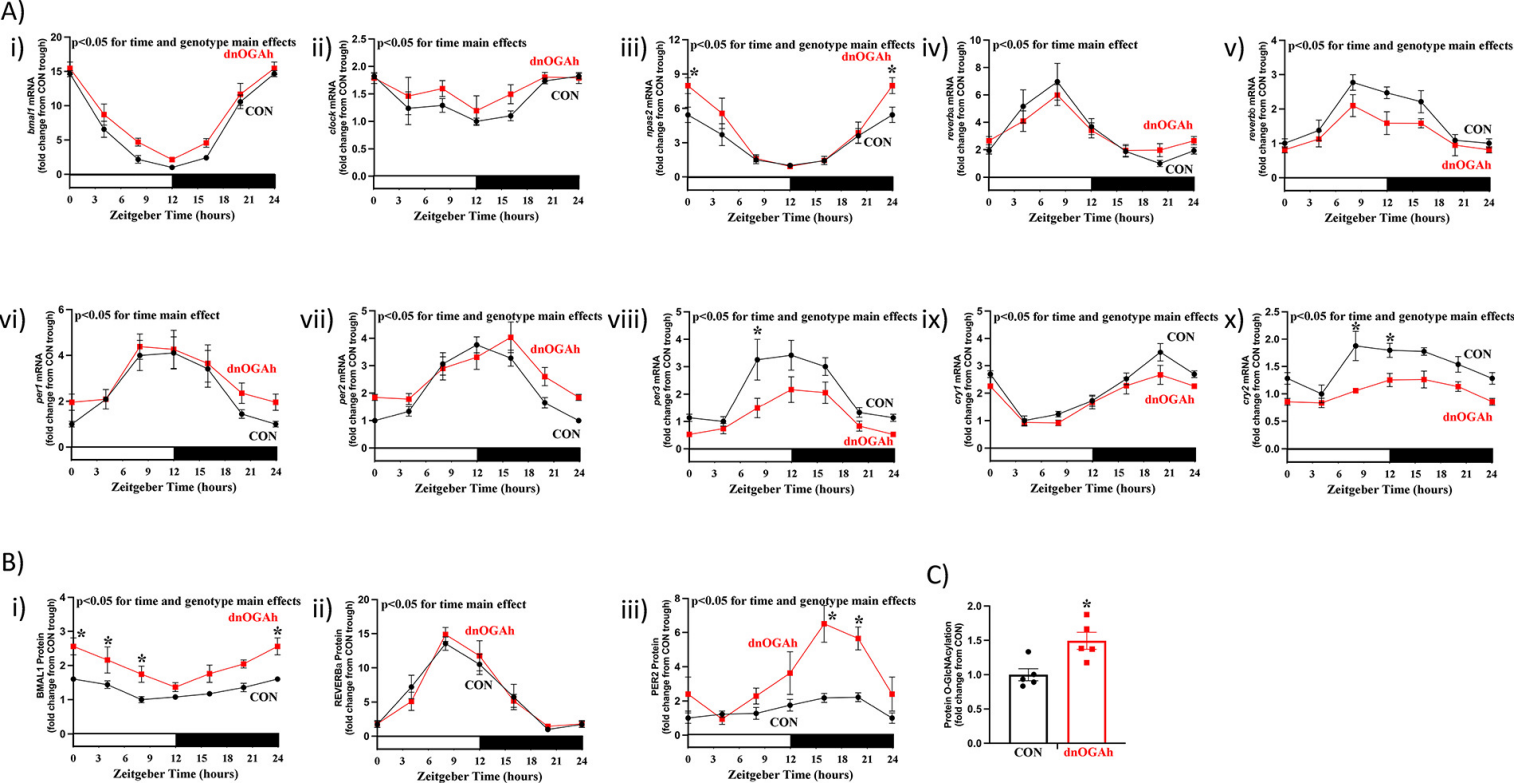
Impact of augmented protein O-GlcNAcylation on circadian clock components in the heart. Hearts were collected from dnOGAh and littermate CON mice at 4 h intervals over a 24 h period, 2 weeks after doxycycline treatment initiation, followed by RT-PCR and Western Blot analyses. **(A)** mRNA levels of *bmal1* (i), *clock* (ii), *npas2* (iii), *reverbα* (iv), r*everbβ* (v), *per1* (vi), *per2* (vii), *per3* (viii), *cry1* (ix), and *cry2* (x) in CON and dnOGAh hearts (*n* = 5−9). **(B)** Protein levels of BMAL1 (i), REVERB*α* (ii), and PER2 (iii) in CON and dnOGAh hearts (*n* = 5). **(C)** Protein O-GlcNAcylation levels in CON and dnOGAh hearts at ZT8 (*n* = 5). In panels A and B, ZT0 and ZT24 are identical (the data are double plotted purely for the sake of presentation). Please see Supplemental files for all original images. Data are presented as mean ± SEM, and have been normalized to the lowest (trough) value in CON hearts. Main effects of time and genotype are reported at the top of the figure panels. *, *p* < 0.05 for CON vs. dnOGAh hearts (at the same time-of-day).

We, and others, have reported that several circadian clock components are O-GlcNAc modified, impacting their cellular localization, DNA binding, and/or protein stability (8, 9, 12, 13). Moreover, disruption of the cardiomyocyte circadian clock impacts protein O-GlcNAcylation levels (Figure 1C), consistent with an interrelationship between the clock and O-GlcNAcylation (13). We therefore hypothesized that cardiomyocyte dnOGA induction would impact the cardiac circadian clock and protein O-GlcNAcylation in a manner similar to that observed in CBK hearts. To test this hypothesis, hearts were isolated from dnOGAh and littermate CON mice at 4 h intervals over the 24 h day, 2 weeks after doxycycline treatment initiation; this duration of transgene induction is not associated with cardiac dysfunction (7). Following RNA isolation, RT-PCR was performed for the same 10 core circadian clock components investigated in CBK hearts. A 2-way ANOVA revealed significant time main effects for all 10 core clock component mRNAs (Figure 2A; Supplementary Table 3). Significant genotype main effects were observed for 7 out of 10 core clock component mRNAs (Figure 2A; Supplementary Table 3); *clock*, *rever**bα*, and *per1* mRNA did not exhibit significant genotype main effects (*p*-values of 0.054, 0.821, and 0.217, respectively). Of those transcripts that exhibited significant genotype main effects, *rever**bβ*, *per3*, *cry1*, and *cry2* were repressed, while *bmal1*, *npas2*, and *per2* were induced (Figure 2A; Supplementary Table 3). Cosinor analysis indicated that all 10 core clock component mRNAs exhibit 24 h oscillations in CON hearts; these oscillations were significantly altered in dnOGAh hearts for only *npas2* mRNA (Supplementary Table 4). At the protein level, 3 core circadian clock components were investigated; BMAL1, REVERBα, and PER2. A 2-way ANOVA revealed significant time main effects for all 3 core clock proteins, and significant genotype main effects for BMAL1 and PER2 (Figure 2B; Supplementary Table 3); REVERBα did not exhibit a significant genotype main effect (*p* = 0.916). Both BMAL1 and PER2 protein levels were increased in dnOGAh hearts (Figure 2B; Supplementary Table 3). Cosinor analysis indicated that all 3 core clock component proteins exhibit 24 h oscillations in CON hearts; PER2 oscillations were significantly augmented in dnOGAh hearts (Supplementary Table 4). Consistent with the cardiomyocyte-specific nature of the murine model, dnOGA mice exhibited 24 h oscillations in energy expenditure, food intake, and physical activity that were comparable to littermate control mice (Supplementary Figure 1B, as well as Supplementary Tables 3, 4). Similarly, 24 h oscillations in *bmal1*, *rever**bα* and *dbp* mRNA levels were not different between CON and dnOGAh livers (Supplementary Figure 2). Next, we investigated protein O-GlcNAcylation levels in CON and dnOGA hearts isolated during the light phase (ZT8); protein O-GlcNAcylation levels were significantly increased in dnOGAh hearts (Figure 2C). Collectively, these data are consistent with the concept that increasing protein O-GlcNAcylation in the heart impacts the circadian clock, in a manner that is distinct from CBK mice.

### Attenuation of circadian clock output following dnOGA induction

**Figure 3:**
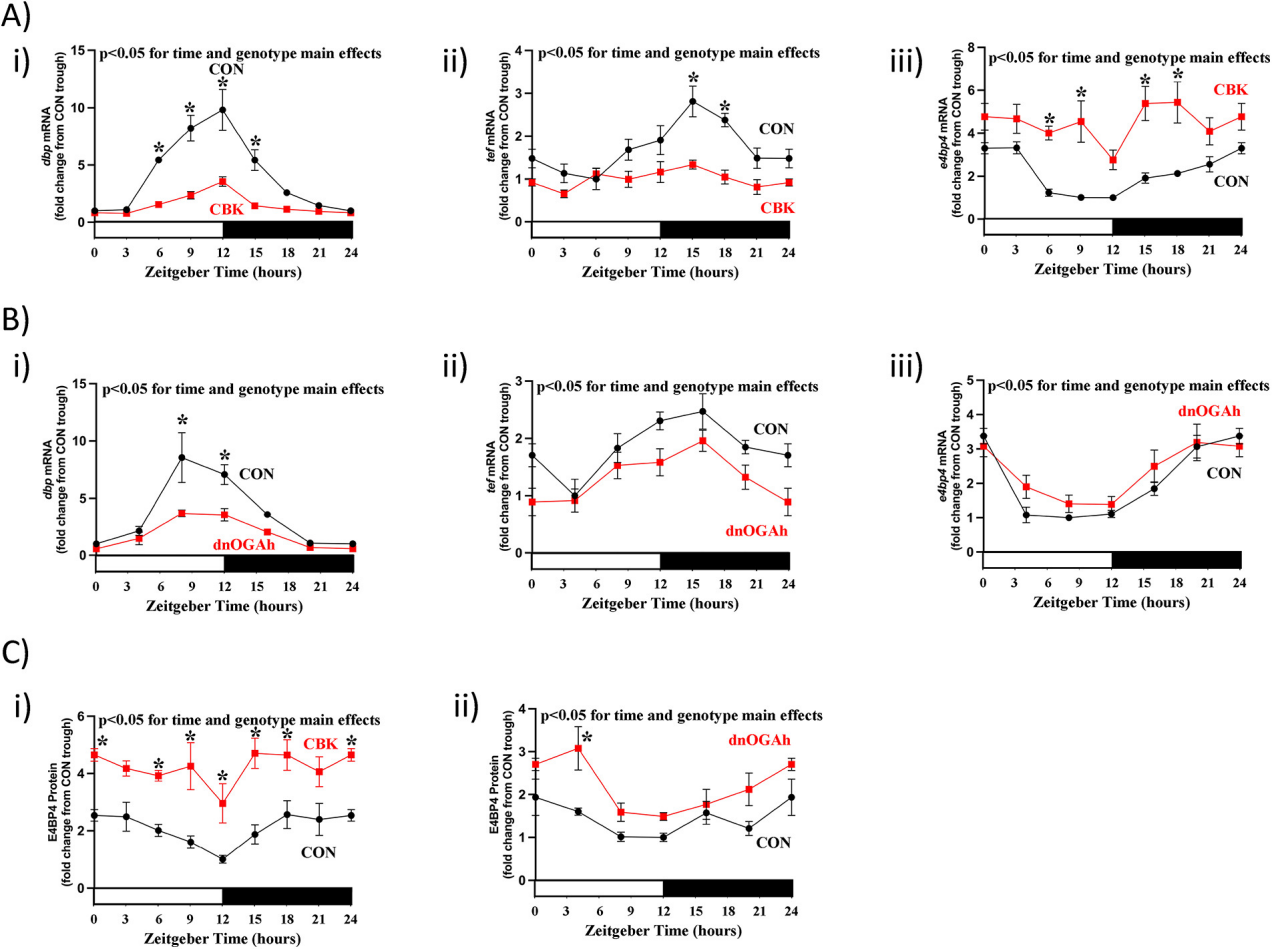
Diurnal variations in direct circadian clock-controlled genes/proteins in CBK and dnOGA hearts. Hearts were collected either from CBK and littermate CON mice at 3 h intervals over a 24 h period, or from dnOGAh and littermate CON mice at 4 h intervals over a 24 h period (2 weeks after doxycycline treatment initiation), followed by RT-PCR and Western Blot analyses. **(A)** mRNA levels of direct circadian clock-controlled transcription factors *dbp* (i), *tef* (ii), and *e4bp4* (iii) in CON and CBK hearts (*n* = 5−6). **(B)** mRNA levels of direct circadian clock-controlled transcription factors *dbp* (i), *tef* (ii), and *e4bp4* (iii) in CON and dnOGA hearts (*n* = 5−9). **(C)** E4BP4 protein levels in CBK (i) and dnOGAh (ii) hearts compared to littermate CON hearts (*n* = 5−6). In all panels, ZT0 and ZT24 are identical (the data are double plotted purely for the sake of presentation). Please see Supplemental files for all original images. Data are presented as mean ± SEM, and have been normalized to the lowest (trough) value in CON hearts. Main effects of time and genotype are reported at the top of the figure panels. *, *p* < 0.05 for CON vs. CBK/dnOGAh hearts (at the same time-of-day).

Core circadian clock components directly regulate the expression of numerous genes that do not feedback on the clock mechanism, but instead impact cellular functions (10). These clock output genes include the PAS transcription factors, DBP and TEF, which are direct BMAL1/CLOCK targets; DNA binding activity of DBP and TEF is directly antagonized by the REV-ERBα/β target E4BP4 (23, 24). As such, *dbp*, *tef*, and *e4bp4* mRNA levels were next assessed in both CBK and dnOGAh mouse models. A 2-way ANOVA revealed significant time and genotype main effects for all 3 clock output mRNAs in both CBK and dnOGAh hearts (Figures 3A,B, as well as Supplementary Tables 1, 3). Transcripts for *dbp* and *tef* were significantly decreased in CBK and dnOGAh hearts, while *e4bp4* mRNA was significantly increased in hearts of both mouse models (Figures 3A,B, as well as Supplementary Tables 1, 3). Cosine analysis revealed that 24 h oscillations of these 3 output mRNAs were either abolished or significantly attenuated in CBK hearts (Supplementary Table 2). Similarly, 24 h oscillations were significantly attenuated for *dbp* mRNA in dnOGAh hearts (Supplementary Table 4). E4BP4 protein levels were next investigated in both mouse models. A 2-way ANOVA revealed significant time and genotype main effects for E4BP4 protein in both CBK and dnOGAh; E4BP4 protein was increased in both models (Figure 3C, as well as Supplementary Tables 1, 3). Although 24 h oscillations in E4BP4 protein were abolished in CBK hearts, these oscillations were not significantly altered in dnOGAh hearts (Supplementary Tables 2–4).

**Figure 4:**
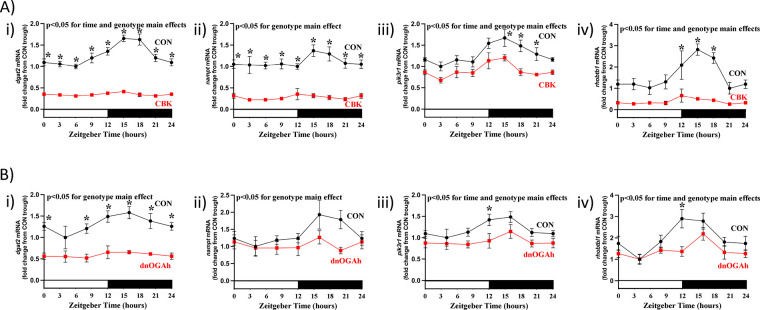
Diurnal variations in candidate clock-controlled genes in CBK and dnOGA hearts. Hearts were collected either from CBK and littermate CON mice at 3 h intervals over a 24 h period, or from dnOGAh and littermate CON mice at 4 h intervals over a 24 h period (2 weeks after doxycycline treatment initiation), followed by RT-PCR and Western Blot analyses. **(A)** mRNA levels of the clock-controlled genes *dgat2* (i), *nampt* (ii), *pik3r1* (iii), and *rhobtb1* (iv) in CON and CBK hearts (*n* = 5−6). **(B)** mRNA levels of the clock-controlled genes *dgat2* (i), *nampt* (ii), *pik3r1* (iii), and *rhobtb1* (iv) in CON and dnOGA hearts (*n* = 5−9). In all panels, ZT0 and ZT24 are identical (the data are double plotted purely for the sake of presentation). Data are presented as mean ± SEM, and have been normalized to the lowest (trough) value in CON hearts. Main effects of time and genotype are reported at the top of the figure panels. *, *p* < 0.05 for CON vs. CBK/dnOGAh hearts (at the same time-of-day).

Given similarities between CBK and dnOGAh hearts, in terms of impact on established clock output genes (i.e., *dbp*, *tef*, *e4bp4*), we next investigated transcripts involved in metabolism (*dgat2*, *nampt*) and signaling (*pik3r1*, *rhobtb1*) that we have previously reported to be regulated by the cardiomyocyte circadian clock (14, 25). A 2-way ANOVA revealed significant time main effects for *dgat2*, *pik3r1*, and *rhobtb1* (Figures 4A,B, as well as Supplementary Tables 1, 3). In addition, genotype main effects were found for all 4 clock-controlled mRNAs, in both CBK and dnOGAh hearts; these 4 transcripts were decreased in both models (Figures 4A,B, as well as Supplementary Tables 1, 3. Cosine analysis revealed significant 24 h oscillations of these 4 transcripts in CON hearts (except for CBK littermate CON hearts, for which 24 h oscillations did not achieve statistical significance; Supplementary Tables 2–4). 24 h oscillations in these transcripts were abolished in CBK and dnOGA hearts (except for *pik3r1* in CBK hearts and *rhobtb1* in dnOGAh hearts; Supplementary Table 2–4). Collectively, these data are consistent with attenuated circadian clock output in the heart following increased protein O-GlcNAcylation.

### The diurnal transcriptome of dnOGAh hearts is severely attenuated

**Figure 5:**
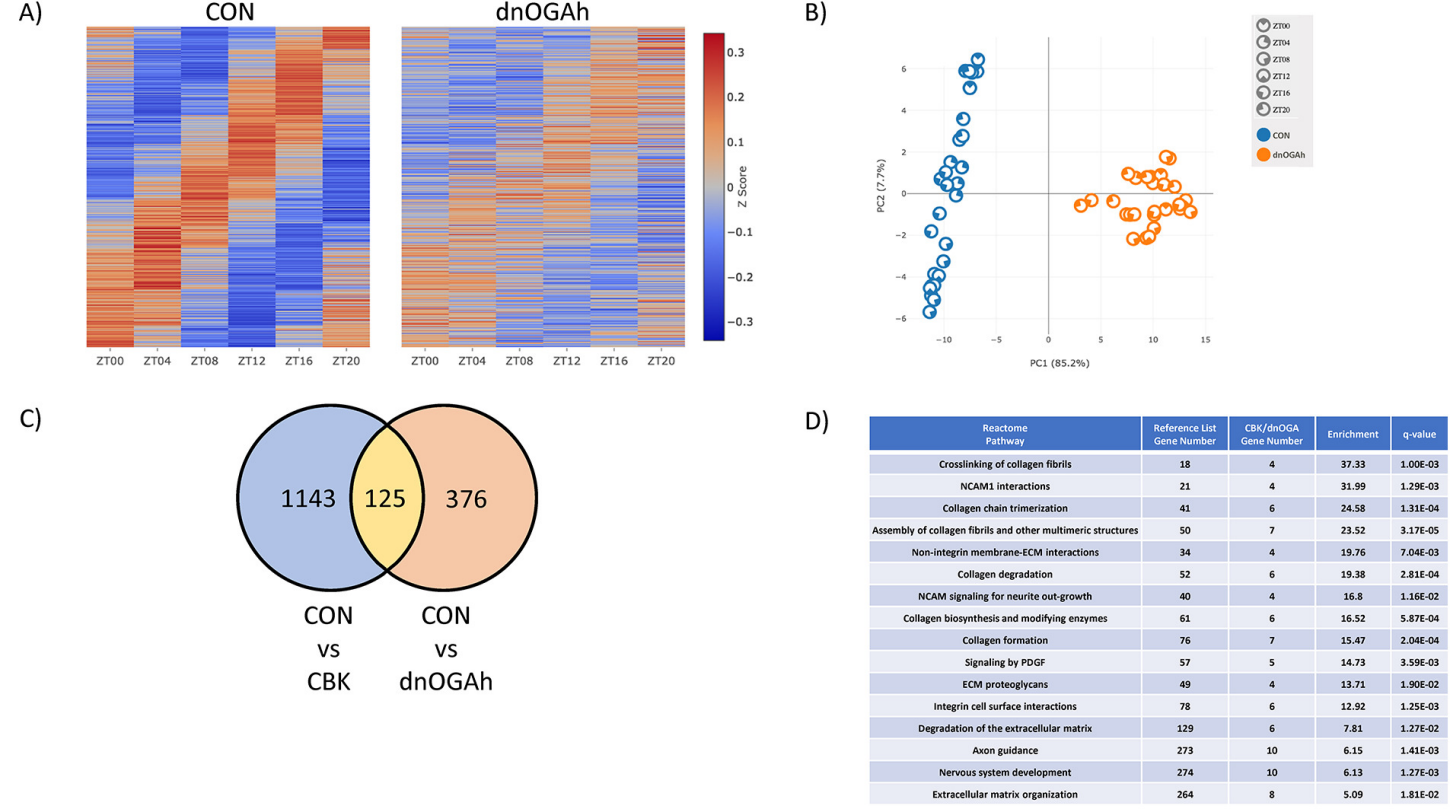
Impact of augmented protein O-GlcNAcylation on circadian governance of the cardiac transcriptome. Hearts were collected from dnOGAh and littermate CON mice at 4 h intervals over a 24 h period, 2 weeks after doxycycline treatment initiation, followed by RNAseq analysis (*n* = 5). **(A)** Heat map illustrating level of expression for genes exhibiting significant 24 h oscillations in CON hearts, that are either abolished or attenuated in dnOGAh hearts. **(B)** PCA plot for genes exhibiting significant 24 h oscillations in CON hearts, that are either abolished or attenuated in dnOGAh hearts. **(C)** Venn Diagram comparing differentially oscillating genes in CBK hearts vs. differentially oscillating genes in dnOGAh hearts. **(D)** Reactome pathway analysis results for oscillating genes that are commonly impacted in CBK and dnOGAh hearts.

Previous transcriptomic analyses indicate that the cardiomyocyte circadian clock orchestrates diurnal variations of approximately 5%–10% of the cardiac transcriptome (14, 26). Given that our candidate gene approach suggested that circadian clock output is attenuated in dnOGAh hearts (Figures 3B,4B), we next evaluated the diurnal transcriptome at a global level. More specifically, RNAseq was performed on RNA isolated from dnOGAh and littermate CON hearts collected at 4 h intervals over the 24 h day (2 weeks after doxycycline treatment initiation). JTK cycle identified 528 transcripts that significantly oscillate with a periodicity of 24 h in CON hearts; the number of oscillating transcripts was only 45 in dnOGA hearts (Supplementary Tables 5, 6, respectively). Further analysis revealed that 496 transcripts oscillate only in CON hearts, 13 transcripts oscillate only in dnOGA hearts, while 32 transcripts oscillate in both CON and dnOGA hearts (Supplementary Tables 7–9, respectively). Of the transcripts oscillating in both CON and dnOGA hearts, 5 had an amplitude that was significantly reduced in dnOGA hearts (Supplementary Table 9). As such, a total of 501 oscillating transcripts in CON hearts were either abolished or significantly attenuated in dnOGA hearts, representing 95% of all oscillating transcripts in CON hearts (Figure 5A). The impact of dnOGA induction on the diurnal transcriptome was further illustrated in a PCA plot; in addition to marked differential expression at the level of genotype, dnOGA hearts do not exhibit the same time-of-day-dependent pattern observed for CON hearts (Figure 5B). Comparing our previously published microarray analysis of CBK hearts (14) with the newly generated RNAseq analysis of dnOGAh hearts, we identified 125 transcripts exhibiting attenuated/abolished 24 h oscillations in both CBK and dnOGA hearts (Figure 5C; Supplementary Table 10). Pathway analysis of these 125 transcripts indicated notable enrichment in collagen and extracellular matrix (Figure 5D). Collectively, these data indicate that the diurnal transcriptome is markedly impaired in dnOGAh hearts.

### Interstitial fibrosis following disruption of the cardiac diurnal transcriptome

**Figure 6:**
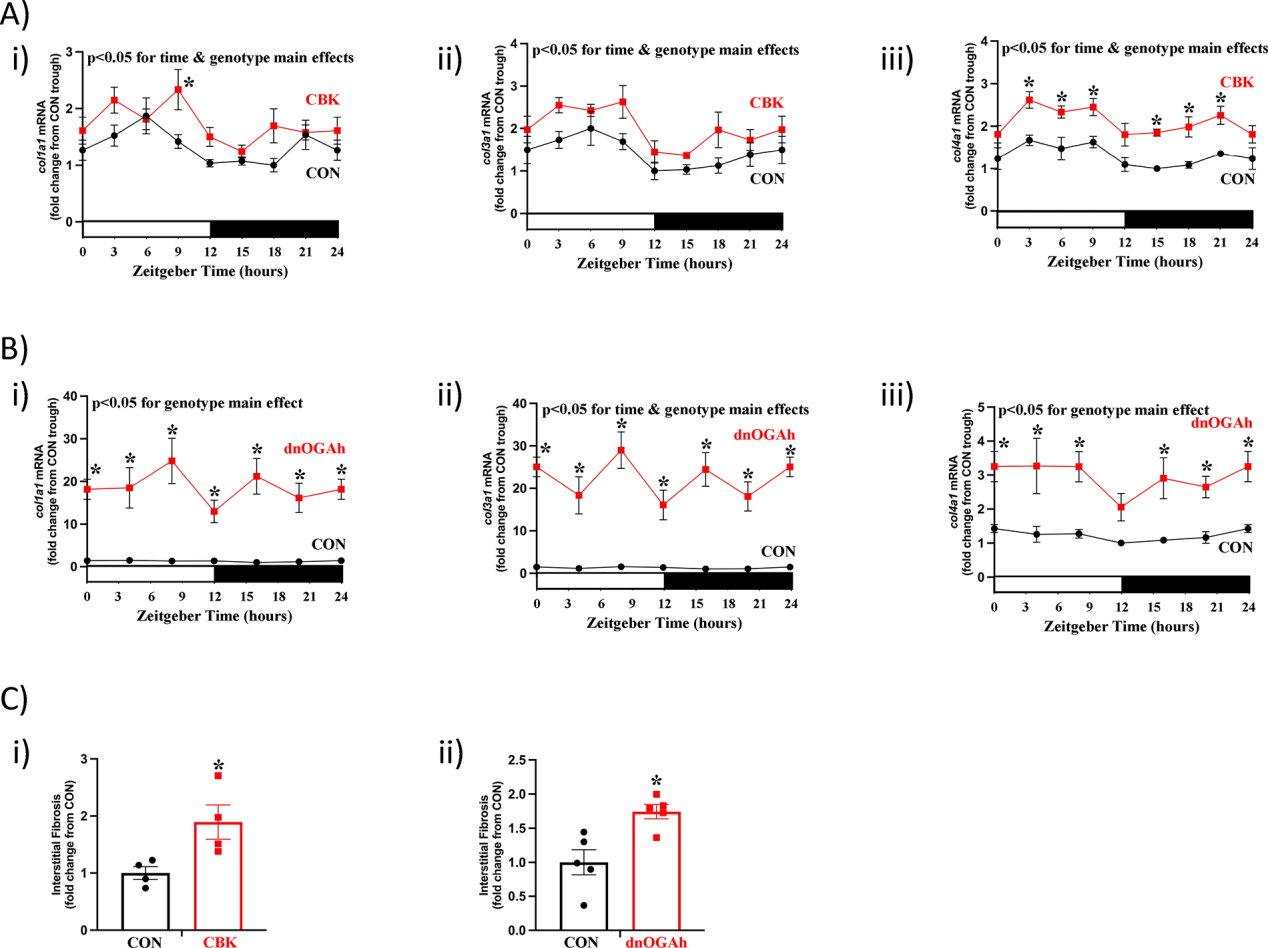
Increased interstitial fibrosis in CBK and dnOGA hearts. Hearts were collected either from CBK and littermate CON mice at 3 h intervals over a 24 h period, or from dnOGAh and littermate CON mice at 4 h intervals over a 24 h period (2 weeks after doxycycline treatment initiation), followed by RT-PCR and histologic analyses. **(A)** mRNA levels of collagen isoforms *col1a1* (i), *col3a1* (ii), and *col4a1* (iii) in CON and CBK hearts (*n* = 5−6). **(B)** mRNA levels of collagen isoforms *col1a1* (i), *col3a1* (ii), and *col4a1* (iii) in CON and dnOGA hearts (*n* = 5−9). **(C)** Interstitial fibrosis in CBK (i) and dnOGAh (ii) hearts compared to littermate CON hearts (*n* = 4−5). In panels A and B, ZT0 and ZT24 are identical (the data are double plotted purely for the sake of presentation). Please see Supplemental files for all original images. Data are presented as mean ± SEM, and have been normalized to the lowest (trough) value in CON hearts. Main effects of time and genotype are reported at the top of the figure panels. *, *p* < 0.05 for CON vs. CBK/dnOGAh hearts (at the same time-of-day).

As indicated in Figure 5D, multiple transcripts involved in the extracellular matrix exhibited attenuated/abolished 24hr oscillations in both CBK and dnOGAh hearts; these transcripts included several collagen isoforms (Supplementary Table 10). RT-PCR was next performed to validate 24 h patterns of *col1a1*, *col3a1*, and *col4a1* mRNA in CBK, dnOGAh, and littermate control hearts. A 2-way ANOVA revealed significant time main effects for *col1a1*, *col3a1*, and *col4a1* mRNA (Figure 6A). In addition, genotype main effects were observed for all 3 collagen isoforms, in both CBK and dnOGAh hearts; these 3 transcripts were increased in both models (Figures 6A,B). Importantly, consistent with increased collagen isoform expression, interstitial fibrosis was augmented in both CBK and dnOGAh hearts (Figure 6C). These observations suggest that increased protein O-GlcNAcylation and attenuated circadian governance are associated with cardiac fibrosis.

## Discussion

Aberrant protein O-GlcNAcylation is associated with cardiac pathology in both humans and animal models (2). Protein O-GlcNAcylation is elevated in the heart during diabetes mellitus, hypertrophy, and ischemia (6, 16, 27). Reducing this PTM within normal levels, through AAV-mediated OGA overexpression, normalized markers of adverse cardiac remodeling and diastolic dysfunction during diabetes (6). Similar observations have been reported in the setting of pressure overload; transgenic mice overexpressing OGA were resistant to transaortic constriction induced systolic dysfunction (16). Conversely, genetic augmentation of protein O-GlcNAcylation specifically in cardiomyocytes *in vivo* is sufficient to induce adverse cardiac remodeling and dysfunction, the severity of which is dependent on the manner by which this PTM is enhanced. More specifically, cardiac-specific OGT overexpression resulted in an approximate 6-fold increase in protein O-GlcNAcylation and heart failure (16), whereas dnOGAh mice exhibit more modest increase in O-GlcNAcylation (<2-fold) and diastolic dysfunction (without impact on systolic parameters) (7).

Despite appreciation that sustained augmentation of protein O-GlcNAcylation contributes towards the pathogenesis of cardiac disease, the mechanisms by which this occurs remain elusive. This likely reflects the large number of proteins that are O-GlcNAc modified, as well as technical limitations associated with both identification and manipulation of specific protein sites for this PTM. Interestingly, multiple similarities exist between aberrant protein O-GlcNAcylation and circadian clock dysfunction. The latter has been reported in multiple cardiac disease states (e.g., diabetes mellitus, pressure overload induced hypertrophy) (20, 28, 29), while genetic ablation of the cardiomyocyte circadian clock results in adverse cardiac remodeling and heart failure (14). Even in humans, perturbed circadian biology secondary to behaviors (e.g., shift work) and/or genetic polymorphisms in clock components, is associated with increased cardiovascular disease risk (30, 31). Much like protein O-GlcNAcylation, circadian clocks regulate a large number of cellular processes, making it difficult to define the precise mechanisms by which circadian disruption leads to cardiac disease.

Protein O-GlcNAcylation and circadian clocks are interconnected, a relationship that was first described in the heart. The cardiomyocyte circadian clock governs 24 h oscillations in cardiac glucose utilization, OGT expression, and protein O-GlcNAcylation levels, all of which peak during the middle of the active period (8). This includes augmented BMAL1 O-GlcNAcylation during the active period, which was later reported to enhance protein stability through inhibition of ubiquitination (8, 9). CLOCK and PER isoforms are also subjected to O-GlcNAcylation; this PTM increases CLOCK protein stability, and affects the subcellular localization of PER proteins (9, 12). Although REVERB*α* binds with OGT, this circadian clock component does not appear to be O-GlcNAc modified. Instead, REVERBα affects OGT subcellular localization, thereby promoting O-GlcNAcylation of nuclear proteins during periods when REVERBα levels are elevated (32).

The majority of previously published studies investigating the impact of protein O-GlcNAcylation on circadian clock function have been acute in nature (i.e., <24 h), *in vitro*, and/or in extra-cardiac tissues. Moreover, the impact of sustained augmentation of protein O-GlcNAcylation on circadian governance of cellular processes is lacking. To gain greater insight, the purpose of the present study was to determine whether a modest elevation of protein O-GlcNAcylation in the adult heart for a prolonged period of time (2 weeks) influenced either the cardiac circadian clock or circadian governance of the cardiac transcriptome. Here, we report that an approximate 1.5-fold increase in protein O-GlcNAcylation in dnOGAh hearts impacted mRNA levels of 7 out of 10 circadian clock components (Figure 2). Consistent with aforementioned previously published studies reporting the impact of O-GlcNAcylation on stability of clock component proteins, BMAL1 and PER2 protein levels were augmented in dnOGAh hearts (to an extent that was greater than changes in mRNA levels), while REVERBα protein levels were unaffected (Figure 2B; Supplementary Table 3). Importantly, circadian governance was severely attenuated, at both the candidate gene level (Figures 3B,4B) and whole transcriptome level (Figures 5A,B). In the latter case, 95% of oscillating transcripts in control hearts were either abolished or significantly attenuated in dnOGAh hearts. Such observations not only underscore the importance of protein O-GlcNAcylation in the operation of circadian clock mechanism, but also highlight the scale by which this PTM affects clock output and temporal control of the transcriptome. With regards to potential functional consequences of aberrant circadian governance, comparison of transcriptomic data from dnOGAh and CBK hearts indicated that the extracellular matrix was commonly impacted in both models (Figure 5D), which was confirmed through RT-PCR and histology (Figure 6).

The present study has led to a number of subsequent questions, including those related to identification of the mechanisms by which circadian governance over the cardiac transcriptome is so dramatically abolished by sustained protein O-GlcNAcylation. Of the 10 circadian clock components investigated at the mRNA level, only *clock* was non-rhythmic in dnOGA hearts (Supplementary Table 4). At the protein level, 24 h oscillations in circadian clock components were either unaffected (i.e., REVERBα) or enhanced (BMAL1 and PER2; *p* = 0.11 and *p* < 0.05, respectively) in dnOGAh hearts (Figure 2B). This is in striking contrast to CBK hearts, for which 24 h oscillations in clock components were largely abolished/attenuated (Figures 1A,B). These observations suggest a potential uncoupling of the core clock mechanism from circadian governance of the transcriptome following a 2-week elevation of protein O-GlcNAcylation. Several possible explanations exist, including influences on direct clock output genes that serve as mechanistic links from the clock to cellular processes. The PAR transcription factors DBP, HLF, and TEF are excellent examples. At the mRNA, *dbp* and *tef* were reduced in dnOGAh hearts, while an antagonist to these transcription factors, E4BP4, was increased at both mRNA and protein levels (Figures 3B,C; Supplementary Table 3). Interestingly, E4BP4 protein levels were augmented in dnOGAh hearts to a greater extent compared with *e4bp4* mRNA, suggesting potential posttranscriptional regulation (Figures 3B,C; Supplementary Table 3). Recent studies indicate that E4BP4 links the cardiomyocyte circadian clock with cardiac metabolism and electrophysiology (33). Moreover, chronic elevations in E4BP4 levels in CBK hearts play a causal role in adverse remodeling and cardiac dysfunction. More specifically, pharmacologic and genetic attenuation of E4BP4 in CBK hearts reduces cardiac fibrosis and heart failure development (33, 34). Whether elevated E4BP4 levels in dnOGAh hearts (Figure 3C) promotes fibrosis (Figure 6) and previously reported contractile dysfunction (7) requires further investigation.

It is noteworthy that we and others have previously reported that approximately 10% of the cardiac transcriptome oscillates with a periodicity of 24 h in wild-type control hearts (35). However, the number of oscillating transcripts identified for CON hearts in the current dnOGAh RNAseq analysis was considerably lower. This is potentially secondary to two experimental factors. First, dnOGAh and littermate CON mice were both treated with doxycycline (initially injection, followed by ingestion) for 2 weeks. Doxycycline has established effects on the microbiome, as well as biological processes in the host (including metabolism) (36, 37). Interestingly, previously published studies have reported that antibiotic-mediated perturbations in the microbiome impacts both the heart and circadian clocks (38). Second, a higher time resolution was utilized for our previously published microarray studies (3 h intervals; 8 time points) compared to the current RNAseq analysis in dnOGA and littermate CON hearts (4 h intervals; 6 time points) (14). An additional limitation of the bulk RNA approach employed by our microarray and RNAseq analyses is that they fail to consider the heterogeneity of the heart. The possibility exists that O-GlcNAcylation phase shifts the cardiomyocyte clock in dnOGAh hearts, whereas the clock remains relatively intact in non-cardiomyocytes. Such an intercellular dyssynchrony would be perceived as attenuated/abolished 24 h oscillations when RNA is pooled from all cell types in the heart (i.e., bulk methods). Finally, the current study investigated diurnal variations, as opposed to circadian rhythms. Future studies should be performed under circadian conditions (e.g., constant darkness), preventing any potential masking effects of light-dark cycles.

In summary, the present study has revealed that a modest increase in protein O-GlcNAcylation for a 2-week period in adult hearts is sufficient to severely attenuate circadian governance of the cardiac transcriptome, in a manner that appears to be distinct from circadian clock disruption. This abolishment of circadian governance in the heart was associated with increased interstitial fibrosis. Future studies are required to define the precise mechanisms by which protein O-GlcNAcylation impairs circadian governance, and whether this plays a role in the etiology of cardiac disease during diabetes mellitus, pressure overload, and/or ischemia.

## Data Availability

The original contributions presented in the study are publicly available. This data can be found here: https://www.ncbi.nlm.nih.gov/geo/query/acc.cgi?acc=GSE301657.

## Supplementary material

Supplementary Figure 1 Diurnal variations in whole-body energy balance in CBK (A) and dnOGA (B) mice. CBK, dnOGA, and littermate CON mice were placed in CLAMS cages, followed by continuous non-invasive assessment of energy expenditure, food intake, and physical activity. In all panels, ZT0 and ZT24 are identical (the data are double plotted purely for the sake of presentation). Data are presented as mean ± SEM, for 5–7 independent observations. Main effects of time are reported at the top of the figure panels.

Supplementary Figure 2 Diurnal variations in hepatic gene expression in dnOGA mice. Livers were collected from dnOGAh and littermate CON mice at 4hr intervals over a 24 h period, 2 weeks after doxycycline treatment initiation, followed by RT-PCR analyses. mRNA levels of bmal1 (i), reverba (ii), and dbp (iii) in CON and dnOGAh livers (*n* = 5–9). ZT0 and ZT24 are identical (the data are double plotted purely for the sake of presentation). Data are presented as mean ± SEM, and have been normalized to the lowest (trough) value in CON hearts. Main effects of time are reported at the top of the figure panels.
