# Supplementary material for: Severe attenuation of circadian clock output in the heart following sustained augmentation of cardiomyocyte protein O-GlcNAcylation
Source: Front Cardiovasc Med. 2025 Jul 17;12:1601407. doi: 10.3389/fcvm.2025.1601407 (PMC12310464; doi:10.3389/fcvm.2025.1601407)
Supplement: Supplementary Figure 1 — Diurnal variations in whole-body energy balance in CBK (A) and dnOGA (B) mice. CBK, dnOGA, and littermate CON mice were placed in CLAMS cages, followed by continuous non-invasive assessment of energy expenditure, food intake, and physical activity. In all panels, ZT0 and ZT24 are identical (the data are double plotted purely for the sake of presentation). Data are presented as mean ± SEM, for 5–7 independent observations. Main effects of time are reported at the top of the figure panels. [file Presentation1.pptx]

## Slide 1
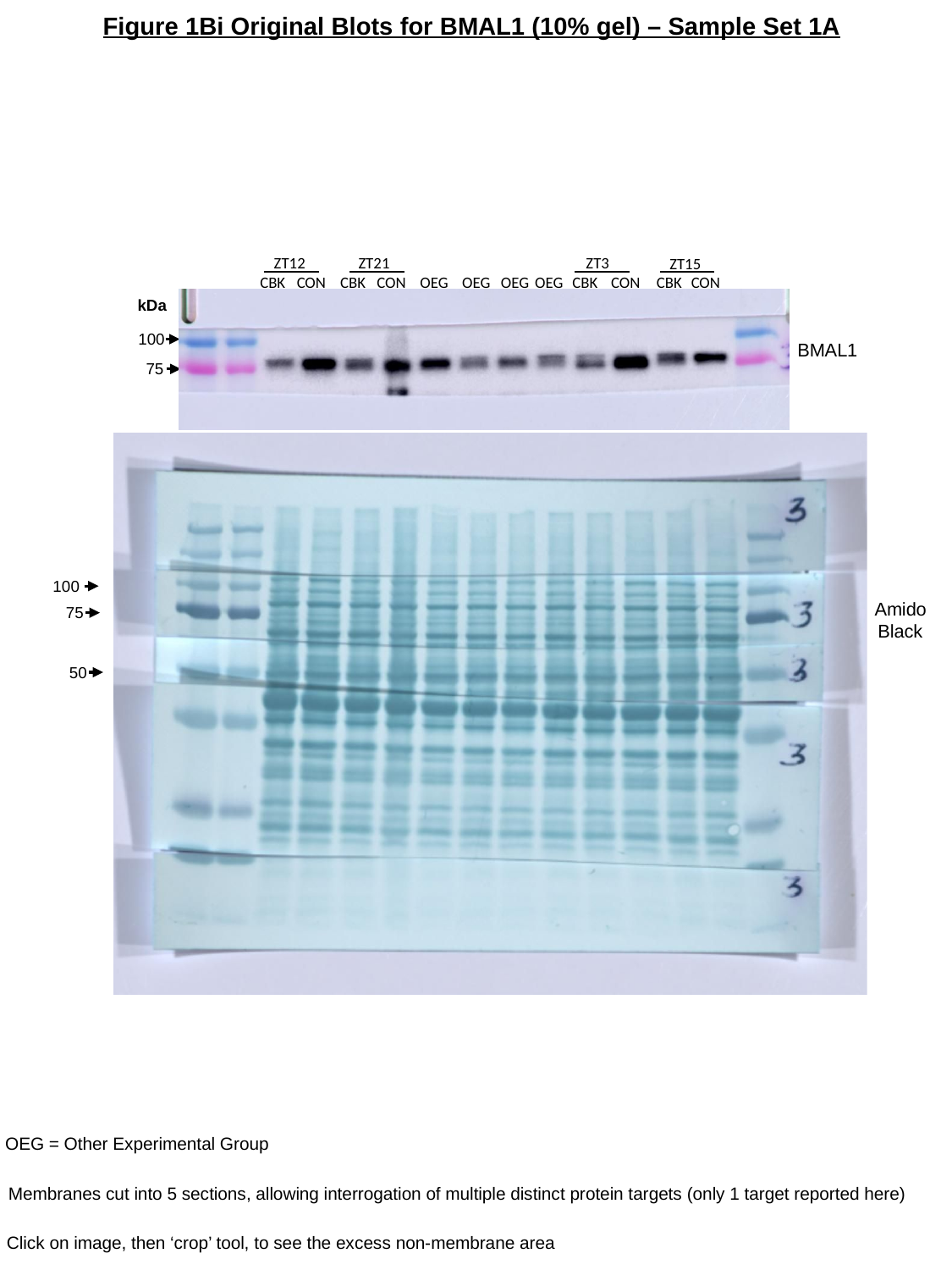

Figure 1Bi Original Blots for BMAL1 (10% gel) – Sample Set 1A
ZT3
ZT12
ZT21
ZT15
CBK
CON
CBK
CON
OEG
OEG
OEG
OEG
CBK
CON
CBK
CON
kDa
100
BMAL1
75
100
Amido
Black
75
50
OEG = Other Experimental Group
Membranes cut into 5 sections, allowing interrogation of multiple distinct protein targets (only 1 target reported here)
Click on image, then ‘crop’ tool, to see the excess non-membrane area

## Slide 2
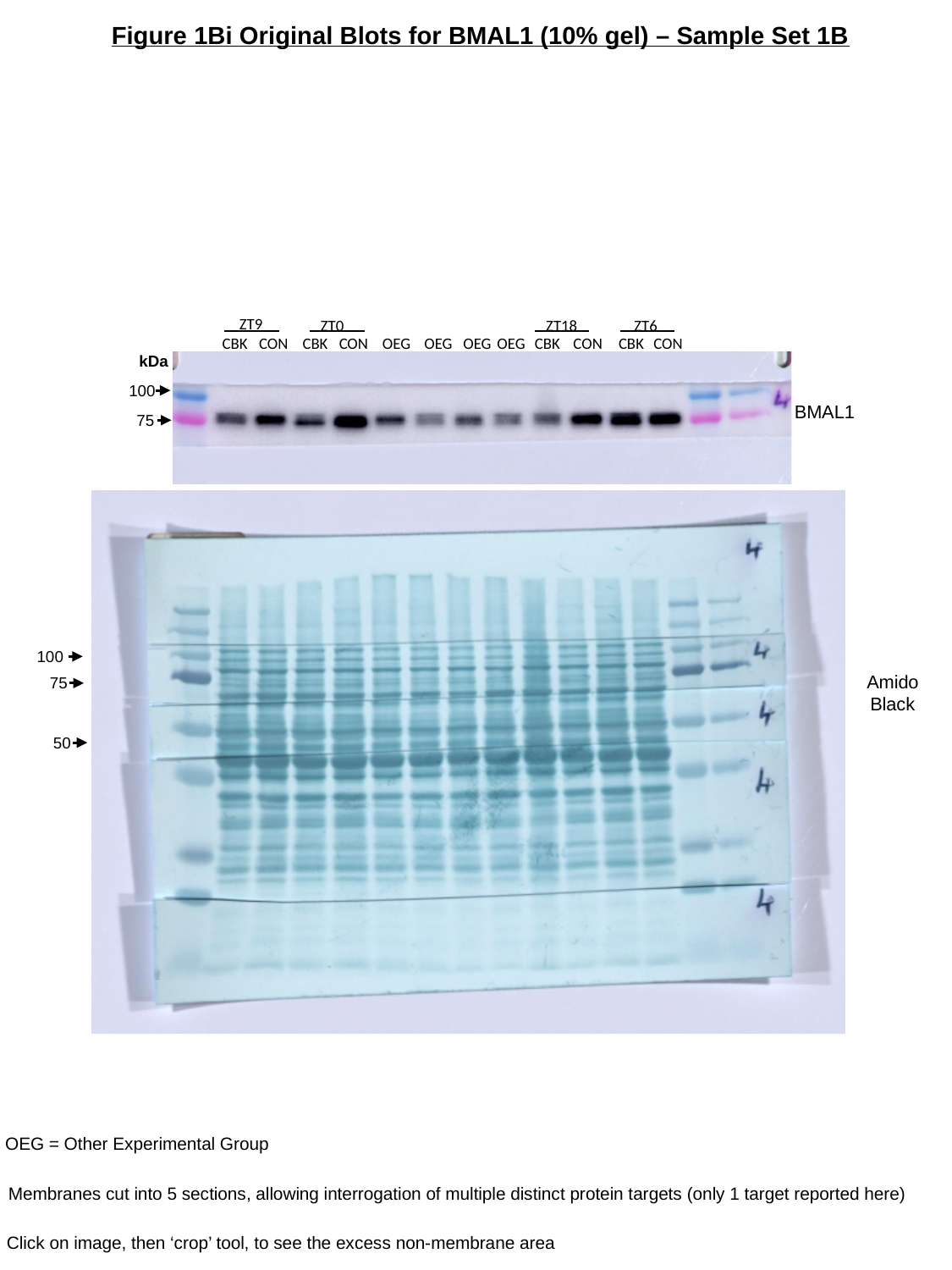

Figure 1Bi Original Blots for BMAL1 (10% gel) – Sample Set 1B
ZT9
ZT0
ZT18
ZT6
CBK
CON
CBK
CON
OEG
OEG
OEG
OEG
CBK
CON
CBK
CON
kDa
100
BMAL1
75
100
Amido
Black
75
50
OEG = Other Experimental Group
Membranes cut into 5 sections, allowing interrogation of multiple distinct protein targets (only 1 target reported here)
Click on image, then ‘crop’ tool, to see the excess non-membrane area

## Slide 3
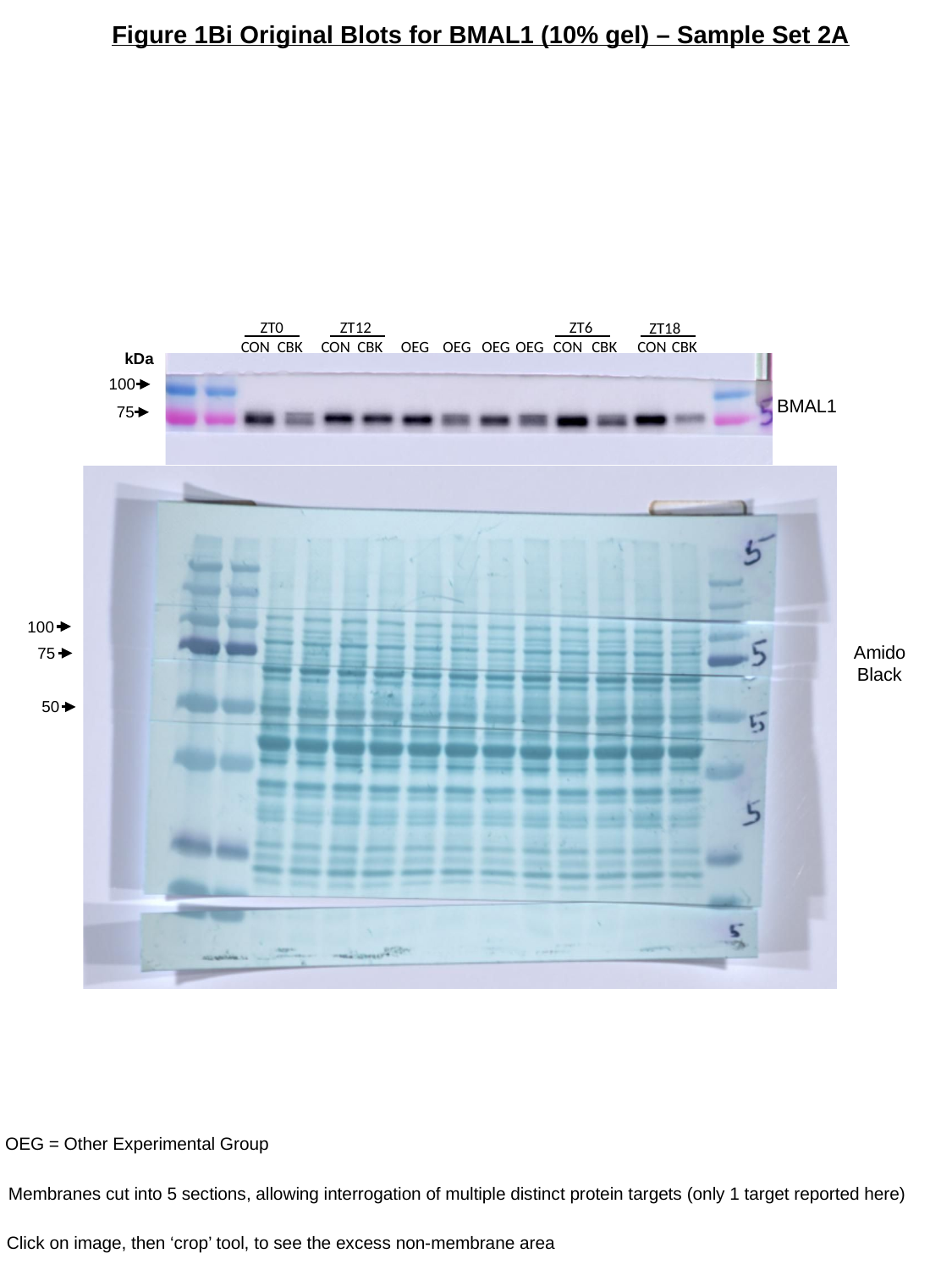

Figure 1Bi Original Blots for BMAL1 (10% gel) – Sample Set 2A
ZT6
ZT0
ZT12
ZT18
CON
CBK
CON
CBK
OEG
OEG
OEG
OEG
CON
CBK
CON
CBK
kDa
100
BMAL1
75
100
Amido
Black
75
50
OEG = Other Experimental Group
Membranes cut into 5 sections, allowing interrogation of multiple distinct protein targets (only 1 target reported here)
Click on image, then ‘crop’ tool, to see the excess non-membrane area

## Slide 4
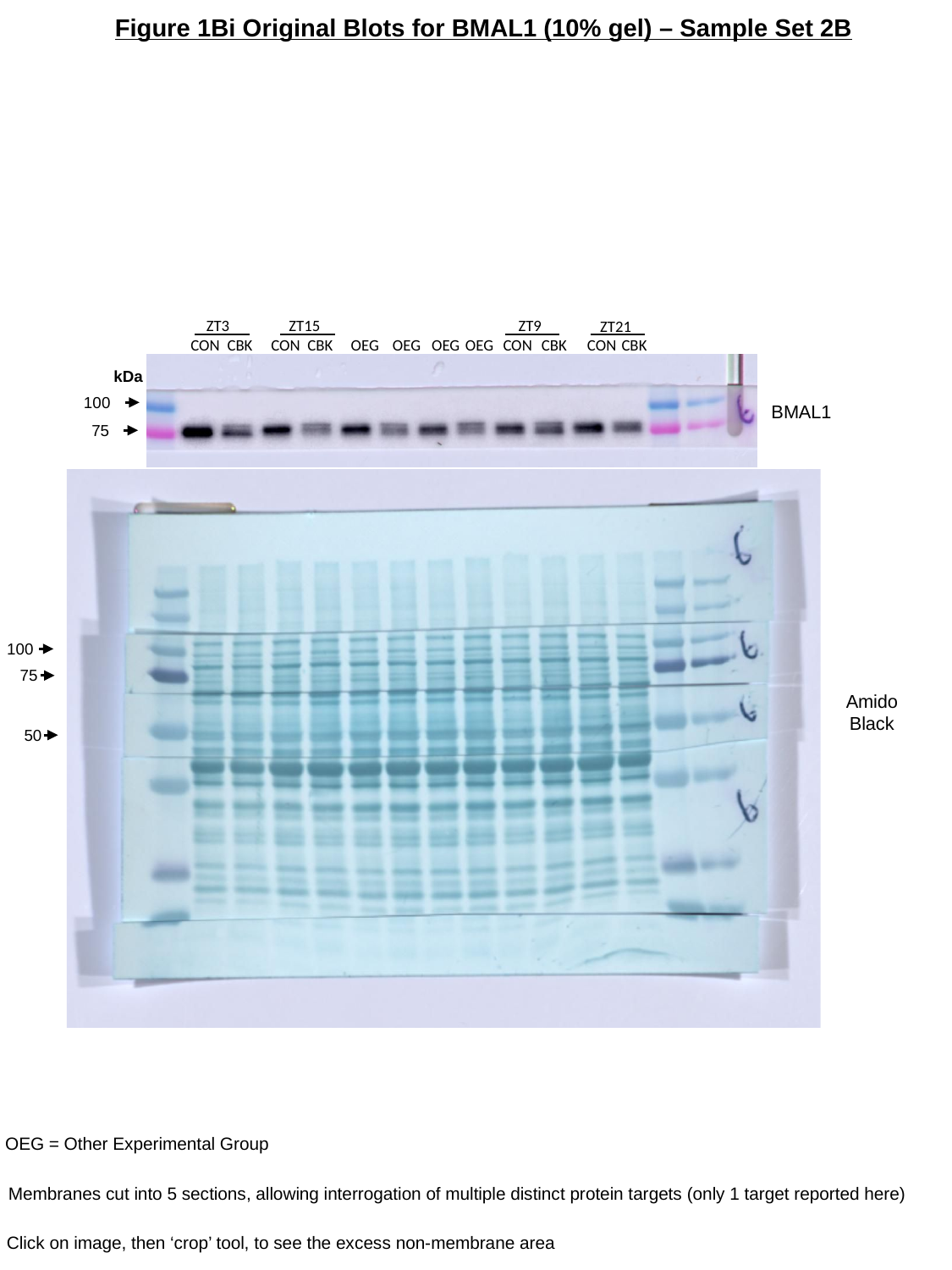

Figure 1Bi Original Blots for BMAL1 (10% gel) – Sample Set 2B
ZT9
ZT3
ZT15
ZT21
CON
CBK
CON
CBK
OEG
OEG
OEG
OEG
CON
CBK
CON
CBK
kDa
100
BMAL1
75
100
75
Amido
Black
50
OEG = Other Experimental Group
Membranes cut into 5 sections, allowing interrogation of multiple distinct protein targets (only 1 target reported here)
Click on image, then ‘crop’ tool, to see the excess non-membrane area

## Slide 5
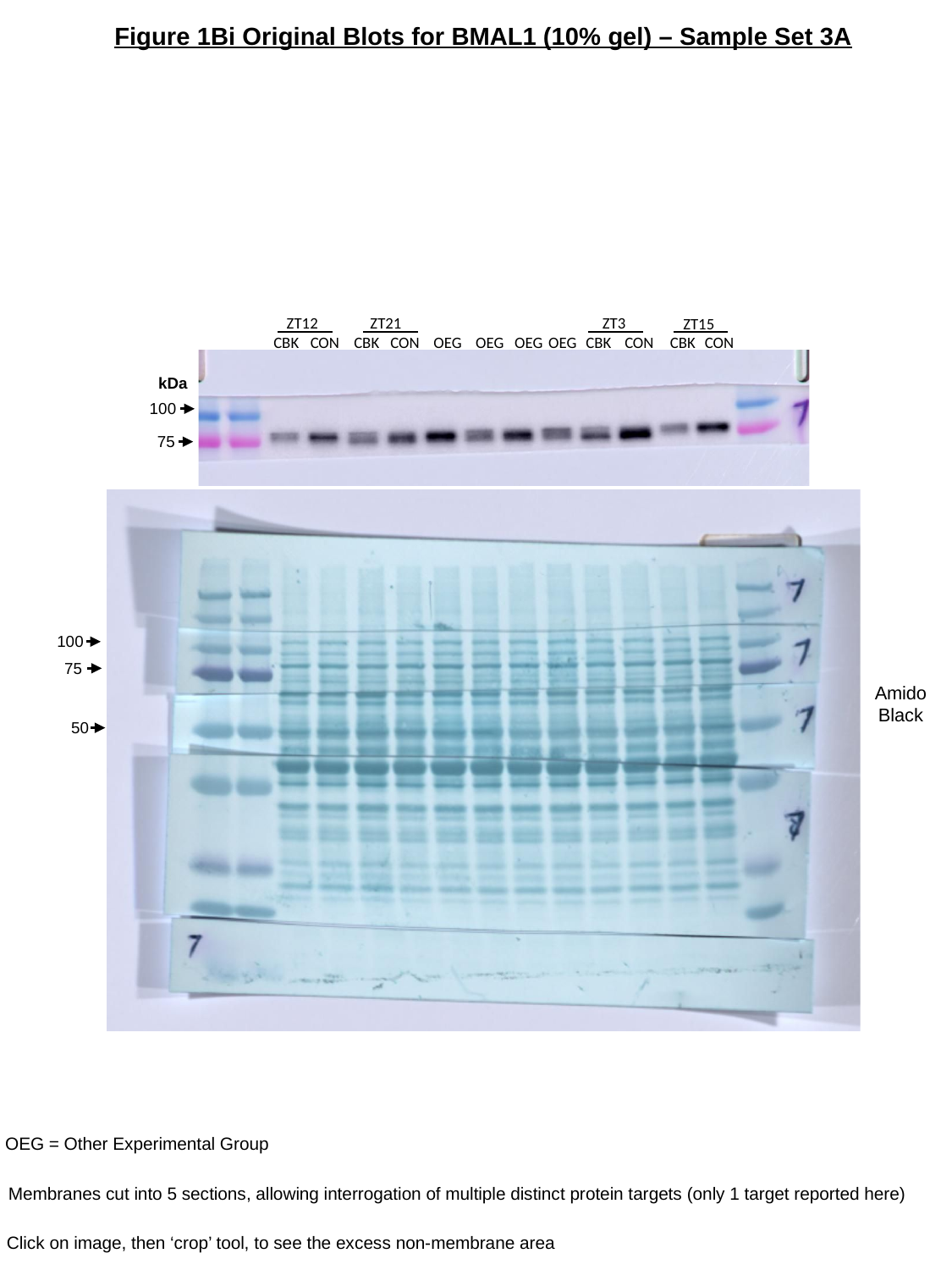

Figure 1Bi Original Blots for BMAL1 (10% gel) – Sample Set 3A
ZT3
ZT12
ZT21
ZT15
CBK
CON
CBK
CON
OEG
OEG
OEG
OEG
CBK
CON
CBK
CON
kDa
100
75
100
75
Amido
Black
50
OEG = Other Experimental Group
Membranes cut into 5 sections, allowing interrogation of multiple distinct protein targets (only 1 target reported here)
Click on image, then ‘crop’ tool, to see the excess non-membrane area

## Slide 6
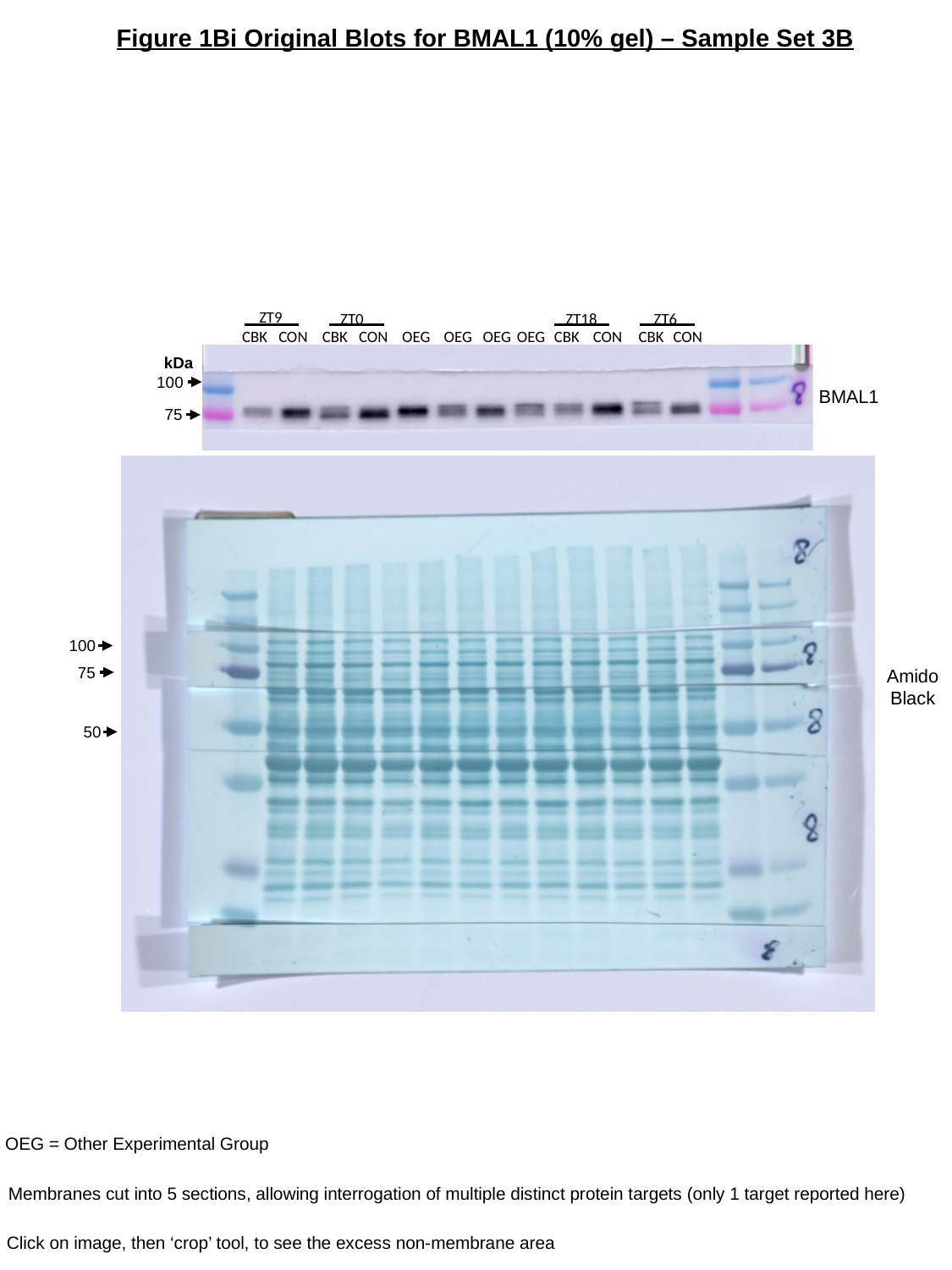

Figure 1Bi Original Blots for BMAL1 (10% gel) – Sample Set 3B
ZT9
ZT0
ZT18
ZT6
CBK
CON
CBK
CON
OEG
OEG
OEG
OEG
CBK
CON
CBK
CON
kDa
100
BMAL1
75
100
75
Amido
Black
50
OEG = Other Experimental Group
Membranes cut into 5 sections, allowing interrogation of multiple distinct protein targets (only 1 target reported here)
Click on image, then ‘crop’ tool, to see the excess non-membrane area

## Slide 7
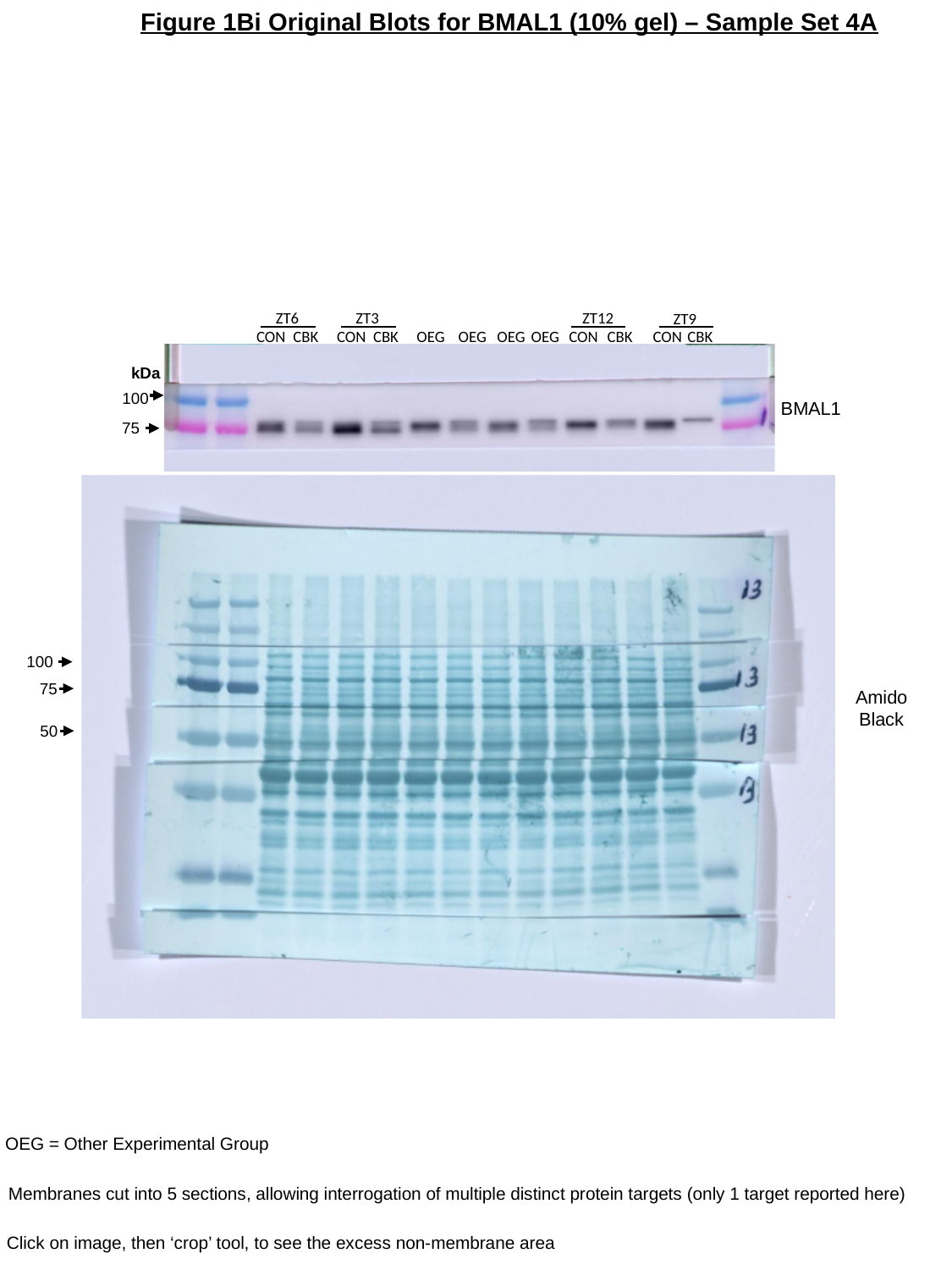

Figure 1Bi Original Blots for BMAL1 (10% gel) – Sample Set 4A
ZT12
ZT6
ZT3
ZT9
CON
CBK
CON
CBK
OEG
OEG
OEG
OEG
CON
CBK
CON
CBK
kDa
100
BMAL1
75
100
75
Amido
Black
50
OEG = Other Experimental Group
Membranes cut into 5 sections, allowing interrogation of multiple distinct protein targets (only 1 target reported here)
Click on image, then ‘crop’ tool, to see the excess non-membrane area

## Slide 8
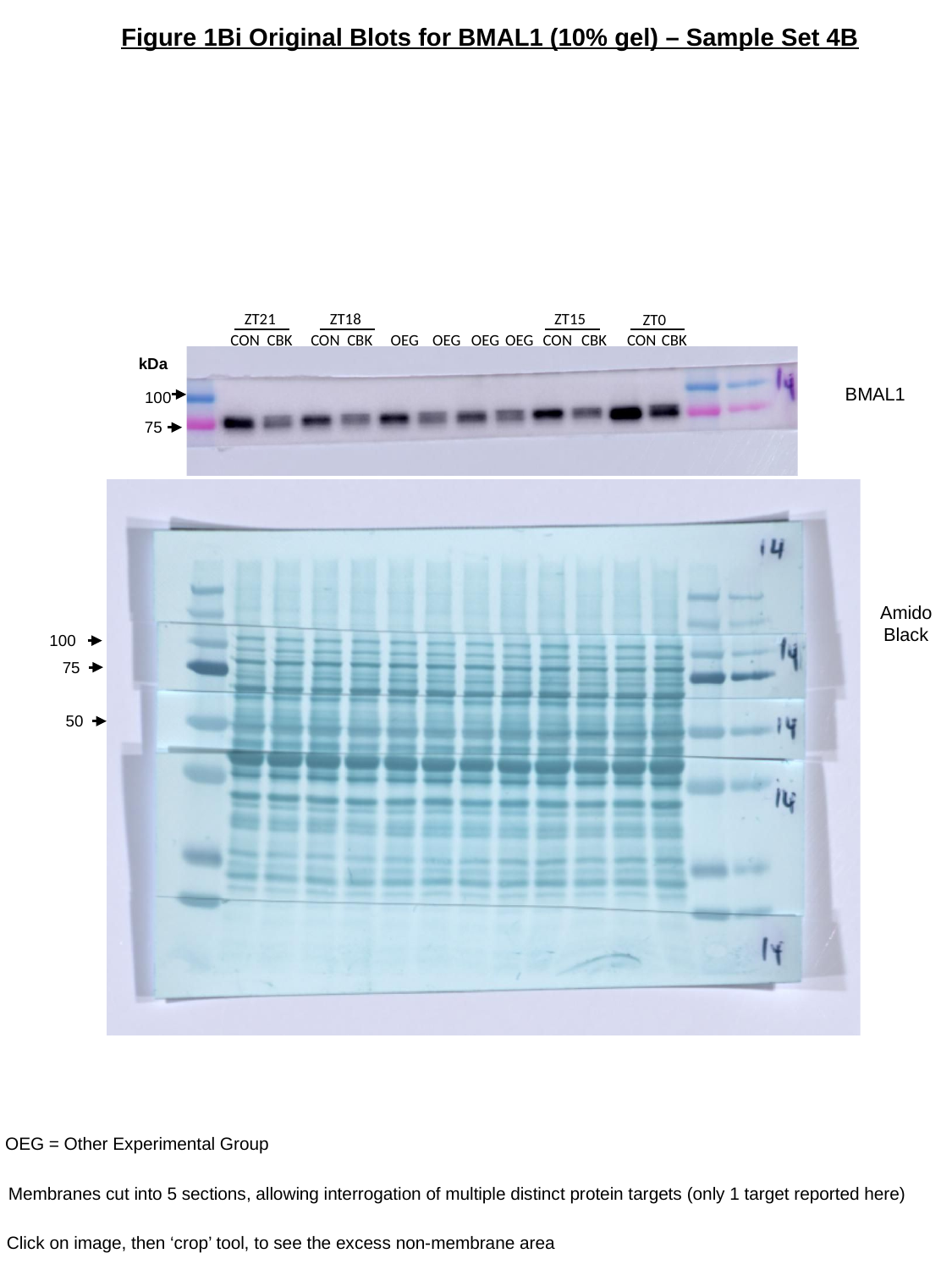

Figure 1Bi Original Blots for BMAL1 (10% gel) – Sample Set 4B
ZT15
ZT21
ZT18
ZT0
CON
CBK
CON
CBK
OEG
OEG
OEG
OEG
CON
CBK
CON
CBK
kDa
BMAL1
100
75
Amido
Black
100
75
50
OEG = Other Experimental Group
Membranes cut into 5 sections, allowing interrogation of multiple distinct protein targets (only 1 target reported here)
Click on image, then ‘crop’ tool, to see the excess non-membrane area

## Slide 9
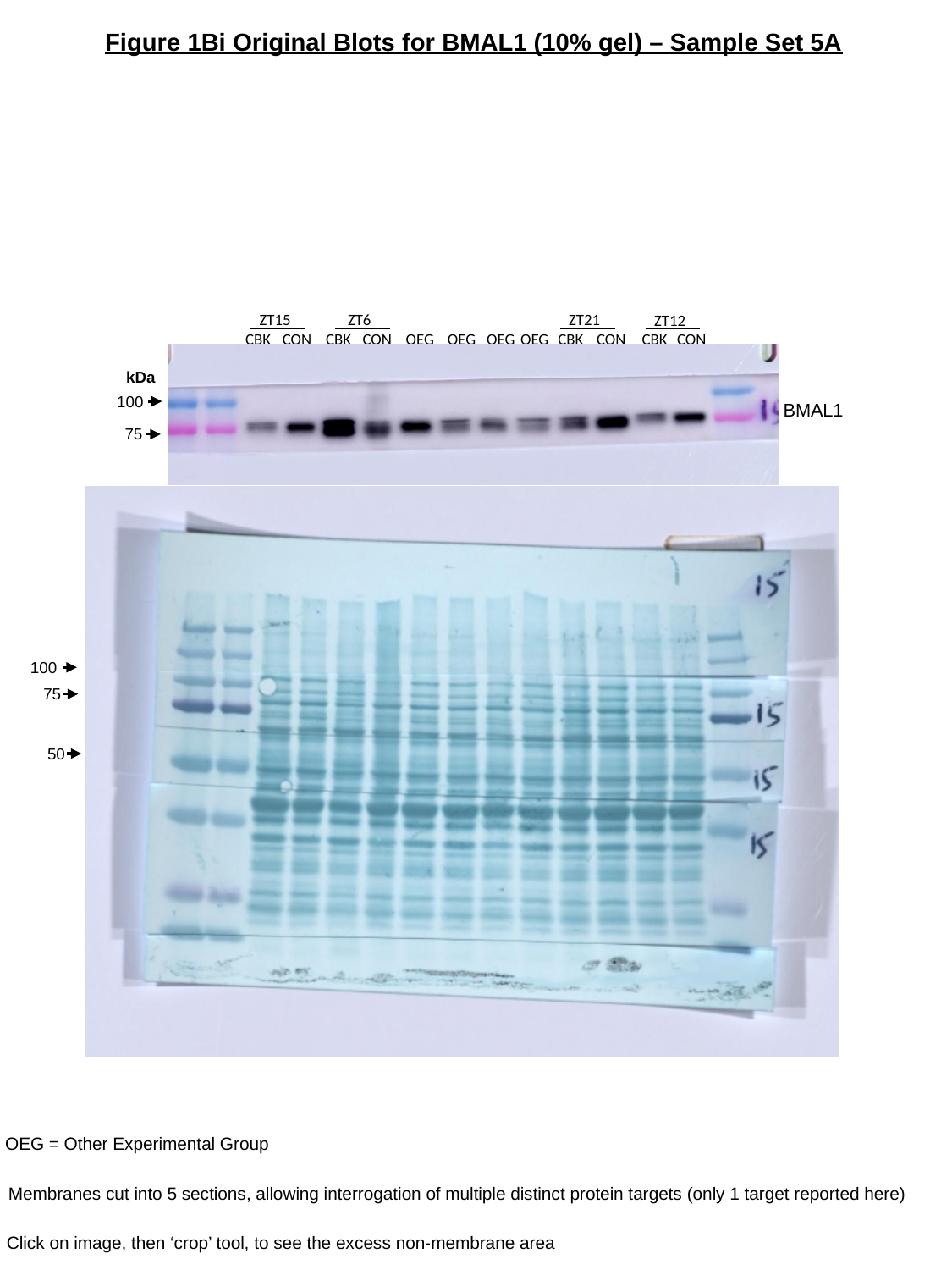

Figure 1Bi Original Blots for BMAL1 (10% gel) – Sample Set 5A
ZT21
ZT15
ZT6
ZT12
CBK
CON
CBK
CON
OEG
OEG
OEG
OEG
CBK
CON
CBK
CON
kDa
100
BMAL1
75
100
75
50
OEG = Other Experimental Group
Membranes cut into 5 sections, allowing interrogation of multiple distinct protein targets (only 1 target reported here)
Click on image, then ‘crop’ tool, to see the excess non-membrane area

## Slide 10
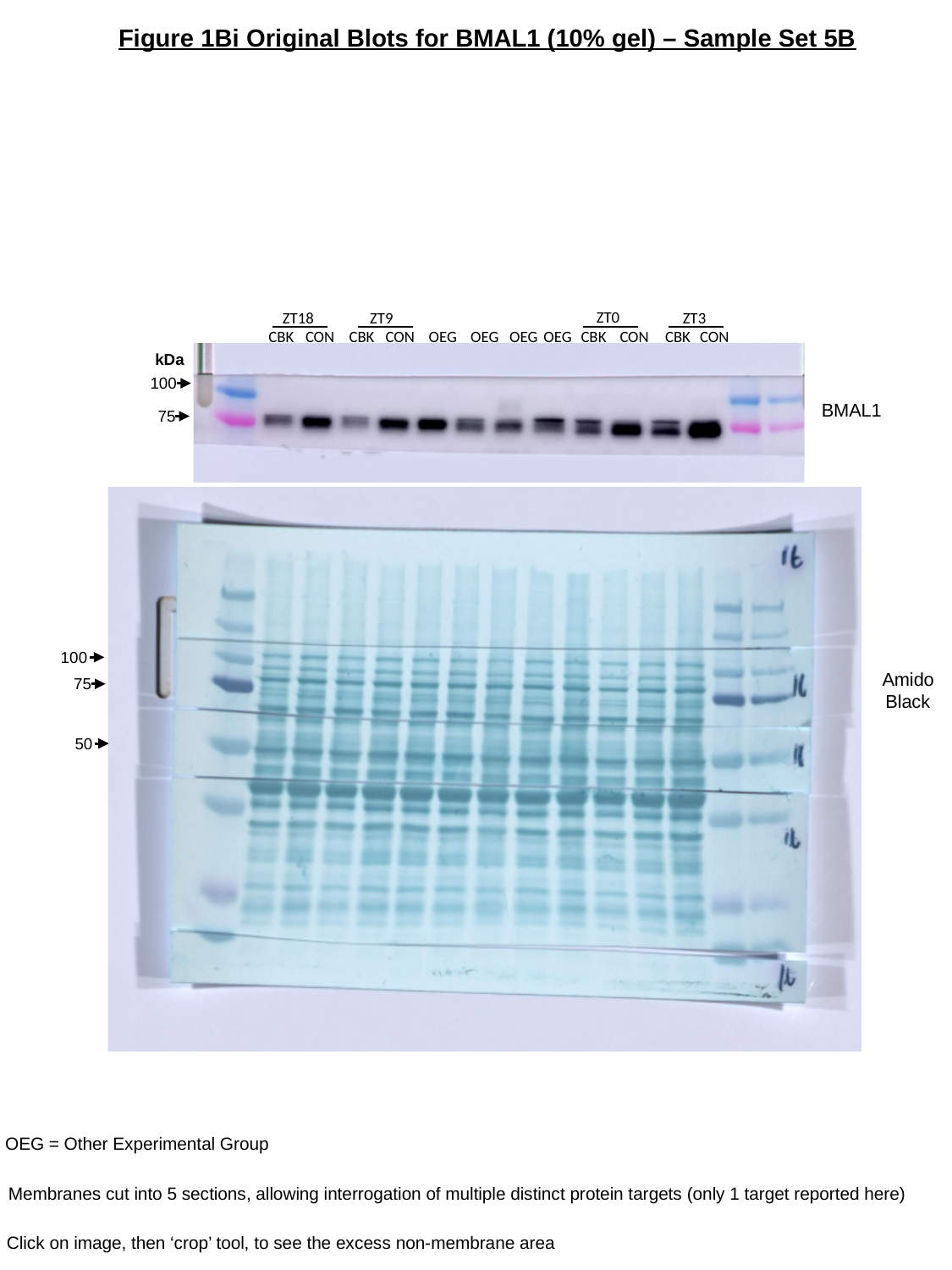

Figure 1Bi Original Blots for BMAL1 (10% gel) – Sample Set 5B
ZT0
ZT18
ZT9
ZT3
CBK
CON
CBK
CON
OEG
OEG
OEG
OEG
CBK
CON
CBK
CON
kDa
100
BMAL1
75
100
Amido
Black
75
50
OEG = Other Experimental Group
Membranes cut into 5 sections, allowing interrogation of multiple distinct protein targets (only 1 target reported here)
Click on image, then ‘crop’ tool, to see the excess non-membrane area

## Slide 11
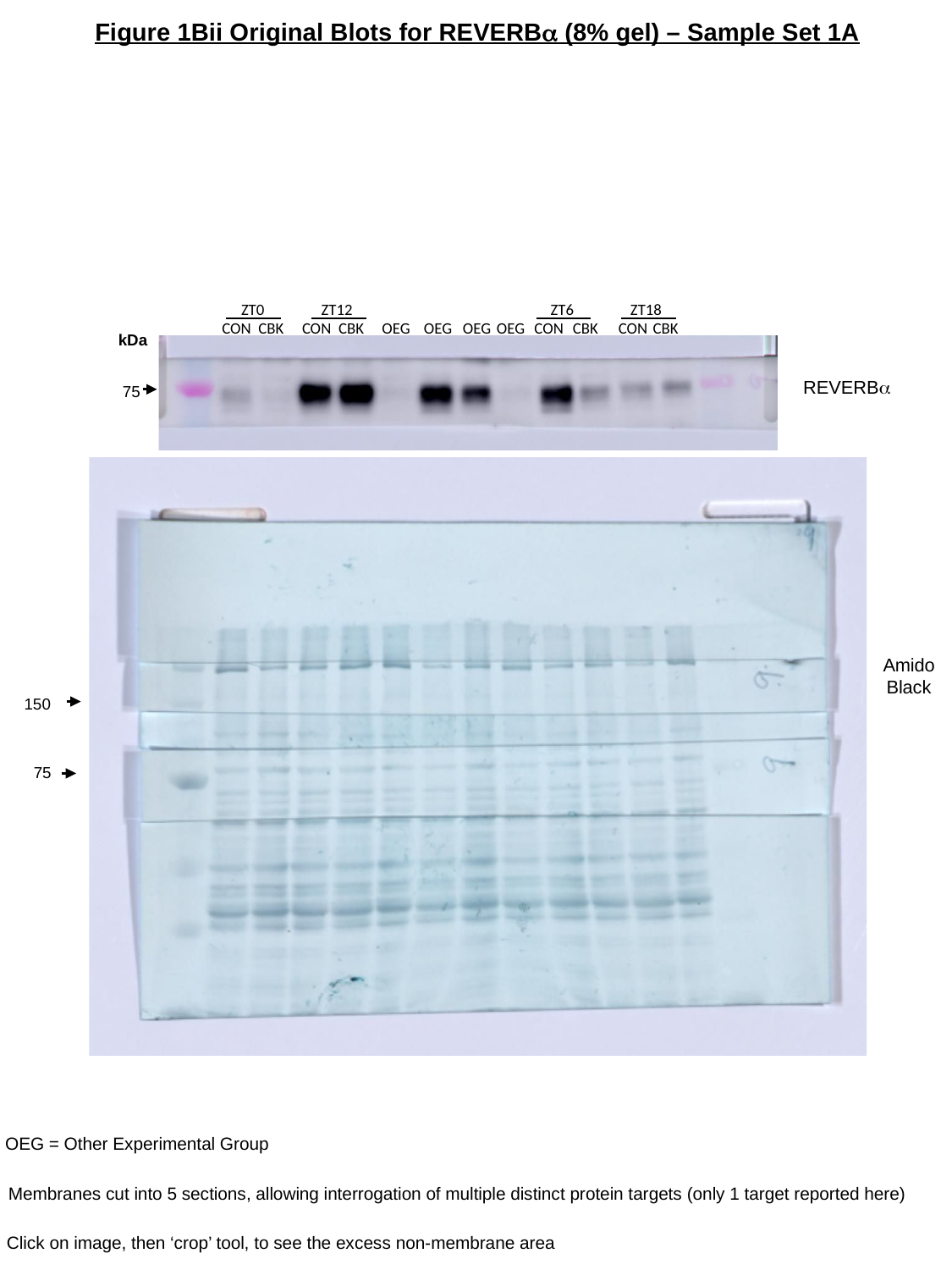

Figure 1Bii Original Blots for REVERBa (8% gel) – Sample Set 1A
ZT6
ZT0
ZT12
ZT18
CON
CBK
CON
CBK
OEG
OEG
OEG
OEG
CON
CBK
CON
CBK
kDa
REVERBa
75
Amido
Black
150
75
OEG = Other Experimental Group
Membranes cut into 5 sections, allowing interrogation of multiple distinct protein targets (only 1 target reported here)
Click on image, then ‘crop’ tool, to see the excess non-membrane area

## Slide 12
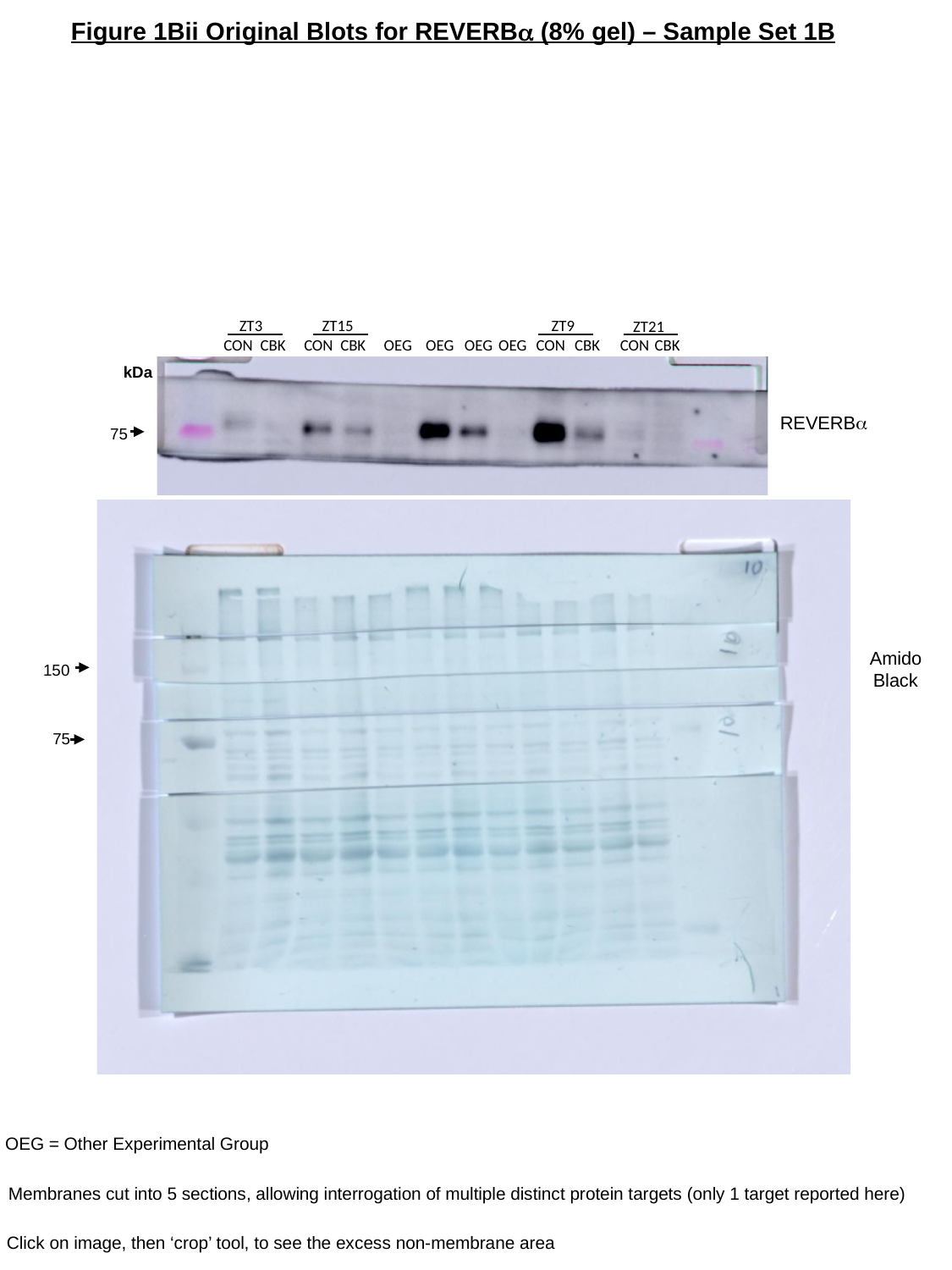

Figure 1Bii Original Blots for REVERBa (8% gel) – Sample Set 1B
ZT9
ZT3
ZT15
ZT21
CON
CBK
CON
CBK
OEG
OEG
OEG
OEG
CON
CBK
CON
CBK
kDa
REVERBa
75
Amido
Black
150
75
OEG = Other Experimental Group
Membranes cut into 5 sections, allowing interrogation of multiple distinct protein targets (only 1 target reported here)
Click on image, then ‘crop’ tool, to see the excess non-membrane area

## Slide 13
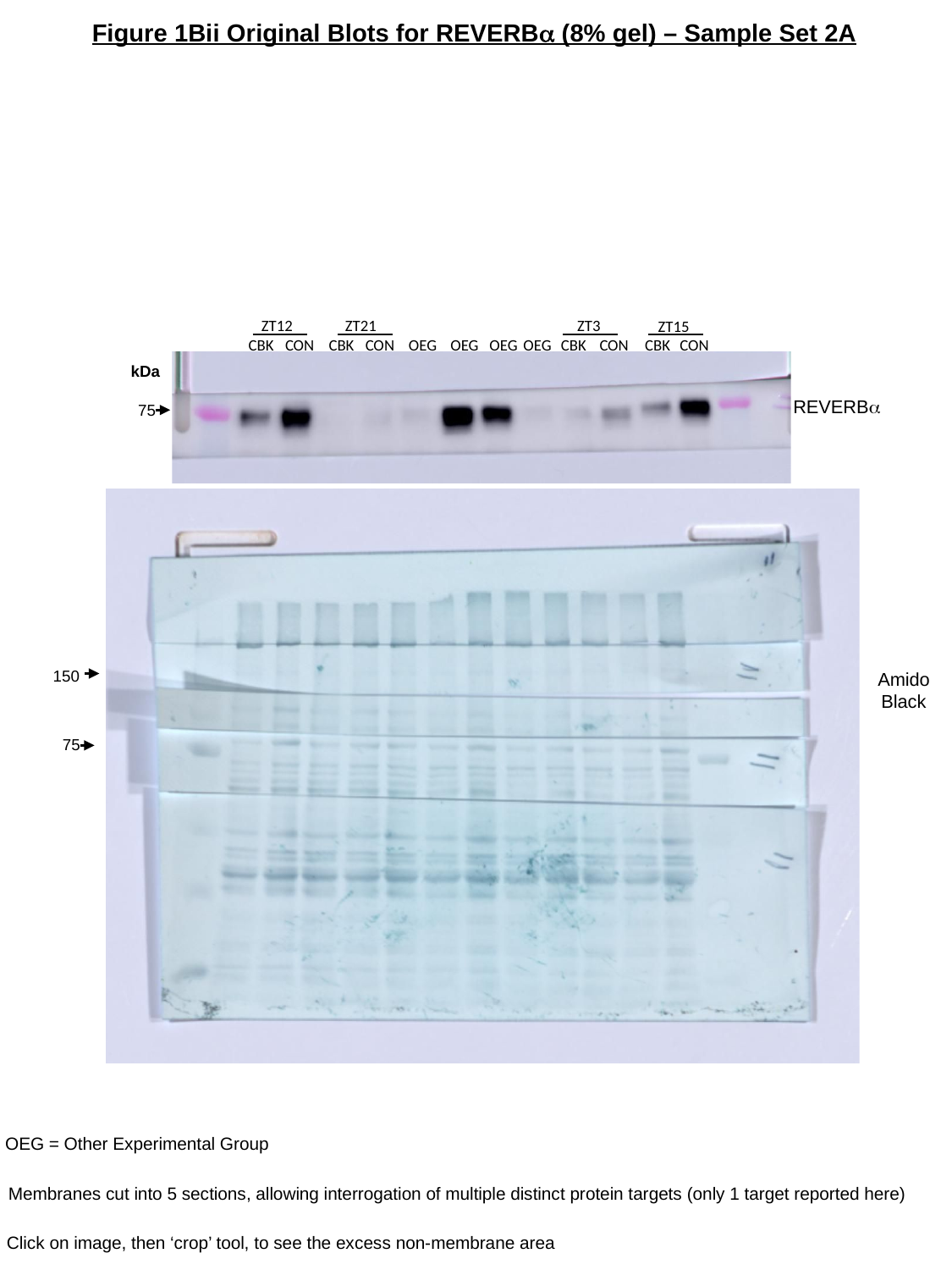

Figure 1Bii Original Blots for REVERBa (8% gel) – Sample Set 2A
ZT3
ZT12
ZT21
ZT15
CBK
CON
CBK
CON
OEG
OEG
OEG
OEG
CBK
CON
CBK
CON
kDa
REVERBa
75
150
Amido
Black
75
OEG = Other Experimental Group
Membranes cut into 5 sections, allowing interrogation of multiple distinct protein targets (only 1 target reported here)
Click on image, then ‘crop’ tool, to see the excess non-membrane area

## Slide 14
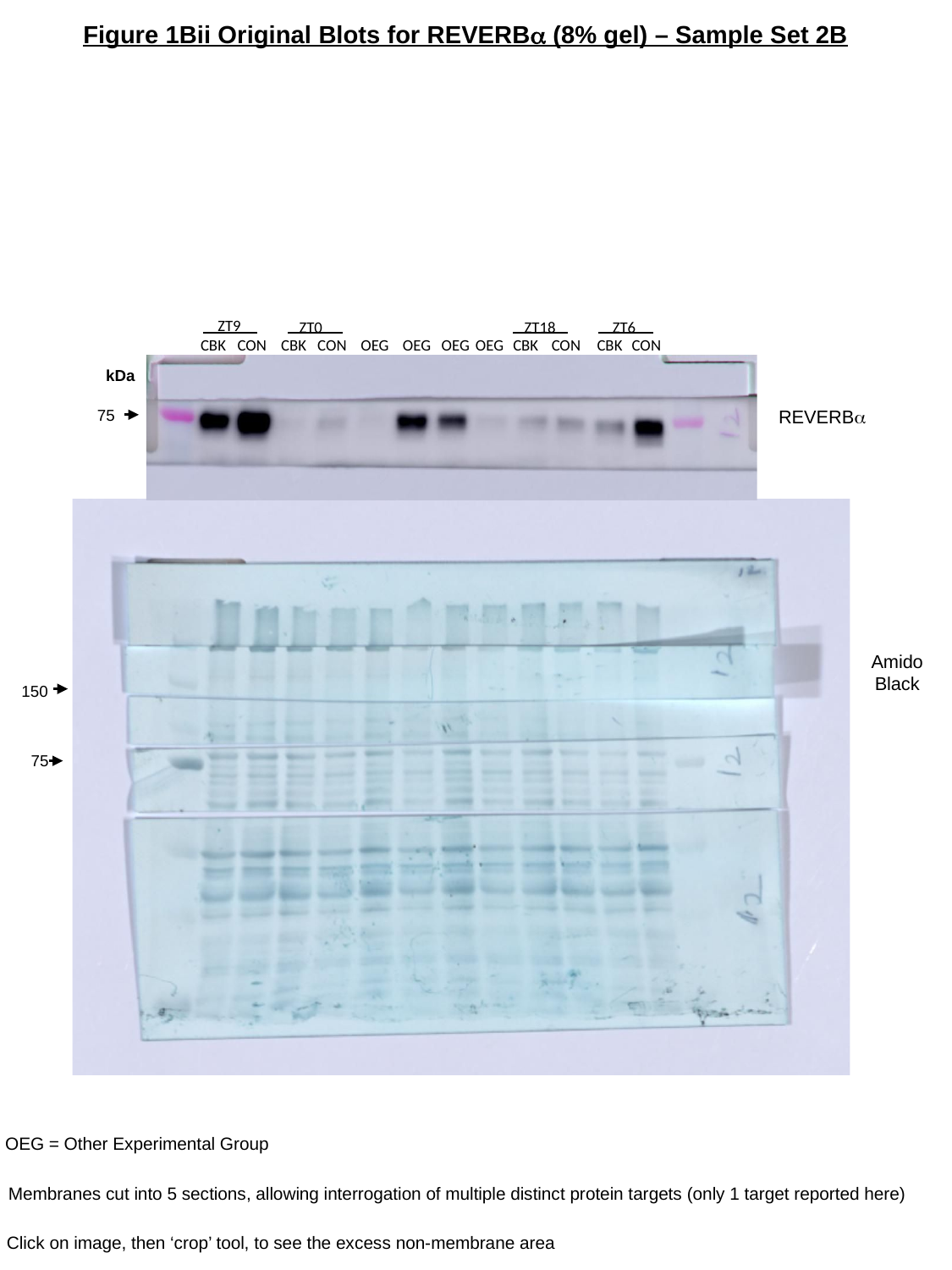

Figure 1Bii Original Blots for REVERBa (8% gel) – Sample Set 2B
ZT9
ZT0
ZT18
ZT6
CBK
CON
CBK
CON
OEG
OEG
OEG
OEG
CBK
CON
CBK
CON
kDa
REVERBa
75
Amido
Black
150
75
OEG = Other Experimental Group
Membranes cut into 5 sections, allowing interrogation of multiple distinct protein targets (only 1 target reported here)
Click on image, then ‘crop’ tool, to see the excess non-membrane area

## Slide 15
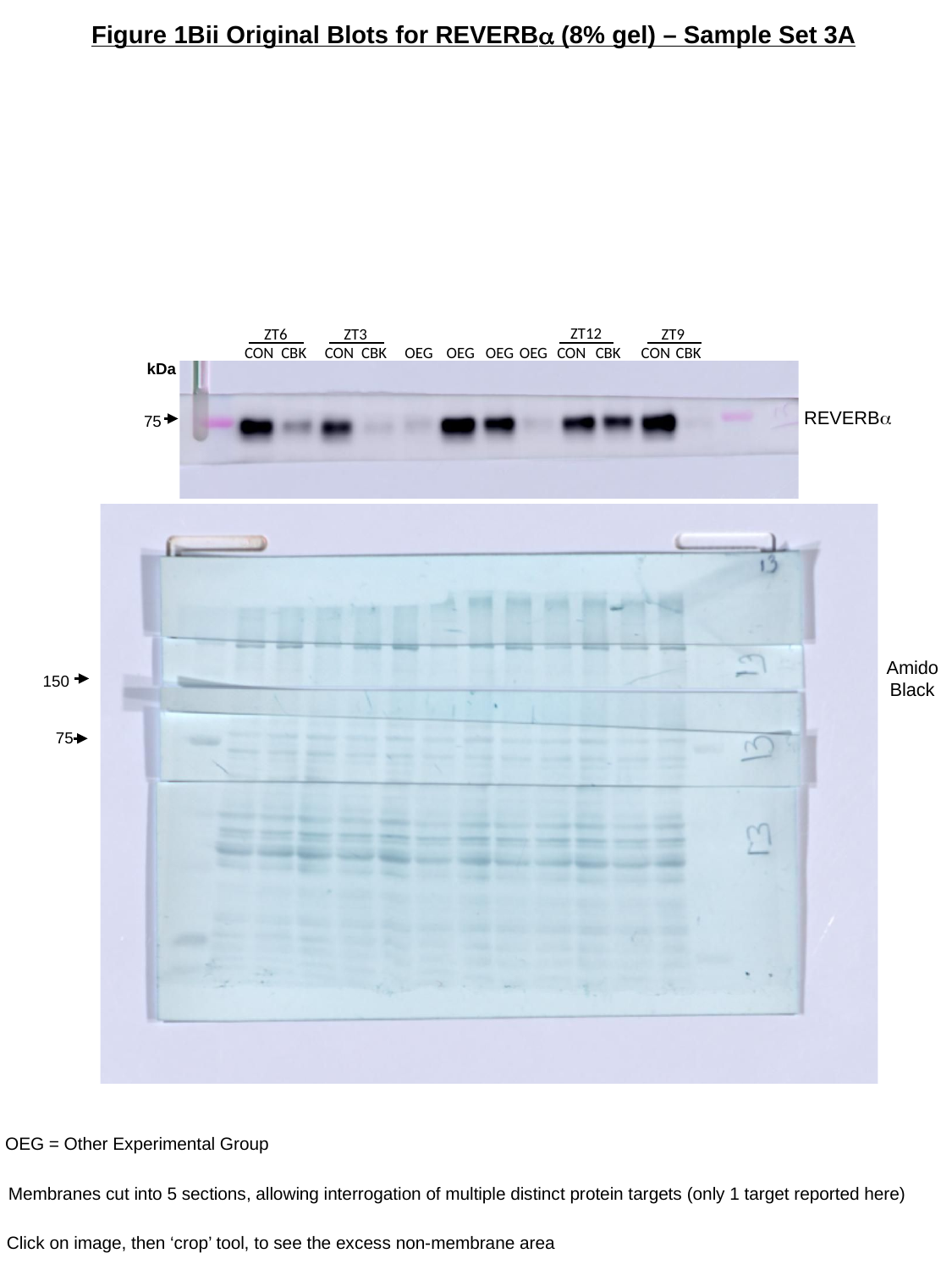

Figure 1Bii Original Blots for REVERBa (8% gel) – Sample Set 3A
ZT12
ZT6
ZT3
ZT9
CON
CBK
CON
CBK
OEG
OEG
OEG
OEG
CON
CBK
CON
CBK
kDa
REVERBa
75
Amido
Black
150
75
OEG = Other Experimental Group
Membranes cut into 5 sections, allowing interrogation of multiple distinct protein targets (only 1 target reported here)
Click on image, then ‘crop’ tool, to see the excess non-membrane area

## Slide 16
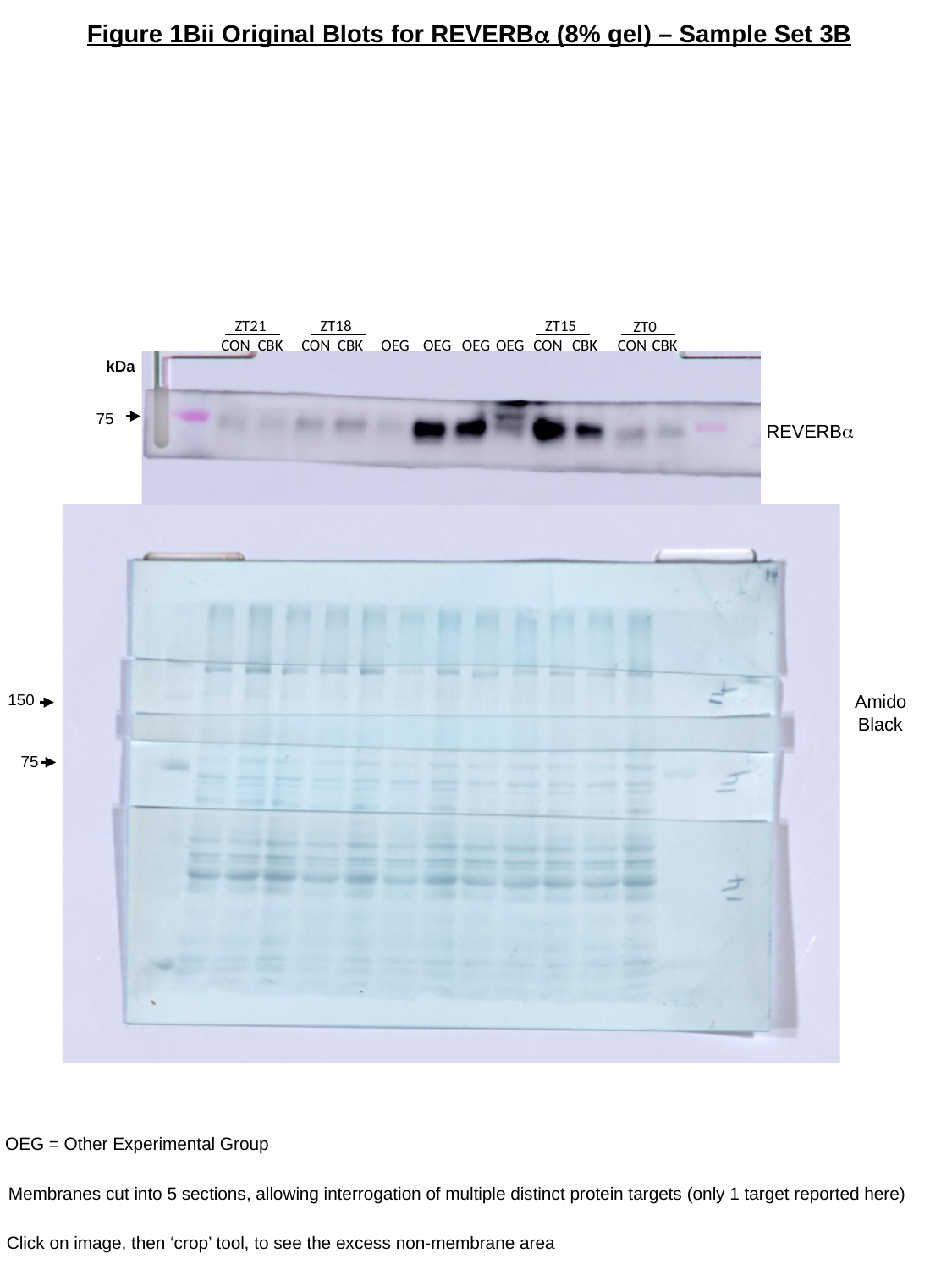

Figure 1Bii Original Blots for REVERBa (8% gel) – Sample Set 3B
ZT15
ZT21
ZT18
ZT0
CON
CBK
CON
CBK
OEG
OEG
OEG
OEG
CON
CBK
CON
CBK
kDa
75
REVERBa
150
Amido
Black
75
OEG = Other Experimental Group
Membranes cut into 5 sections, allowing interrogation of multiple distinct protein targets (only 1 target reported here)
Click on image, then ‘crop’ tool, to see the excess non-membrane area

## Slide 17
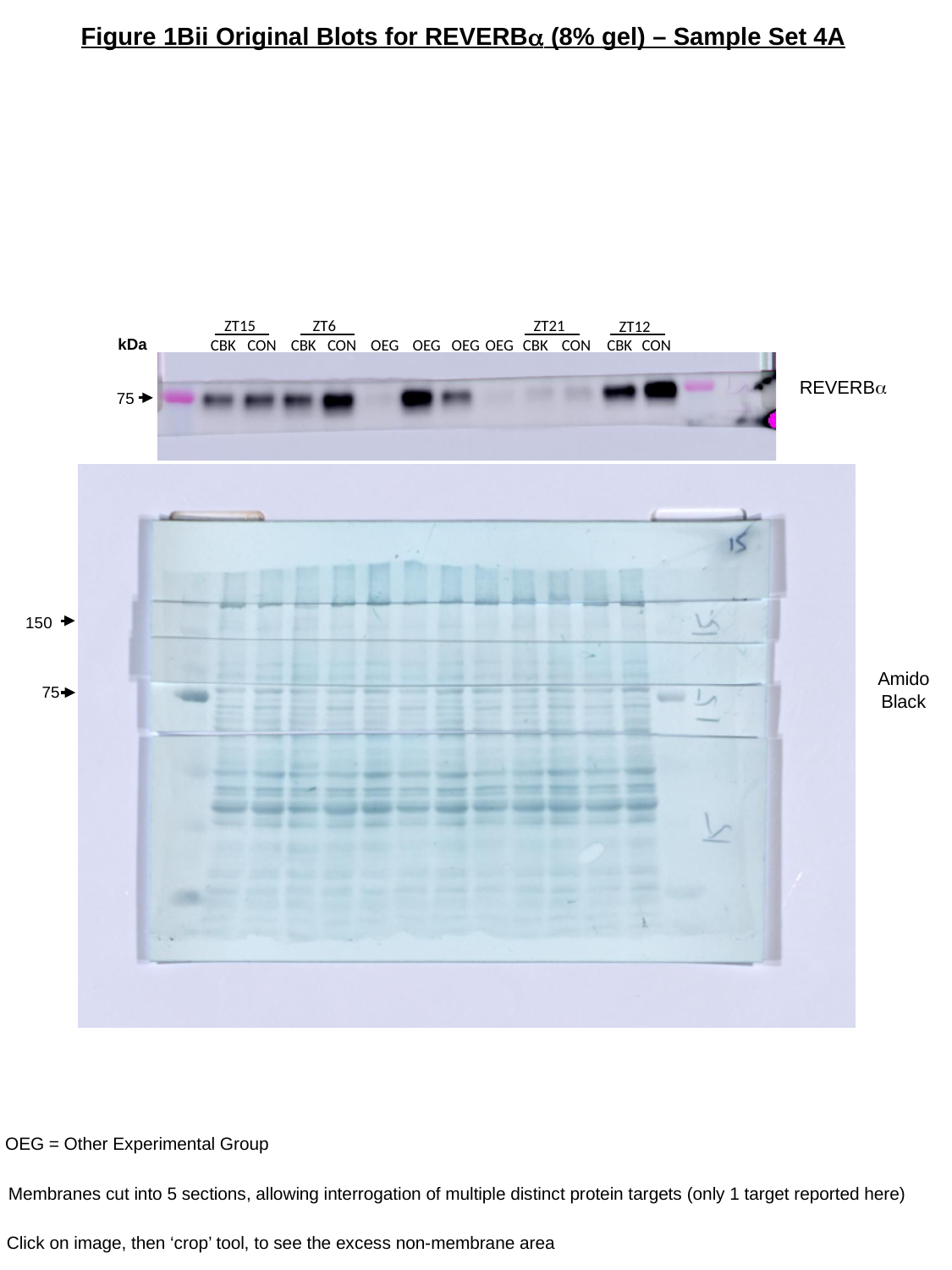

Figure 1Bii Original Blots for REVERBa (8% gel) – Sample Set 4A
ZT21
ZT15
ZT6
ZT12
kDa
CBK
CON
CBK
CON
OEG
OEG
OEG
OEG
CBK
CON
CBK
CON
REVERBa
75
150
Amido
Black
75
OEG = Other Experimental Group
Membranes cut into 5 sections, allowing interrogation of multiple distinct protein targets (only 1 target reported here)
Click on image, then ‘crop’ tool, to see the excess non-membrane area

## Slide 18
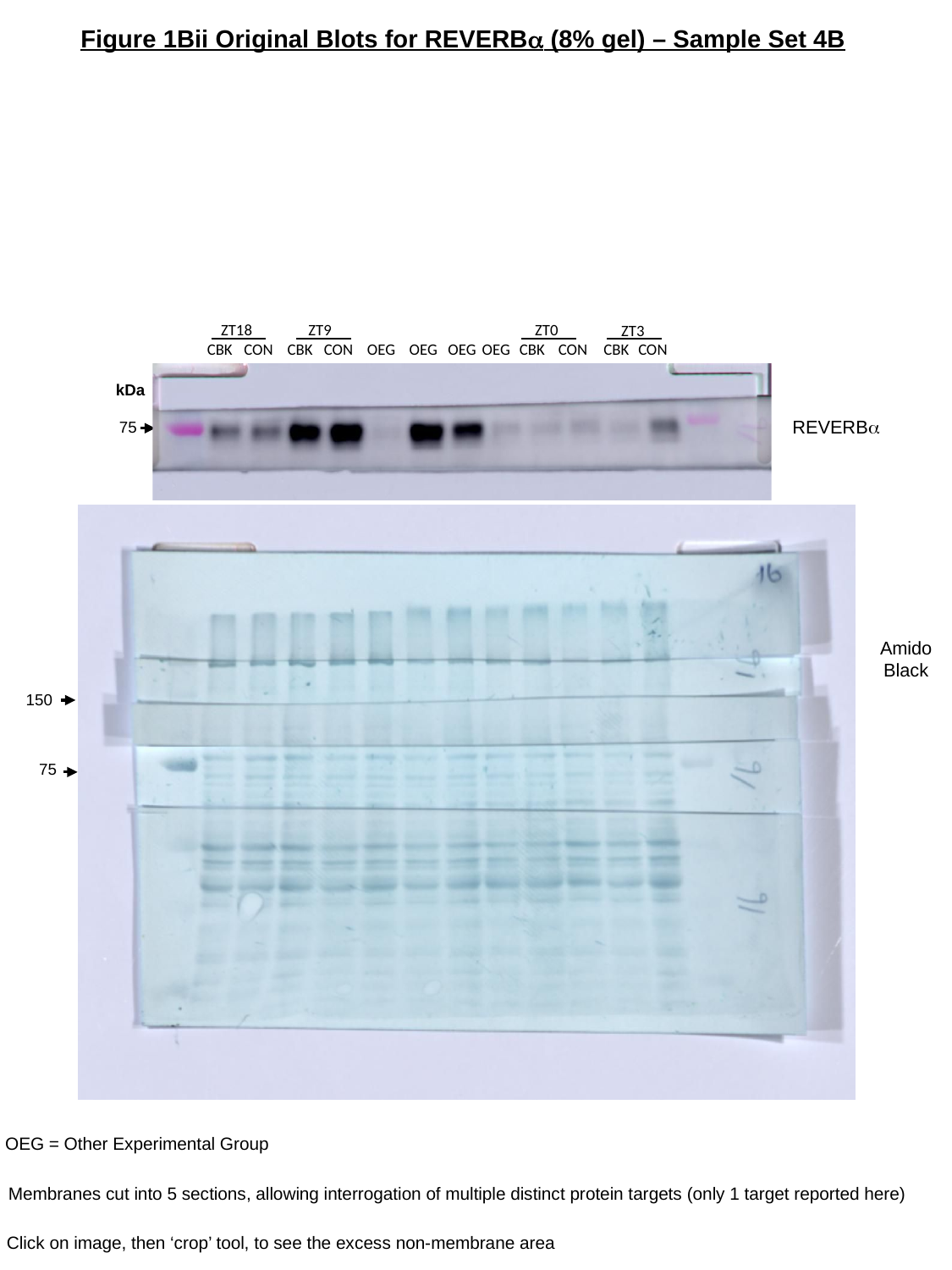

Figure 1Bii Original Blots for REVERBa (8% gel) – Sample Set 4B
ZT0
ZT18
ZT9
ZT3
CBK
CON
CBK
CON
OEG
OEG
OEG
OEG
CBK
CON
CBK
CON
kDa
REVERBa
75
Amido
Black
150
75
OEG = Other Experimental Group
Membranes cut into 5 sections, allowing interrogation of multiple distinct protein targets (only 1 target reported here)
Click on image, then ‘crop’ tool, to see the excess non-membrane area

## Slide 19
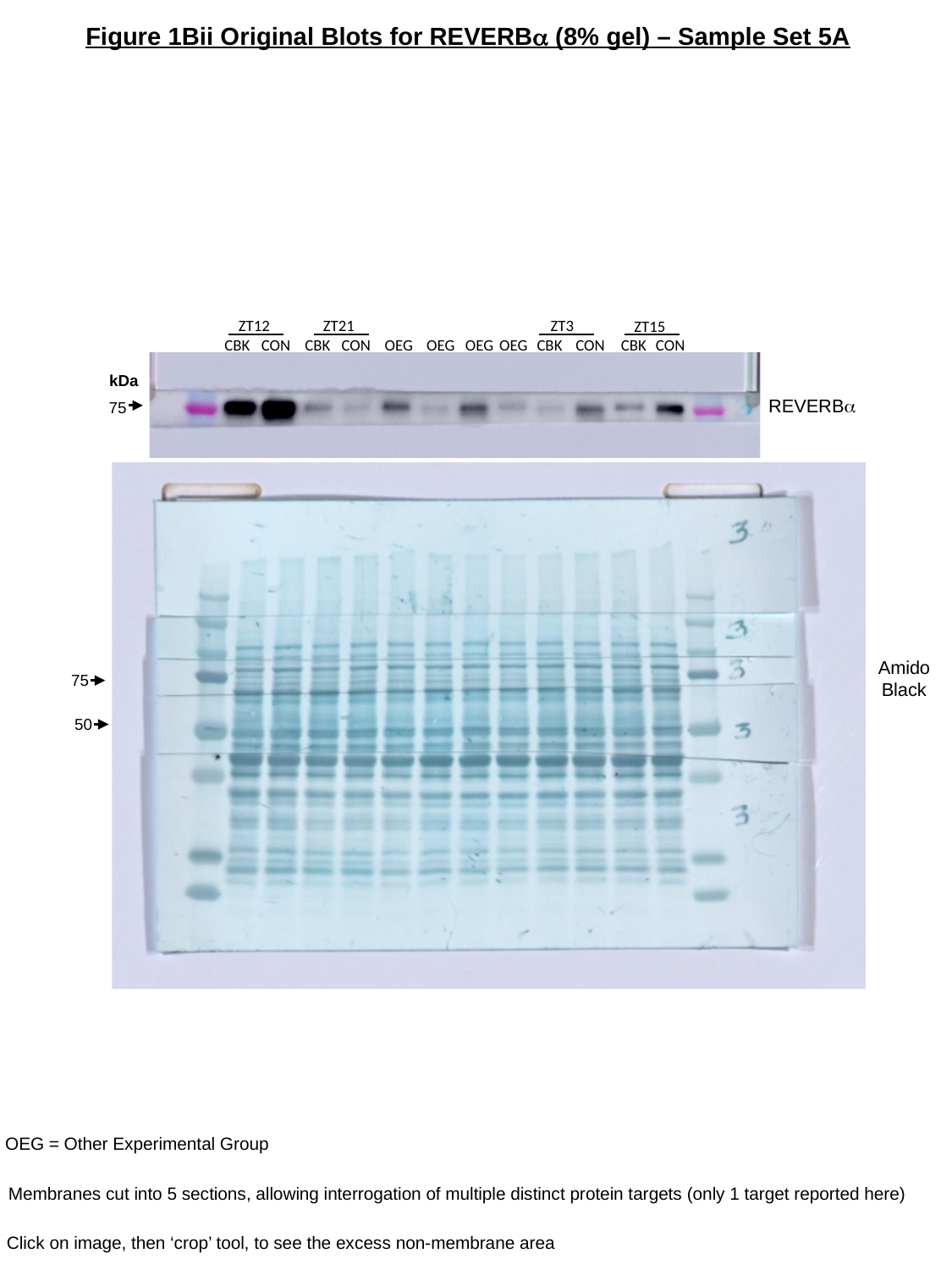

Figure 1Bii Original Blots for REVERBa (8% gel) – Sample Set 5A
ZT3
ZT12
ZT21
ZT15
CBK
CON
CBK
CON
OEG
OEG
OEG
OEG
CBK
CON
CBK
CON
kDa
REVERBa
75
Amido
Black
75
50
OEG = Other Experimental Group
Membranes cut into 5 sections, allowing interrogation of multiple distinct protein targets (only 1 target reported here)
Click on image, then ‘crop’ tool, to see the excess non-membrane area

## Slide 20
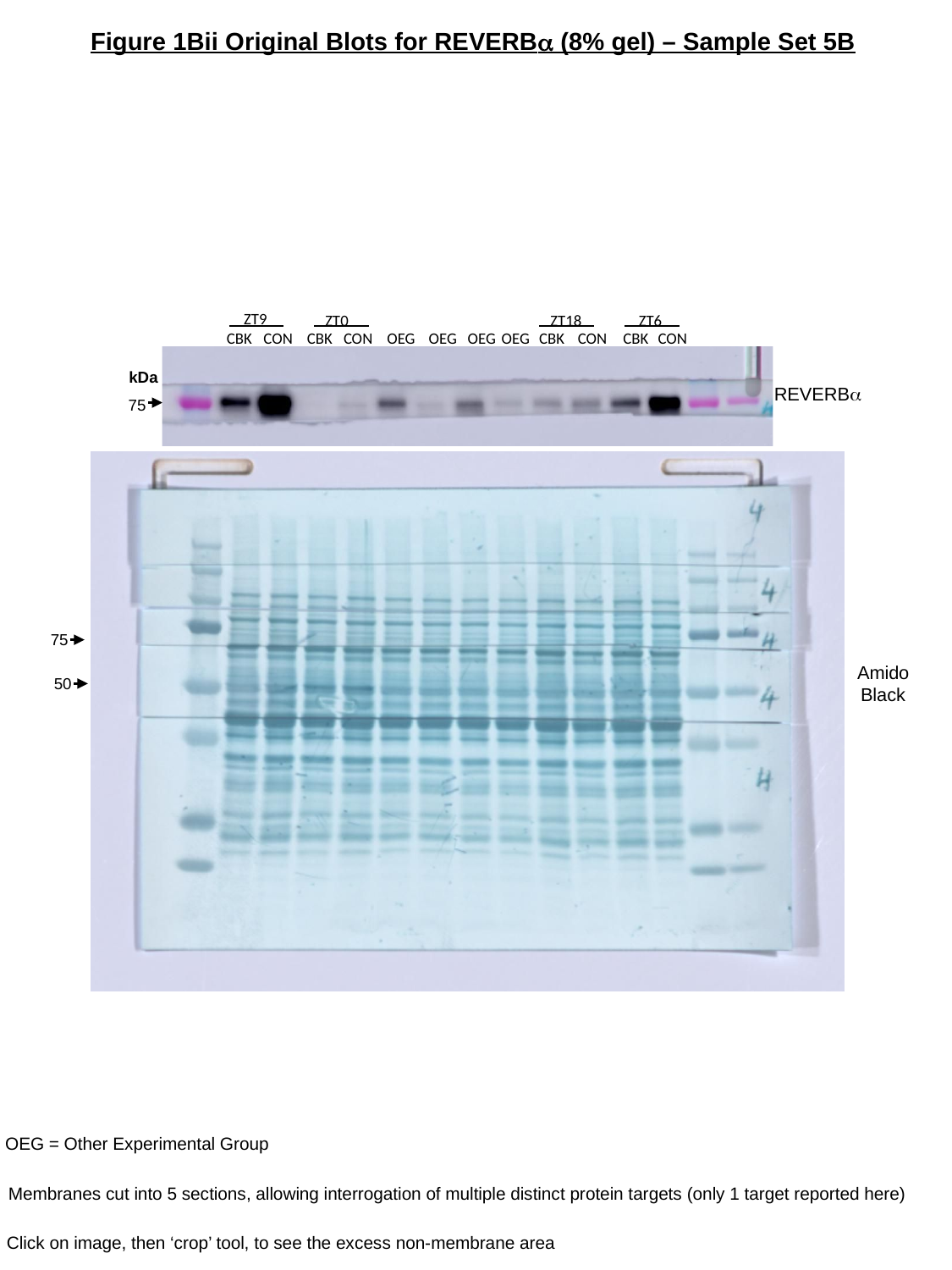

Figure 1Bii Original Blots for REVERBa (8% gel) – Sample Set 5B
ZT9
ZT0
ZT18
ZT6
CBK
CON
CBK
CON
OEG
OEG
OEG
OEG
CBK
CON
CBK
CON
kDa
REVERBa
75
75
Amido
Black
50
OEG = Other Experimental Group
Membranes cut into 5 sections, allowing interrogation of multiple distinct protein targets (only 1 target reported here)
Click on image, then ‘crop’ tool, to see the excess non-membrane area

## Slide 21
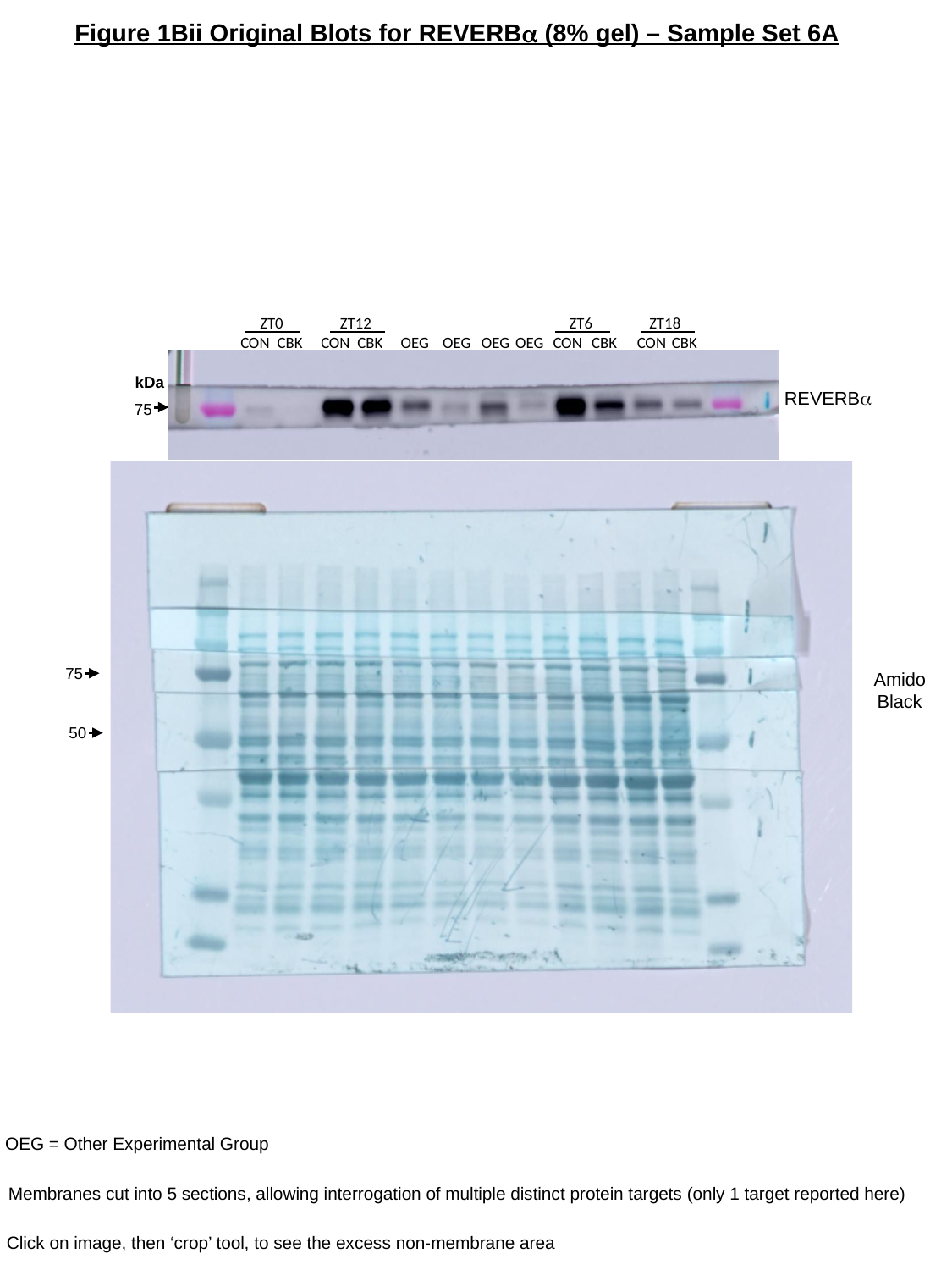

Figure 1Bii Original Blots for REVERBa (8% gel) – Sample Set 6A
ZT6
ZT0
ZT12
ZT18
CON
CBK
CON
CBK
OEG
OEG
OEG
OEG
CON
CBK
CON
CBK
kDa
REVERBa
75
75
Amido
Black
50
OEG = Other Experimental Group
Membranes cut into 5 sections, allowing interrogation of multiple distinct protein targets (only 1 target reported here)
Click on image, then ‘crop’ tool, to see the excess non-membrane area

## Slide 22
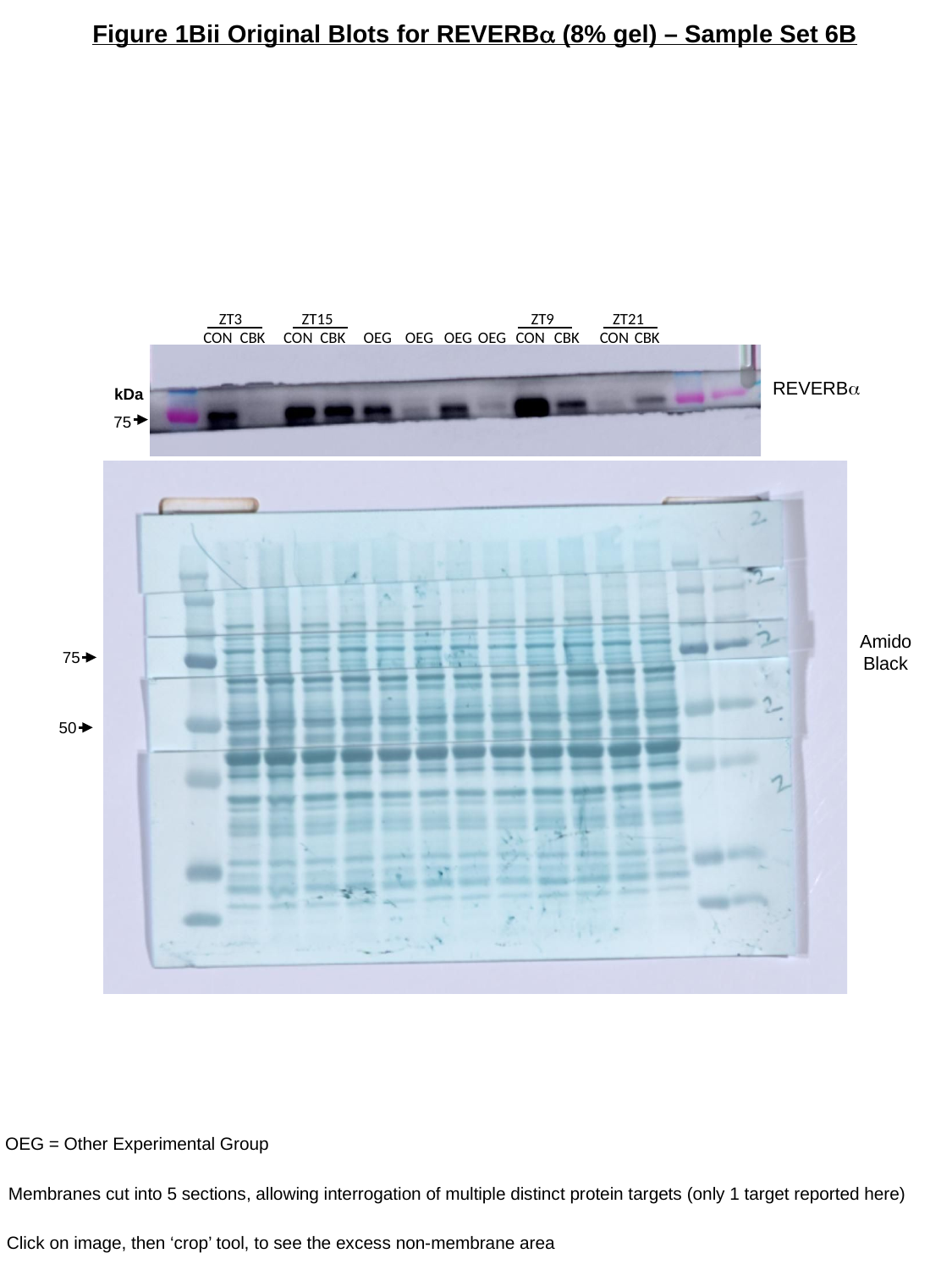

Figure 1Bii Original Blots for REVERBa (8% gel) – Sample Set 6B
ZT9
ZT3
ZT15
ZT21
CON
CBK
CON
CBK
OEG
OEG
OEG
OEG
CON
CBK
CON
CBK
REVERBa
kDa
75
Amido
Black
75
50
OEG = Other Experimental Group
Membranes cut into 5 sections, allowing interrogation of multiple distinct protein targets (only 1 target reported here)
Click on image, then ‘crop’ tool, to see the excess non-membrane area

## Slide 23
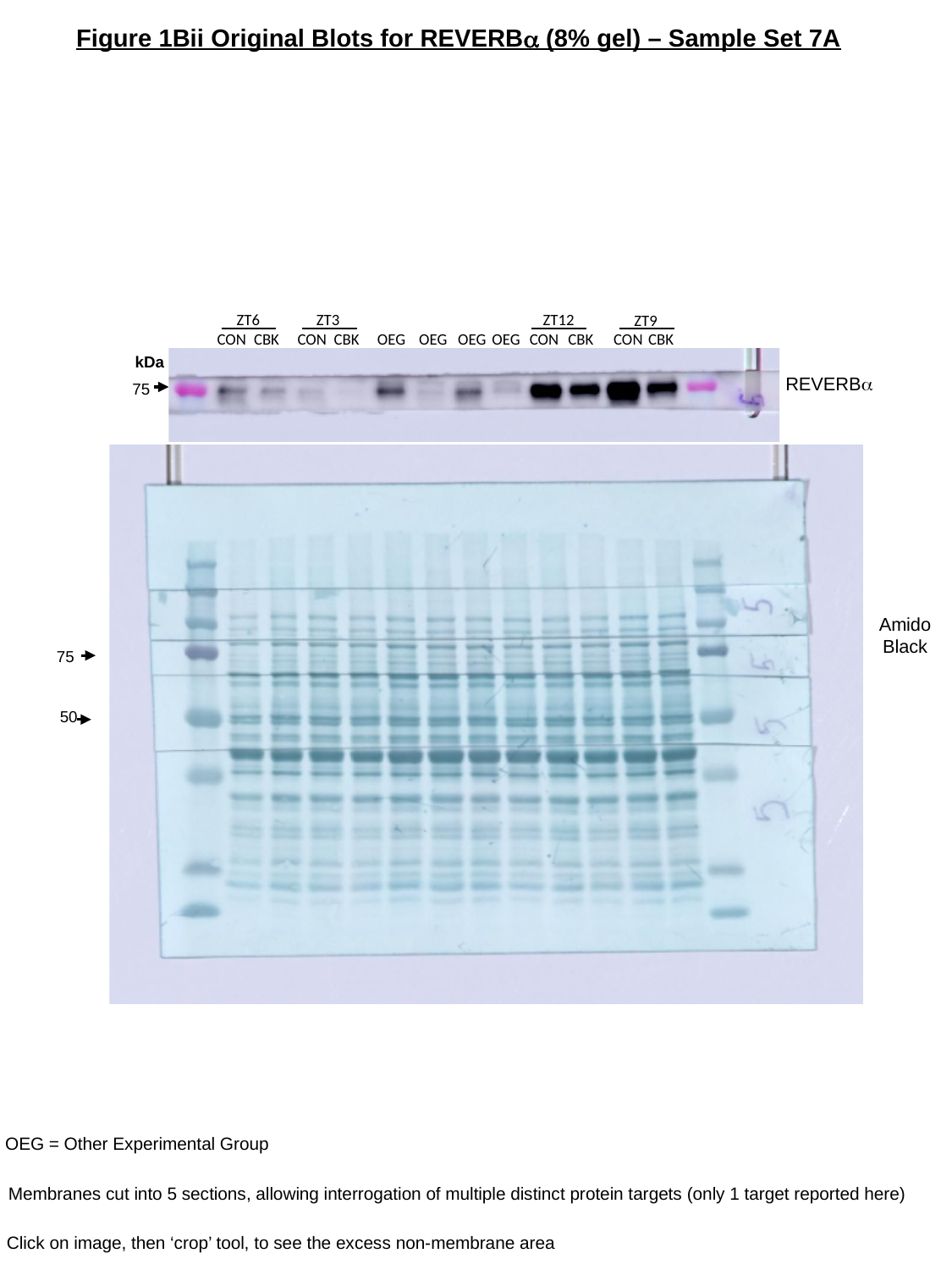

Figure 1Bii Original Blots for REVERBa (8% gel) – Sample Set 7A
ZT12
ZT6
ZT3
ZT9
CON
CBK
CON
CBK
OEG
OEG
OEG
OEG
CON
CBK
CON
CBK
kDa
REVERBa
75
Amido
Black
75
50
OEG = Other Experimental Group
Membranes cut into 5 sections, allowing interrogation of multiple distinct protein targets (only 1 target reported here)
Click on image, then ‘crop’ tool, to see the excess non-membrane area

## Slide 24
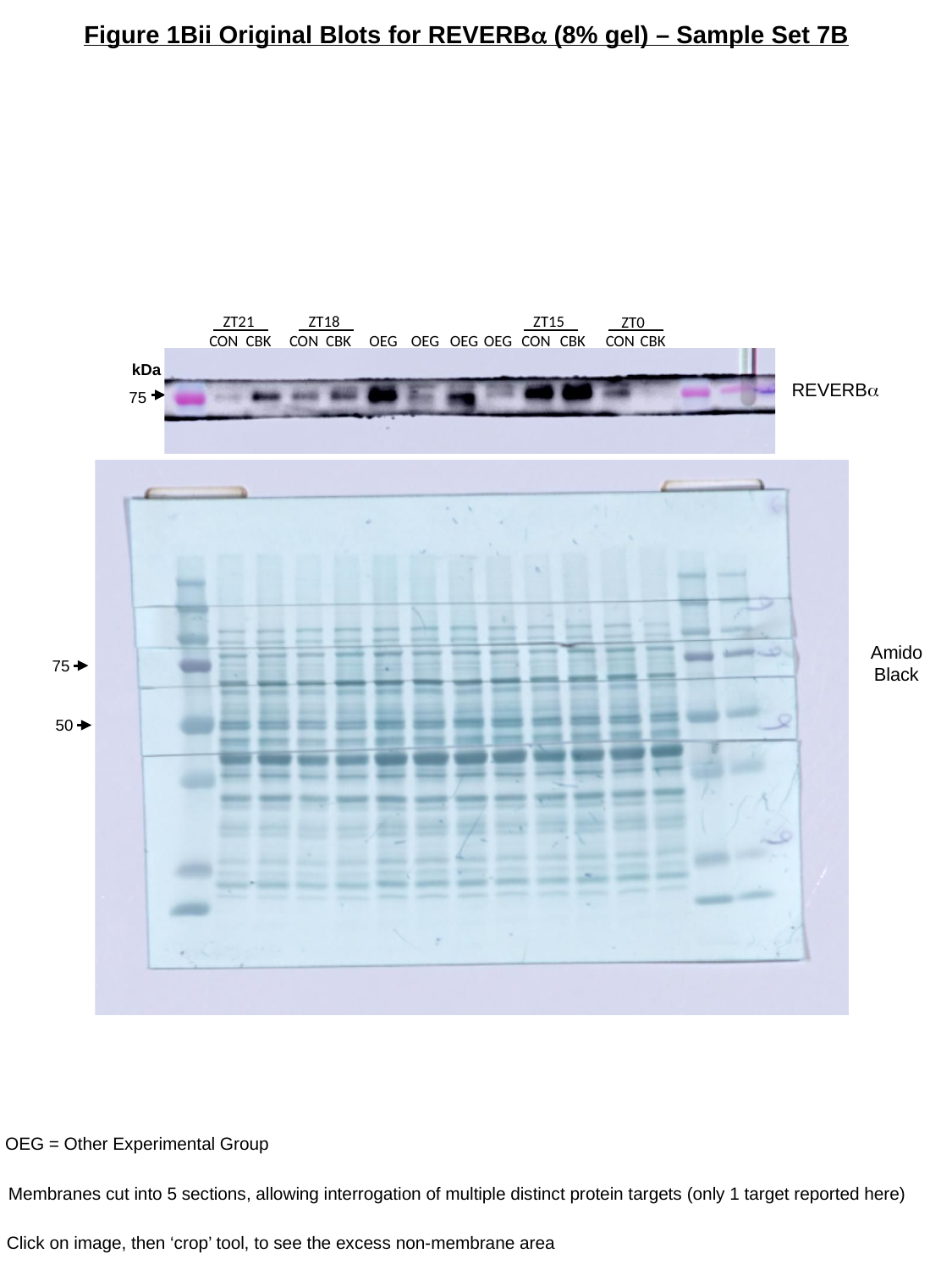

Figure 1Bii Original Blots for REVERBa (8% gel) – Sample Set 7B
ZT15
ZT21
ZT18
ZT0
CON
CBK
CON
CBK
OEG
OEG
OEG
OEG
CON
CBK
CON
CBK
kDa
REVERBa
75
Amido
Black
75
50
OEG = Other Experimental Group
Membranes cut into 5 sections, allowing interrogation of multiple distinct protein targets (only 1 target reported here)
Click on image, then ‘crop’ tool, to see the excess non-membrane area

## Slide 25
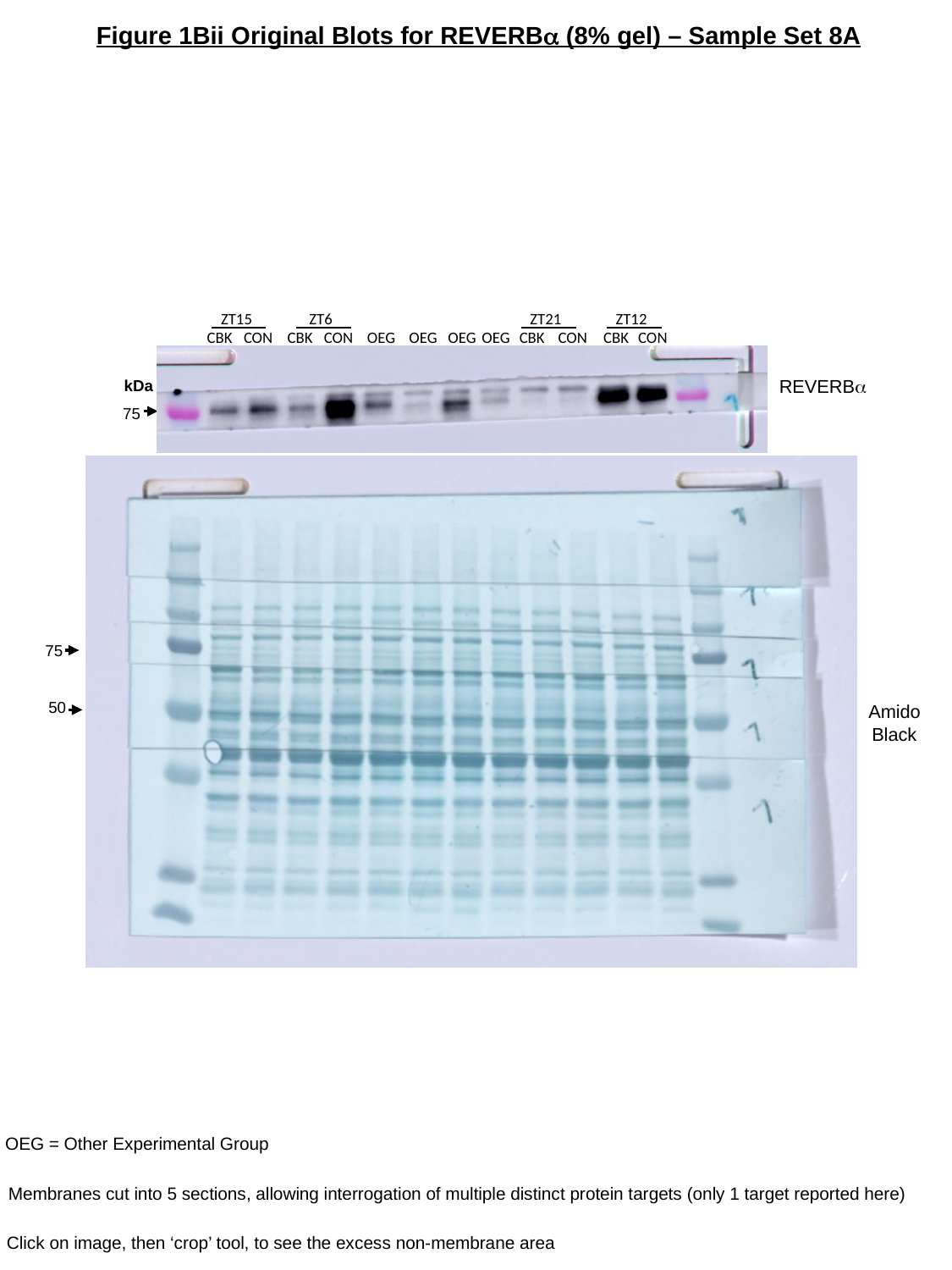

Figure 1Bii Original Blots for REVERBa (8% gel) – Sample Set 8A
ZT21
ZT15
ZT6
ZT12
CBK
CON
CBK
CON
OEG
OEG
OEG
OEG
CBK
CON
CBK
CON
kDa
REVERBa
75
75
50
Amido
Black
OEG = Other Experimental Group
Membranes cut into 5 sections, allowing interrogation of multiple distinct protein targets (only 1 target reported here)
Click on image, then ‘crop’ tool, to see the excess non-membrane area

## Slide 26
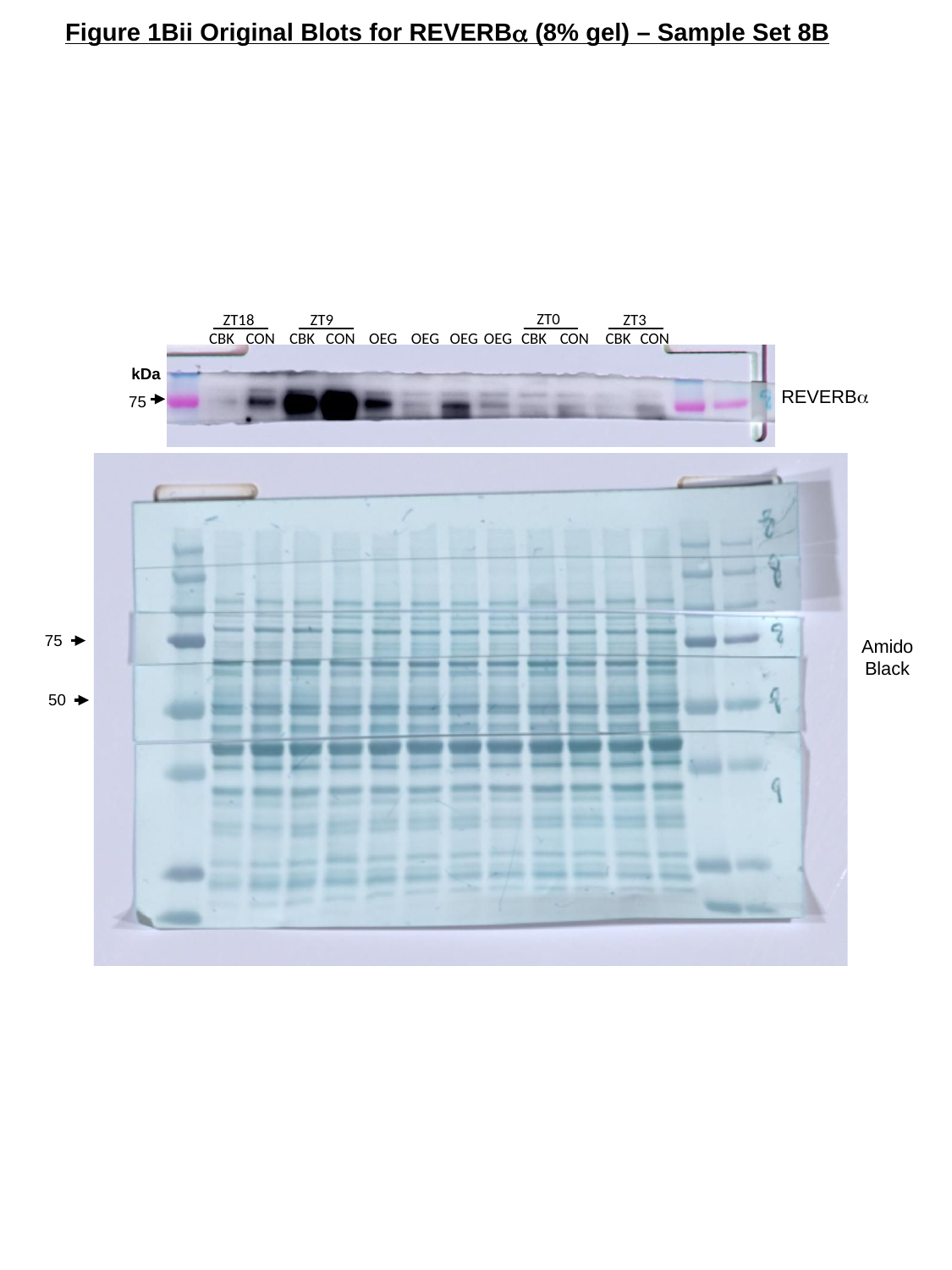

Figure 1Bii Original Blots for REVERBa (8% gel) – Sample Set 8B
ZT0
ZT18
ZT9
ZT3
CBK
CON
CBK
CON
OEG
OEG
OEG
OEG
CBK
CON
CBK
CON
kDa
REVERBa
75
75
Amido
Black
50

## Slide 27
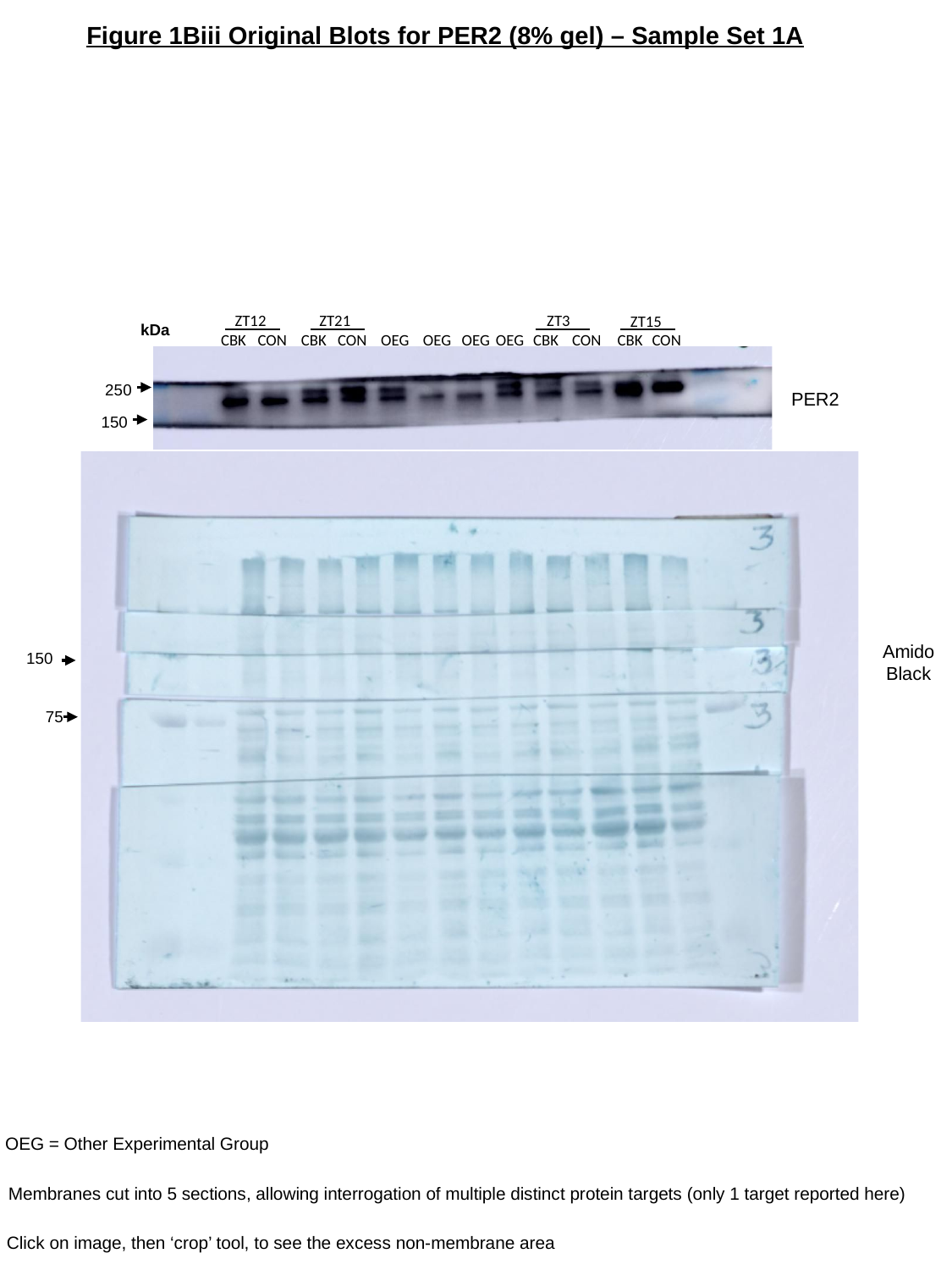

Figure 1Biii Original Blots for PER2 (8% gel) – Sample Set 1A
ZT3
ZT12
ZT21
ZT15
kDa
CBK
CON
CBK
CON
OEG
OEG
OEG
OEG
CBK
CON
CBK
CON
250
PER2
150
Amido
Black
150
75
OEG = Other Experimental Group
Membranes cut into 5 sections, allowing interrogation of multiple distinct protein targets (only 1 target reported here)
Click on image, then ‘crop’ tool, to see the excess non-membrane area

## Slide 28
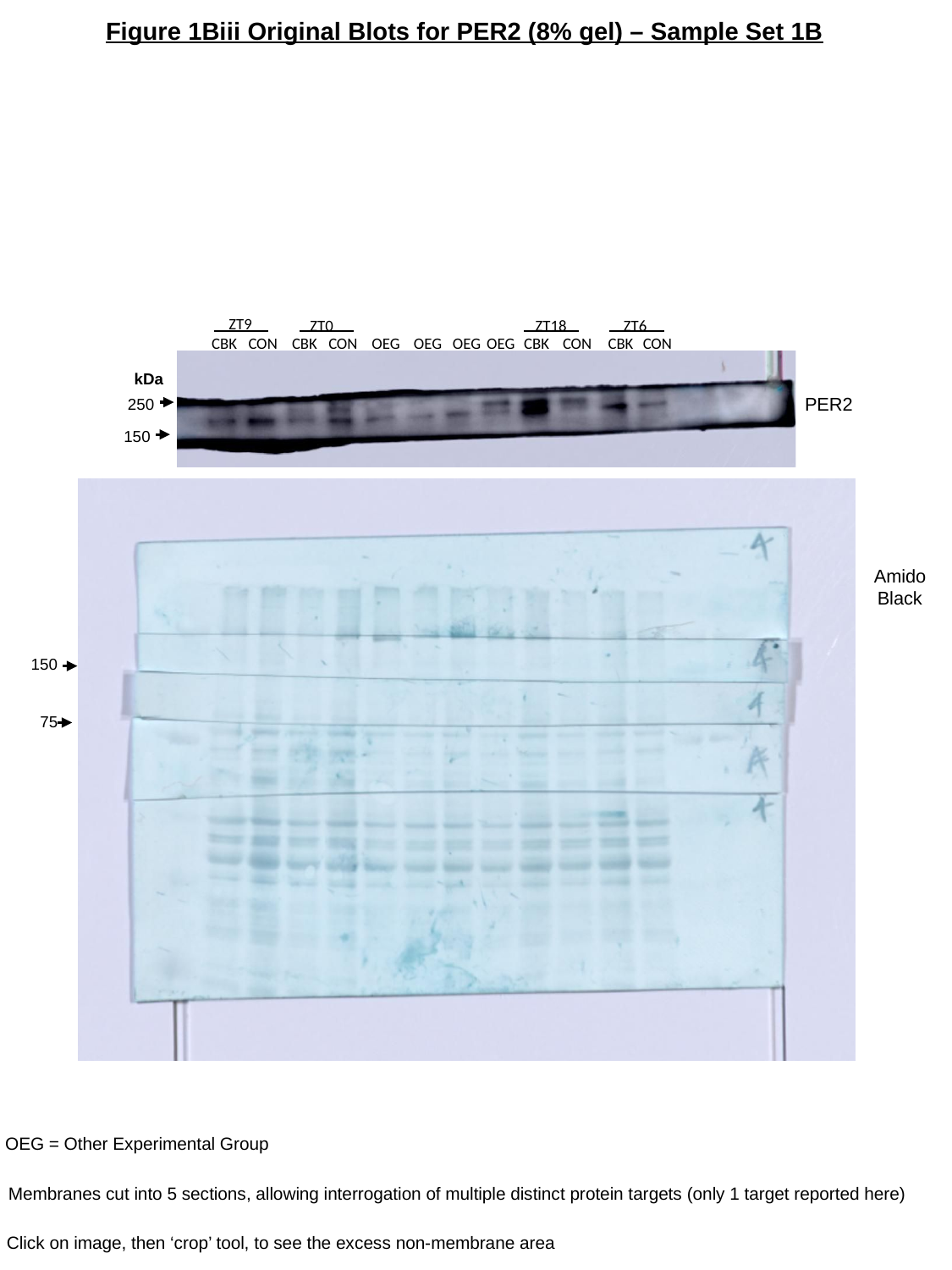

Figure 1Biii Original Blots for PER2 (8% gel) – Sample Set 1B
ZT9
ZT0
ZT18
ZT6
CBK
CON
CBK
CON
OEG
OEG
OEG
OEG
CBK
CON
CBK
CON
kDa
PER2
250
150
Amido
Black
150
75
OEG = Other Experimental Group
Membranes cut into 5 sections, allowing interrogation of multiple distinct protein targets (only 1 target reported here)
Click on image, then ‘crop’ tool, to see the excess non-membrane area

## Slide 29
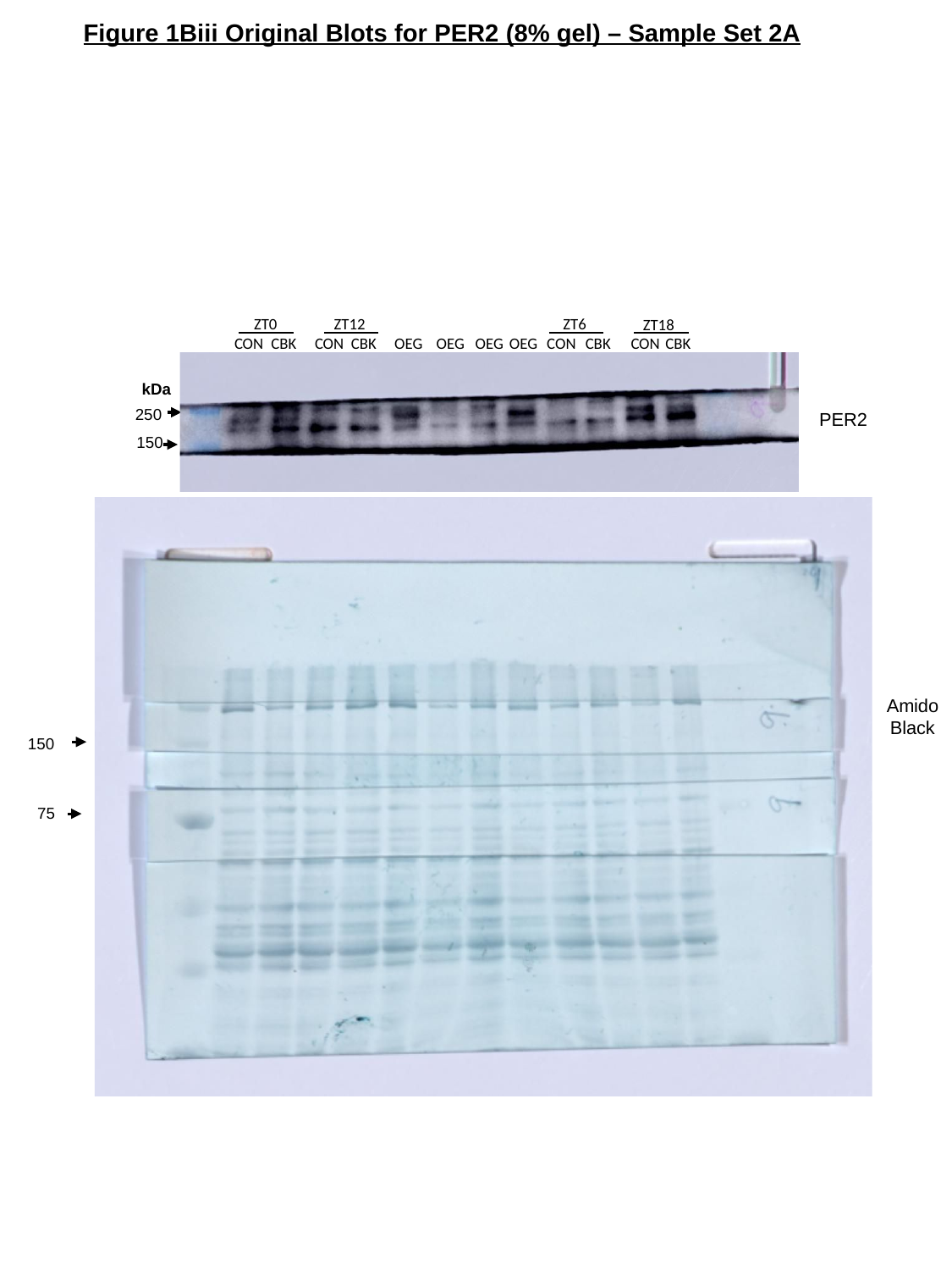

Figure 1Biii Original Blots for PER2 (8% gel) – Sample Set 2A
ZT6
ZT0
ZT12
ZT18
CON
CBK
CON
CBK
OEG
OEG
OEG
OEG
CON
CBK
CON
CBK
kDa
250
PER2
150
Amido
Black
150
75

## Slide 30
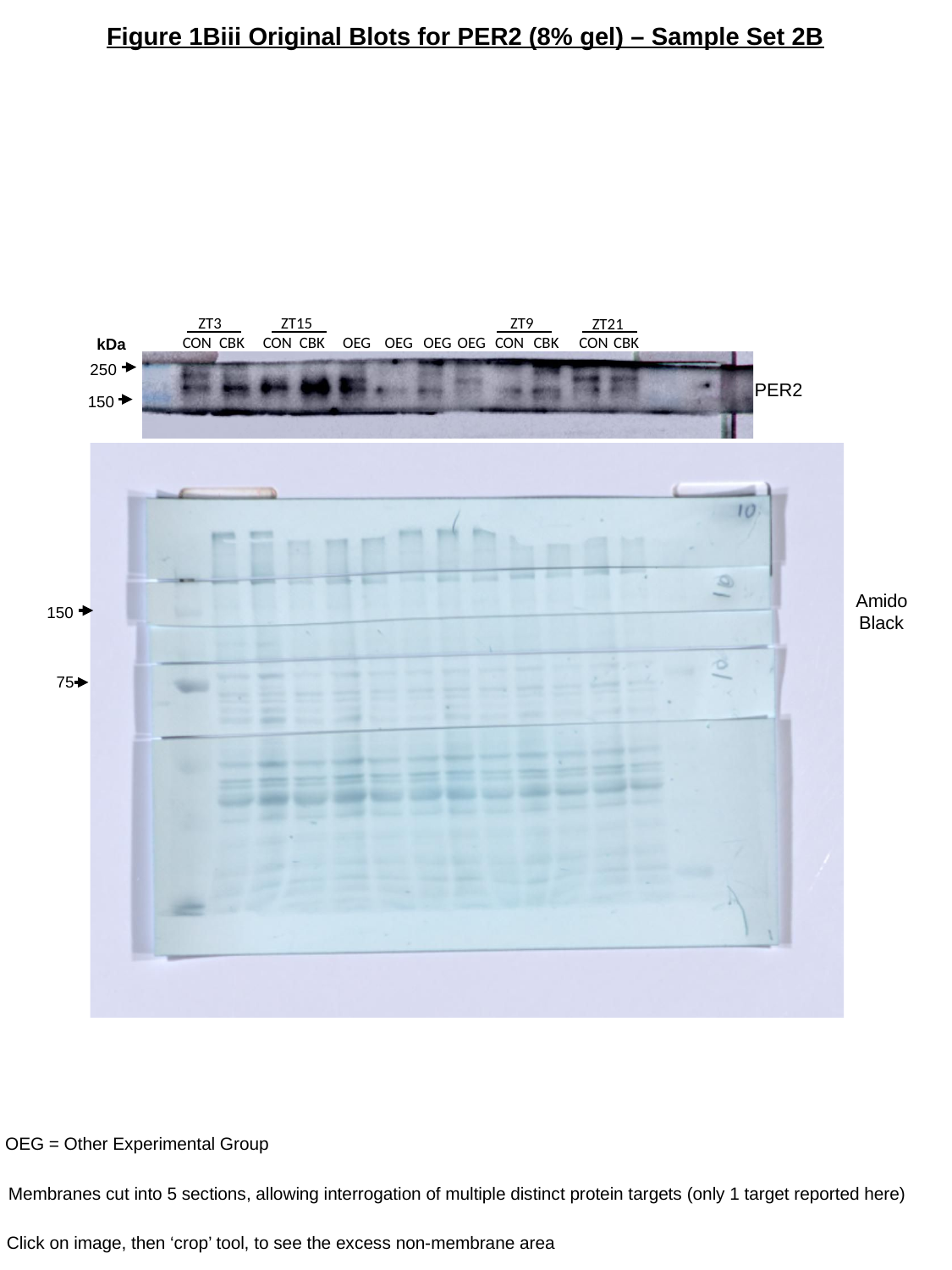

Figure 1Biii Original Blots for PER2 (8% gel) – Sample Set 2B
ZT9
ZT3
ZT15
ZT21
kDa
CON
CBK
CON
CBK
OEG
OEG
OEG
OEG
CON
CBK
CON
CBK
250
PER2
150
Amido
Black
150
75
OEG = Other Experimental Group
Membranes cut into 5 sections, allowing interrogation of multiple distinct protein targets (only 1 target reported here)
Click on image, then ‘crop’ tool, to see the excess non-membrane area

## Slide 31
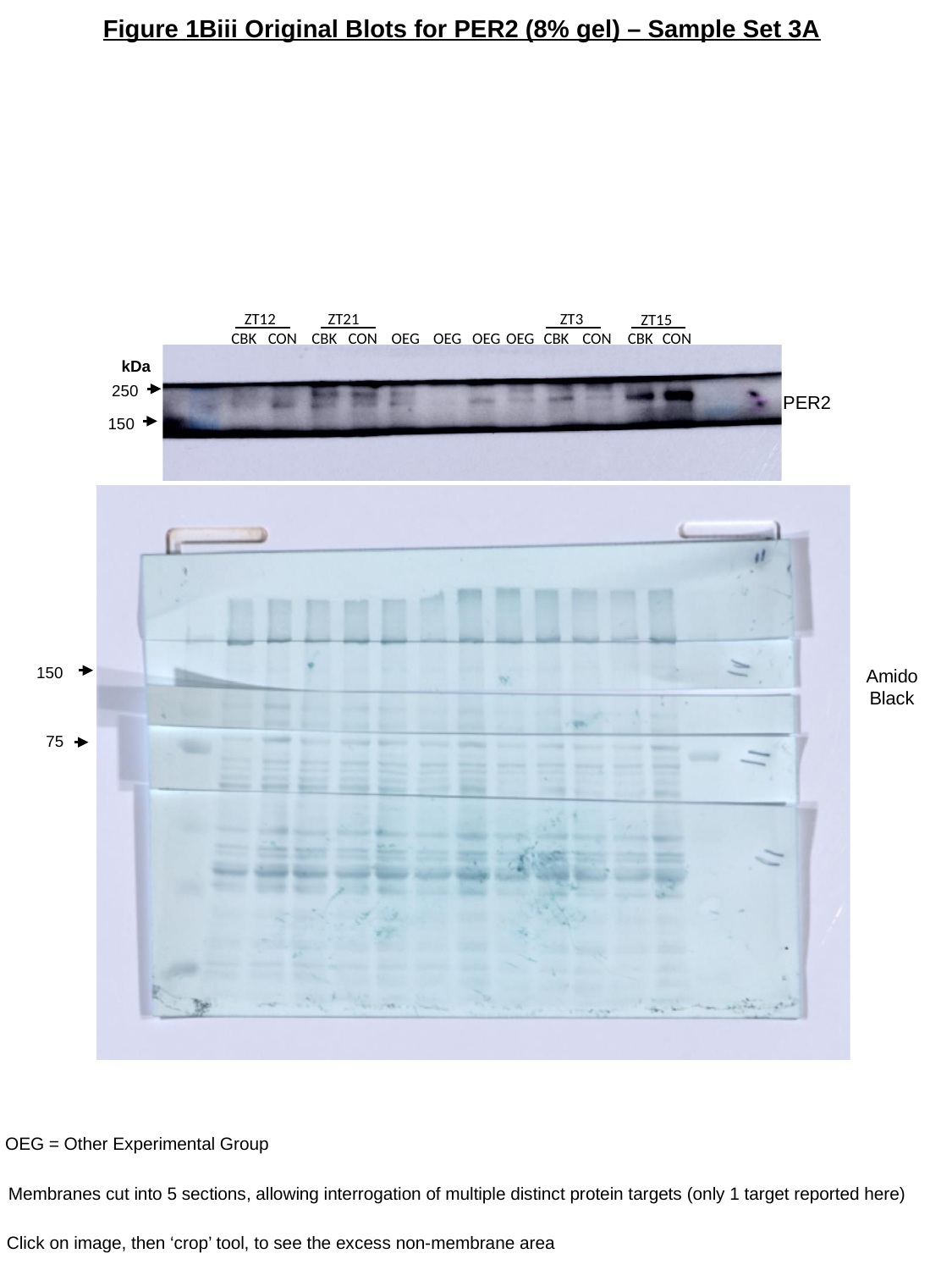

Figure 1Biii Original Blots for PER2 (8% gel) – Sample Set 3A
ZT3
ZT12
ZT21
ZT15
CBK
CON
CBK
CON
OEG
OEG
OEG
OEG
CBK
CON
CBK
CON
kDa
250
PER2
150
150
Amido
Black
75
OEG = Other Experimental Group
Membranes cut into 5 sections, allowing interrogation of multiple distinct protein targets (only 1 target reported here)
Click on image, then ‘crop’ tool, to see the excess non-membrane area

## Slide 32
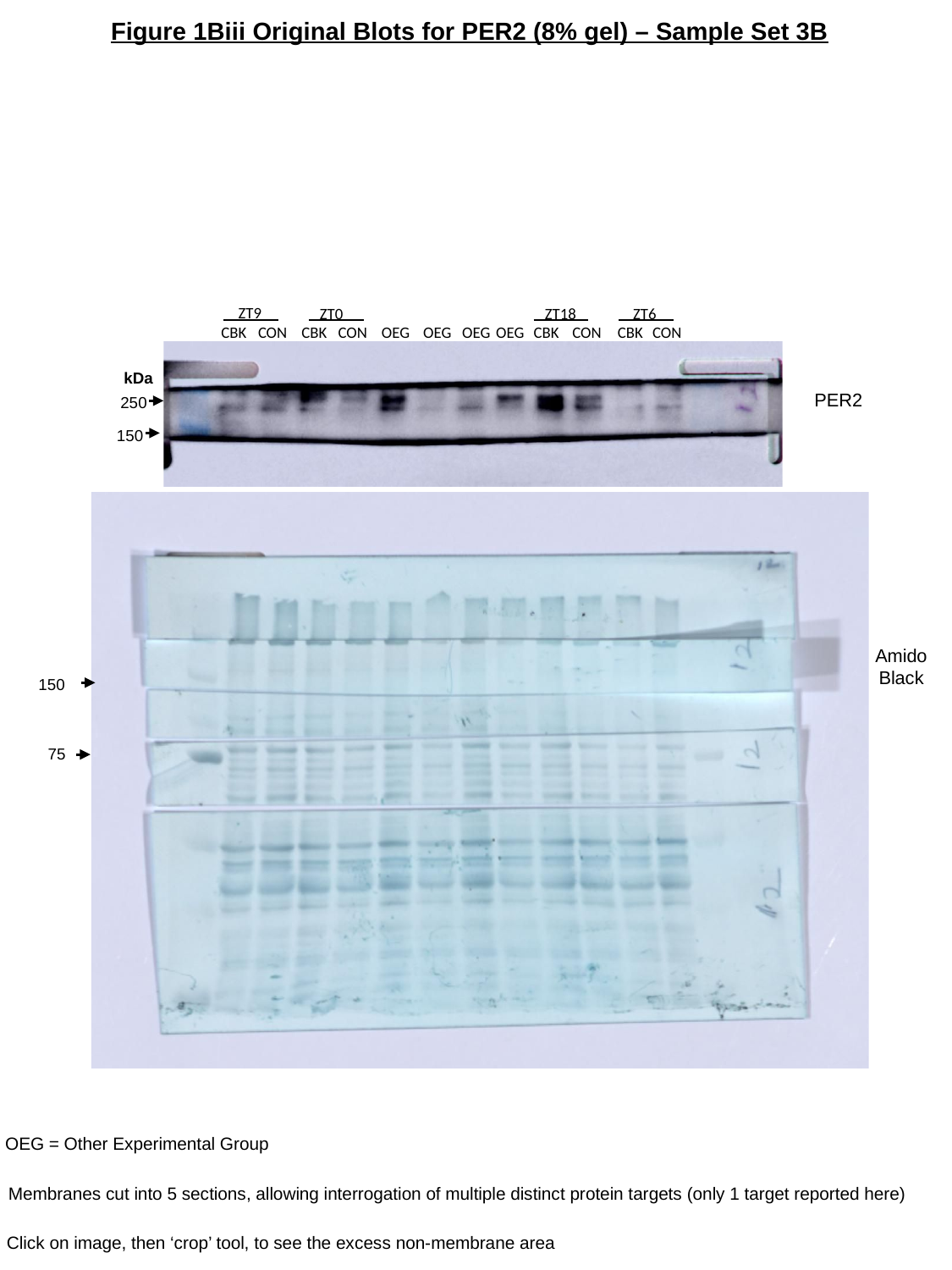

Figure 1Biii Original Blots for PER2 (8% gel) – Sample Set 3B
ZT9
ZT0
ZT18
ZT6
CBK
CON
CBK
CON
OEG
OEG
OEG
OEG
CBK
CON
CBK
CON
kDa
PER2
250
150
Amido
Black
150
75
OEG = Other Experimental Group
Membranes cut into 5 sections, allowing interrogation of multiple distinct protein targets (only 1 target reported here)
Click on image, then ‘crop’ tool, to see the excess non-membrane area

## Slide 33
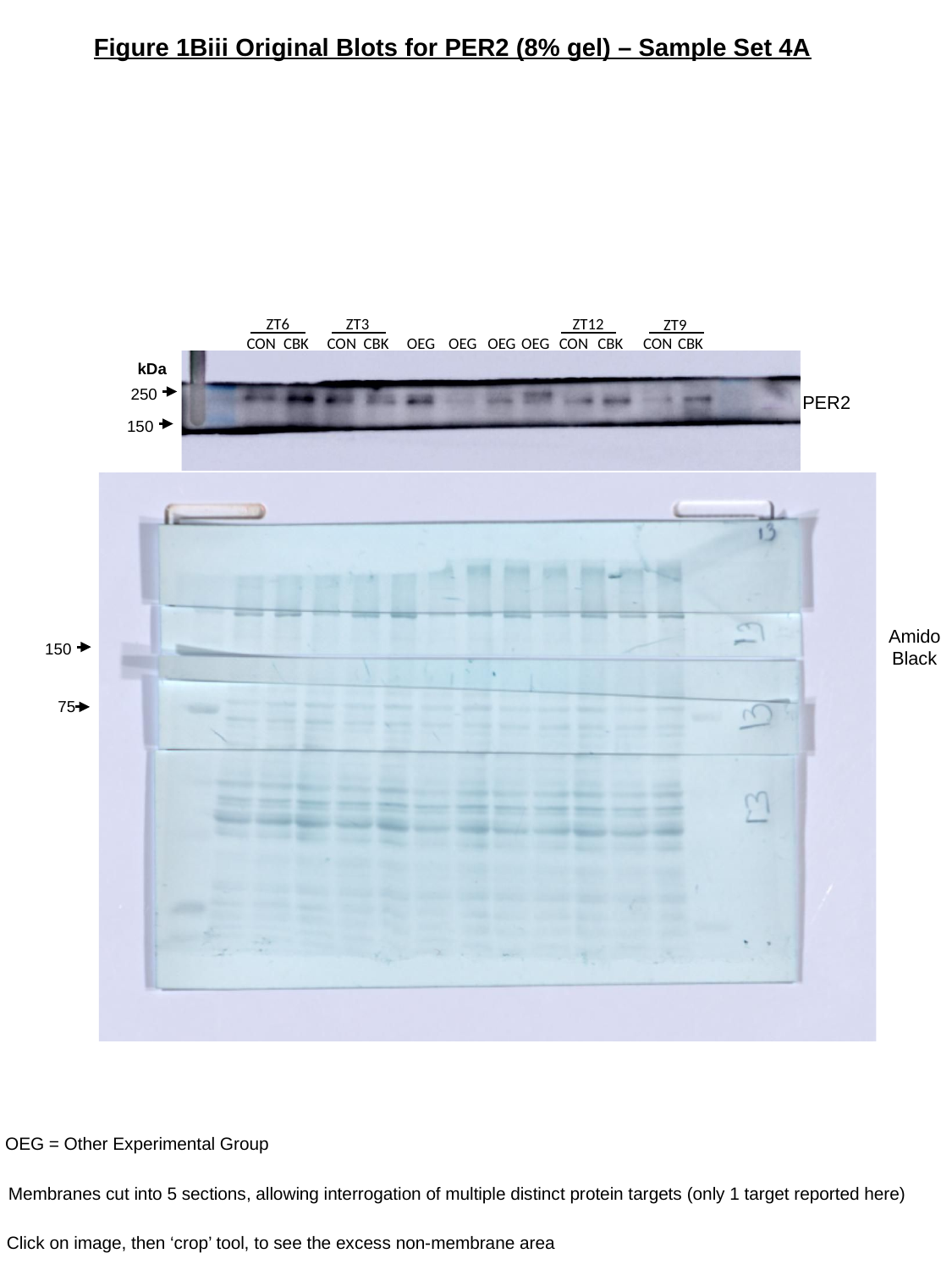

Figure 1Biii Original Blots for PER2 (8% gel) – Sample Set 4A
ZT12
ZT6
ZT3
ZT9
CON
CBK
CON
CBK
OEG
OEG
OEG
OEG
CON
CBK
CON
CBK
kDa
250
PER2
150
Amido
Black
150
75
OEG = Other Experimental Group
Membranes cut into 5 sections, allowing interrogation of multiple distinct protein targets (only 1 target reported here)
Click on image, then ‘crop’ tool, to see the excess non-membrane area

## Slide 34
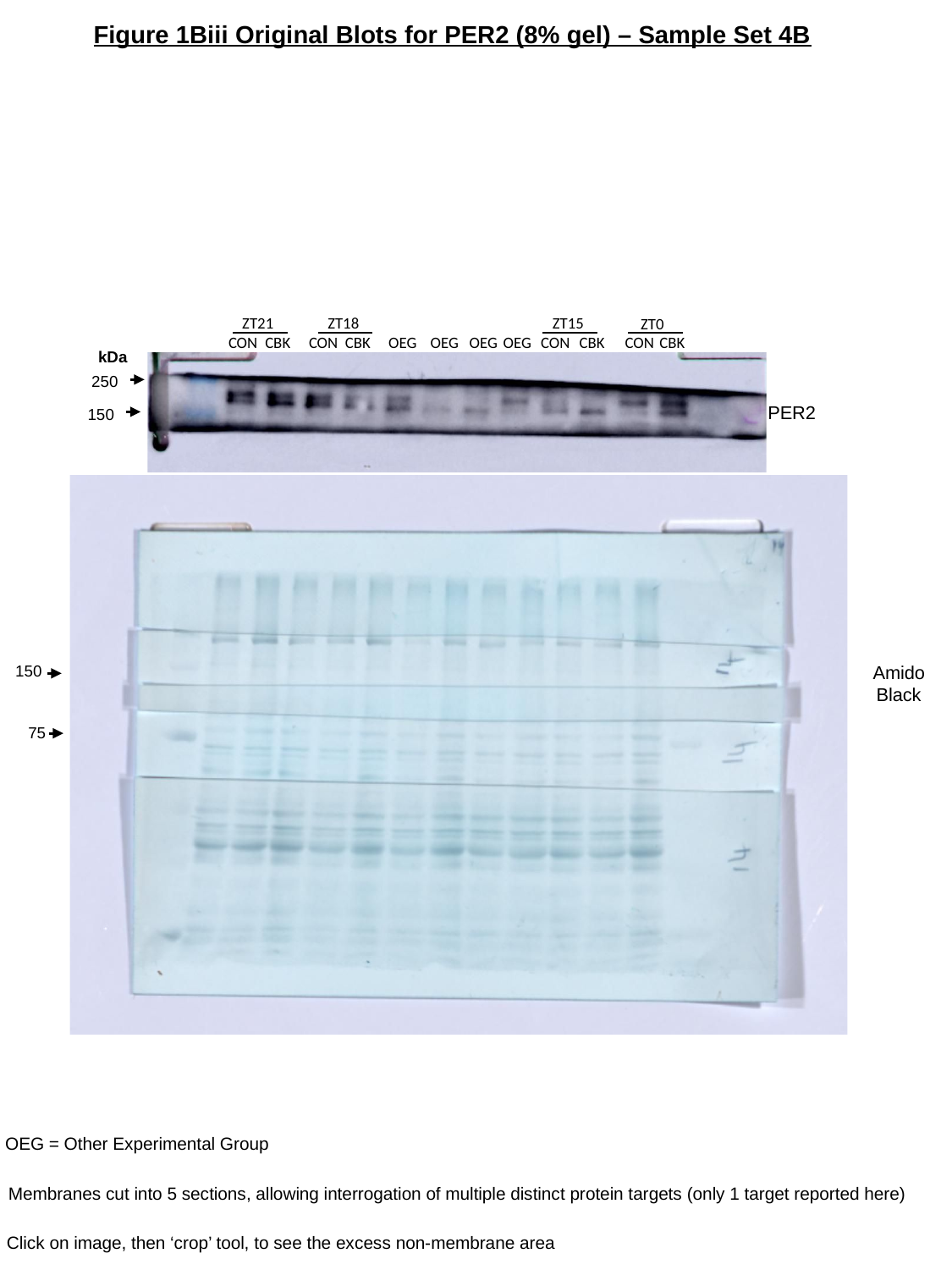

Figure 1Biii Original Blots for PER2 (8% gel) – Sample Set 4B
ZT15
ZT21
ZT18
ZT0
CON
CBK
CON
CBK
OEG
OEG
OEG
OEG
CON
CBK
CON
CBK
kDa
250
PER2
150
150
Amido
Black
75
OEG = Other Experimental Group
Membranes cut into 5 sections, allowing interrogation of multiple distinct protein targets (only 1 target reported here)
Click on image, then ‘crop’ tool, to see the excess non-membrane area

## Slide 35
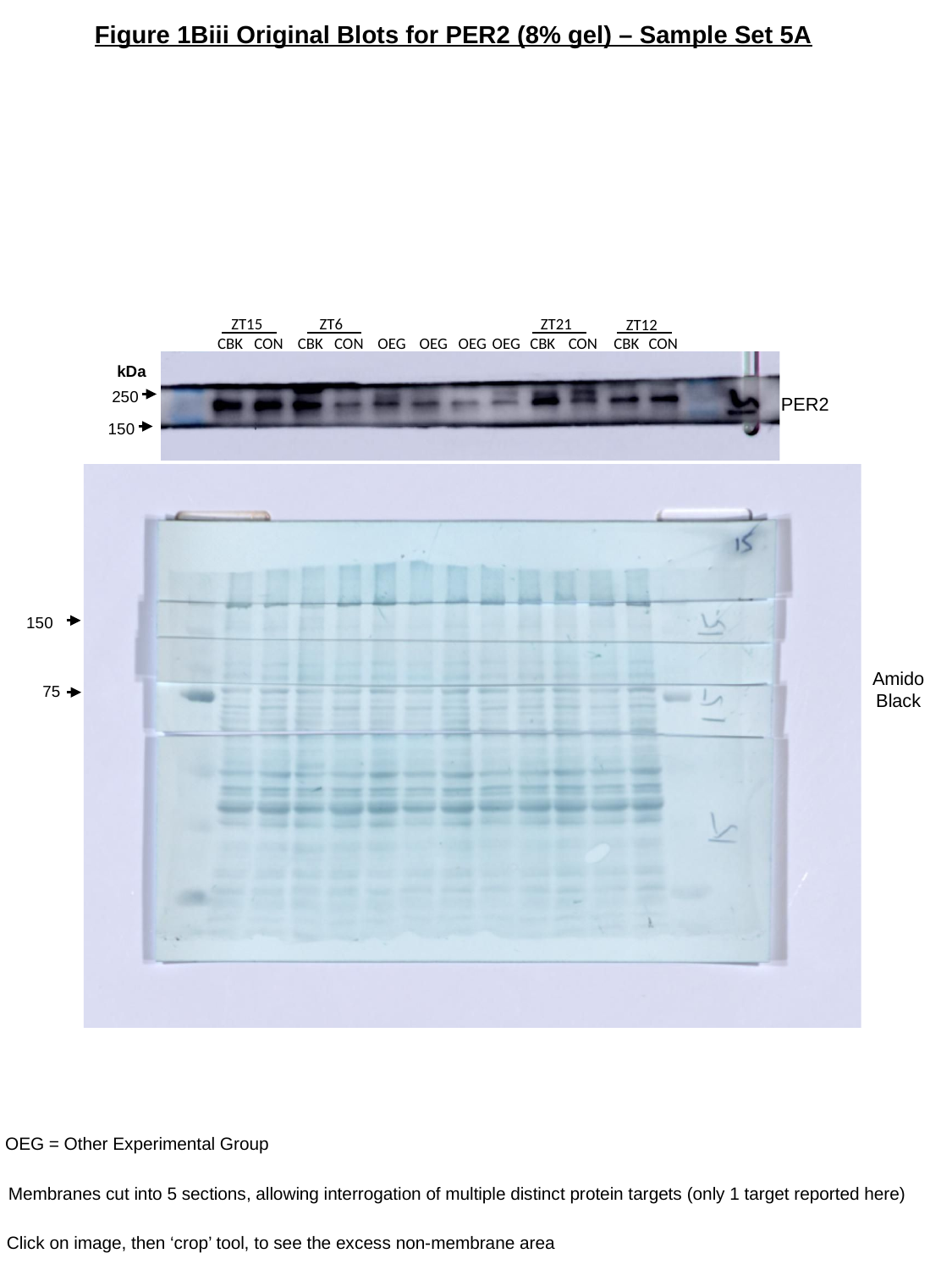

Figure 1Biii Original Blots for PER2 (8% gel) – Sample Set 5A
ZT21
ZT15
ZT6
ZT12
CBK
CON
CBK
CON
OEG
OEG
OEG
OEG
CBK
CON
CBK
CON
kDa
250
PER2
150
150
Amido
Black
75
OEG = Other Experimental Group
Membranes cut into 5 sections, allowing interrogation of multiple distinct protein targets (only 1 target reported here)
Click on image, then ‘crop’ tool, to see the excess non-membrane area

## Slide 36
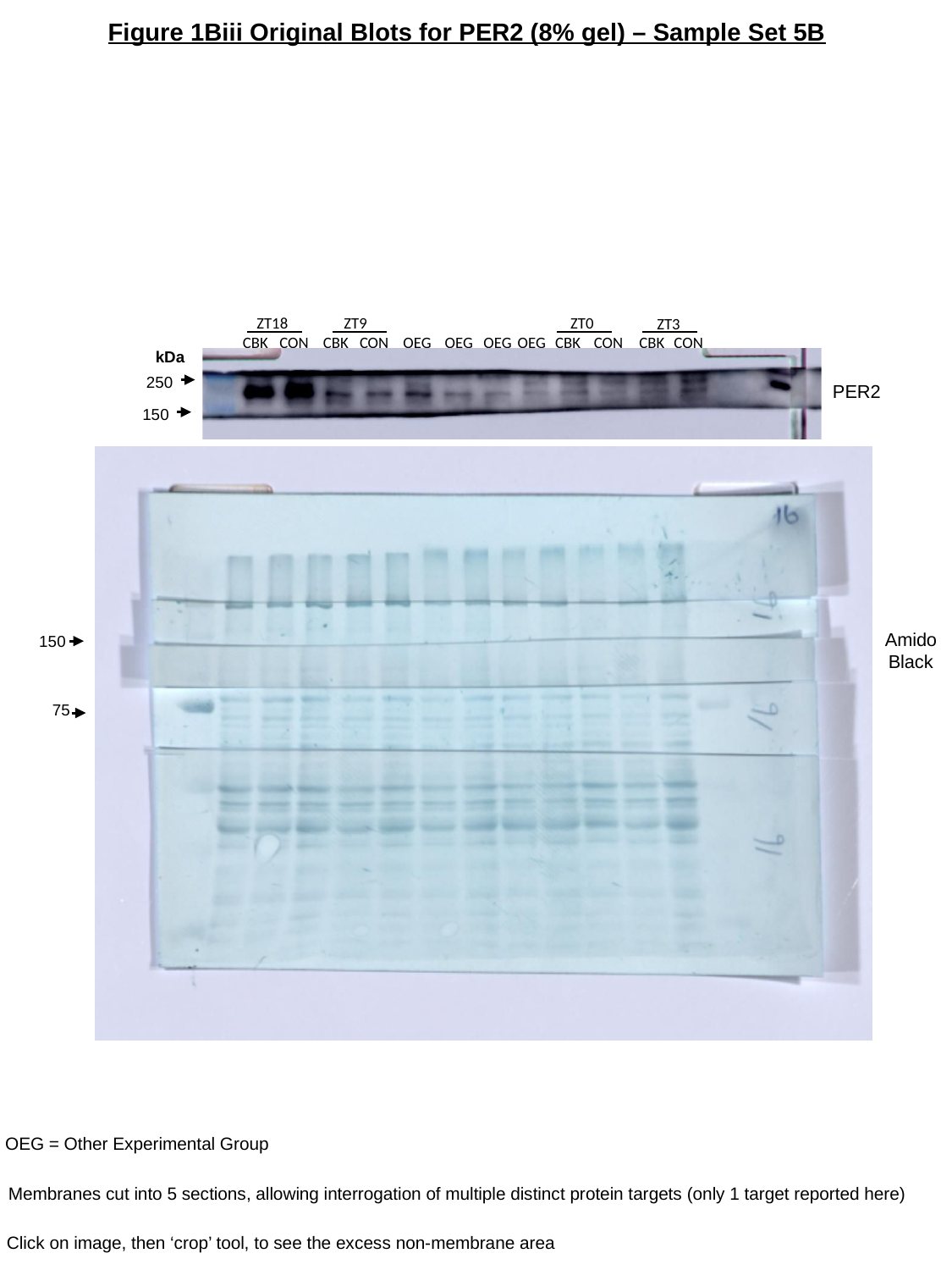

Figure 1Biii Original Blots for PER2 (8% gel) – Sample Set 5B
ZT0
ZT18
ZT9
ZT3
CBK
CON
CBK
CON
OEG
OEG
OEG
OEG
CBK
CON
CBK
CON
kDa
250
PER2
150
Amido
Black
150
75
OEG = Other Experimental Group
Membranes cut into 5 sections, allowing interrogation of multiple distinct protein targets (only 1 target reported here)
Click on image, then ‘crop’ tool, to see the excess non-membrane area

## Slide 37
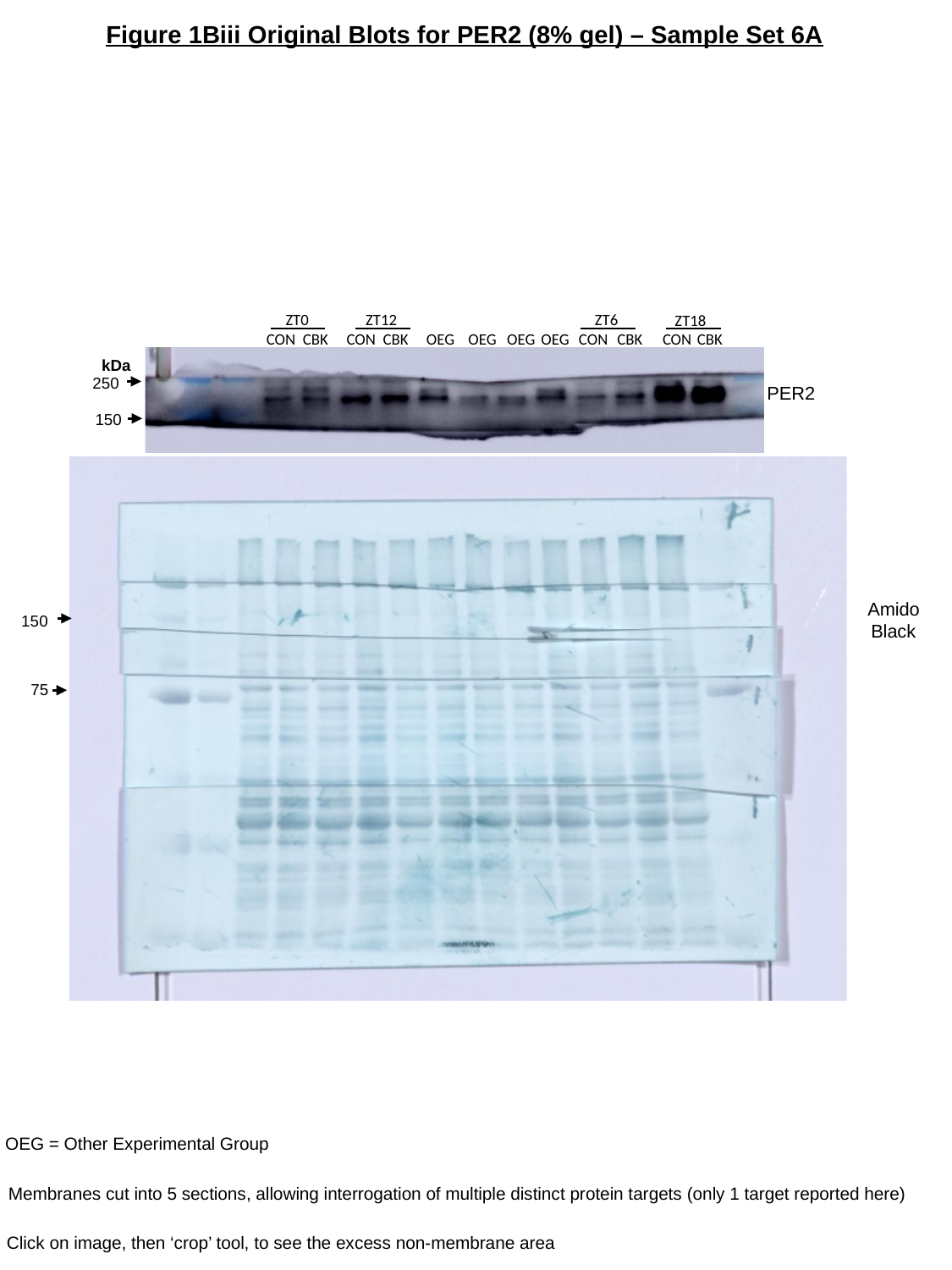

Figure 1Biii Original Blots for PER2 (8% gel) – Sample Set 6A
ZT6
ZT0
ZT12
ZT18
CON
CBK
CON
CBK
OEG
OEG
OEG
OEG
CON
CBK
CON
CBK
kDa
250
PER2
150
Amido
Black
150
75
OEG = Other Experimental Group
Membranes cut into 5 sections, allowing interrogation of multiple distinct protein targets (only 1 target reported here)
Click on image, then ‘crop’ tool, to see the excess non-membrane area

## Slide 38
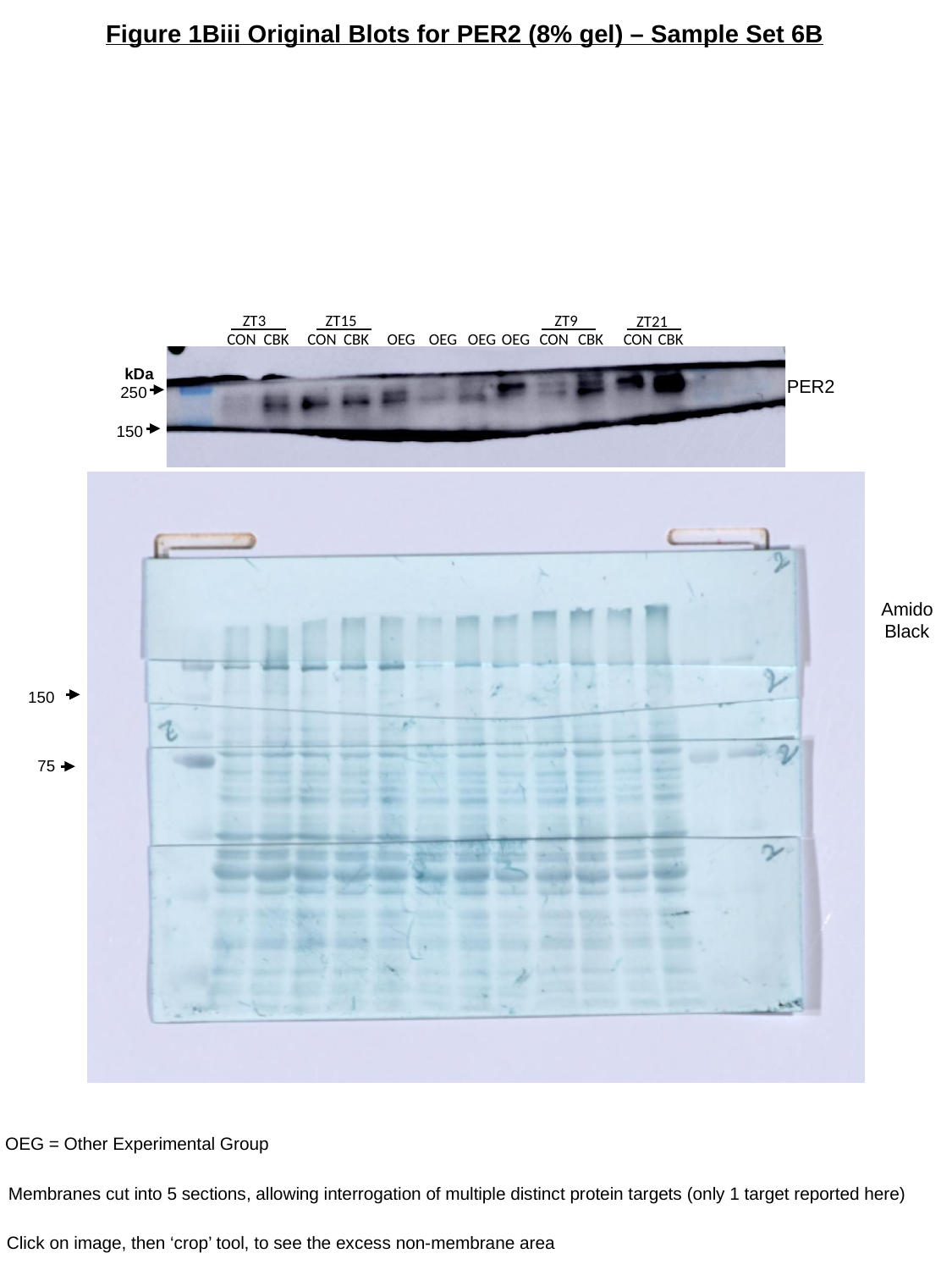

Figure 1Biii Original Blots for PER2 (8% gel) – Sample Set 6B
ZT9
ZT3
ZT15
ZT21
CON
CBK
CON
CBK
OEG
OEG
OEG
OEG
CON
CBK
CON
CBK
kDa
PER2
250
150
Amido
Black
150
75
OEG = Other Experimental Group
Membranes cut into 5 sections, allowing interrogation of multiple distinct protein targets (only 1 target reported here)
Click on image, then ‘crop’ tool, to see the excess non-membrane area

## Slide 39
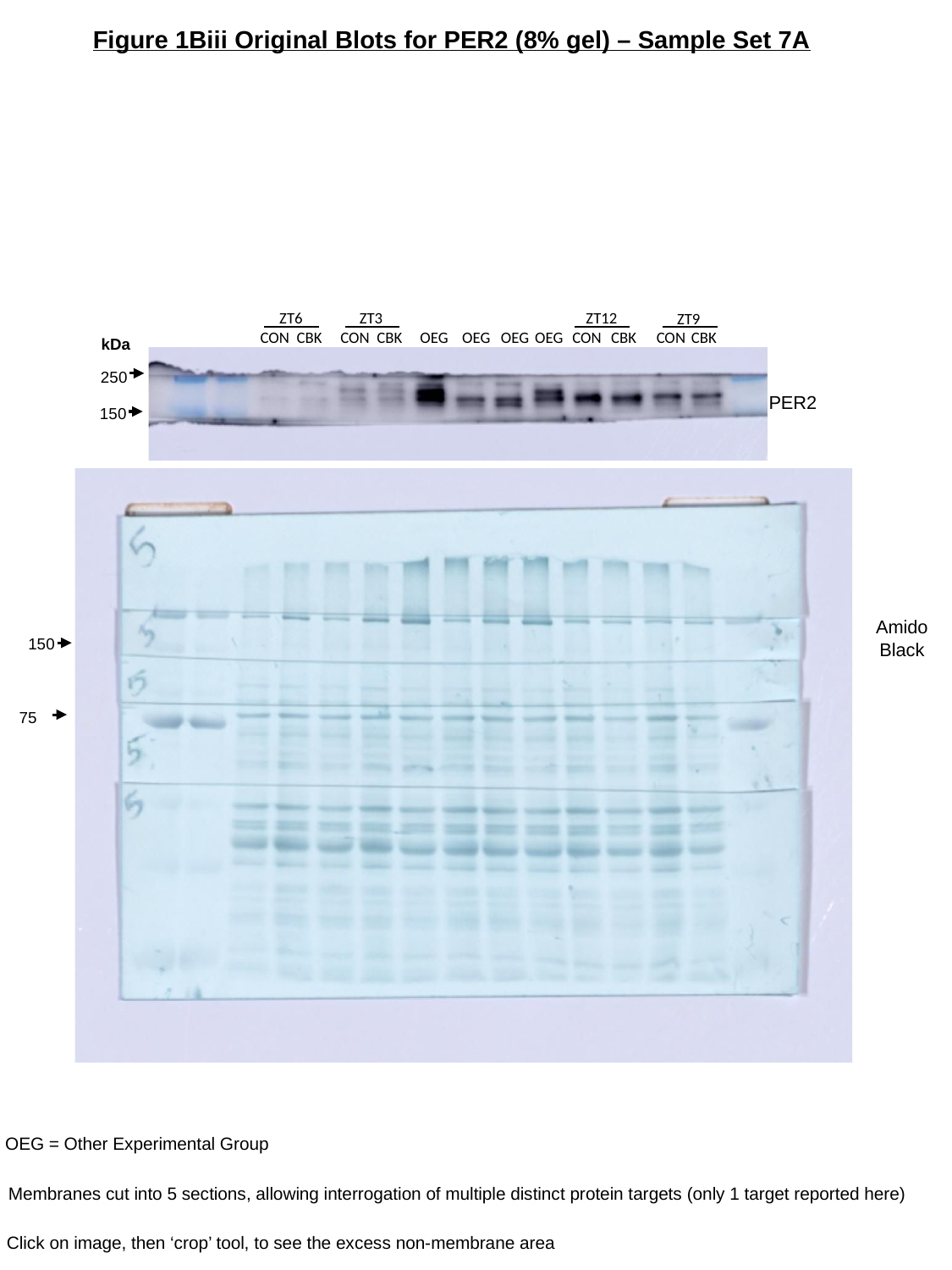

Figure 1Biii Original Blots for PER2 (8% gel) – Sample Set 7A
ZT12
ZT6
ZT3
ZT9
CON
CBK
CON
CBK
OEG
OEG
OEG
OEG
CON
CBK
CON
CBK
kDa
250
PER2
150
Amido
Black
150
75
OEG = Other Experimental Group
Membranes cut into 5 sections, allowing interrogation of multiple distinct protein targets (only 1 target reported here)
Click on image, then ‘crop’ tool, to see the excess non-membrane area

## Slide 40
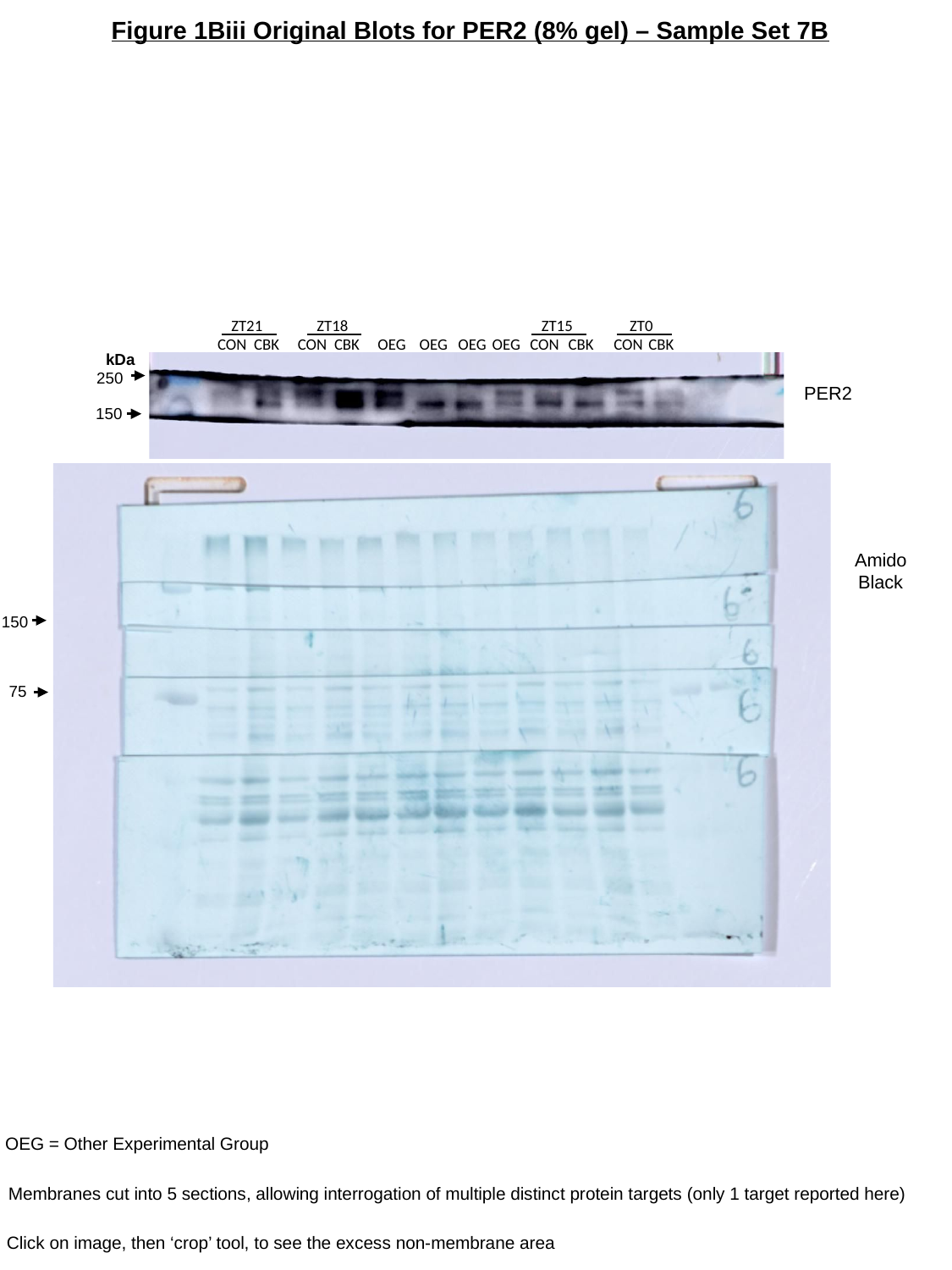

Figure 1Biii Original Blots for PER2 (8% gel) – Sample Set 7B
ZT15
ZT21
ZT18
ZT0
CON
CBK
CON
CBK
OEG
OEG
OEG
OEG
CON
CBK
CON
CBK
kDa
250
PER2
150
Amido
Black
150
75
OEG = Other Experimental Group
Membranes cut into 5 sections, allowing interrogation of multiple distinct protein targets (only 1 target reported here)
Click on image, then ‘crop’ tool, to see the excess non-membrane area

## Slide 41
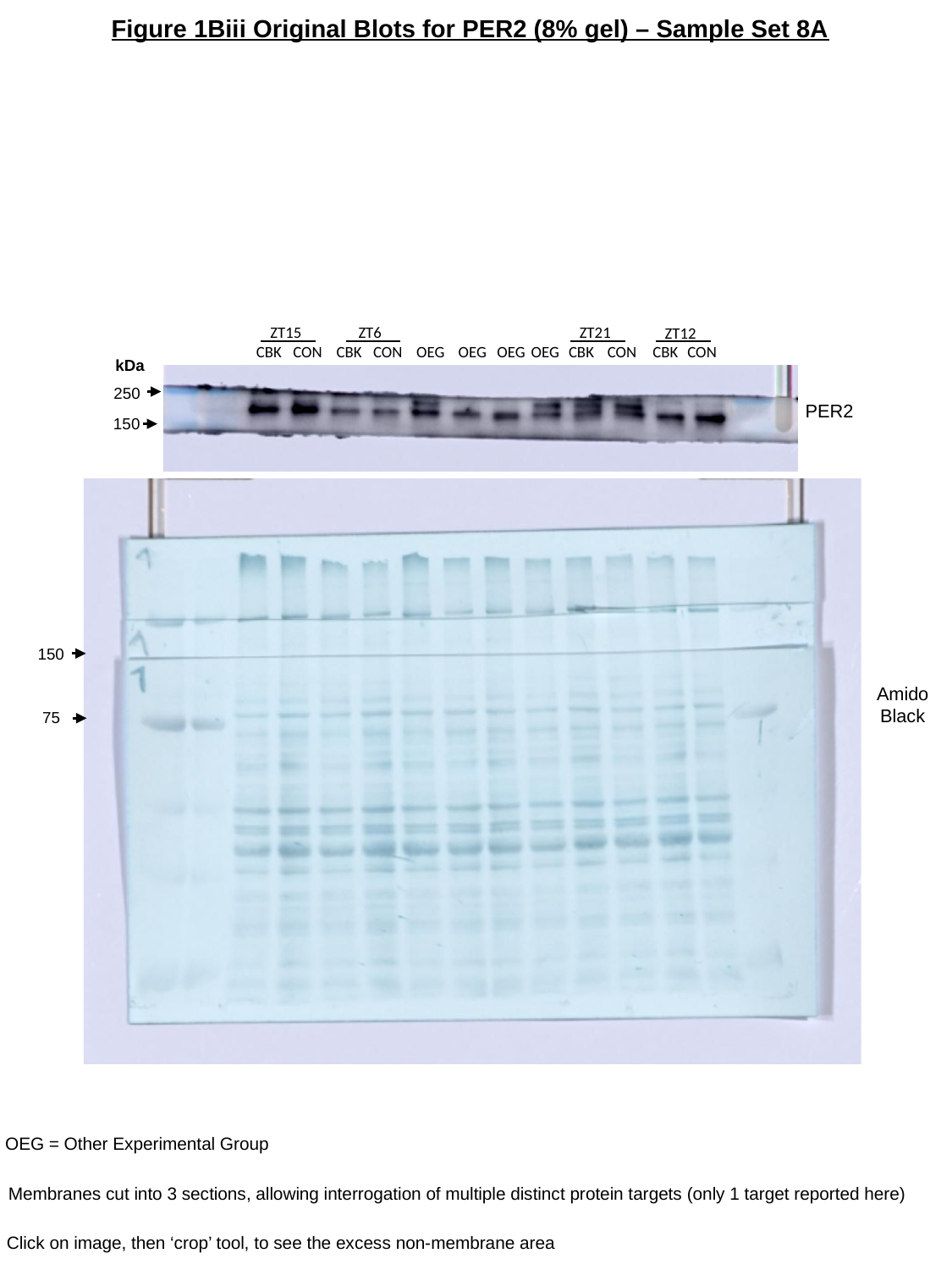

Figure 1Biii Original Blots for PER2 (8% gel) – Sample Set 8A
ZT21
ZT15
ZT6
ZT12
CBK
CON
CBK
CON
OEG
OEG
OEG
OEG
CBK
CON
CBK
CON
kDa
250
PER2
150
150
Amido
Black
75
OEG = Other Experimental Group
Membranes cut into 3 sections, allowing interrogation of multiple distinct protein targets (only 1 target reported here)
Click on image, then ‘crop’ tool, to see the excess non-membrane area

## Slide 42
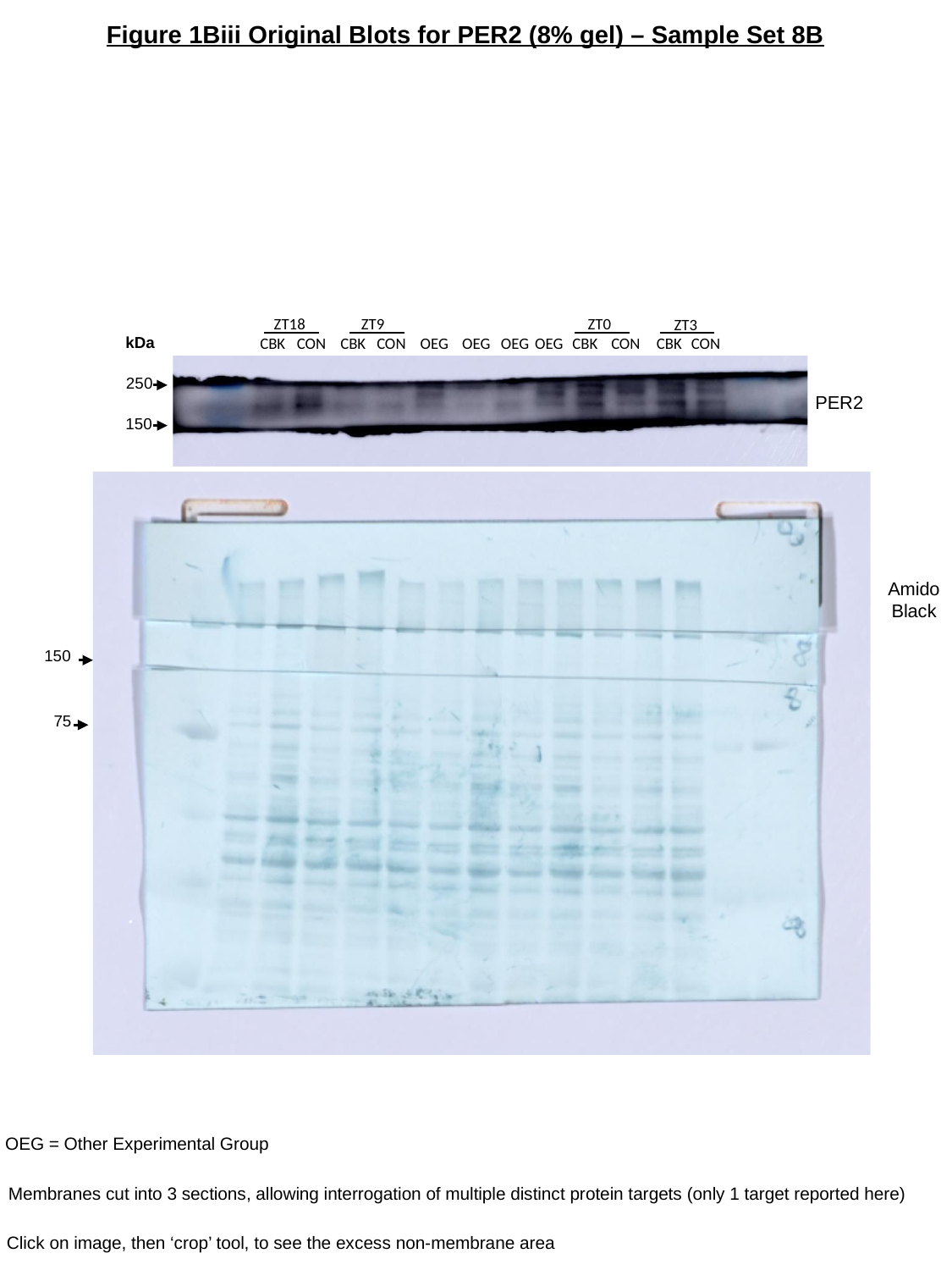

Figure 1Biii Original Blots for PER2 (8% gel) – Sample Set 8B
ZT0
ZT18
ZT9
ZT3
kDa
CBK
CON
CBK
CON
OEG
OEG
OEG
OEG
CBK
CON
CBK
CON
250
PER2
150
Amido
Black
150
75
OEG = Other Experimental Group
Membranes cut into 3 sections, allowing interrogation of multiple distinct protein targets (only 1 target reported here)
Click on image, then ‘crop’ tool, to see the excess non-membrane area

## Slide 43
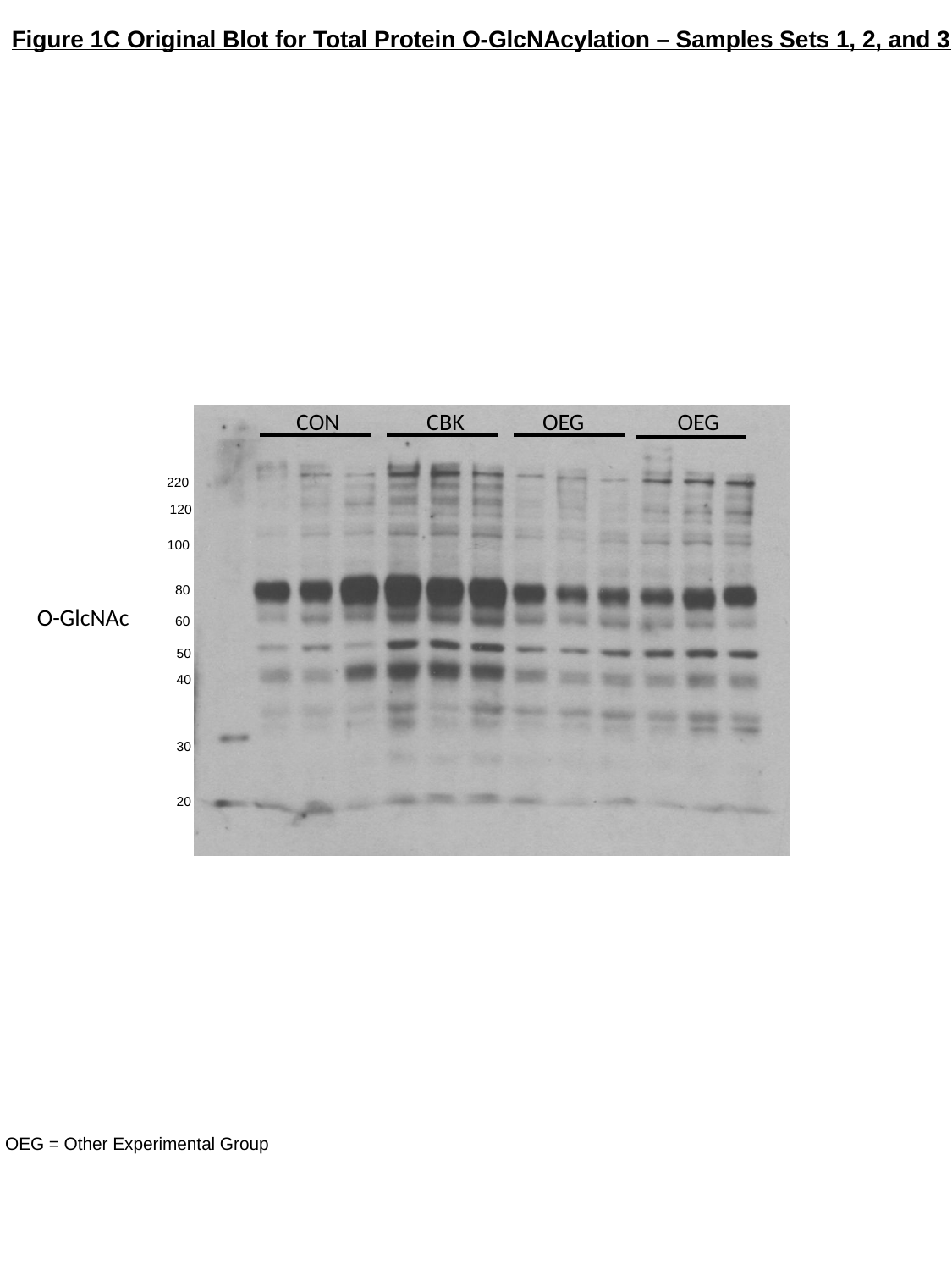

Figure 1C Original Blot for Total Protein O-GlcNAcylation – Samples Sets 1, 2, and 3
CON
CBK
OEG
OEG
220
120
100
80
O-GlcNAc
60
50
40
30
20
OEG = Other Experimental Group

## Slide 44
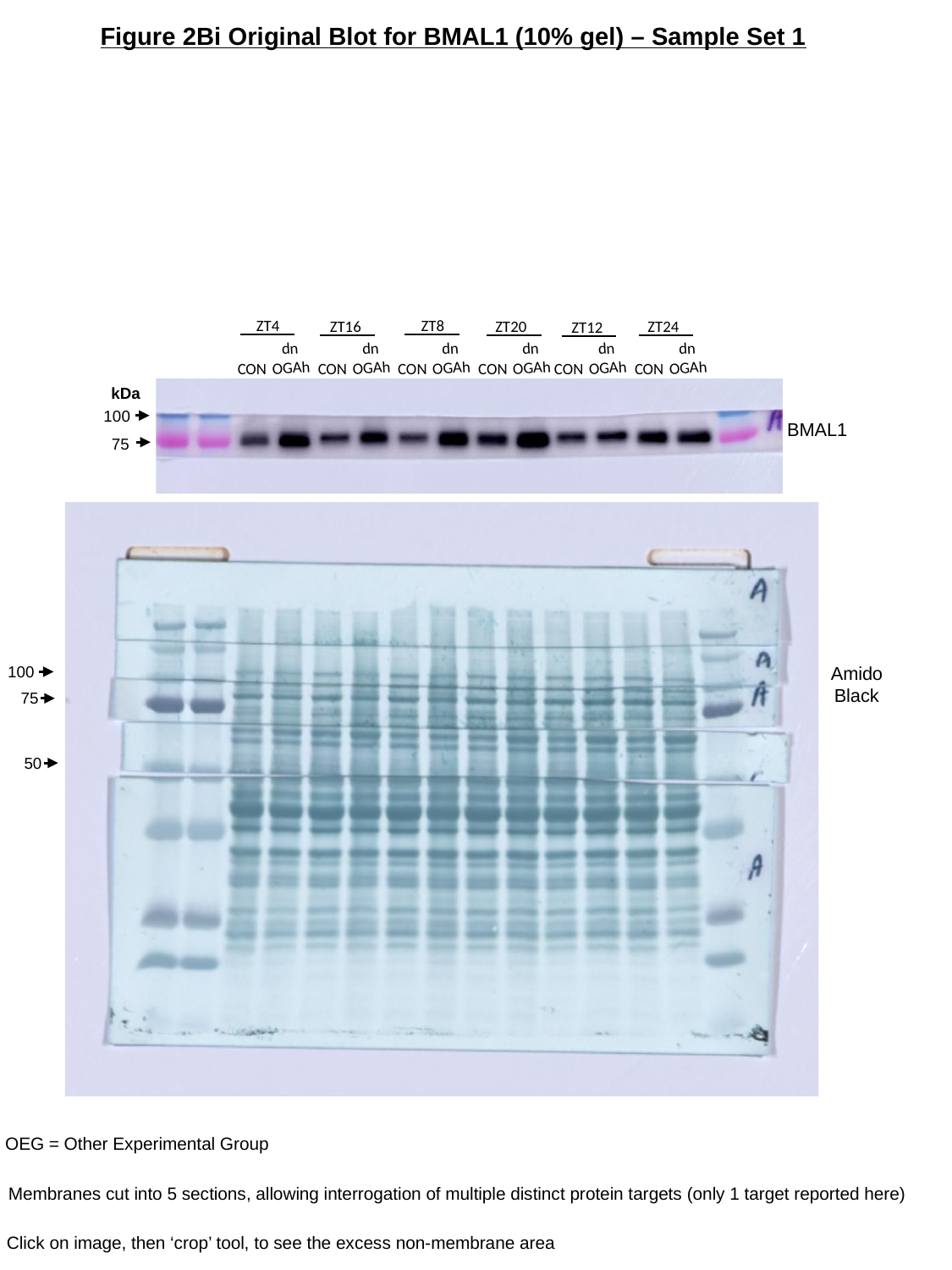

Figure 2Bi Original Blot for BMAL1 (10% gel) – Sample Set 1
ZT4
ZT8
ZT16
ZT24
ZT20
ZT12
dn
OGAh
dn
OGAh
dn
OGAh
dn
OGAh
dn
OGAh
dn
OGAh
CON
CON
CON
CON
CON
CON
kDa
100
BMAL1
75
100
Amido
Black
75
50
OEG = Other Experimental Group
Membranes cut into 5 sections, allowing interrogation of multiple distinct protein targets (only 1 target reported here)
Click on image, then ‘crop’ tool, to see the excess non-membrane area

## Slide 45
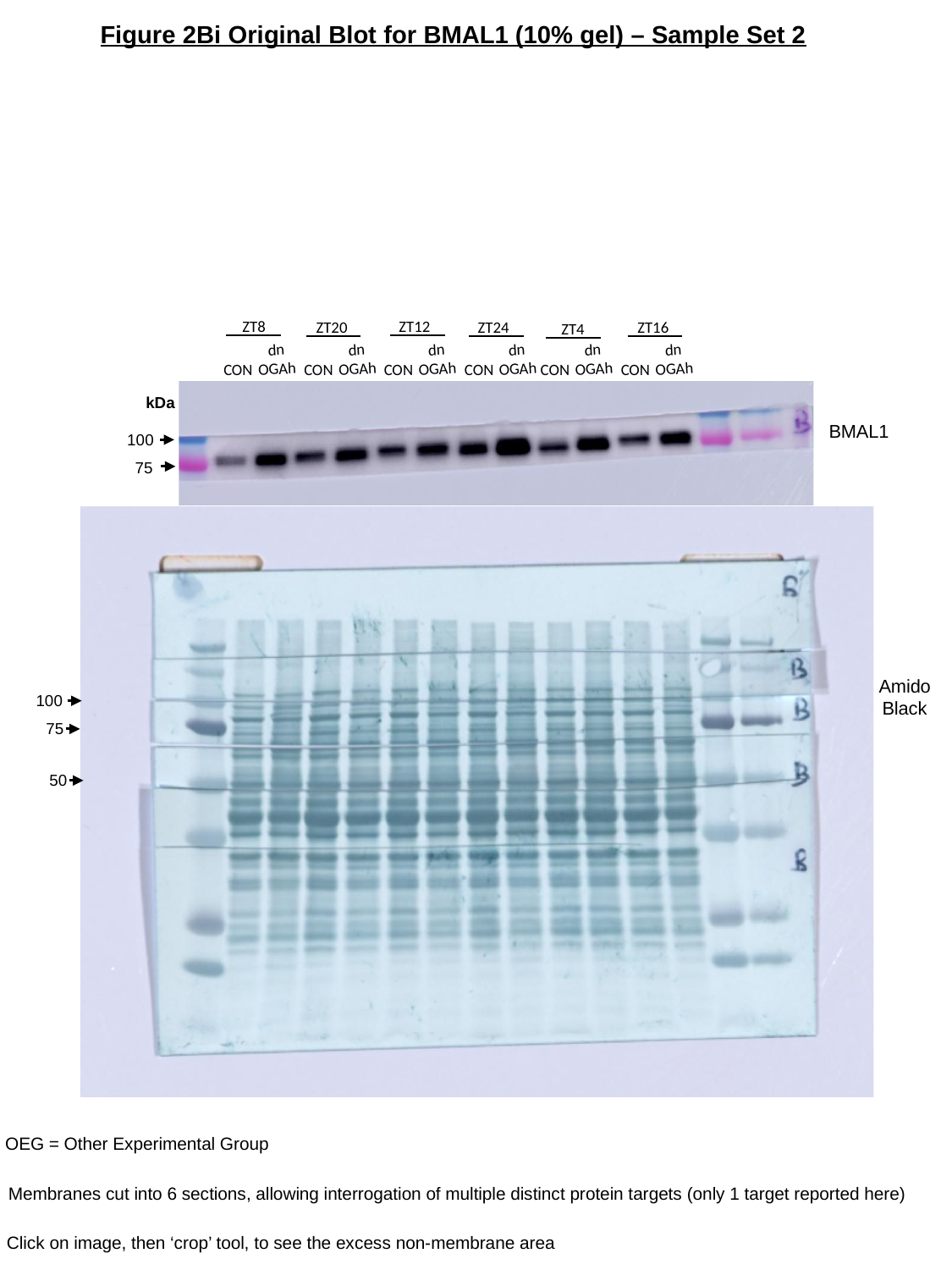

Figure 2Bi Original Blot for BMAL1 (10% gel) – Sample Set 2
ZT8
ZT12
ZT20
ZT16
ZT24
ZT4
dn
OGAh
dn
OGAh
dn
OGAh
dn
OGAh
dn
OGAh
dn
OGAh
CON
CON
CON
CON
CON
CON
kDa
BMAL1
100
75
Amido
Black
100
75
50
OEG = Other Experimental Group
Membranes cut into 6 sections, allowing interrogation of multiple distinct protein targets (only 1 target reported here)
Click on image, then ‘crop’ tool, to see the excess non-membrane area

## Slide 46
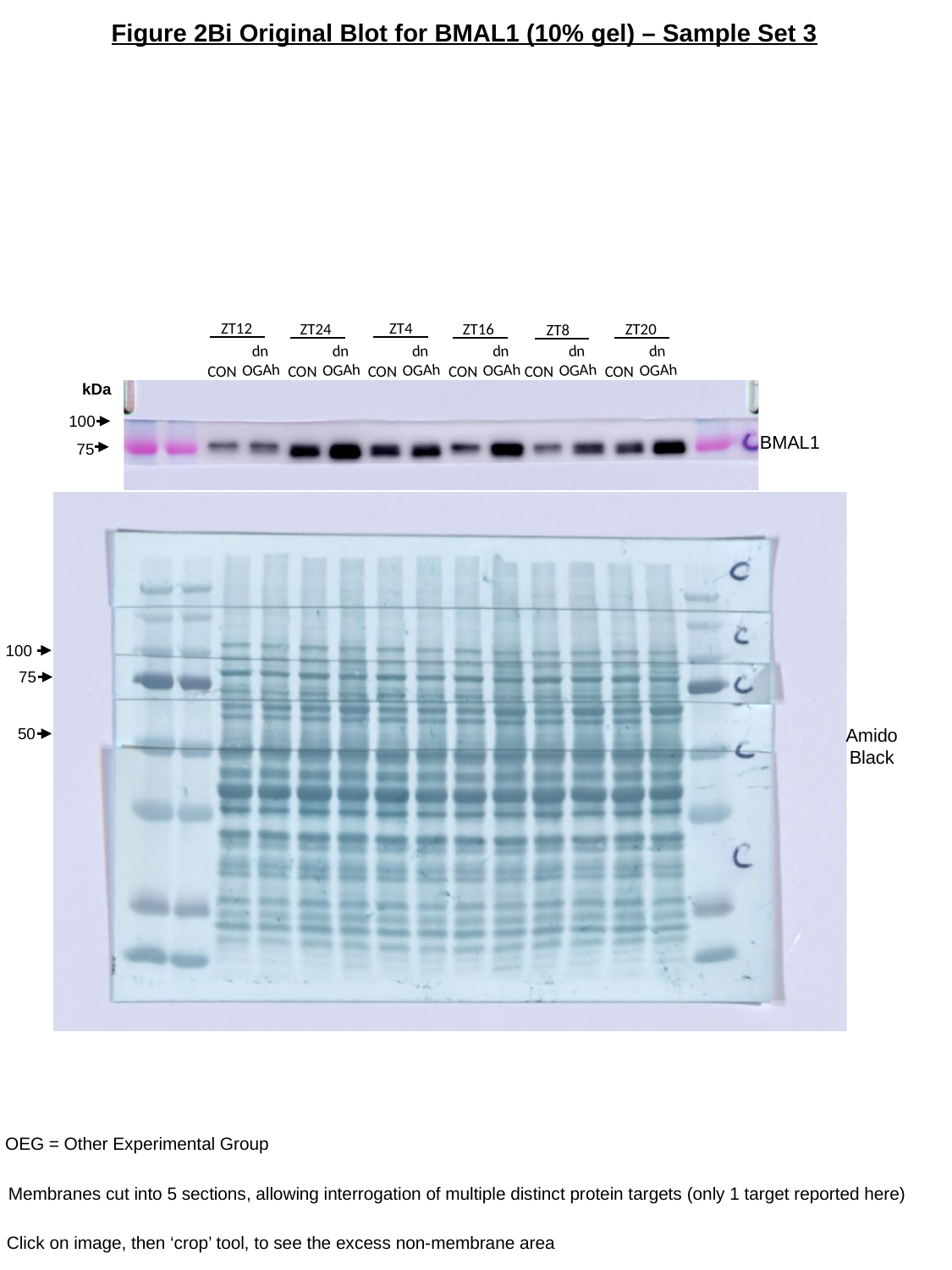

Figure 2Bi Original Blot for BMAL1 (10% gel) – Sample Set 3
ZT12
ZT4
ZT24
ZT20
ZT16
ZT8
dn
OGAh
dn
OGAh
dn
OGAh
dn
OGAh
dn
OGAh
dn
OGAh
CON
CON
CON
CON
CON
CON
kDa
100
BMAL1
75
100
75
50
Amido
Black
OEG = Other Experimental Group
Membranes cut into 5 sections, allowing interrogation of multiple distinct protein targets (only 1 target reported here)
Click on image, then ‘crop’ tool, to see the excess non-membrane area

## Slide 47
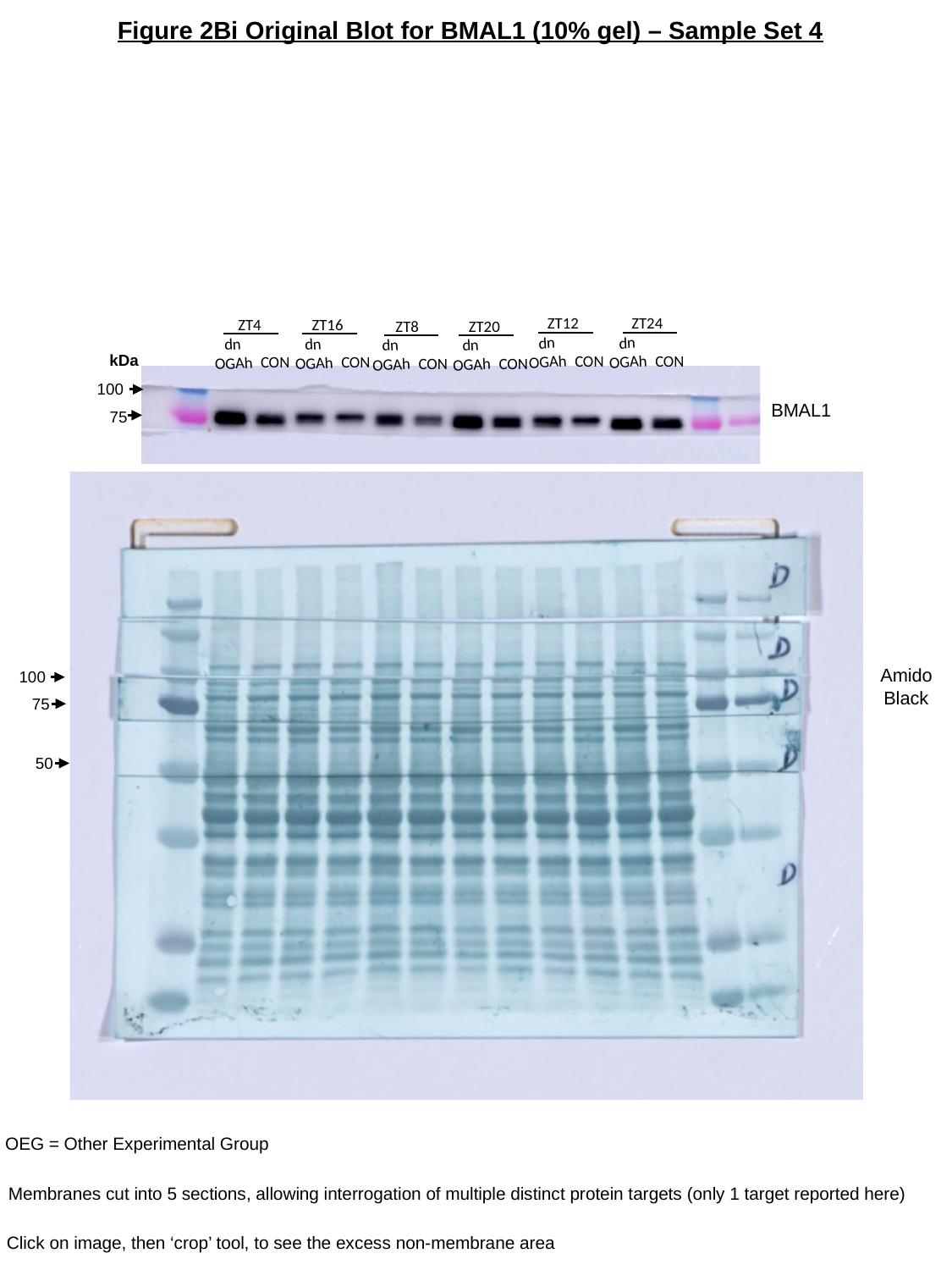

Figure 2Bi Original Blot for BMAL1 (10% gel) – Sample Set 4
ZT12
ZT24
ZT4
ZT16
ZT8
ZT20
dn
OGAh
dn
OGAh
dn
OGAh
dn
OGAh
dn
OGAh
dn
OGAh
kDa
CON
CON
CON
CON
CON
CON
100
BMAL1
75
Amido
Black
100
75
50
OEG = Other Experimental Group
Membranes cut into 5 sections, allowing interrogation of multiple distinct protein targets (only 1 target reported here)
Click on image, then ‘crop’ tool, to see the excess non-membrane area

## Slide 48
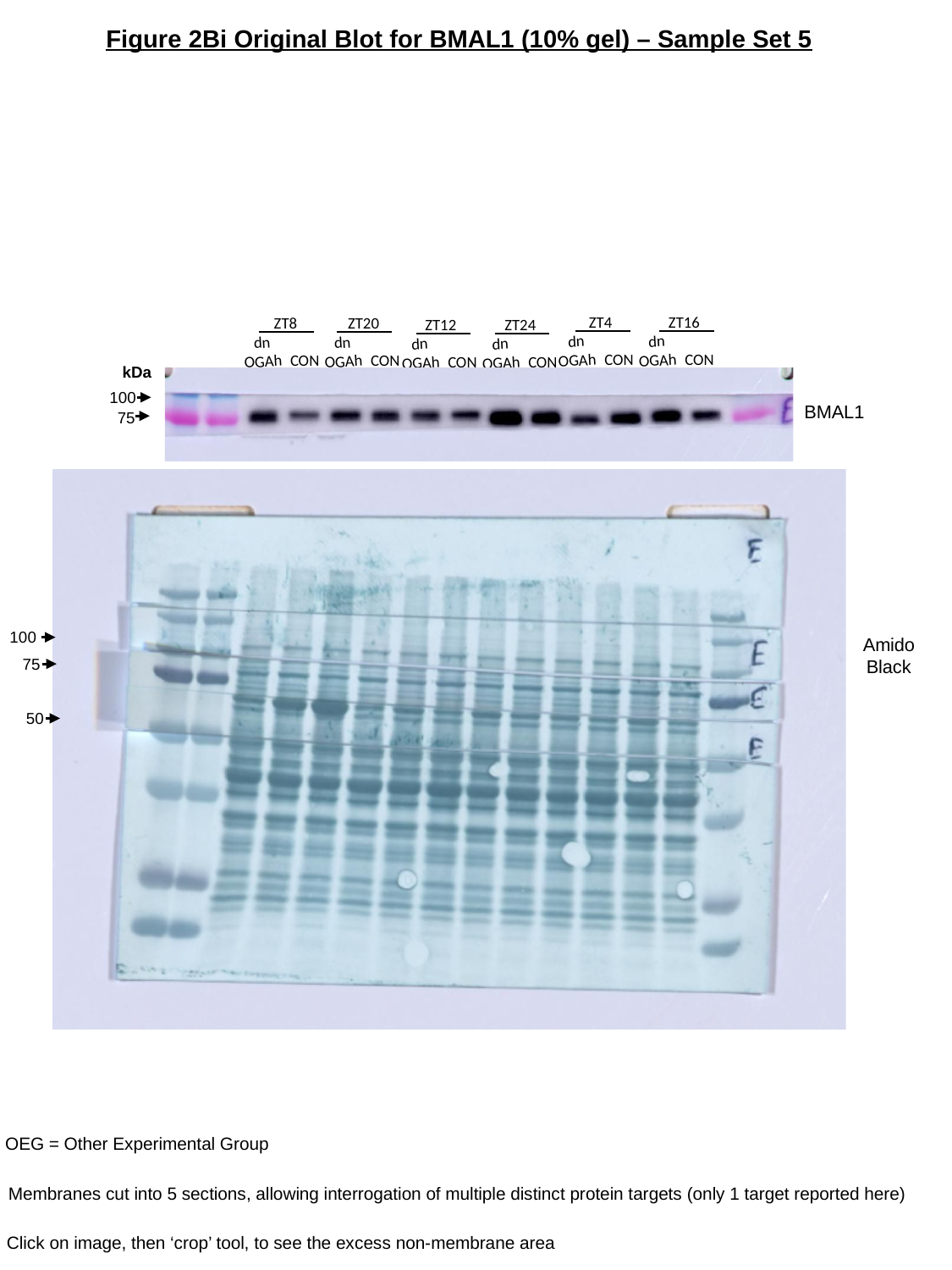

Figure 2Bi Original Blot for BMAL1 (10% gel) – Sample Set 5
ZT4
ZT16
ZT8
ZT20
ZT12
ZT24
dn
OGAh
dn
OGAh
dn
OGAh
dn
OGAh
dn
OGAh
dn
OGAh
CON
CON
CON
CON
CON
CON
kDa
100
BMAL1
75
100
Amido
Black
75
50
OEG = Other Experimental Group
Membranes cut into 5 sections, allowing interrogation of multiple distinct protein targets (only 1 target reported here)
Click on image, then ‘crop’ tool, to see the excess non-membrane area

## Slide 49
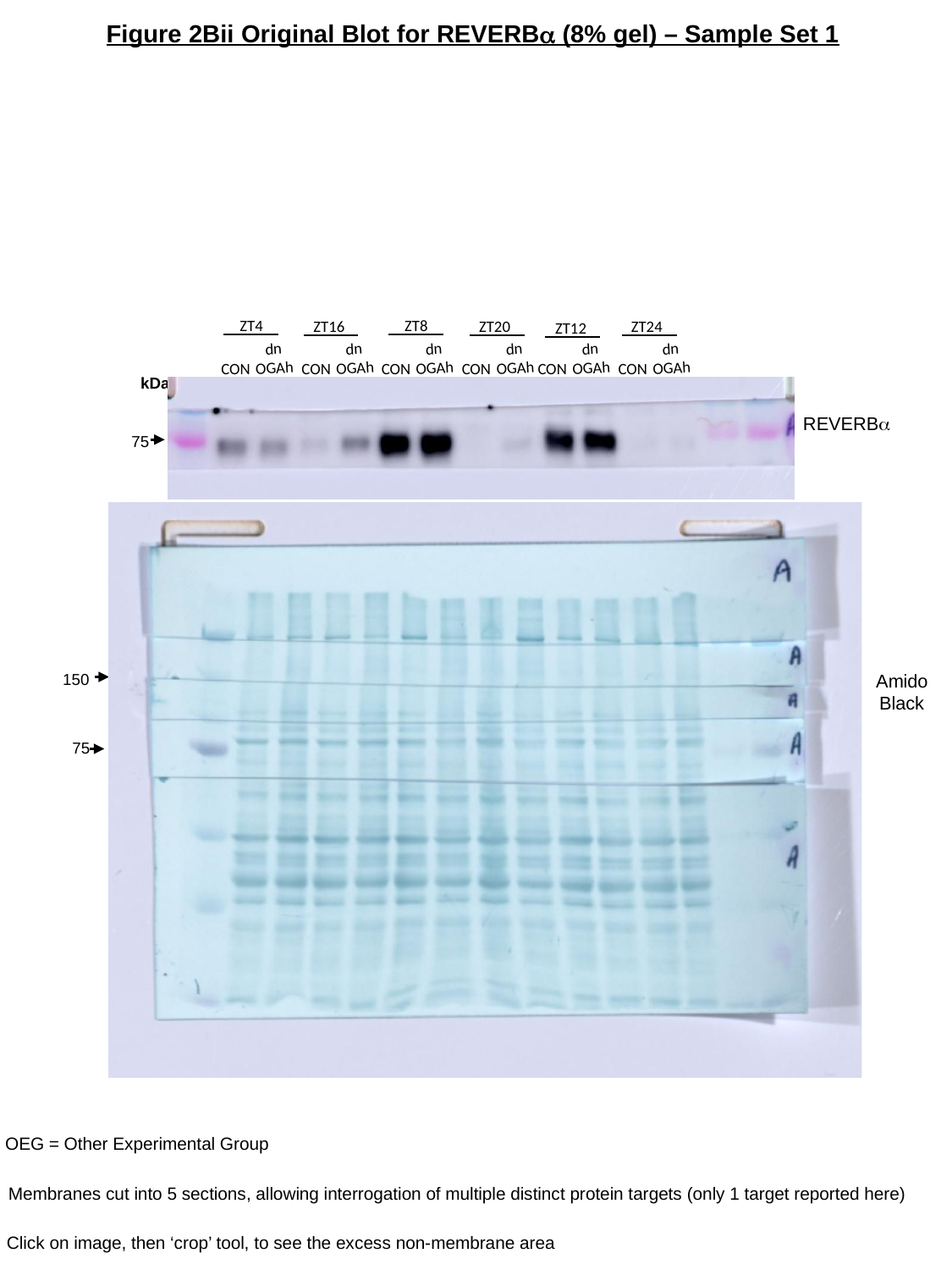

Figure 2Bii Original Blot for REVERBa (8% gel) – Sample Set 1
ZT4
ZT8
ZT16
ZT24
ZT20
ZT12
dn
OGAh
dn
OGAh
dn
OGAh
dn
OGAh
dn
OGAh
dn
OGAh
CON
CON
CON
CON
CON
CON
kDa
REVERBa
75
150
75
Amido
Black
OEG = Other Experimental Group
Membranes cut into 5 sections, allowing interrogation of multiple distinct protein targets (only 1 target reported here)
Click on image, then ‘crop’ tool, to see the excess non-membrane area

## Slide 50
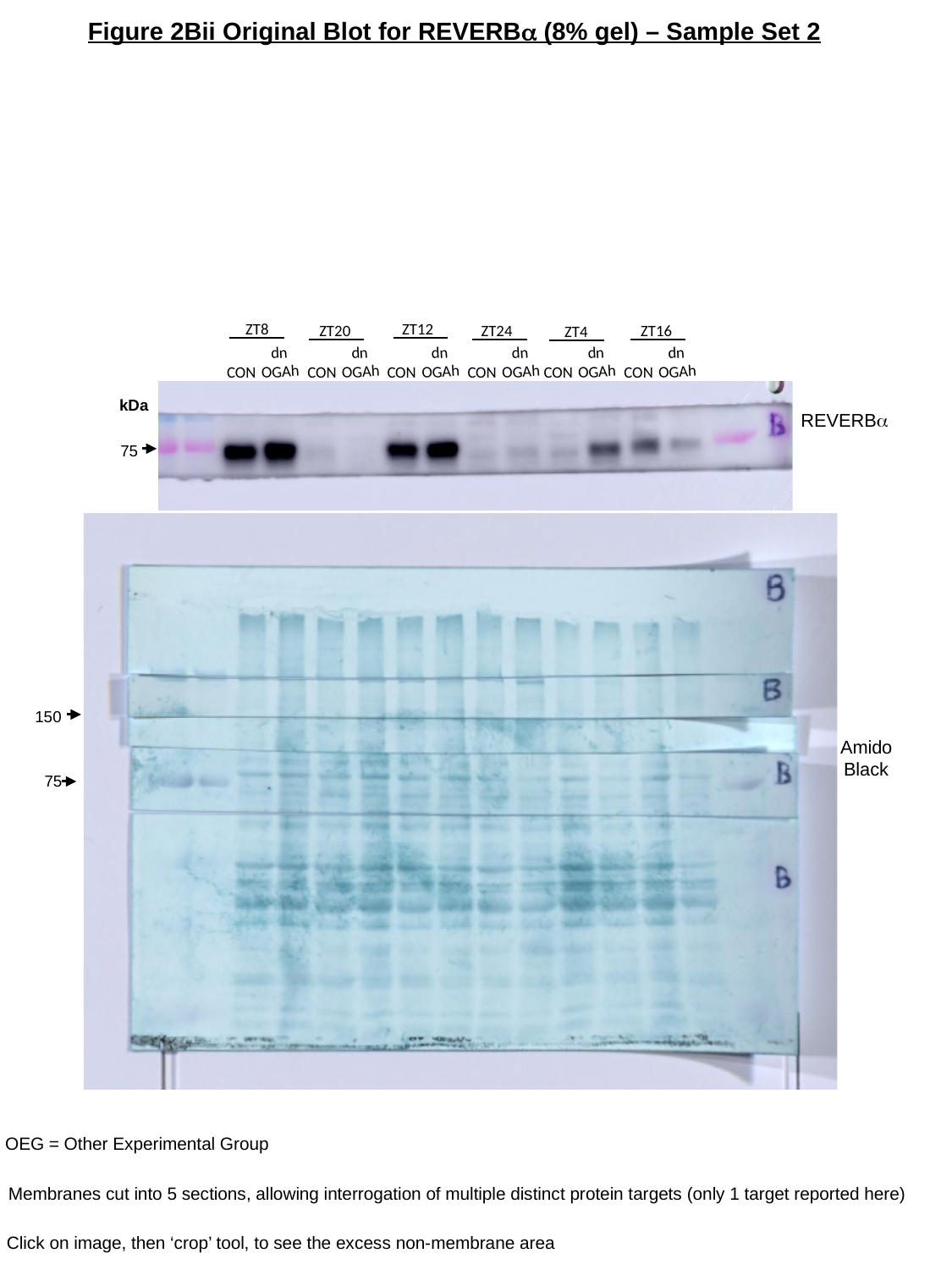

Figure 2Bii Original Blot for REVERBa (8% gel) – Sample Set 2
ZT8
ZT12
ZT20
ZT16
ZT24
ZT4
dn
OGAh
dn
OGAh
dn
OGAh
dn
OGAh
dn
OGAh
dn
OGAh
CON
CON
CON
CON
CON
CON
kDa
REVERBa
75
150
Amido
Black
75
OEG = Other Experimental Group
Membranes cut into 5 sections, allowing interrogation of multiple distinct protein targets (only 1 target reported here)
Click on image, then ‘crop’ tool, to see the excess non-membrane area

## Slide 51
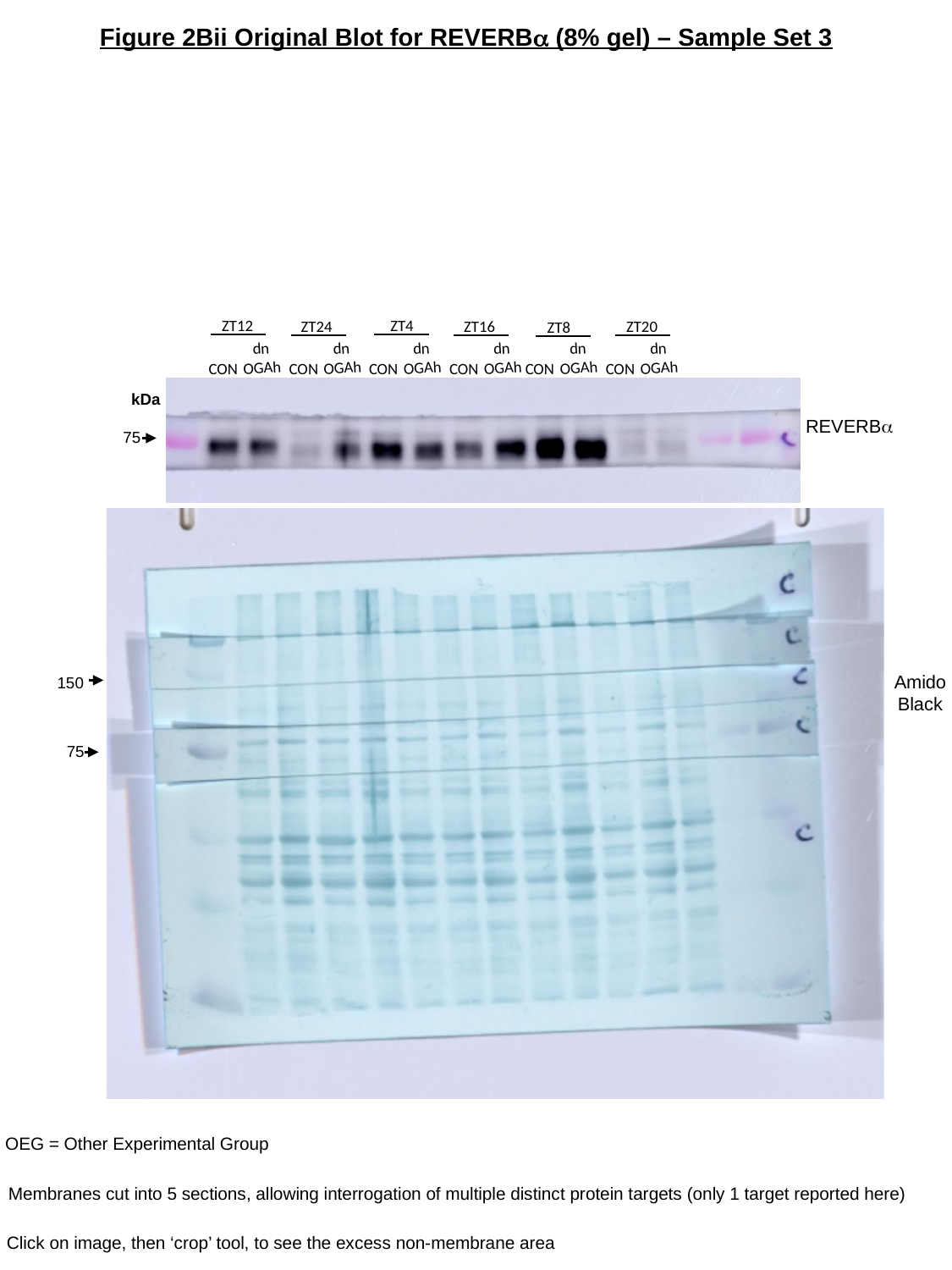

Figure 2Bii Original Blot for REVERBa (8% gel) – Sample Set 3
ZT12
ZT4
ZT24
ZT20
ZT16
ZT8
dn
OGAh
dn
OGAh
dn
OGAh
dn
OGAh
dn
OGAh
dn
OGAh
CON
CON
CON
CON
CON
CON
kDa
REVERBa
75
Amido
Black
150
75
OEG = Other Experimental Group
Membranes cut into 5 sections, allowing interrogation of multiple distinct protein targets (only 1 target reported here)
Click on image, then ‘crop’ tool, to see the excess non-membrane area

## Slide 52
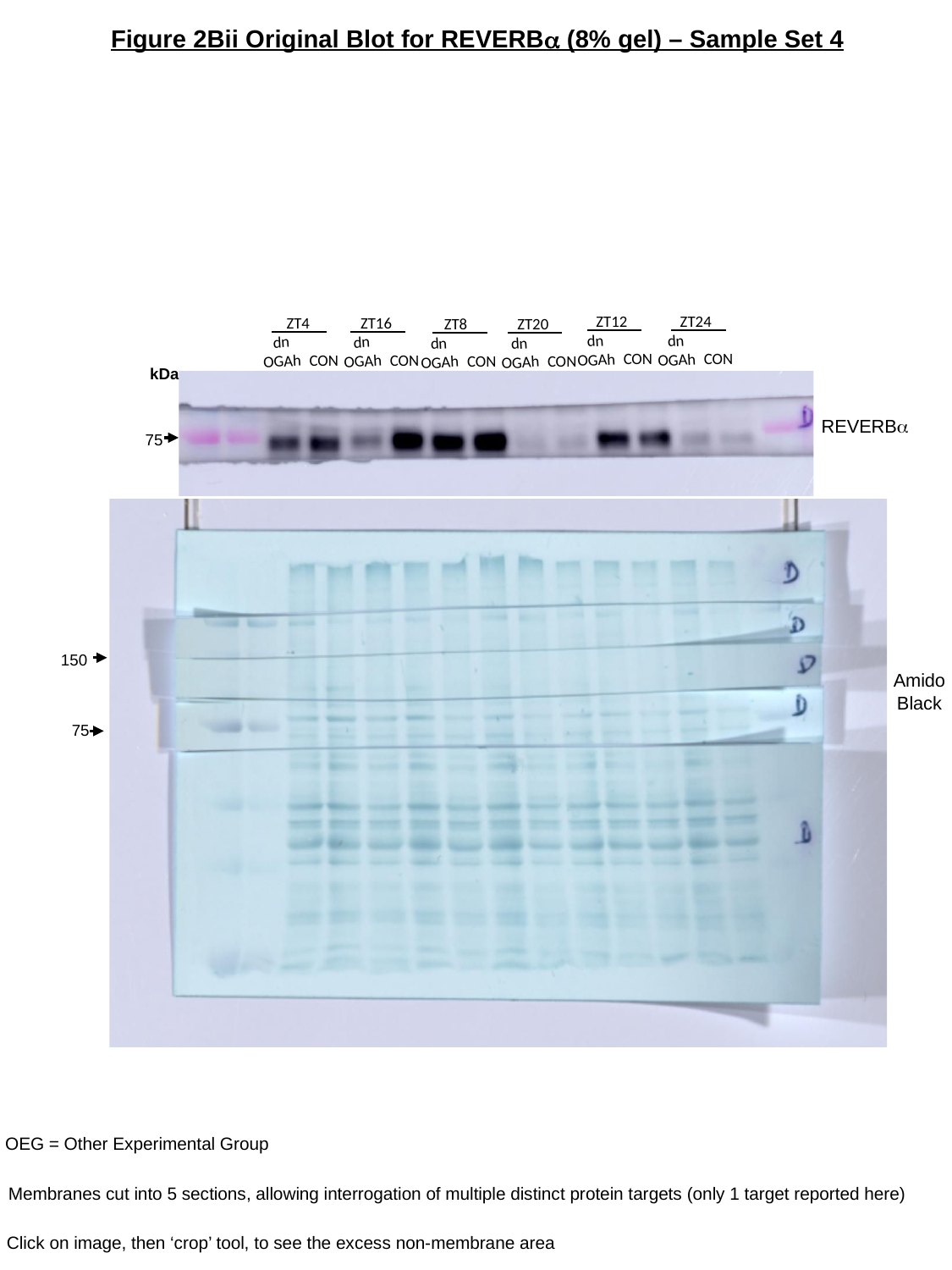

Figure 2Bii Original Blot for REVERBa (8% gel) – Sample Set 4
ZT12
ZT24
ZT4
ZT16
ZT8
ZT20
dn
OGAh
dn
OGAh
dn
OGAh
dn
OGAh
dn
OGAh
dn
OGAh
CON
CON
CON
CON
CON
CON
kDa
REVERBa
75
150
Amido
Black
75
OEG = Other Experimental Group
Membranes cut into 5 sections, allowing interrogation of multiple distinct protein targets (only 1 target reported here)
Click on image, then ‘crop’ tool, to see the excess non-membrane area

## Slide 53
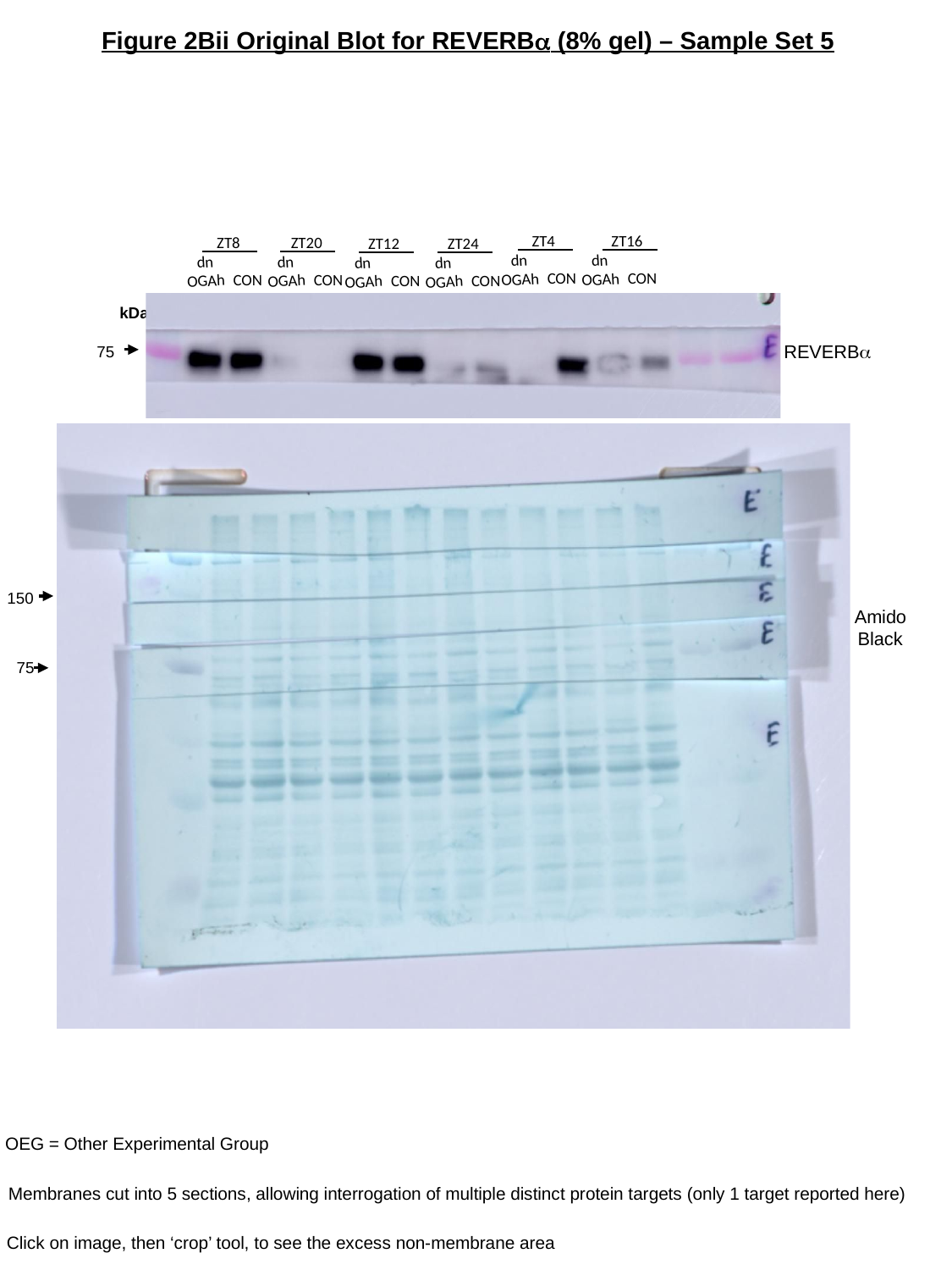

Figure 2Bii Original Blot for REVERBa (8% gel) – Sample Set 5
ZT4
ZT16
ZT8
ZT20
ZT12
ZT24
dn
OGAh
dn
OGAh
dn
OGAh
dn
OGAh
dn
OGAh
dn
OGAh
CON
CON
CON
CON
CON
CON
kDa
REVERBa
75
150
Amido
Black
75
OEG = Other Experimental Group
Membranes cut into 5 sections, allowing interrogation of multiple distinct protein targets (only 1 target reported here)
Click on image, then ‘crop’ tool, to see the excess non-membrane area

## Slide 54
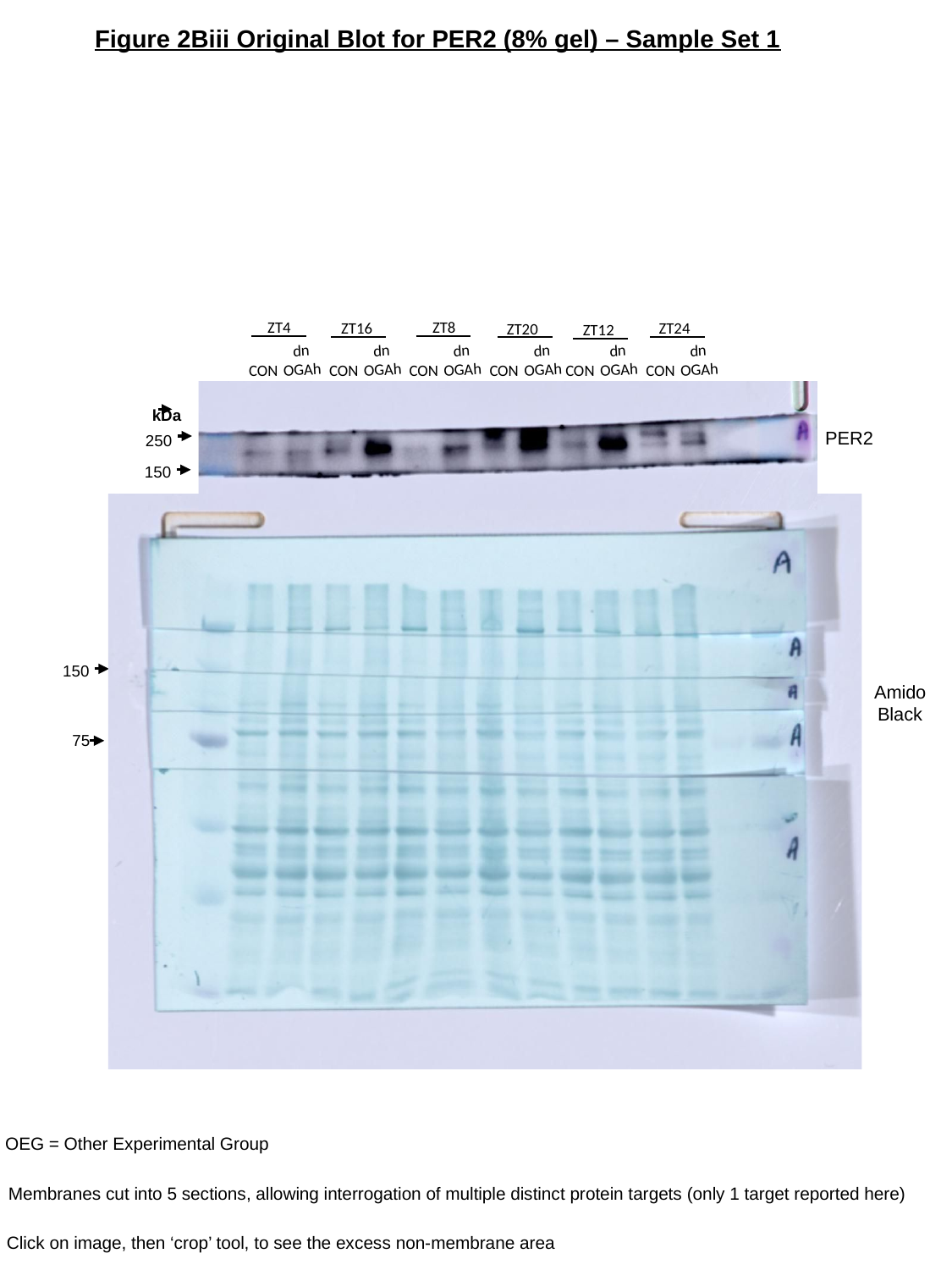

Figure 2Biii Original Blot for PER2 (8% gel) – Sample Set 1
ZT4
ZT8
ZT16
ZT24
ZT20
ZT12
dn
OGAh
dn
OGAh
dn
OGAh
dn
OGAh
dn
OGAh
dn
OGAh
CON
CON
CON
CON
CON
CON
kDa
PER2
250
150
150
75
Amido
Black
OEG = Other Experimental Group
Membranes cut into 5 sections, allowing interrogation of multiple distinct protein targets (only 1 target reported here)
Click on image, then ‘crop’ tool, to see the excess non-membrane area

## Slide 55
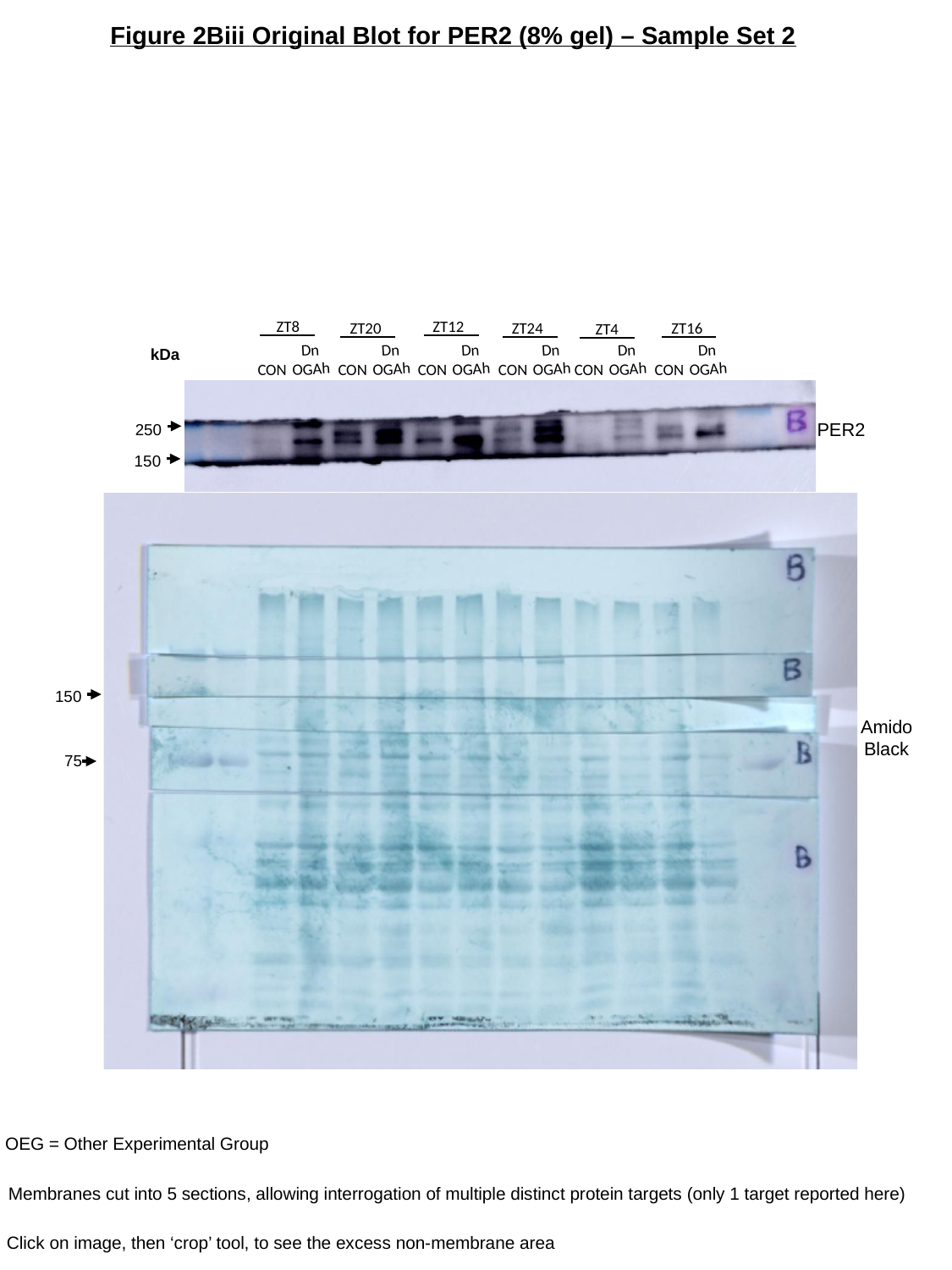

Figure 2Biii Original Blot for PER2 (8% gel) – Sample Set 2
ZT8
ZT12
ZT20
ZT16
ZT24
ZT4
kDa
Dn
OGAh
Dn
OGAh
Dn
OGAh
Dn
OGAh
Dn
OGAh
Dn
OGAh
CON
CON
CON
CON
CON
CON
PER2
250
150
150
Amido
Black
75
OEG = Other Experimental Group
Membranes cut into 5 sections, allowing interrogation of multiple distinct protein targets (only 1 target reported here)
Click on image, then ‘crop’ tool, to see the excess non-membrane area

## Slide 56
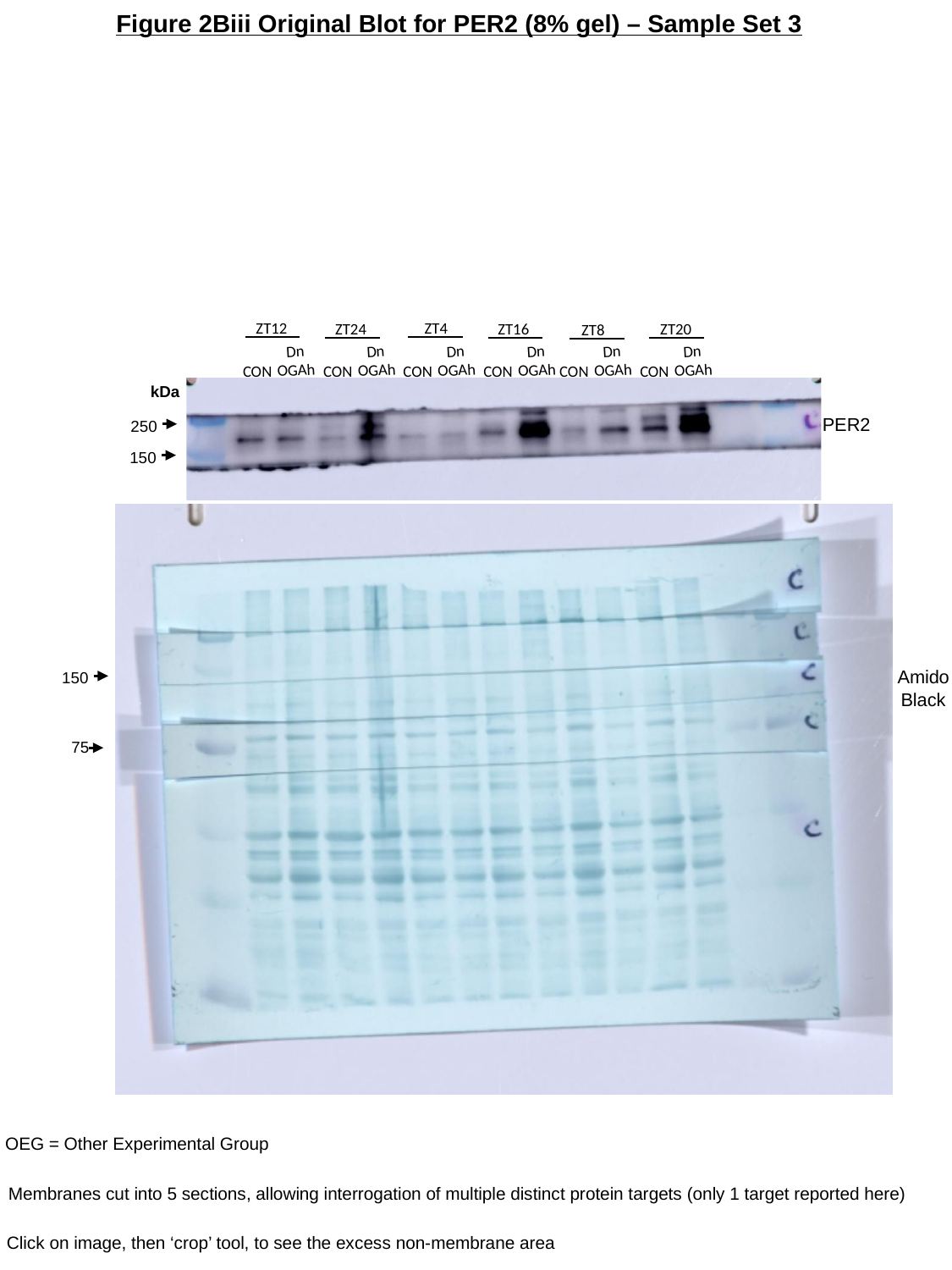

Figure 2Biii Original Blot for PER2 (8% gel) – Sample Set 3
ZT12
ZT4
ZT24
ZT20
ZT16
ZT8
Dn
OGAh
Dn
OGAh
Dn
OGAh
Dn
OGAh
Dn
OGAh
Dn
OGAh
CON
CON
CON
CON
CON
CON
kDa
PER2
250
150
Amido
Black
150
75
OEG = Other Experimental Group
Membranes cut into 5 sections, allowing interrogation of multiple distinct protein targets (only 1 target reported here)
Click on image, then ‘crop’ tool, to see the excess non-membrane area

## Slide 57
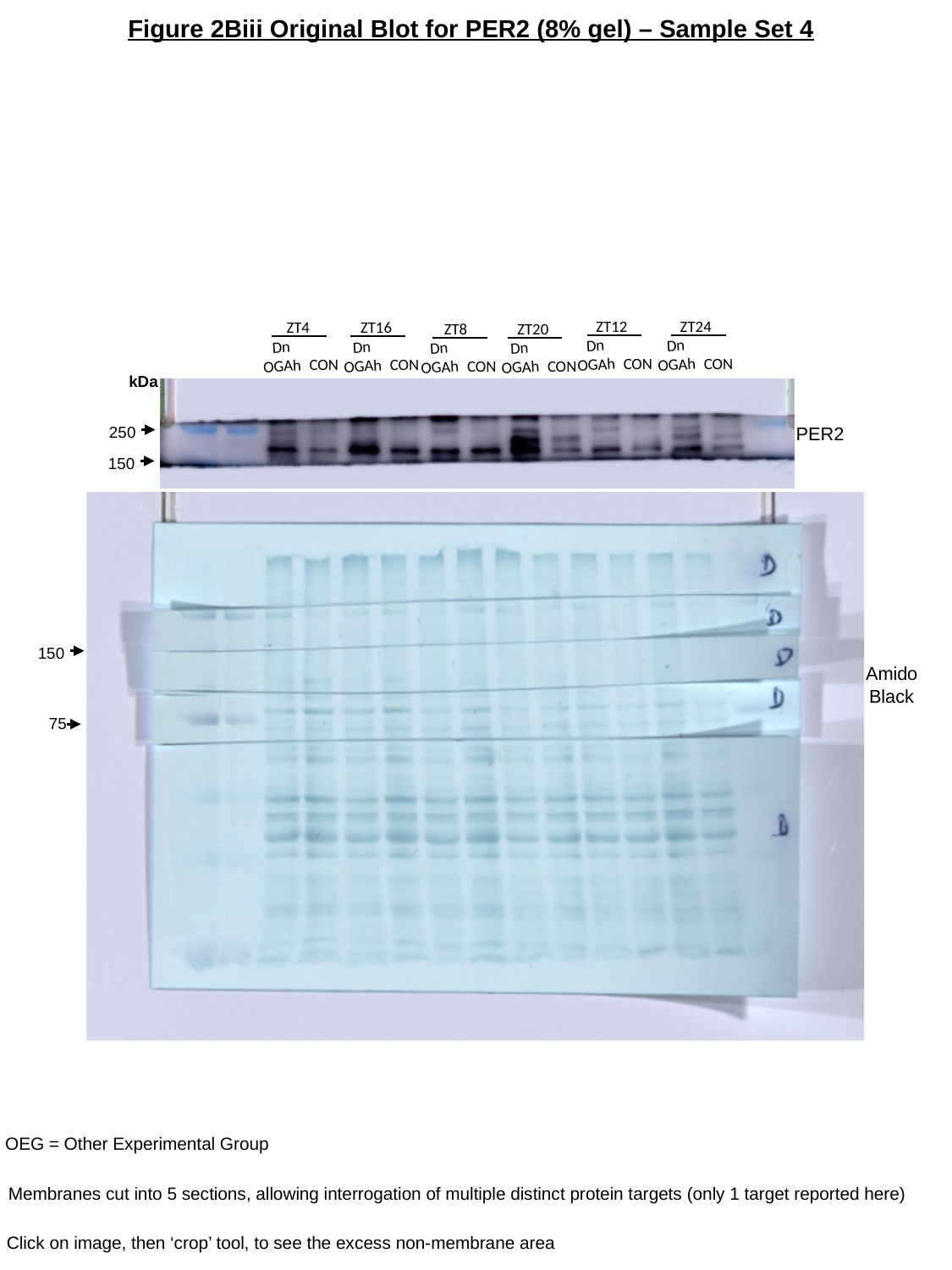

Figure 2Biii Original Blot for PER2 (8% gel) – Sample Set 4
ZT12
ZT24
ZT4
ZT16
ZT8
ZT20
Dn
OGAh
Dn
OGAh
Dn
OGAh
Dn
OGAh
Dn
OGAh
Dn
OGAh
CON
CON
CON
CON
CON
CON
kDa
250
PER2
150
150
Amido
Black
75
OEG = Other Experimental Group
Membranes cut into 5 sections, allowing interrogation of multiple distinct protein targets (only 1 target reported here)
Click on image, then ‘crop’ tool, to see the excess non-membrane area

## Slide 58
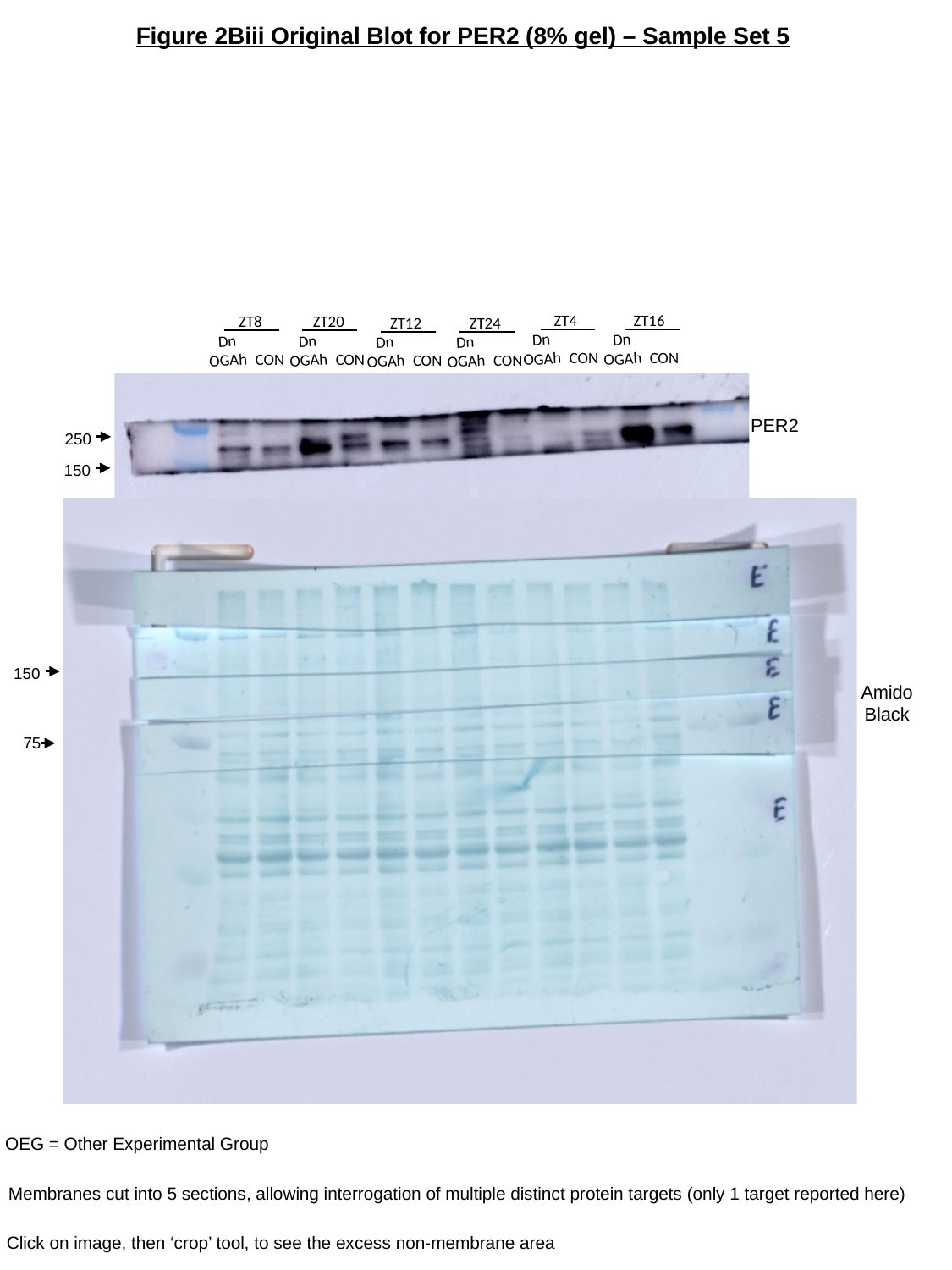

Figure 2Biii Original Blot for PER2 (8% gel) – Sample Set 5
ZT4
ZT16
ZT8
ZT20
ZT12
ZT24
Dn
OGAh
Dn
OGAh
Dn
OGAh
Dn
OGAh
Dn
OGAh
Dn
OGAh
CON
CON
CON
CON
CON
CON
PER2
250
150
150
Amido
Black
75
OEG = Other Experimental Group
Membranes cut into 5 sections, allowing interrogation of multiple distinct protein targets (only 1 target reported here)
Click on image, then ‘crop’ tool, to see the excess non-membrane area

## Slide 59
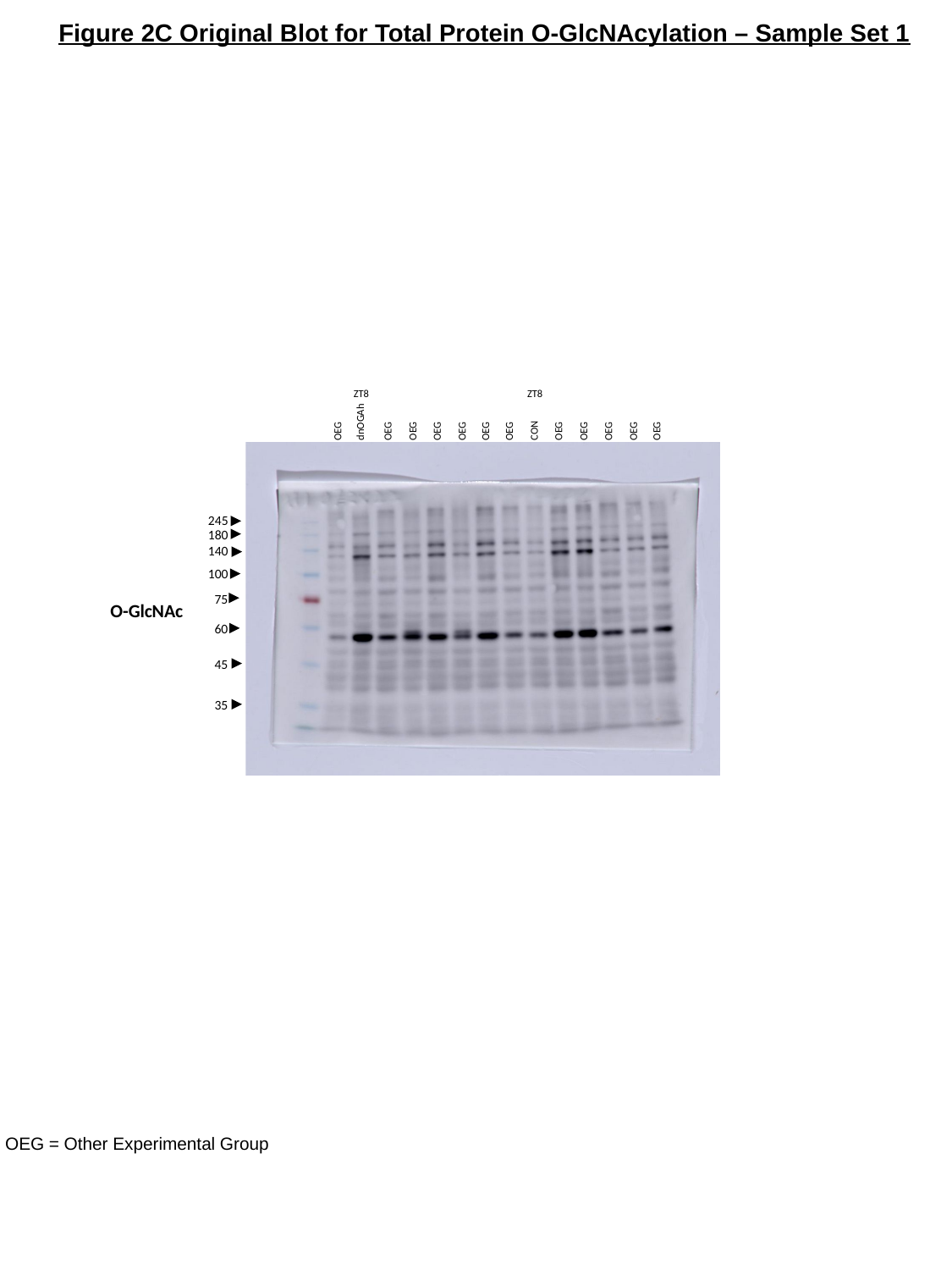

Figure 2C Original Blot for Total Protein O-GlcNAcylation – Sample Set 1
ZT8
ZT8
dnOGAh
CON
OEG
OEG
OEG
OEG
OEG
OEG
OEG
OEG
OEG
OEG
OEG
OEG
245
180
140
100
75
O-GlcNAc
60
45
35
OEG = Other Experimental Group

## Slide 60
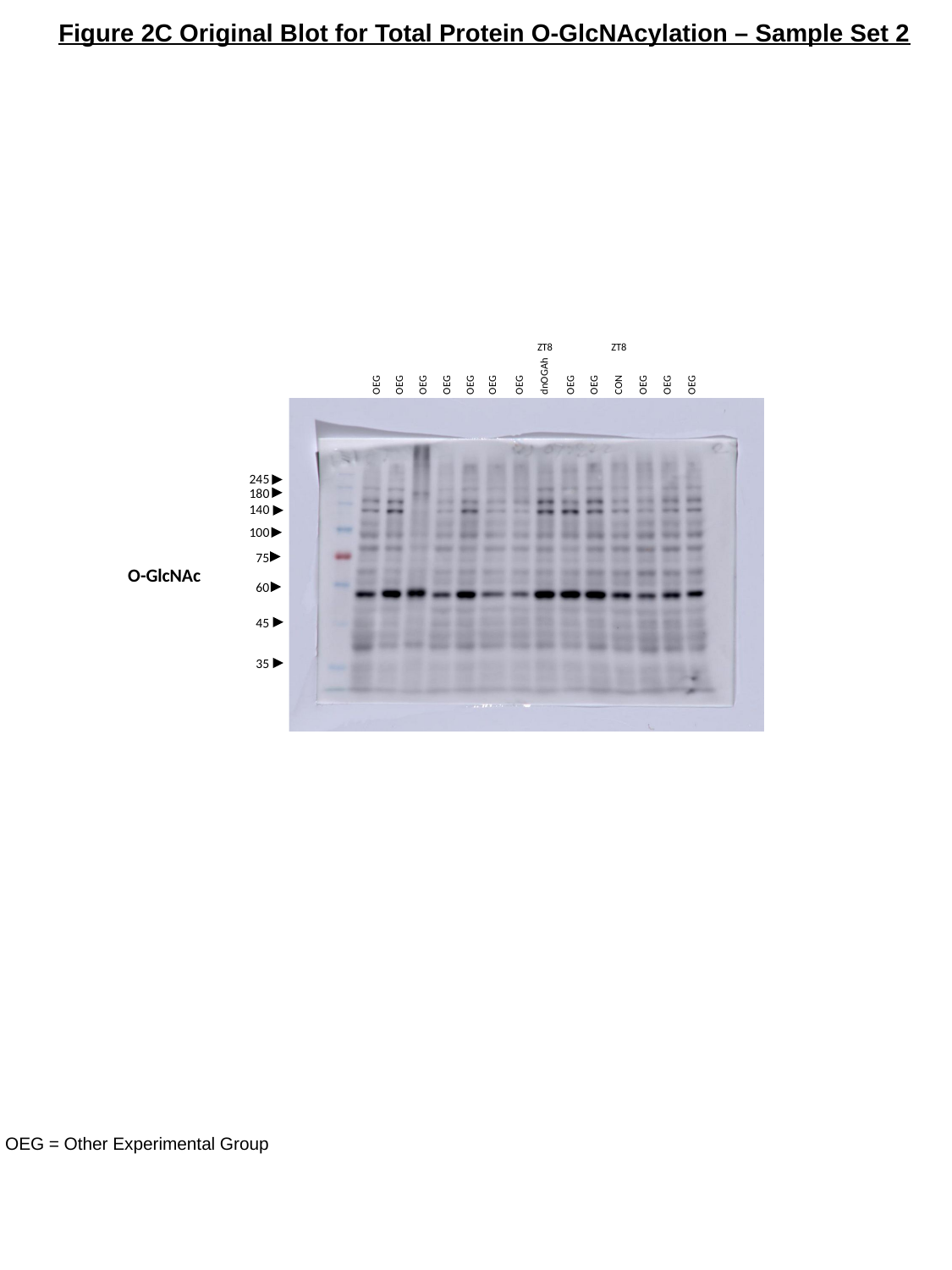

Figure 2C Original Blot for Total Protein O-GlcNAcylation – Sample Set 2
ZT8
ZT8
dnOGAh
CON
OEG
OEG
OEG
OEG
OEG
OEG
OEG
OEG
OEG
OEG
OEG
OEG
245
180
140
100
75
O-GlcNAc
60
45
35
OEG = Other Experimental Group

## Slide 61
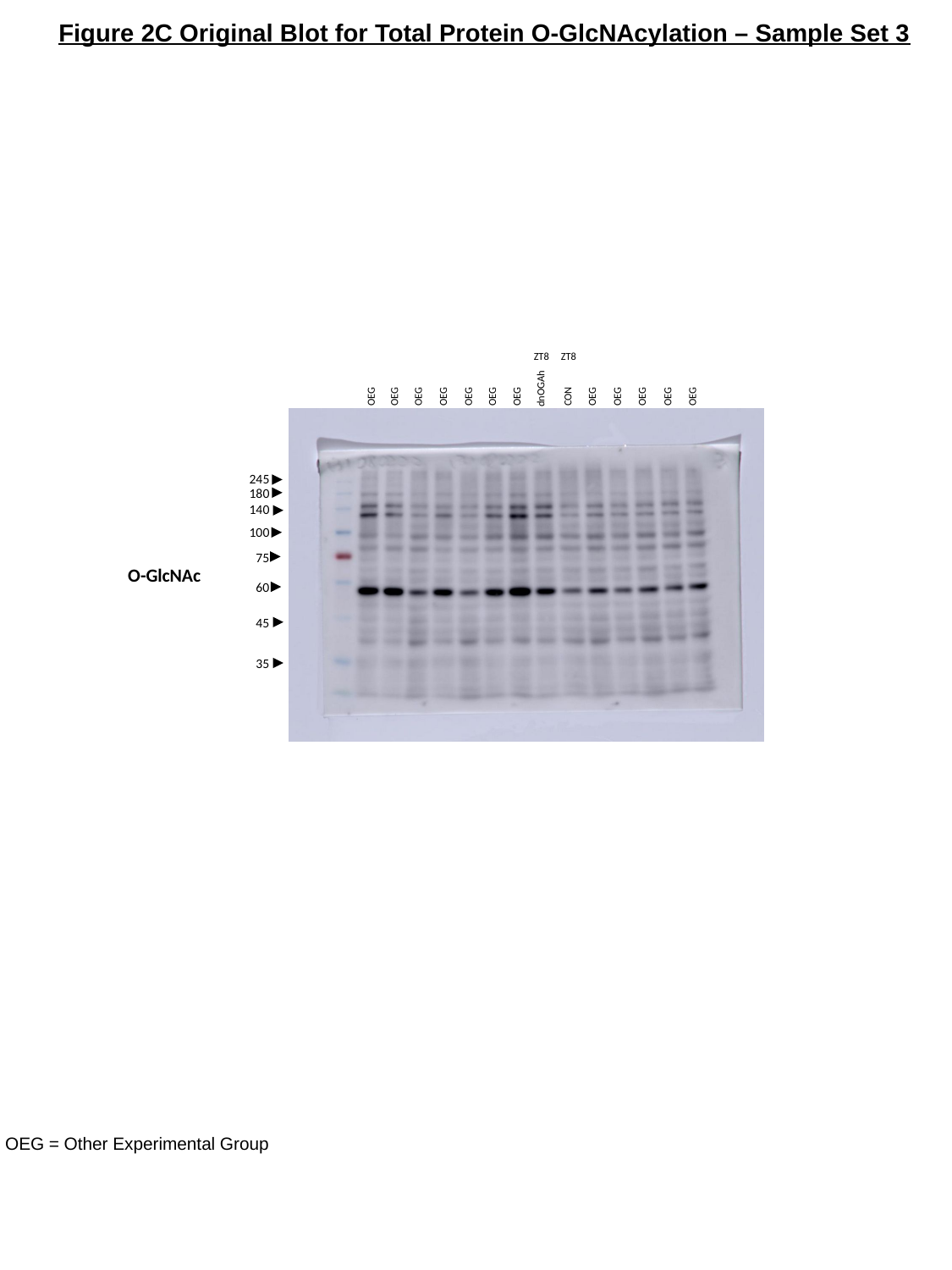

Figure 2C Original Blot for Total Protein O-GlcNAcylation – Sample Set 3
ZT8
ZT8
dnOGAh
CON
OEG
OEG
OEG
OEG
OEG
OEG
OEG
OEG
OEG
OEG
OEG
OEG
245
180
140
100
75
O-GlcNAc
60
45
35
OEG = Other Experimental Group

## Slide 62
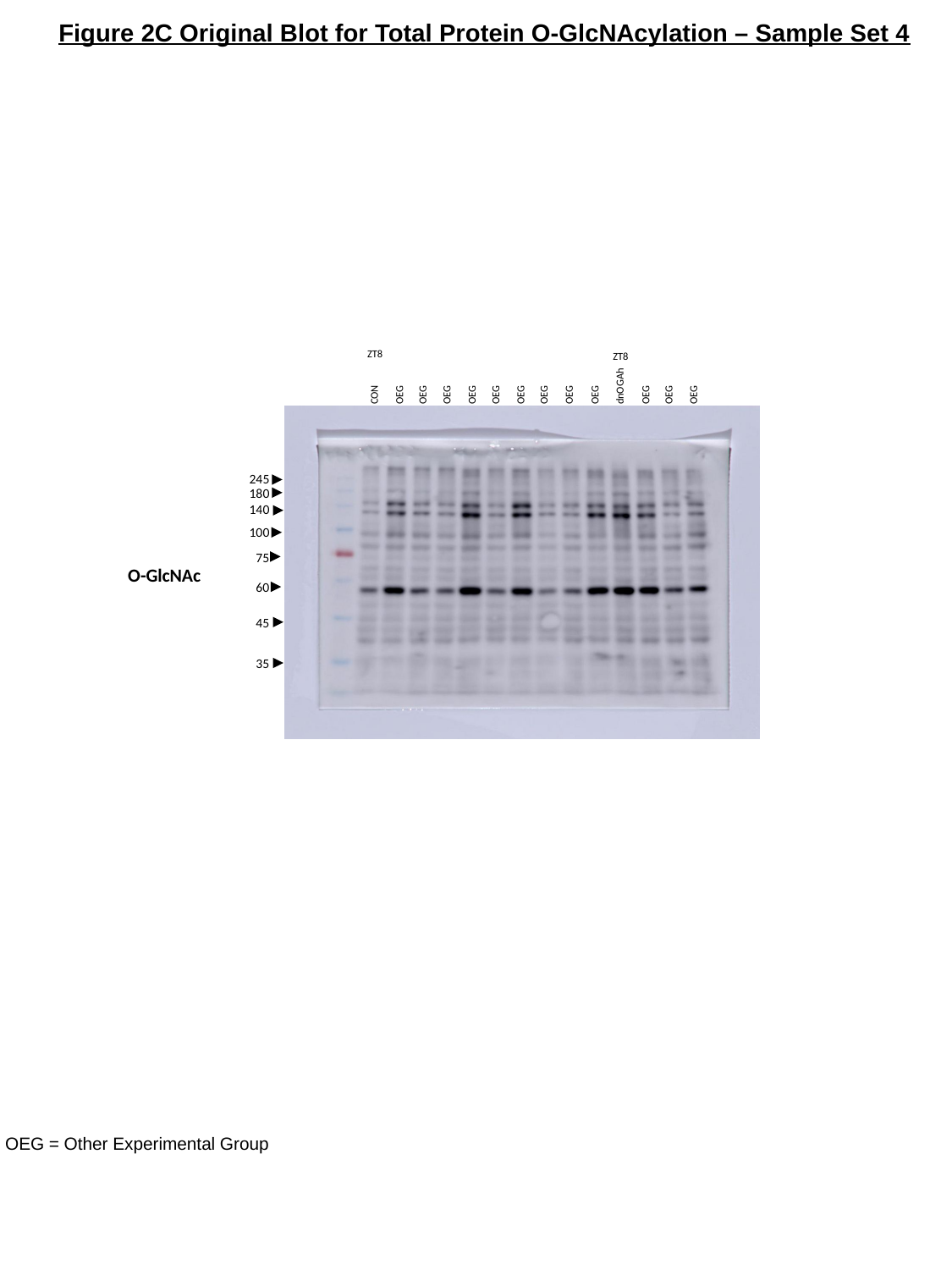

Figure 2C Original Blot for Total Protein O-GlcNAcylation – Sample Set 4
ZT8
ZT8
dnOGAh
CON
OEG
OEG
OEG
OEG
OEG
OEG
OEG
OEG
OEG
OEG
OEG
OEG
245
180
140
100
75
O-GlcNAc
60
45
35
OEG = Other Experimental Group

## Slide 63
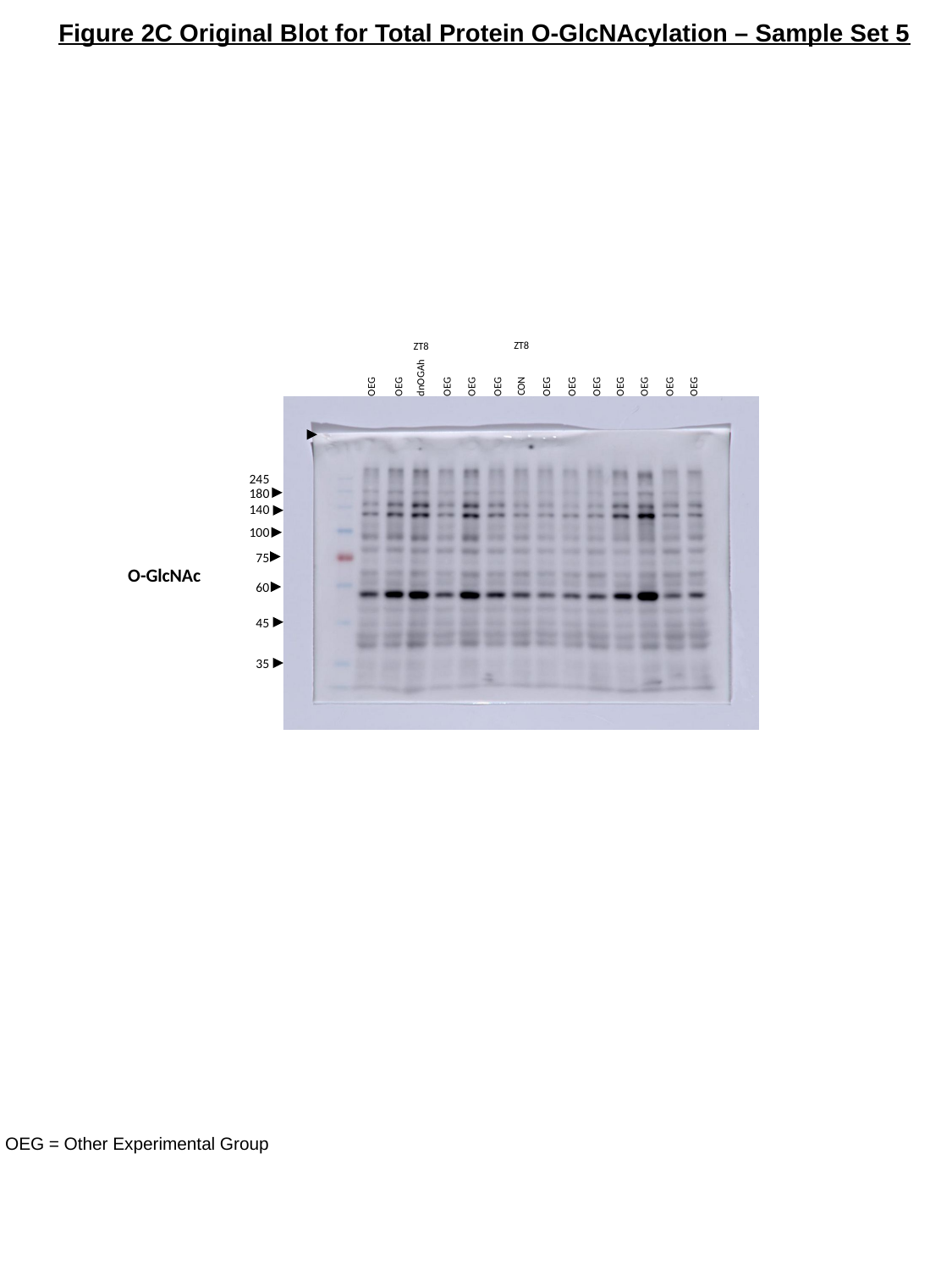

Figure 2C Original Blot for Total Protein O-GlcNAcylation – Sample Set 5
ZT8
ZT8
dnOGAh
CON
OEG
OEG
OEG
OEG
OEG
OEG
OEG
OEG
OEG
OEG
OEG
OEG
245
180
140
100
75
O-GlcNAc
60
45
35
OEG = Other Experimental Group

## Slide 64
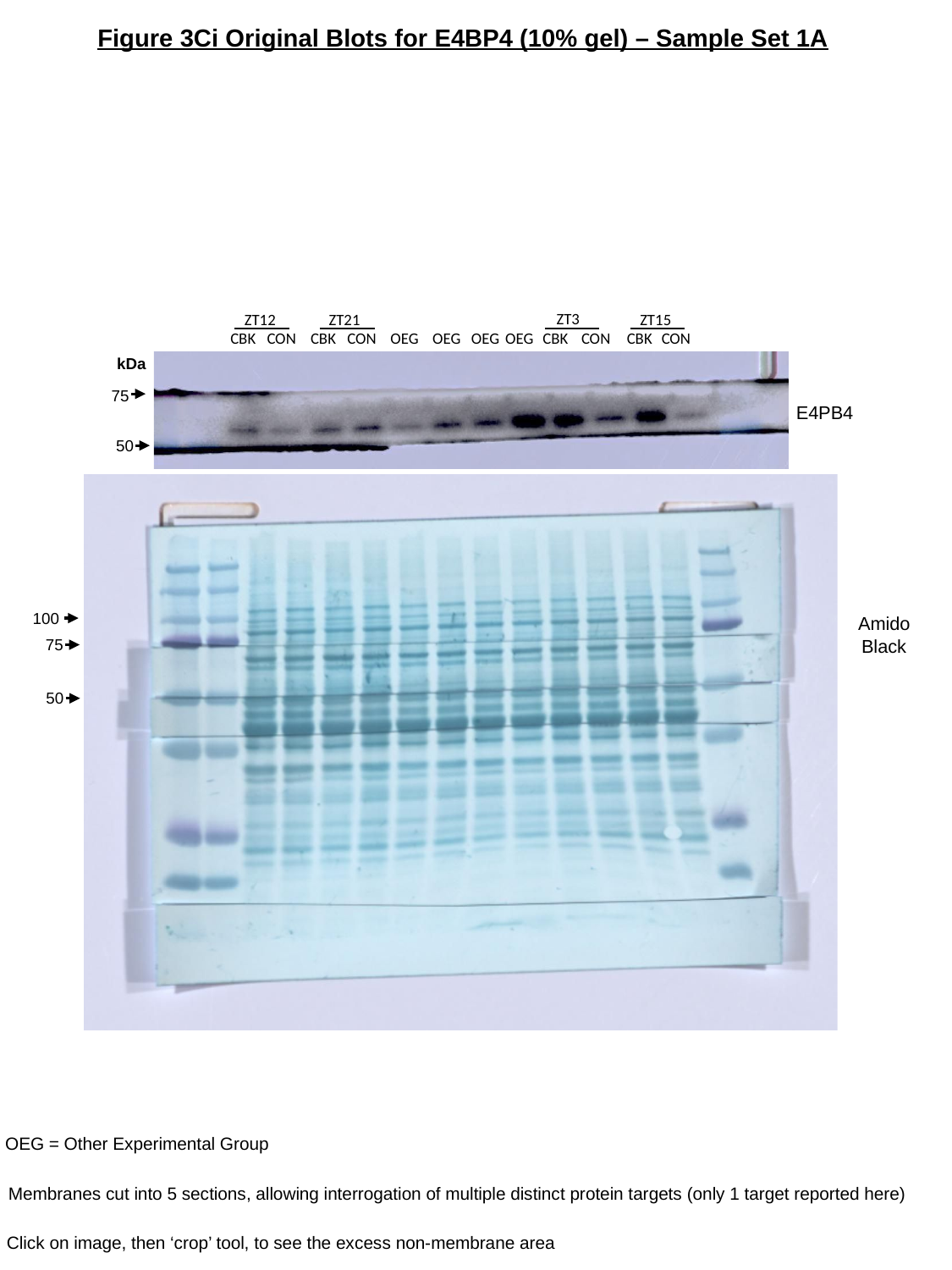

Figure 3Ci Original Blots for E4BP4 (10% gel) – Sample Set 1A
ZT3
ZT12
ZT21
ZT15
CBK
CON
CBK
CON
OEG
OEG
OEG
OEG
CBK
CON
CBK
CON
kDa
75
E4PB4
50
100
Amido
Black
75
50
OEG = Other Experimental Group
Membranes cut into 5 sections, allowing interrogation of multiple distinct protein targets (only 1 target reported here)
Click on image, then ‘crop’ tool, to see the excess non-membrane area

## Slide 65
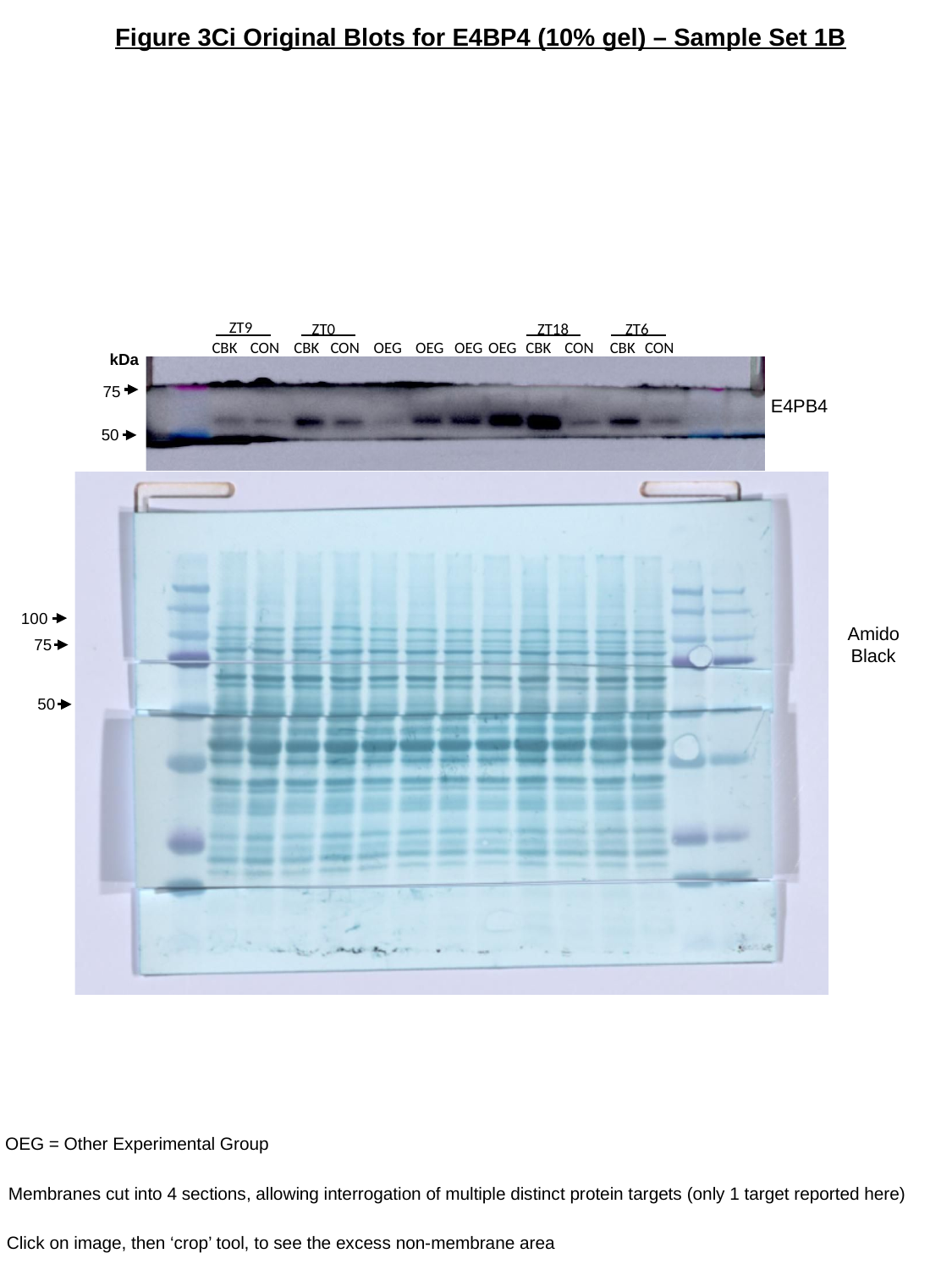

Figure 3Ci Original Blots for E4BP4 (10% gel) – Sample Set 1B
ZT9
ZT0
ZT18
ZT6
CBK
CON
CBK
CON
OEG
OEG
OEG
OEG
CBK
CON
CBK
CON
kDa
75
E4PB4
50
100
Amido
Black
75
50
OEG = Other Experimental Group
Membranes cut into 4 sections, allowing interrogation of multiple distinct protein targets (only 1 target reported here)
Click on image, then ‘crop’ tool, to see the excess non-membrane area

## Slide 66
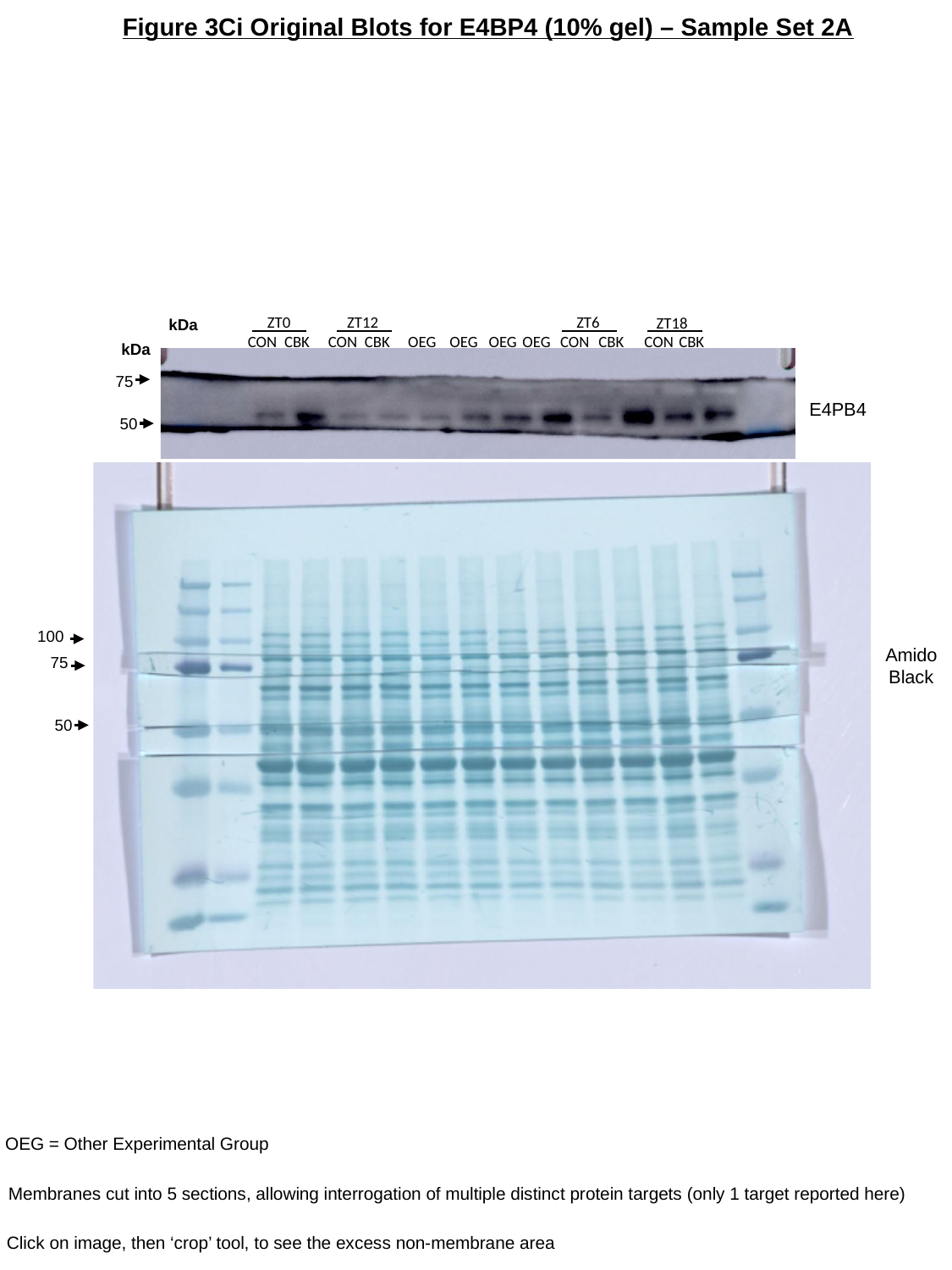

Figure 3Ci Original Blots for E4BP4 (10% gel) – Sample Set 2A
kDa
ZT6
ZT0
ZT12
ZT18
CON
CBK
CON
CBK
OEG
OEG
OEG
OEG
CON
CBK
CON
CBK
kDa
75
E4PB4
50
100
Amido
Black
75
50
OEG = Other Experimental Group
Membranes cut into 5 sections, allowing interrogation of multiple distinct protein targets (only 1 target reported here)
Click on image, then ‘crop’ tool, to see the excess non-membrane area

## Slide 67
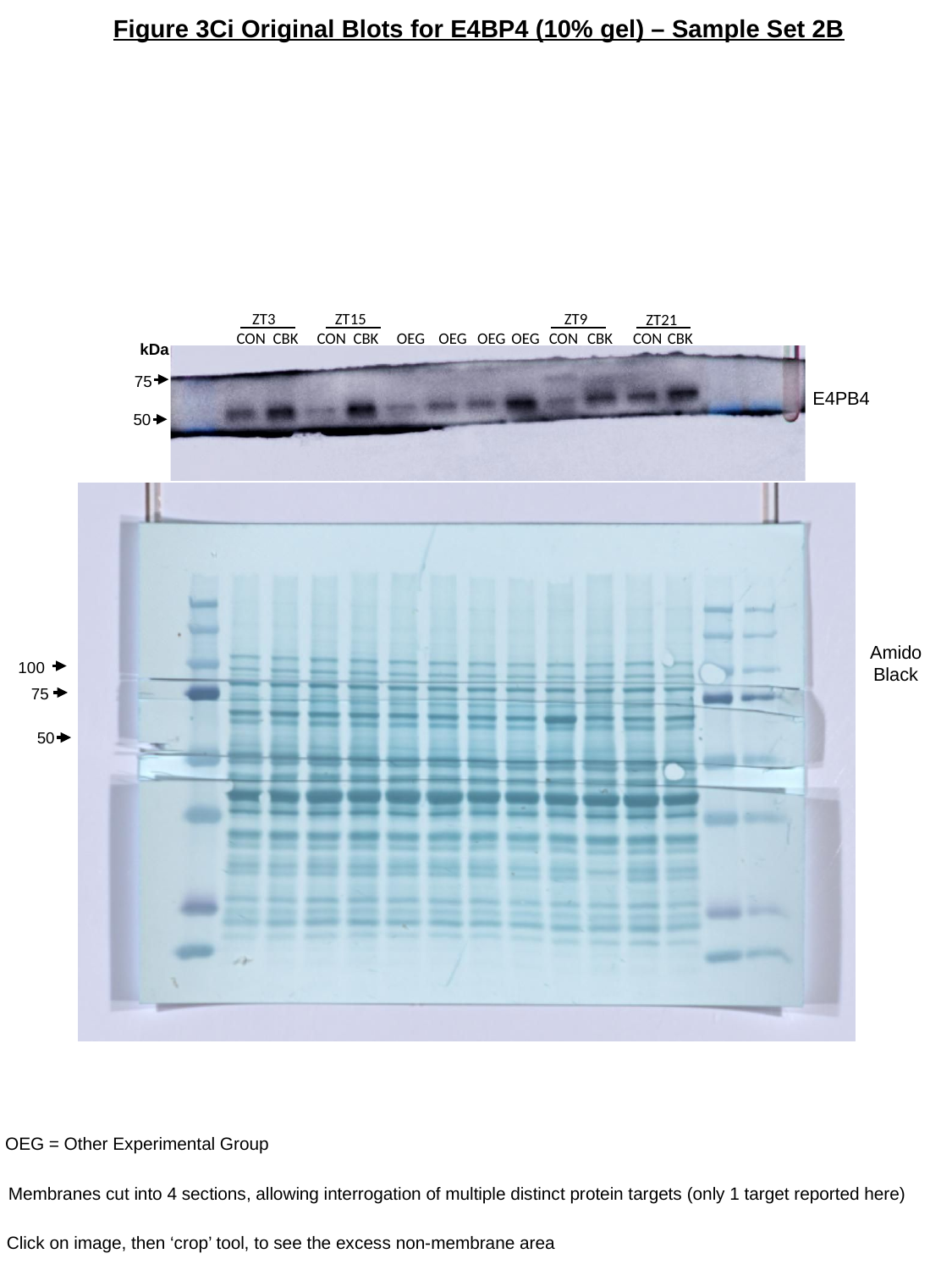

Figure 3Ci Original Blots for E4BP4 (10% gel) – Sample Set 2B
ZT9
ZT3
ZT15
ZT21
CON
CBK
CON
CBK
OEG
OEG
OEG
OEG
CON
CBK
CON
CBK
kDa
75
E4PB4
50
Amido
Black
100
75
50
OEG = Other Experimental Group
Membranes cut into 4 sections, allowing interrogation of multiple distinct protein targets (only 1 target reported here)
Click on image, then ‘crop’ tool, to see the excess non-membrane area

## Slide 68
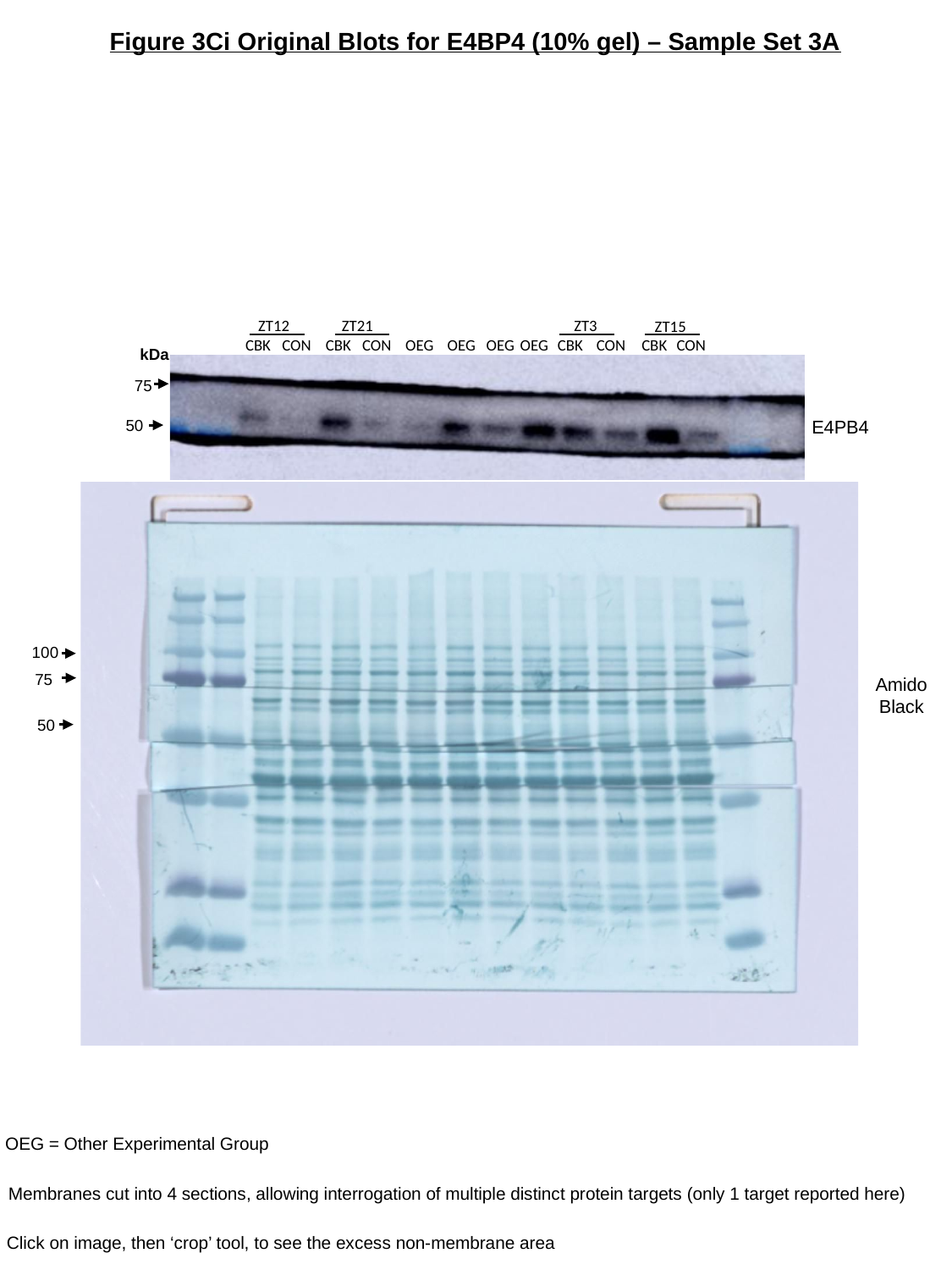

Figure 3Ci Original Blots for E4BP4 (10% gel) – Sample Set 3A
ZT3
ZT12
ZT21
ZT15
CBK
CON
CBK
CON
OEG
OEG
OEG
OEG
CBK
CON
CBK
CON
kDa
75
E4PB4
50
100
75
Amido
Black
50
OEG = Other Experimental Group
Membranes cut into 4 sections, allowing interrogation of multiple distinct protein targets (only 1 target reported here)
Click on image, then ‘crop’ tool, to see the excess non-membrane area

## Slide 69
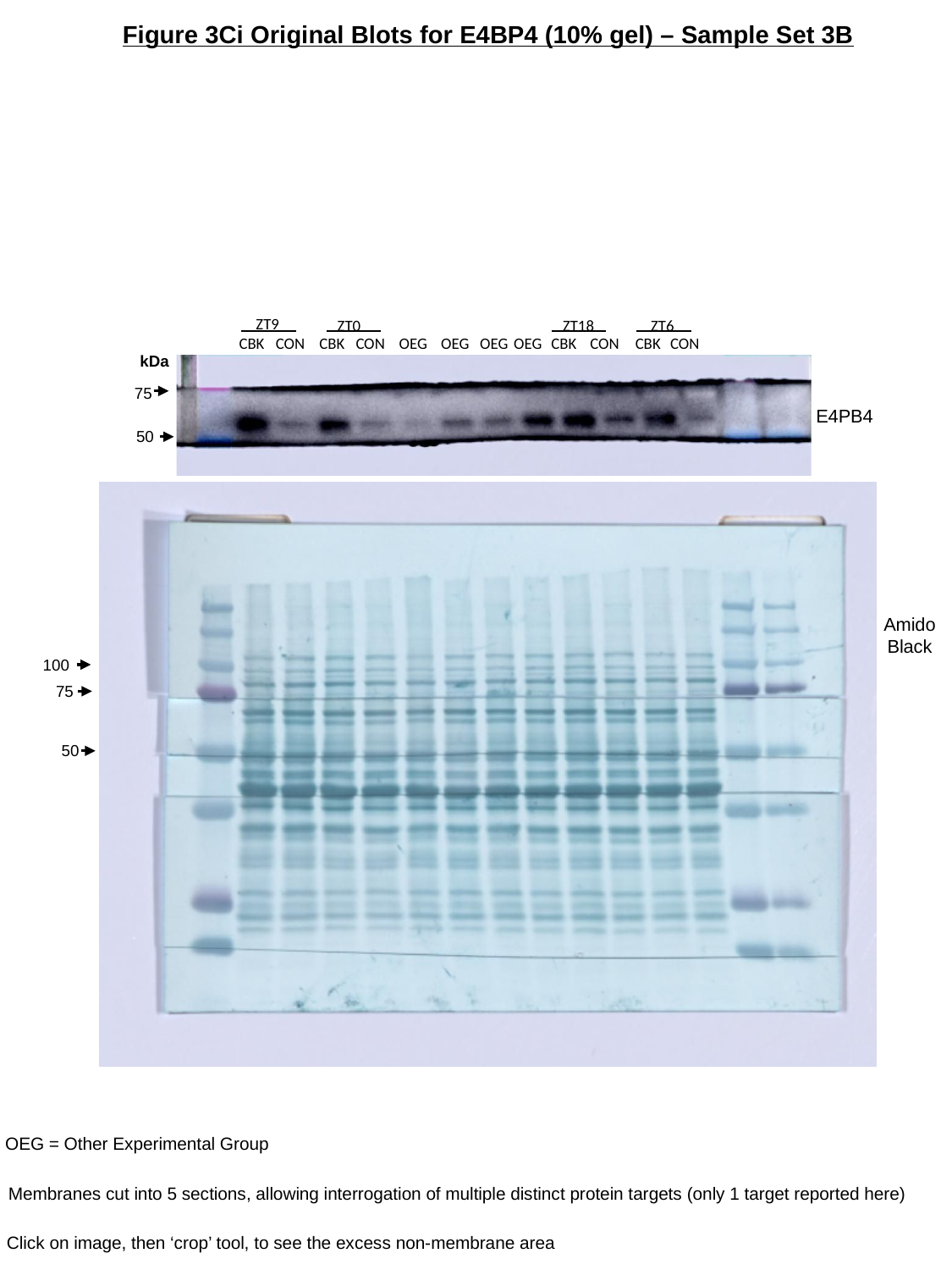

Figure 3Ci Original Blots for E4BP4 (10% gel) – Sample Set 3B
ZT9
ZT0
ZT18
ZT6
CBK
CON
CBK
CON
OEG
OEG
OEG
OEG
CBK
CON
CBK
CON
kDa
75
E4PB4
50
Amido
Black
100
75
50
OEG = Other Experimental Group
Membranes cut into 5 sections, allowing interrogation of multiple distinct protein targets (only 1 target reported here)
Click on image, then ‘crop’ tool, to see the excess non-membrane area

## Slide 70
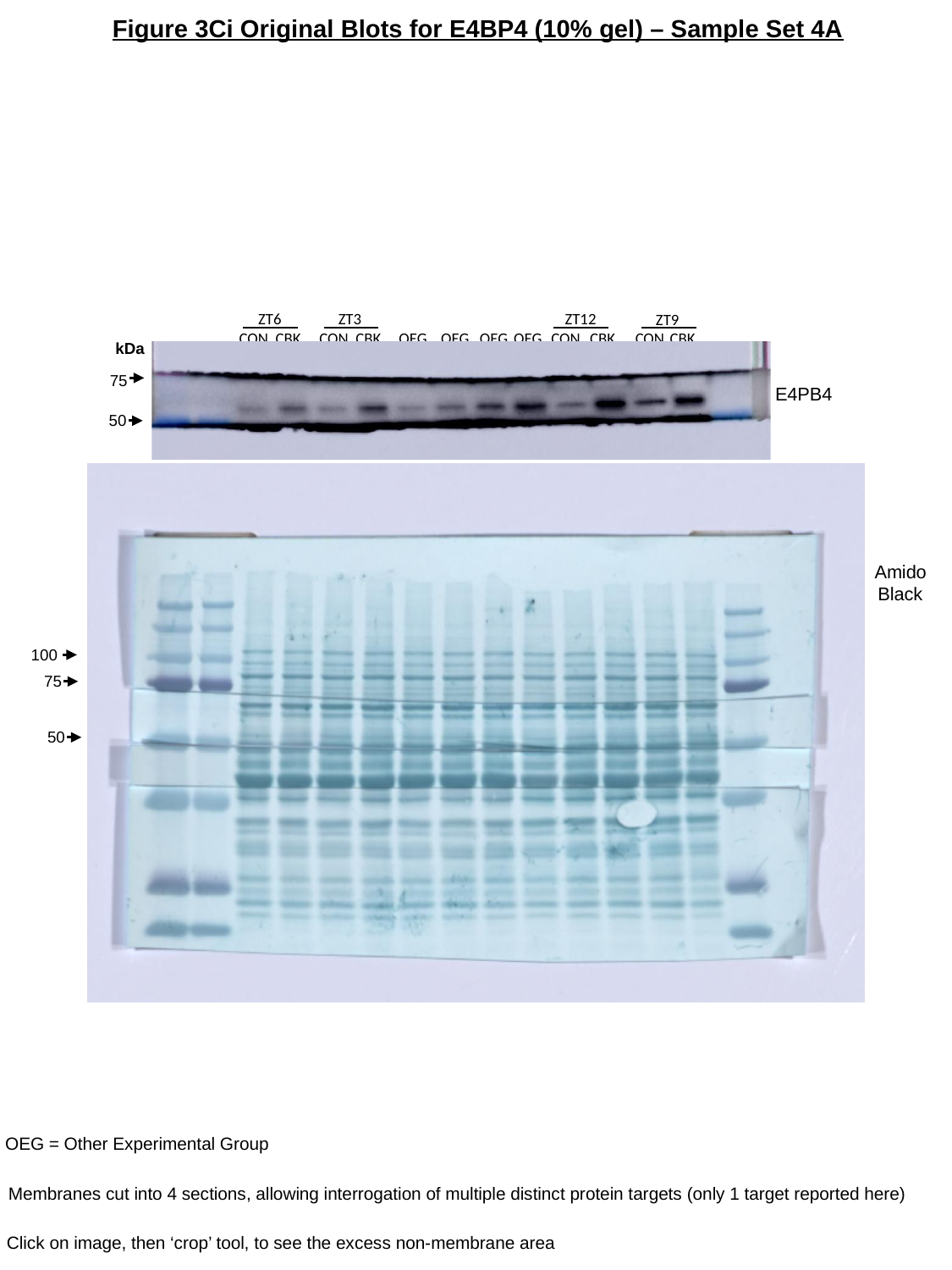

Figure 3Ci Original Blots for E4BP4 (10% gel) – Sample Set 4A
ZT12
ZT6
ZT3
ZT9
CON
CBK
CON
CBK
OEG
OEG
OEG
OEG
CON
CBK
CON
CBK
kDa
75
E4PB4
50
Amido
Black
100
75
50
OEG = Other Experimental Group
Membranes cut into 4 sections, allowing interrogation of multiple distinct protein targets (only 1 target reported here)
Click on image, then ‘crop’ tool, to see the excess non-membrane area

## Slide 71
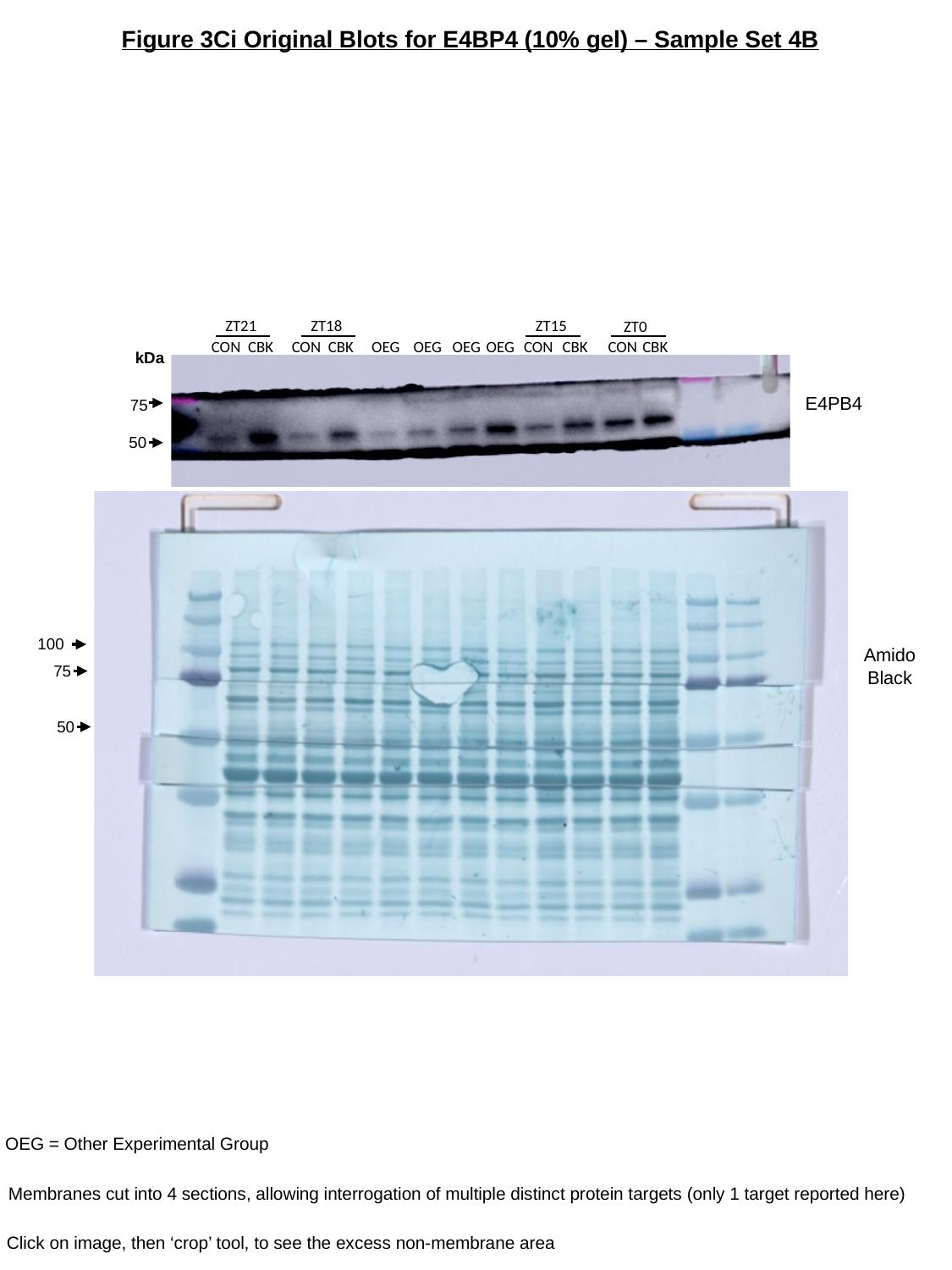

Figure 3Ci Original Blots for E4BP4 (10% gel) – Sample Set 4B
ZT15
ZT21
ZT18
ZT0
CON
CBK
CON
CBK
OEG
OEG
OEG
OEG
CON
CBK
CON
CBK
kDa
E4PB4
75
50
100
Amido
Black
75
50
OEG = Other Experimental Group
Membranes cut into 4 sections, allowing interrogation of multiple distinct protein targets (only 1 target reported here)
Click on image, then ‘crop’ tool, to see the excess non-membrane area

## Slide 72
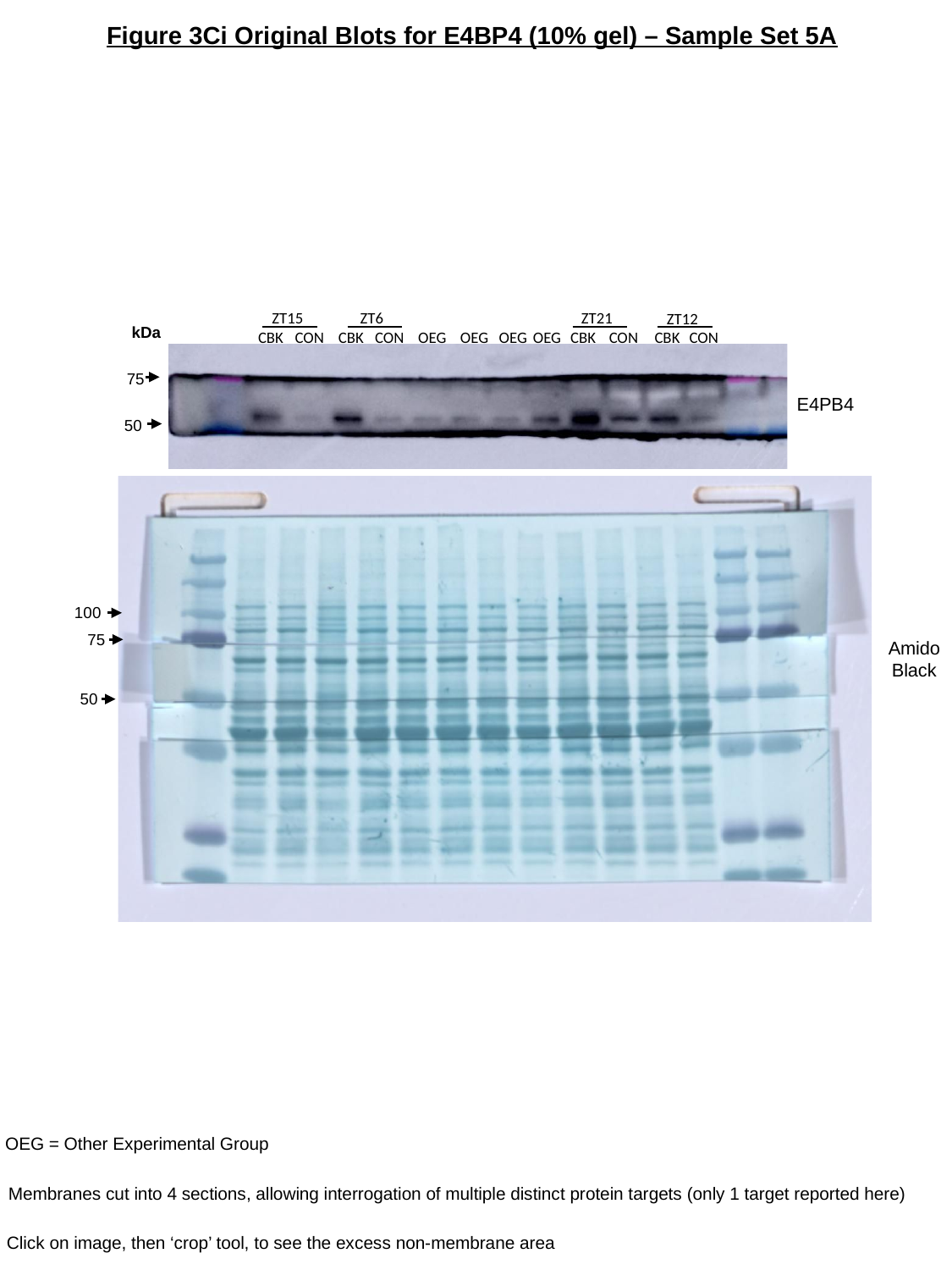

Figure 3Ci Original Blots for E4BP4 (10% gel) – Sample Set 5A
ZT21
ZT15
ZT6
ZT12
kDa
CBK
CON
CBK
CON
OEG
OEG
OEG
OEG
CBK
CON
CBK
CON
75
E4PB4
50
100
75
Amido
Black
50
OEG = Other Experimental Group
Membranes cut into 4 sections, allowing interrogation of multiple distinct protein targets (only 1 target reported here)
Click on image, then ‘crop’ tool, to see the excess non-membrane area

## Slide 73
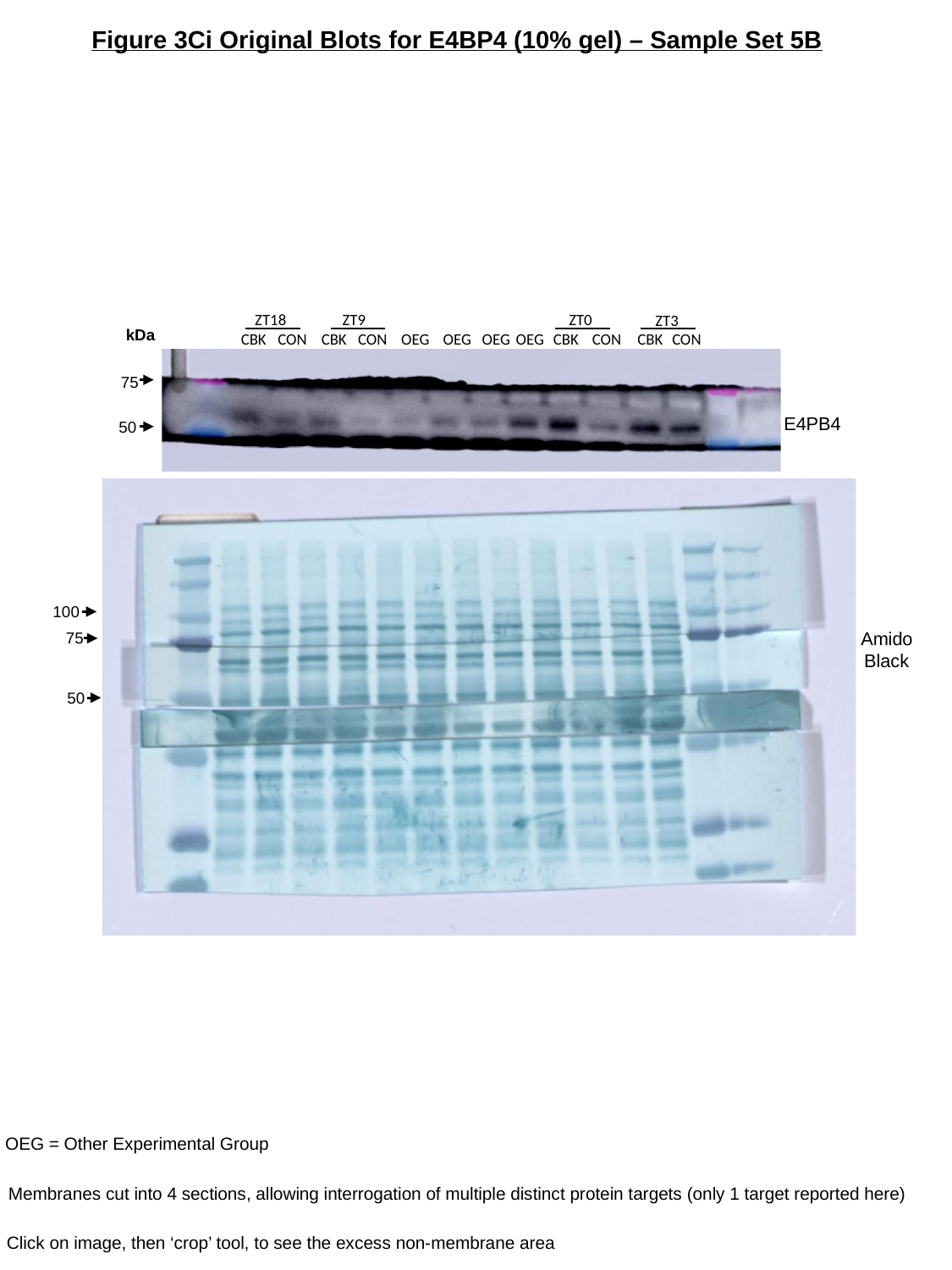

Figure 3Ci Original Blots for E4BP4 (10% gel) – Sample Set 5B
ZT0
ZT18
ZT9
ZT3
kDa
CBK
CON
CBK
CON
OEG
OEG
OEG
OEG
CBK
CON
CBK
CON
75
E4PB4
50
100
Amido
Black
75
50
OEG = Other Experimental Group
Membranes cut into 4 sections, allowing interrogation of multiple distinct protein targets (only 1 target reported here)
Click on image, then ‘crop’ tool, to see the excess non-membrane area

## Slide 74
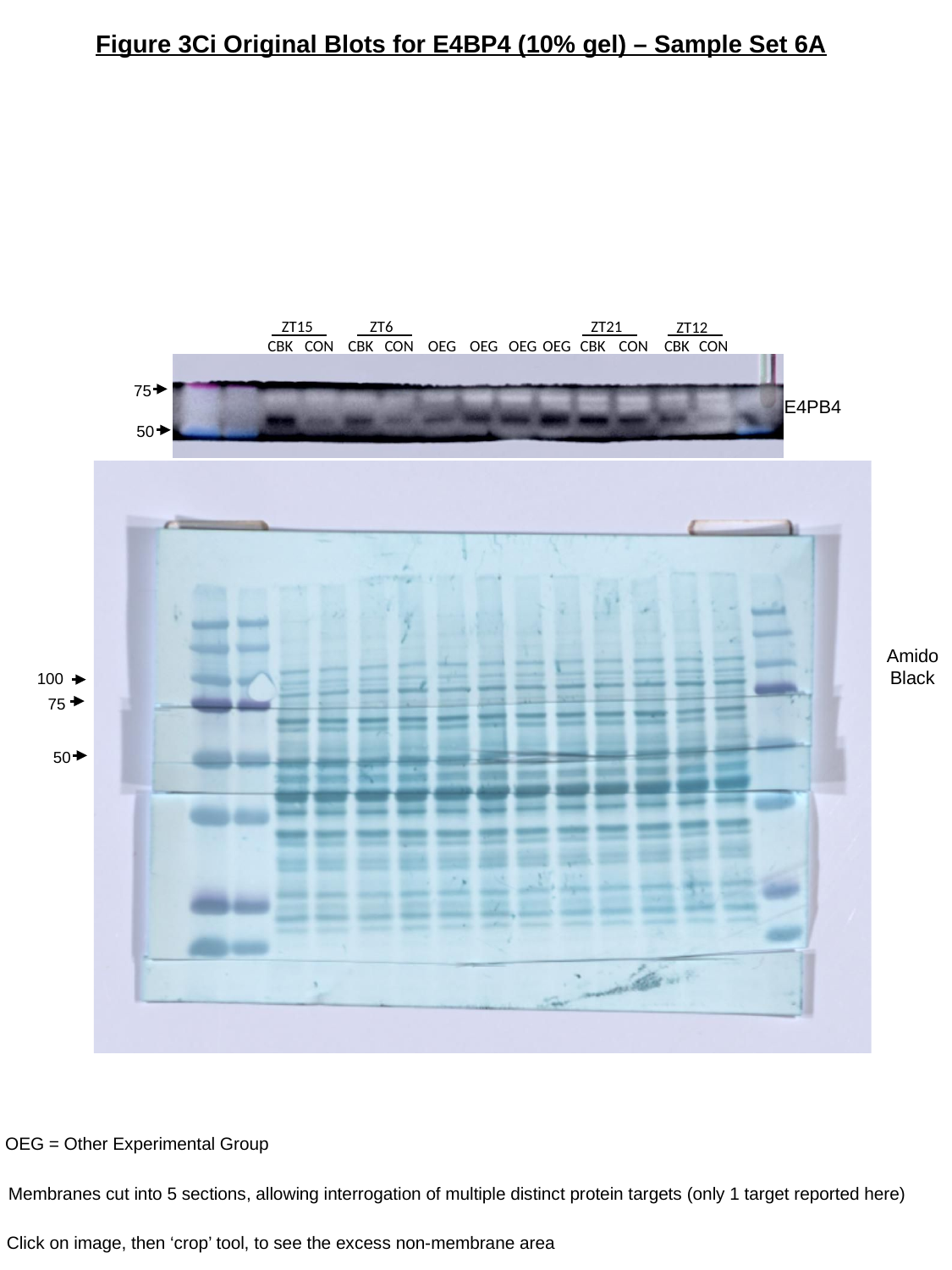

Figure 3Ci Original Blots for E4BP4 (10% gel) – Sample Set 6A
ZT21
ZT15
ZT6
ZT12
CBK
CON
CBK
CON
OEG
OEG
OEG
OEG
CBK
CON
CBK
CON
75
E4PB4
50
Amido
Black
100
75
50
OEG = Other Experimental Group
Membranes cut into 5 sections, allowing interrogation of multiple distinct protein targets (only 1 target reported here)
Click on image, then ‘crop’ tool, to see the excess non-membrane area

## Slide 75
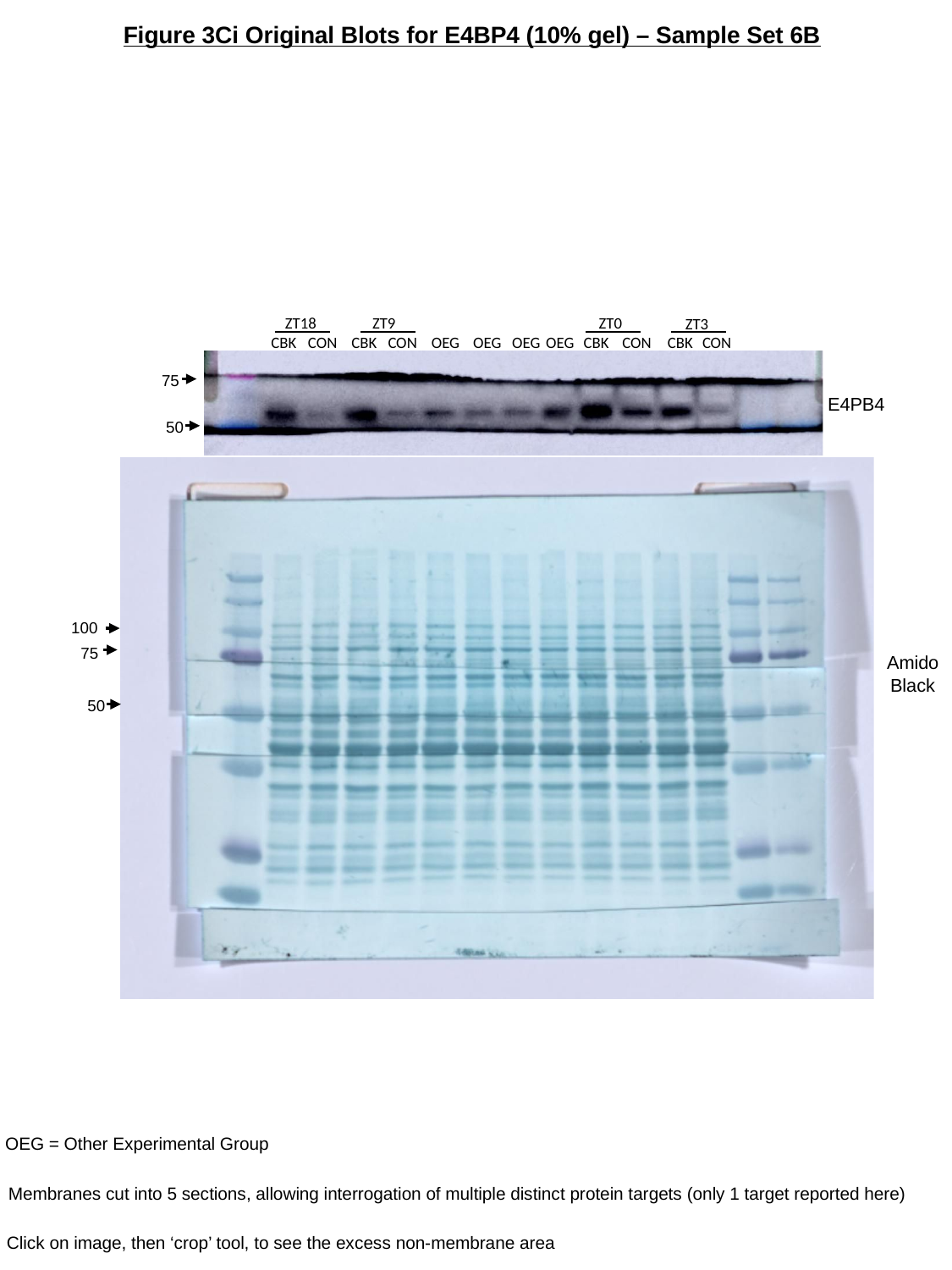

Figure 3Ci Original Blots for E4BP4 (10% gel) – Sample Set 6B
ZT0
ZT18
ZT9
ZT3
CBK
CON
CBK
CON
OEG
OEG
OEG
OEG
CBK
CON
CBK
CON
75
E4PB4
50
100
75
Amido
Black
50
OEG = Other Experimental Group
Membranes cut into 5 sections, allowing interrogation of multiple distinct protein targets (only 1 target reported here)
Click on image, then ‘crop’ tool, to see the excess non-membrane area

## Slide 76
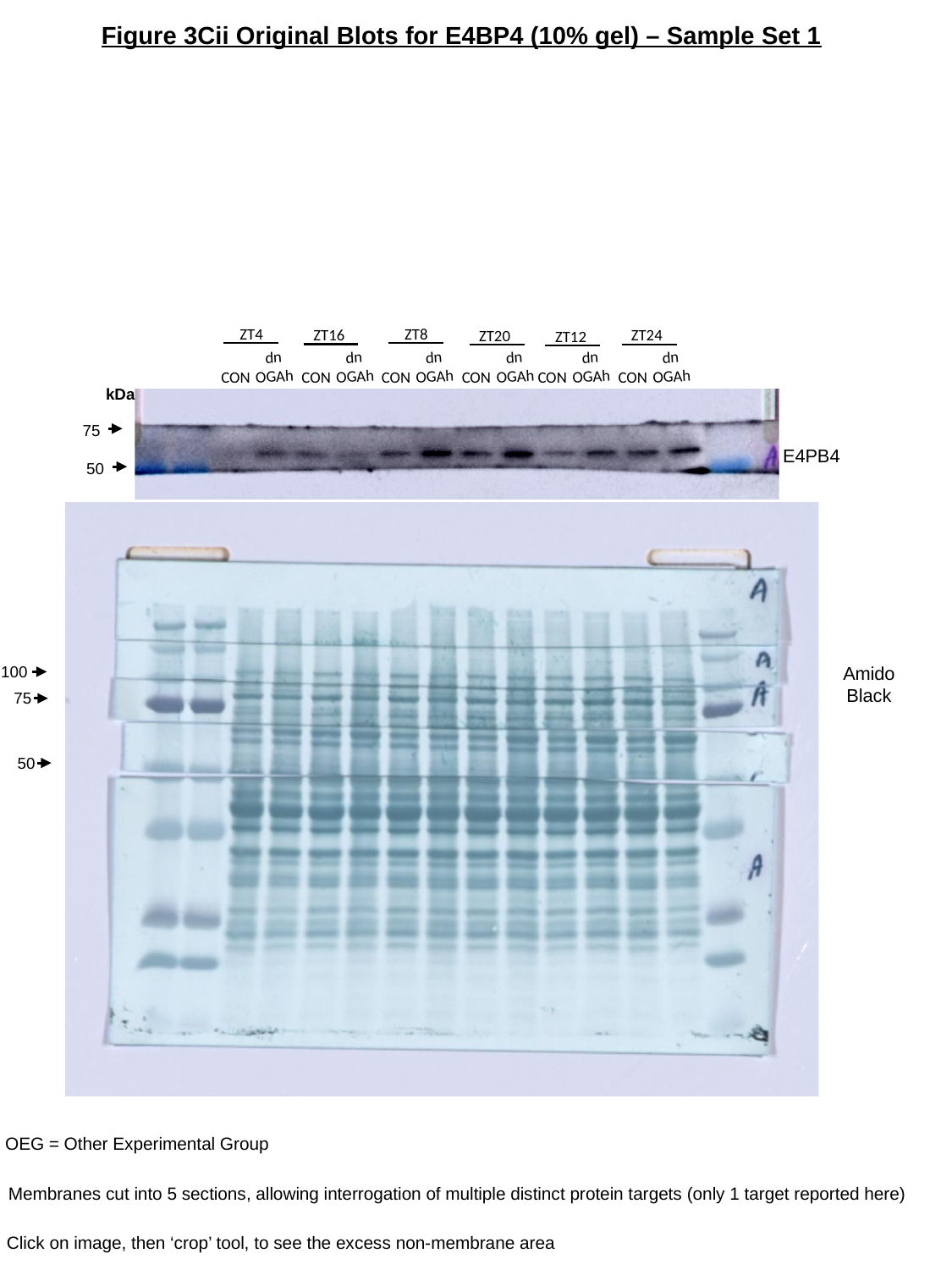

Figure 3Cii Original Blots for E4BP4 (10% gel) – Sample Set 1
ZT4
ZT8
ZT16
ZT24
ZT20
ZT12
dn
OGAh
dn
OGAh
dn
OGAh
dn
OGAh
dn
OGAh
dn
OGAh
CON
CON
CON
CON
CON
CON
kDa
75
E4PB4
50
100
Amido
Black
75
50
OEG = Other Experimental Group
Membranes cut into 5 sections, allowing interrogation of multiple distinct protein targets (only 1 target reported here)
Click on image, then ‘crop’ tool, to see the excess non-membrane area

## Slide 77
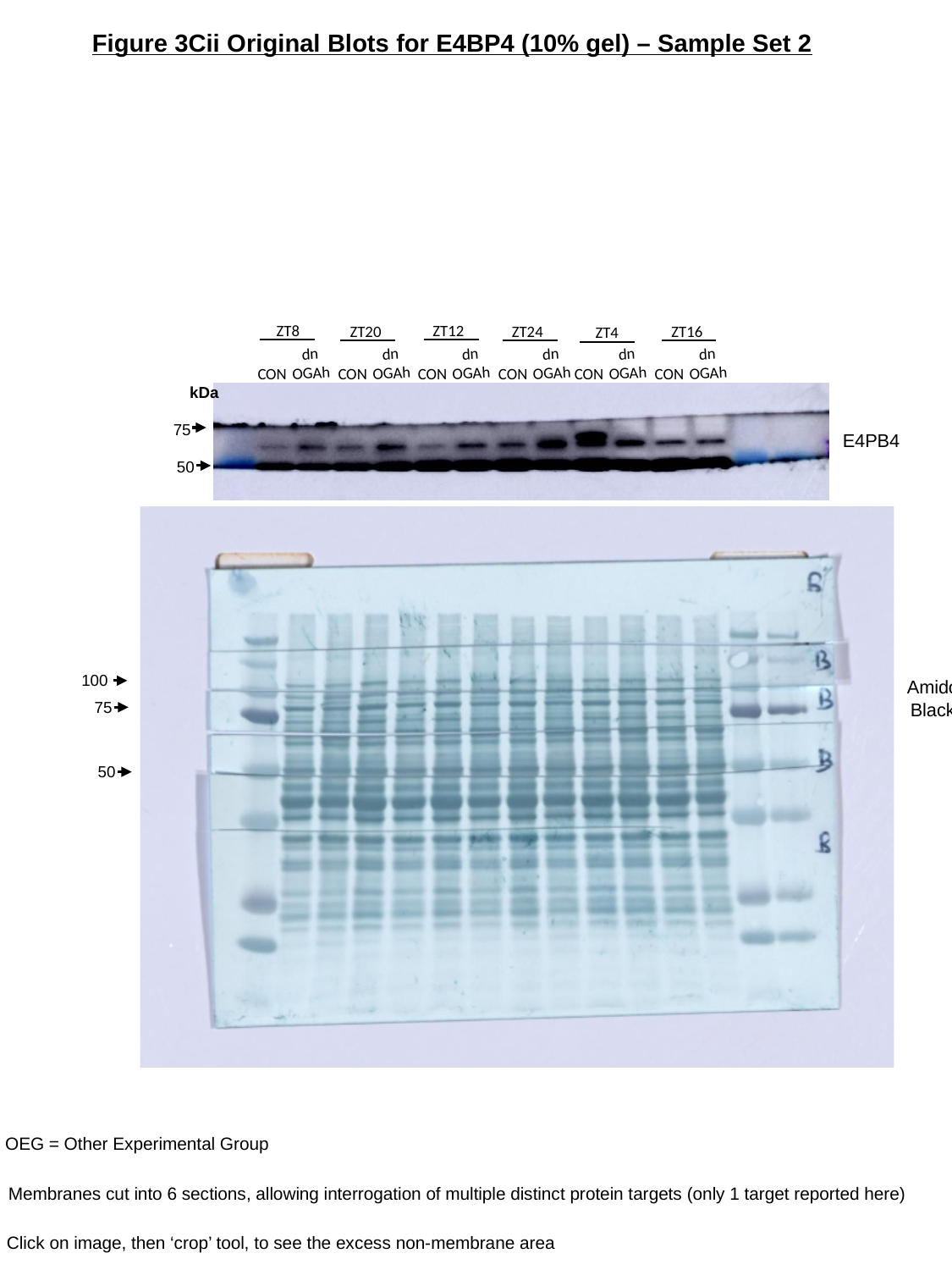

Figure 3Cii Original Blots for E4BP4 (10% gel) – Sample Set 2
ZT8
ZT12
ZT20
ZT16
ZT24
ZT4
dn
OGAh
dn
OGAh
dn
OGAh
dn
OGAh
dn
OGAh
dn
OGAh
CON
CON
CON
CON
CON
CON
kDa
75
E4PB4
50
100
Amido
Black
75
50
OEG = Other Experimental Group
Membranes cut into 6 sections, allowing interrogation of multiple distinct protein targets (only 1 target reported here)
Click on image, then ‘crop’ tool, to see the excess non-membrane area

## Slide 78
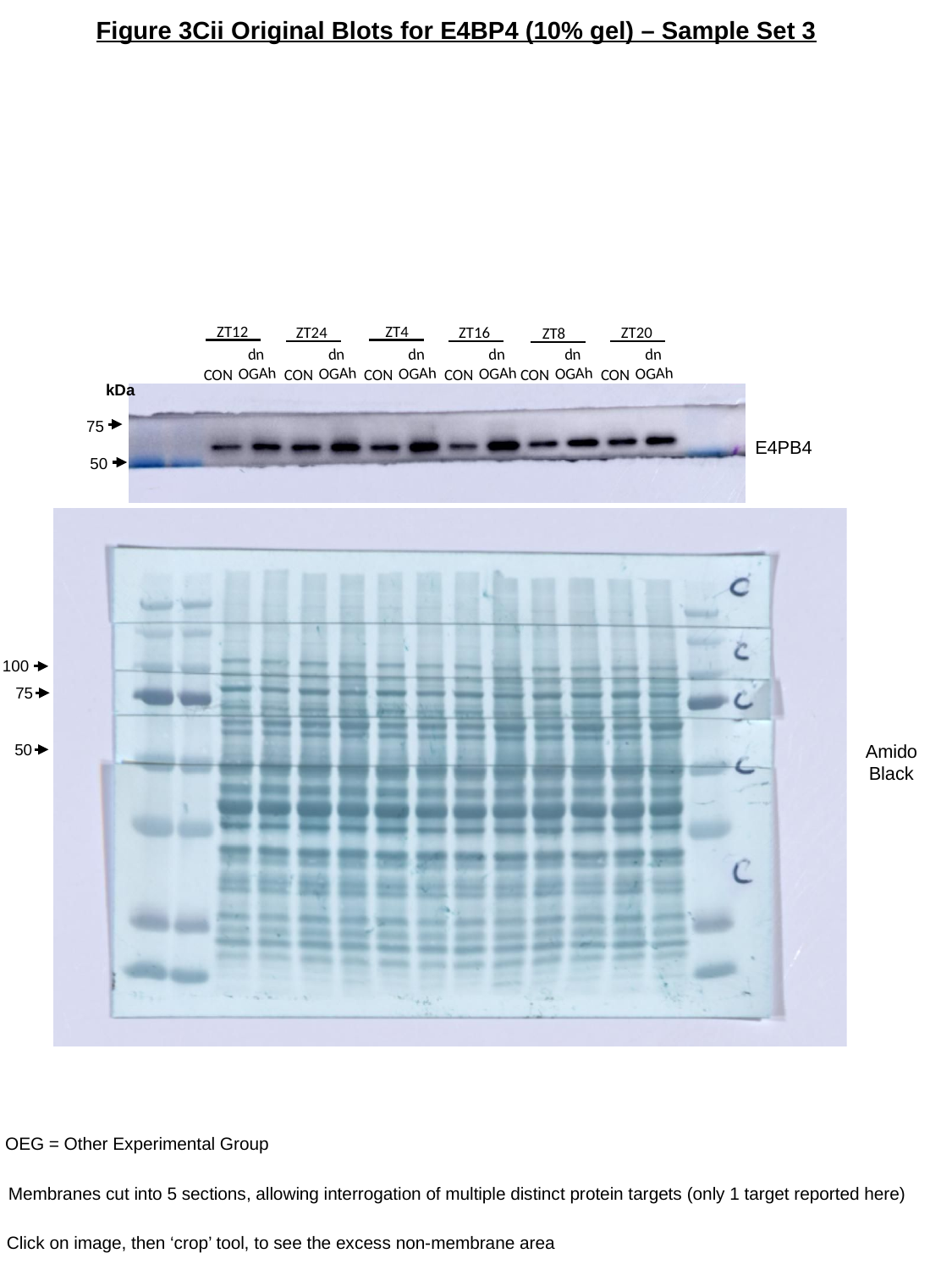

Figure 3Cii Original Blots for E4BP4 (10% gel) – Sample Set 3
ZT12
ZT4
ZT24
ZT20
ZT16
ZT8
dn
OGAh
dn
OGAh
dn
OGAh
dn
OGAh
dn
OGAh
dn
OGAh
CON
CON
CON
CON
CON
CON
kDa
75
E4PB4
50
100
75
50
Amido
Black
OEG = Other Experimental Group
Membranes cut into 5 sections, allowing interrogation of multiple distinct protein targets (only 1 target reported here)
Click on image, then ‘crop’ tool, to see the excess non-membrane area

## Slide 79
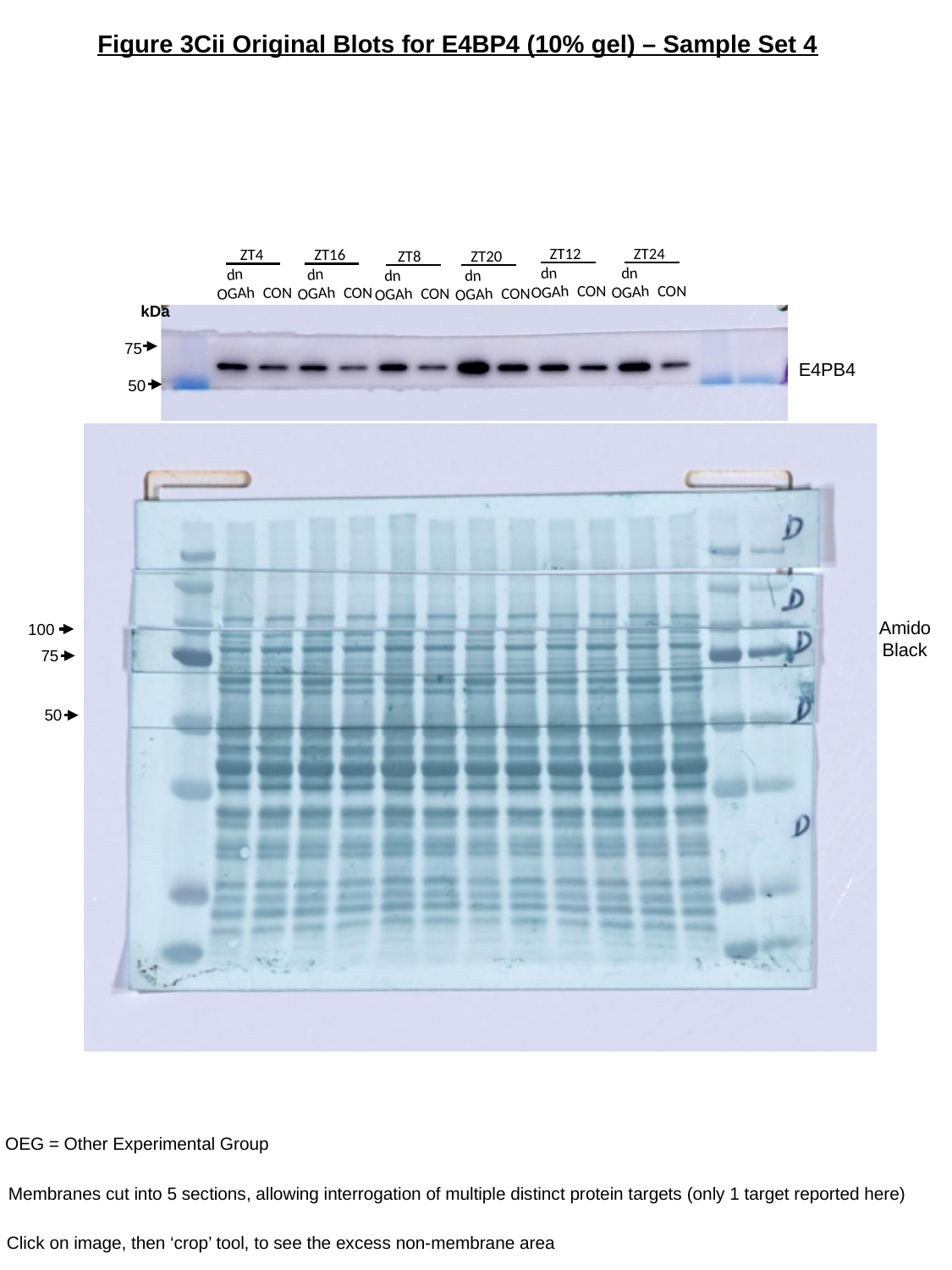

Figure 3Cii Original Blots for E4BP4 (10% gel) – Sample Set 4
ZT12
ZT24
ZT4
ZT16
ZT8
ZT20
dn
OGAh
dn
OGAh
dn
OGAh
dn
OGAh
dn
OGAh
dn
OGAh
CON
CON
CON
CON
CON
CON
kDa
75
E4PB4
50
Amido
Black
100
75
50
OEG = Other Experimental Group
Membranes cut into 5 sections, allowing interrogation of multiple distinct protein targets (only 1 target reported here)
Click on image, then ‘crop’ tool, to see the excess non-membrane area

## Slide 80
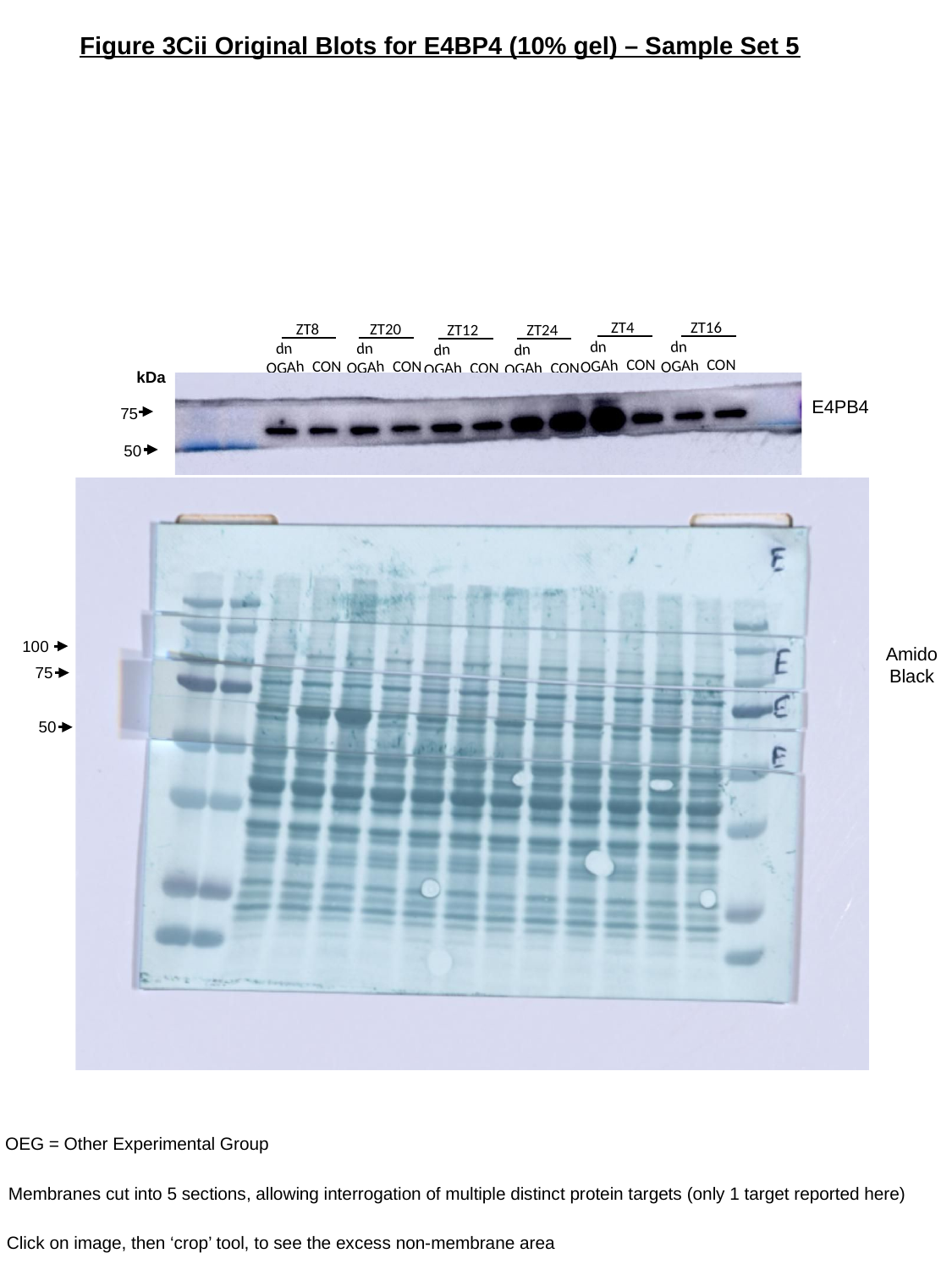

Figure 3Cii Original Blots for E4BP4 (10% gel) – Sample Set 5
ZT4
ZT16
ZT8
ZT20
ZT12
ZT24
dn
OGAh
dn
OGAh
dn
OGAh
dn
OGAh
dn
OGAh
dn
OGAh
CON
CON
CON
CON
CON
CON
kDa
E4PB4
75
50
100
Amido
Black
75
50
OEG = Other Experimental Group
Membranes cut into 5 sections, allowing interrogation of multiple distinct protein targets (only 1 target reported here)
Click on image, then ‘crop’ tool, to see the excess non-membrane area
