# Supplementary material for: Severe attenuation of circadian clock output in the heart following sustained augmentation of cardiomyocyte protein O-GlcNAcylation
Source: Front Cardiovasc Med. 2025 Jul 17;12:1601407. doi: 10.3389/fcvm.2025.1601407 (PMC12310464; doi:10.3389/fcvm.2025.1601407)

Figure 6Ci Original Picosirius Red Staining for Fibrosis - Control Heart 1

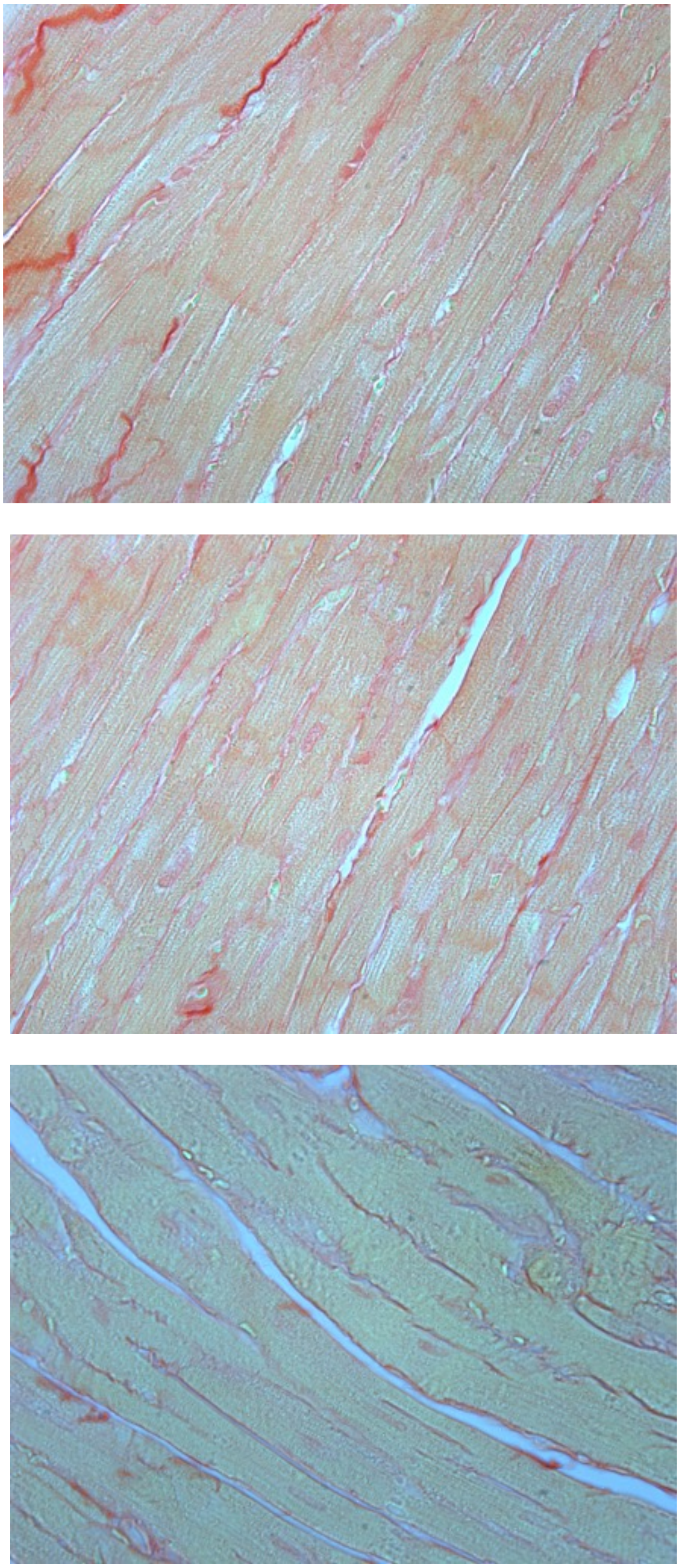

Figure 6Ci Original Picosirius Red Staining for Fibrosis - Control Heart 2

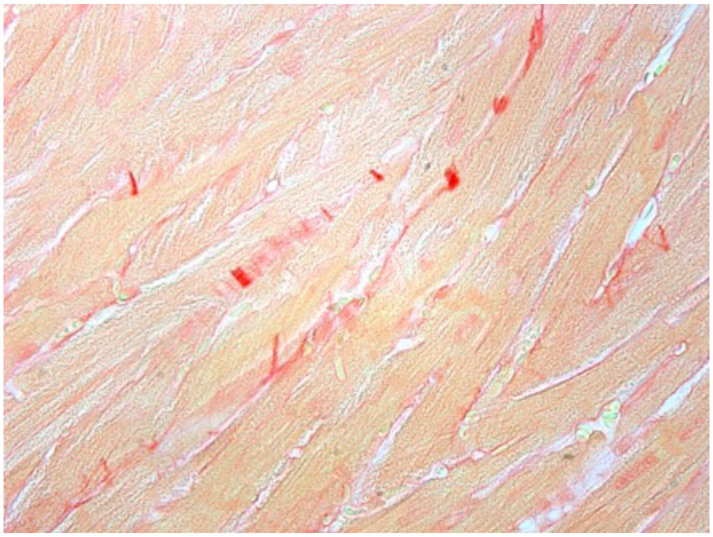

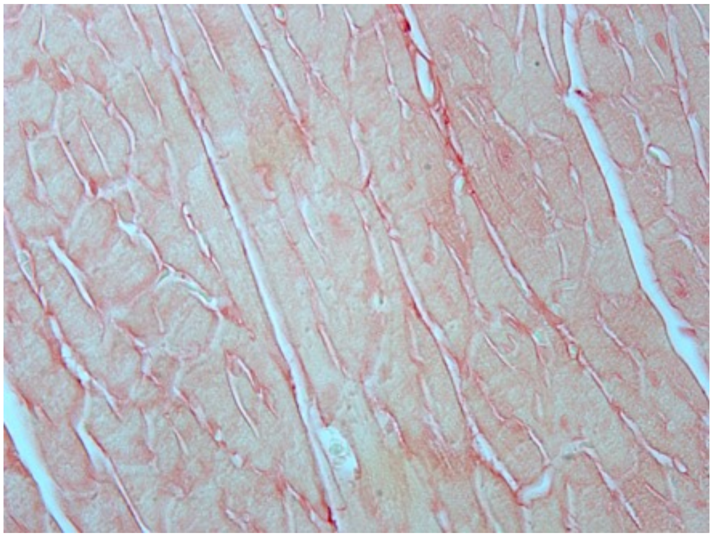

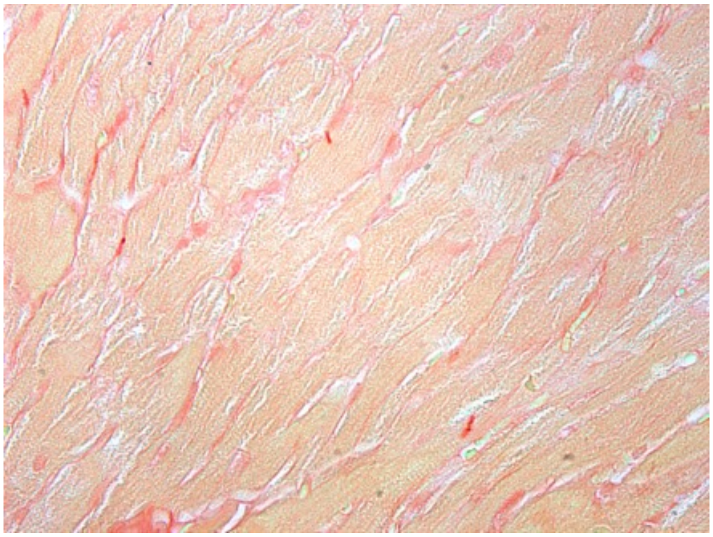

Figure 6Ci Original Picosirius Red Staining for Fibrosis - Control Heart 3

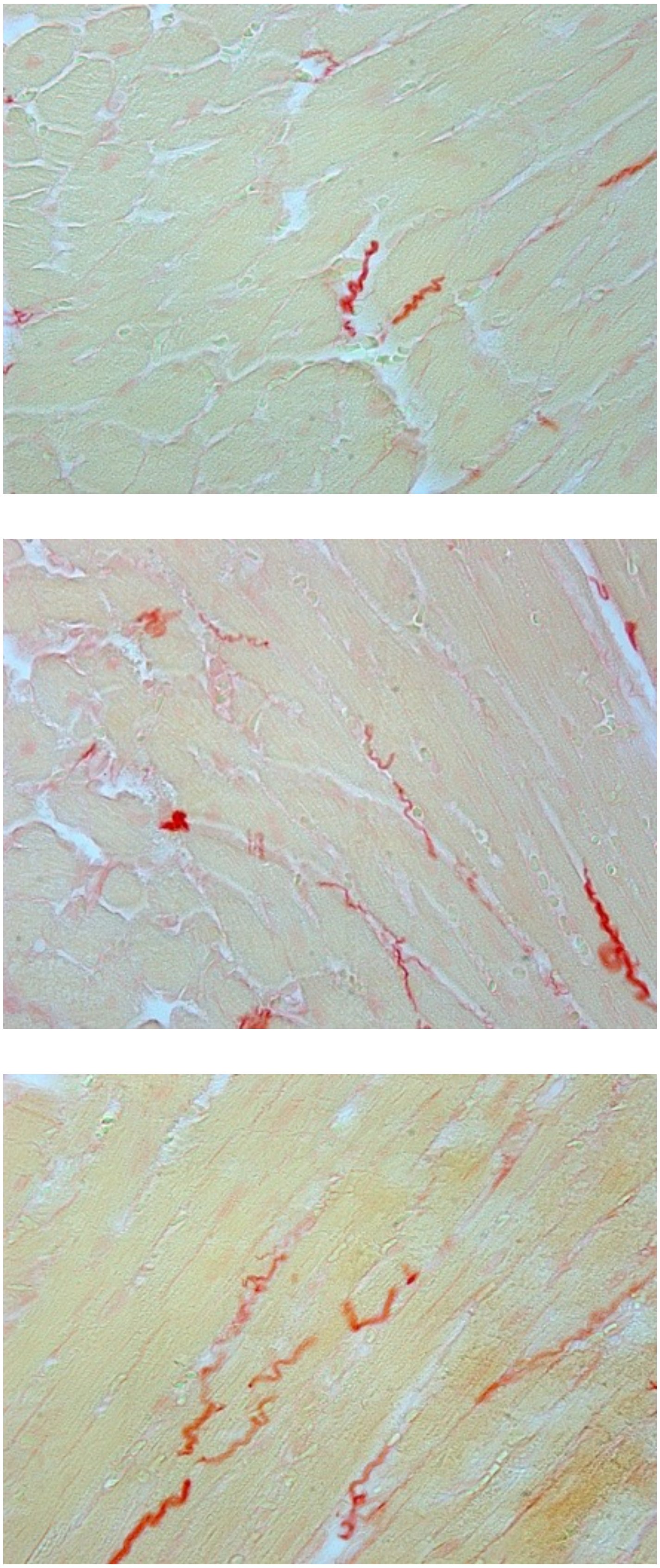

Figure 6Ci Original Picosirius Red Staining for Fibrosis - Control Heart 4

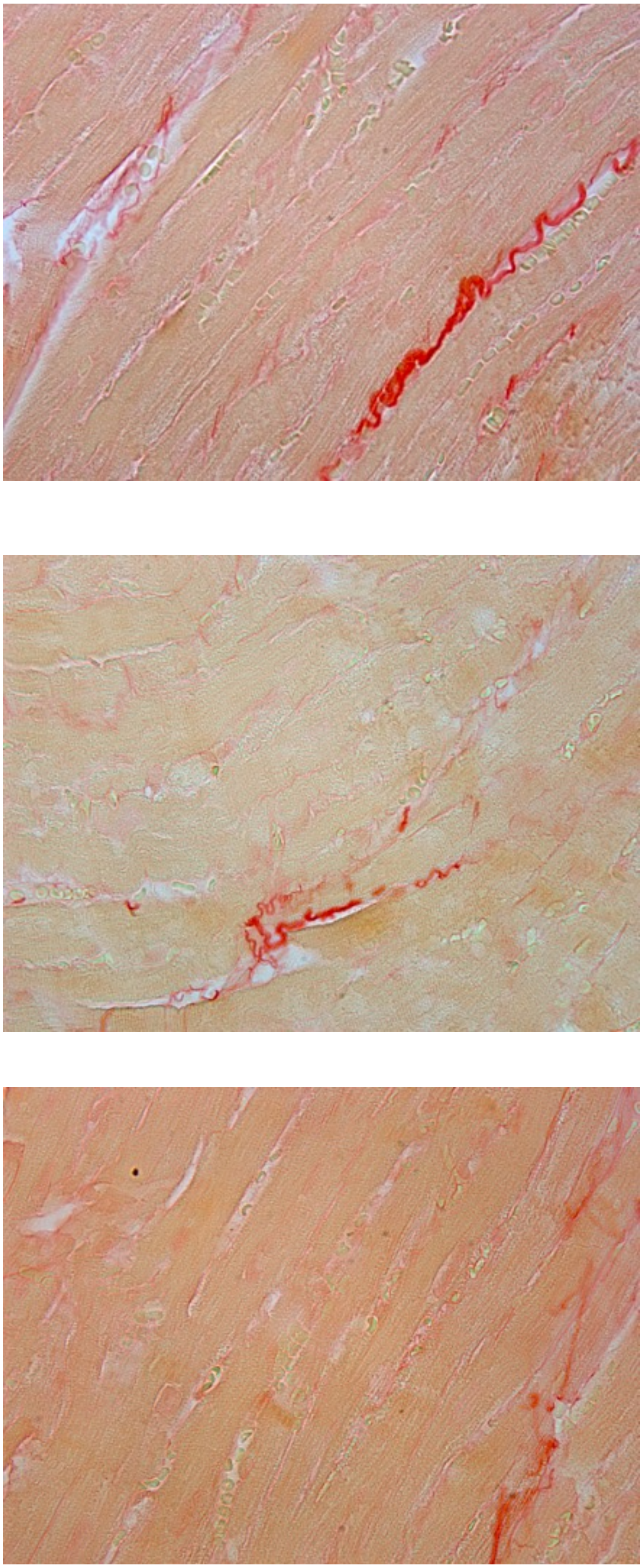

Figure 6Ci Original Picosirius Red Staining for Fibrosis - CBK Heart 1

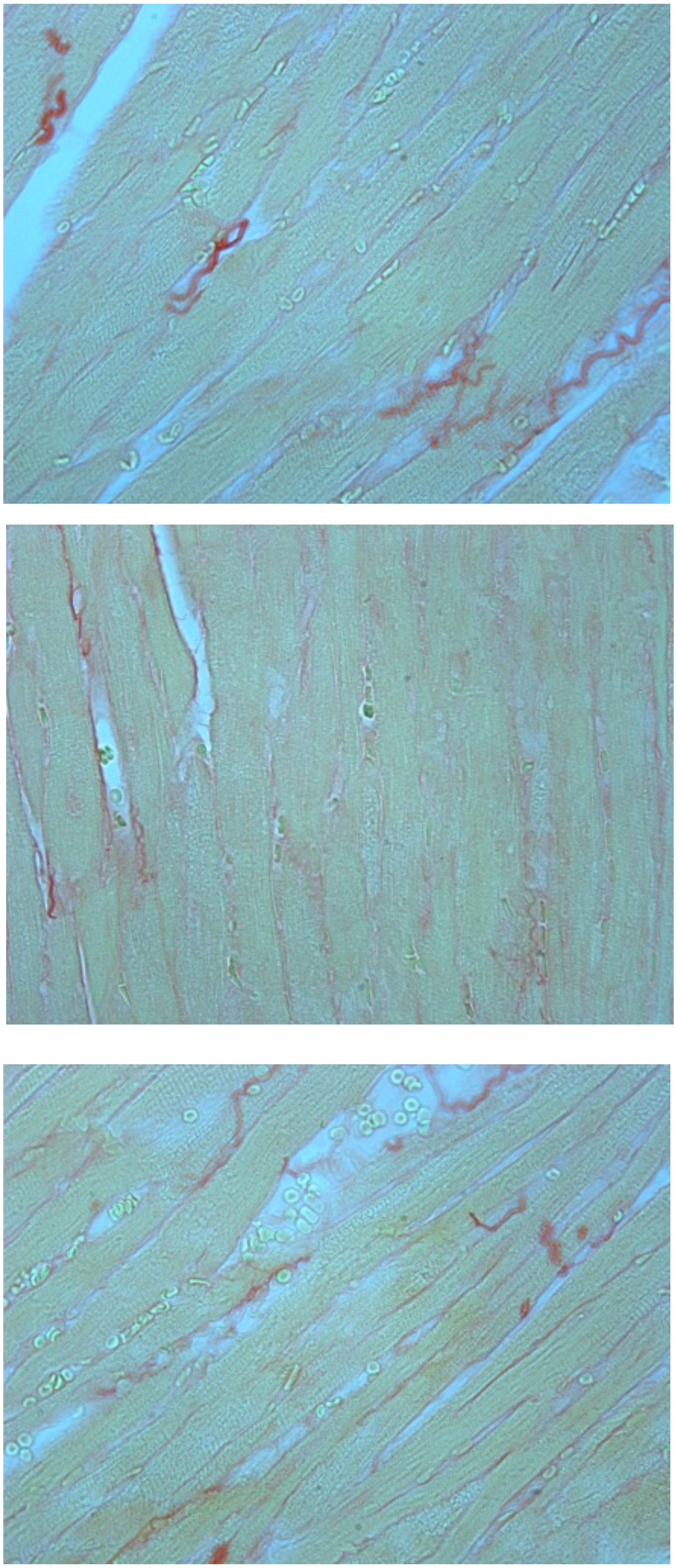

Figure 6Ci Original Picosirius Red Staining for Fibrosis – CBK Heart 2

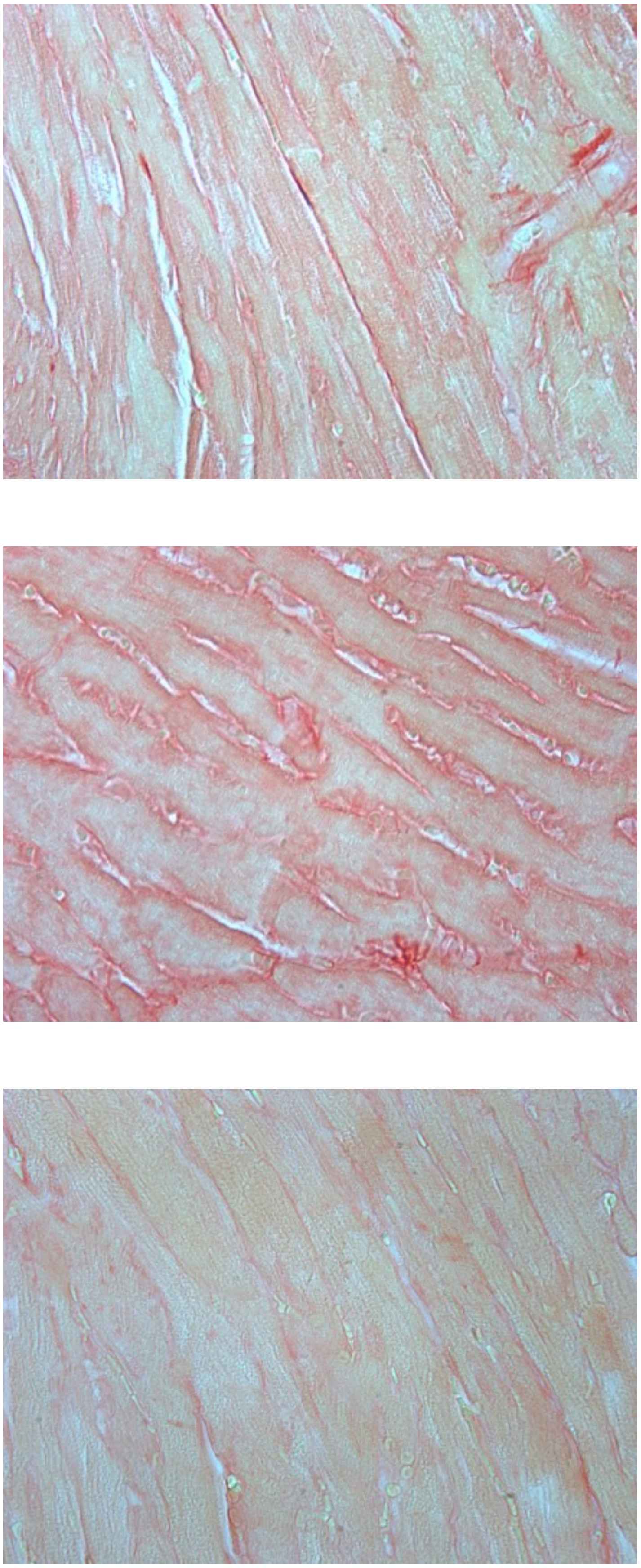

Figure 6Ci Original Picosirius Red Staining for Fibrosis - CBK Heart 3

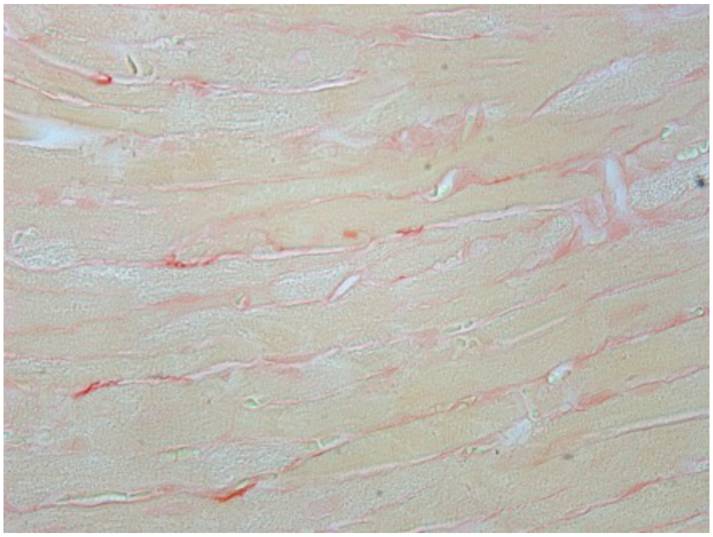

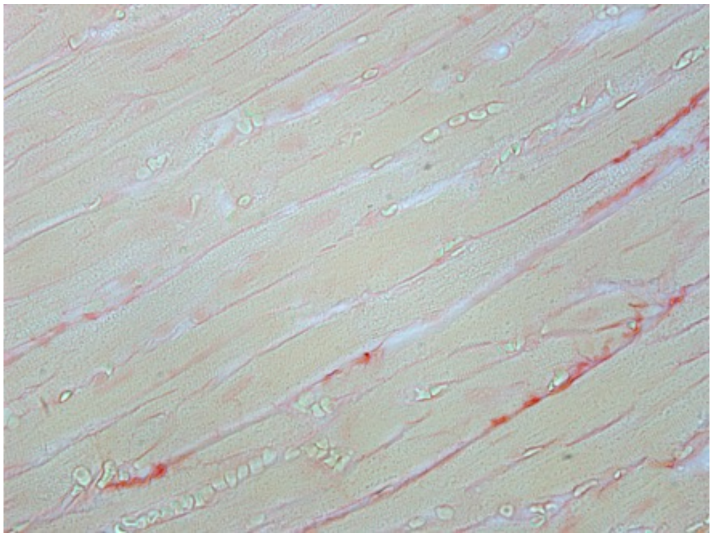

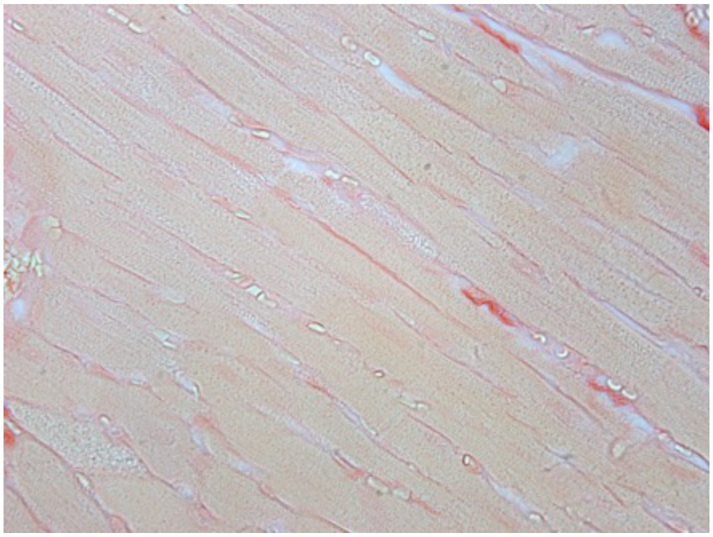

Figure 6Ci Original Picosirius Red Staining for Fibrosis - CBK Heart 4

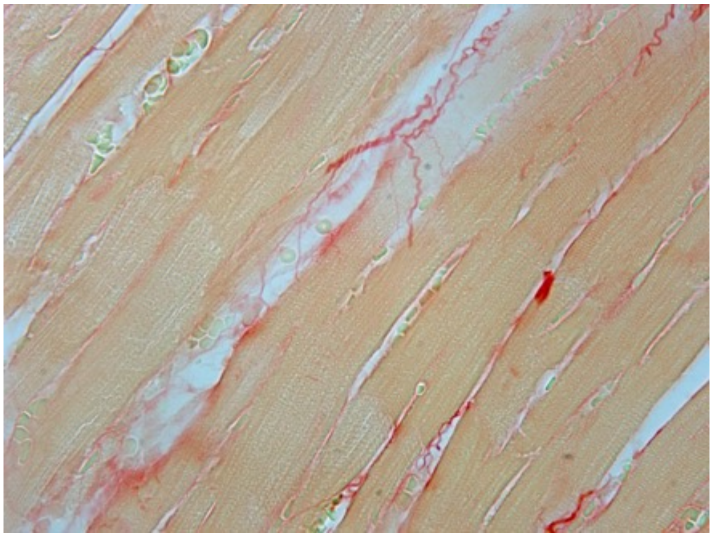

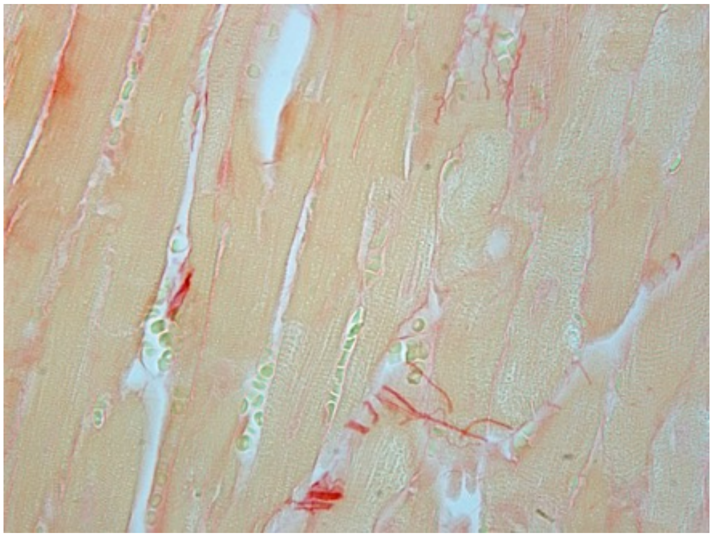

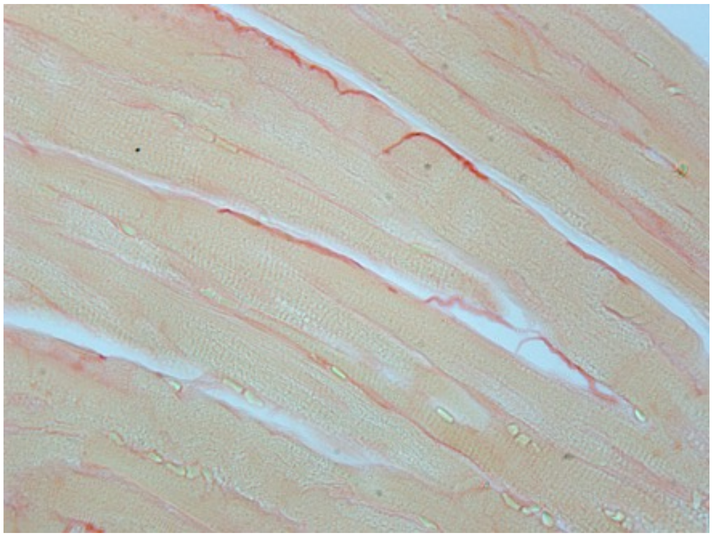

Figure 6Cii Original Trichrome Staining for Fibrosis - Control Heart 1

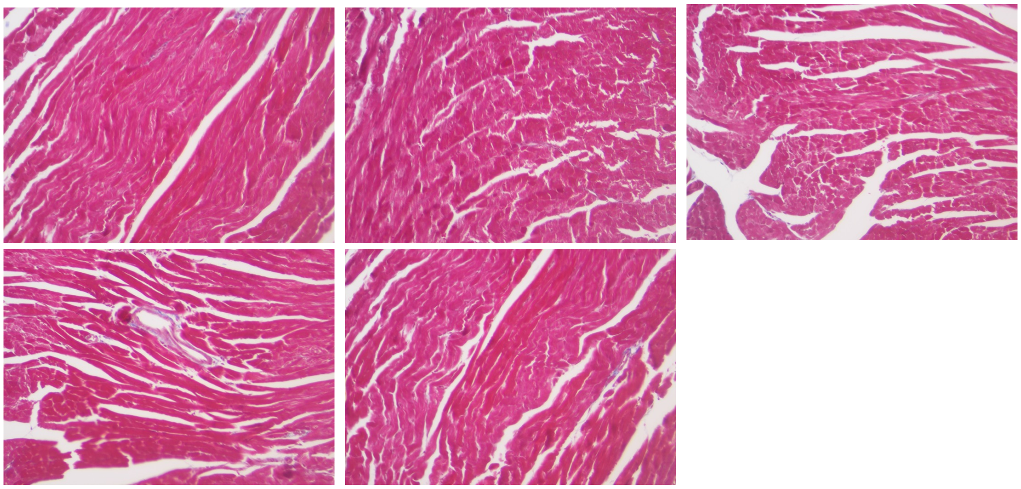

Figure 6Cii Original Trichrome Staining for Fibrosis - Control Heart 2

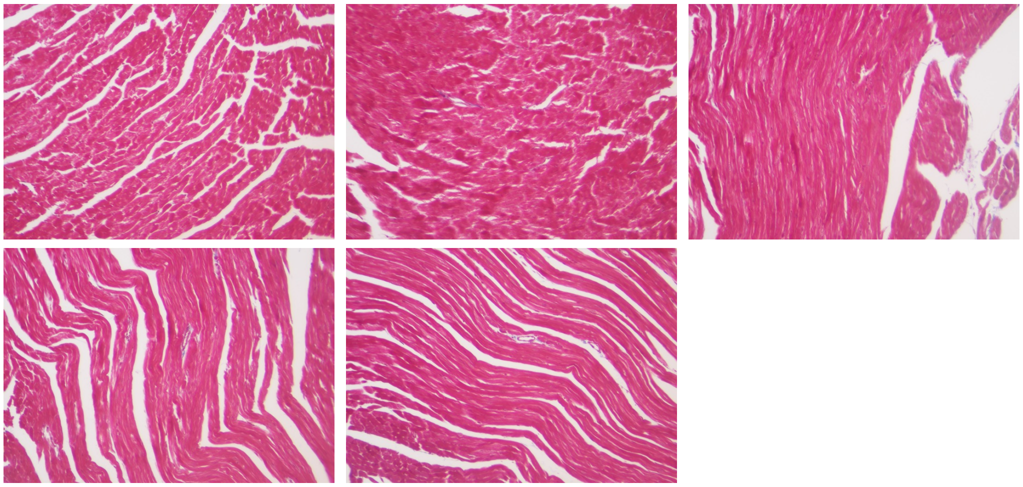

Figure 6Cii Original Trichrome Staining for Fibrosis - Control Heart 3

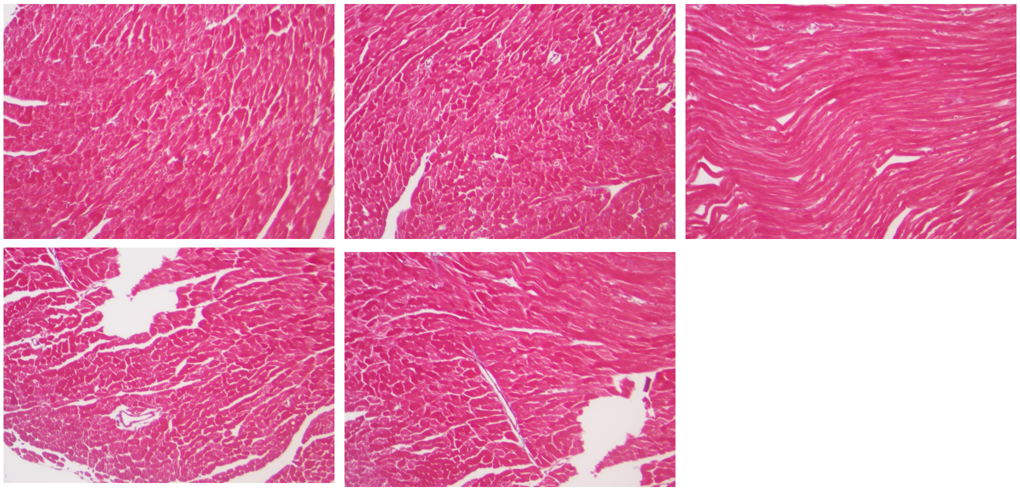

Figure 6Cii Original Trichrome Staining for Fibrosis - Control Heart 4

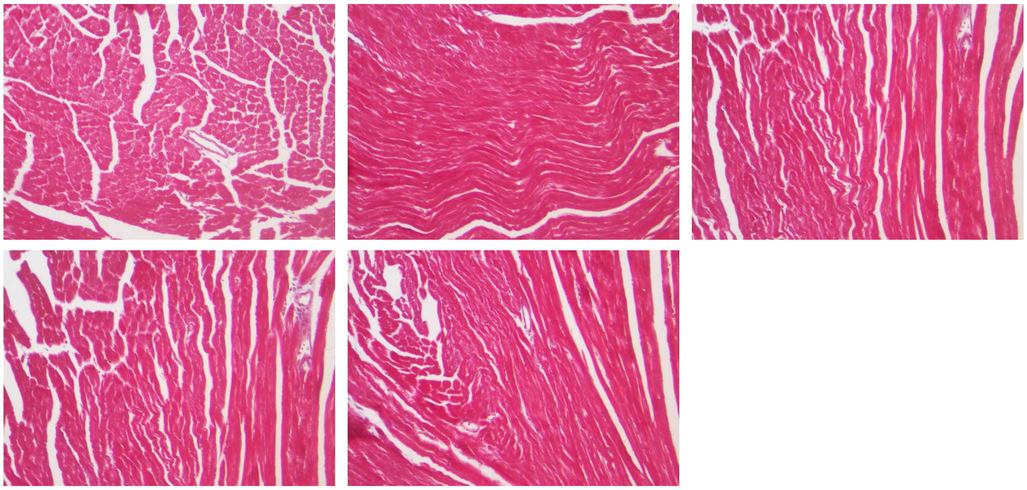

Figure 6Cii Original Trichrome Staining for Fibrosis - Control Heart 5

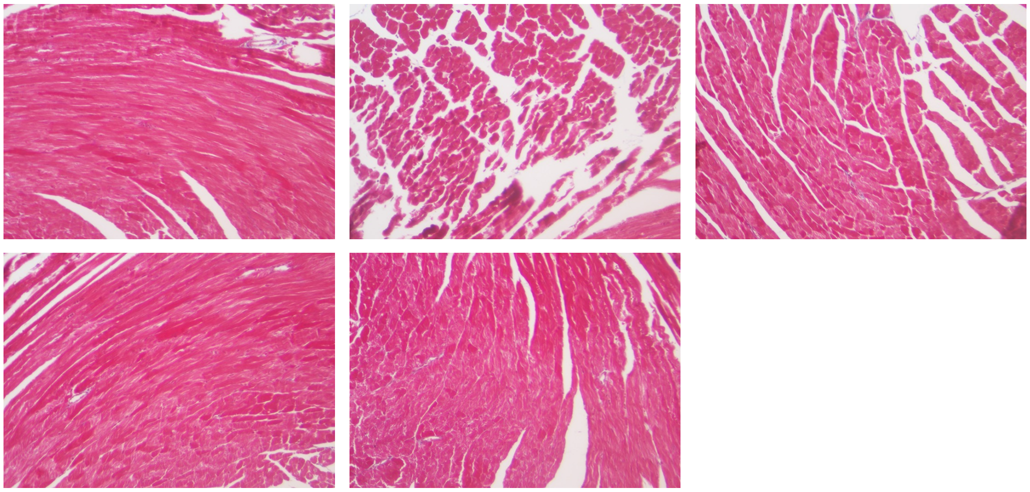

Figure 6Cii Original Trichrome Staining for Fibrosis - dnOGAh Heart 1

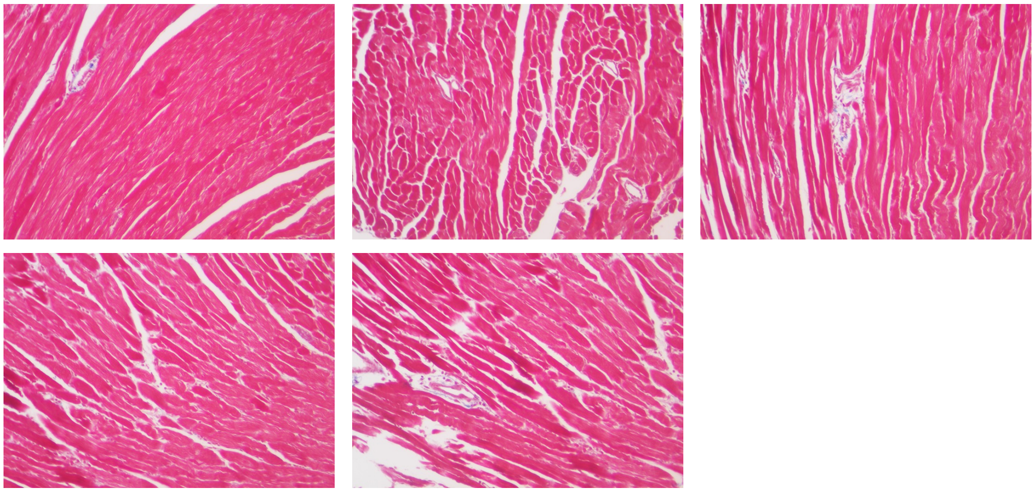

Figure 6Cii Original Trichrome Staining for Fibrosis - dnOGAh Heart 2

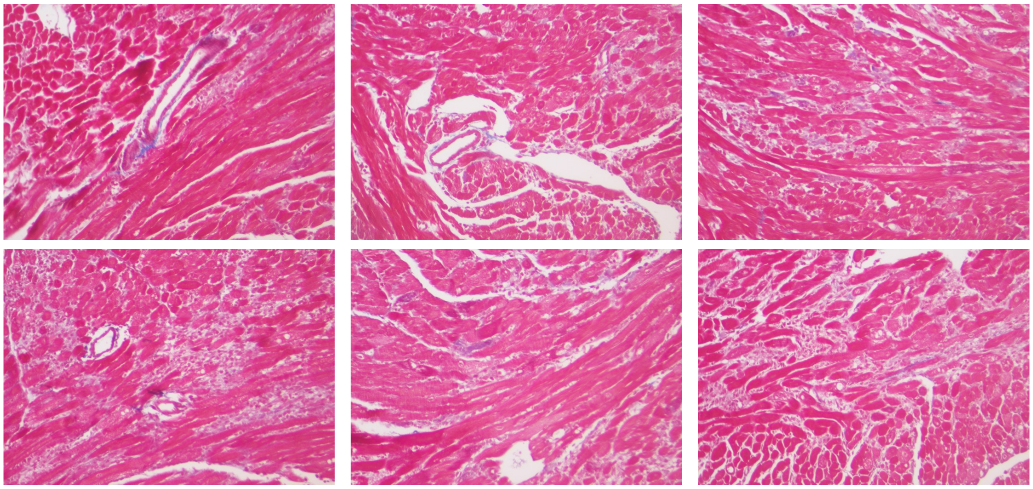

Figure 6Cii Original Trichrome Staining for Fibrosis - dnOGAh Heart 3

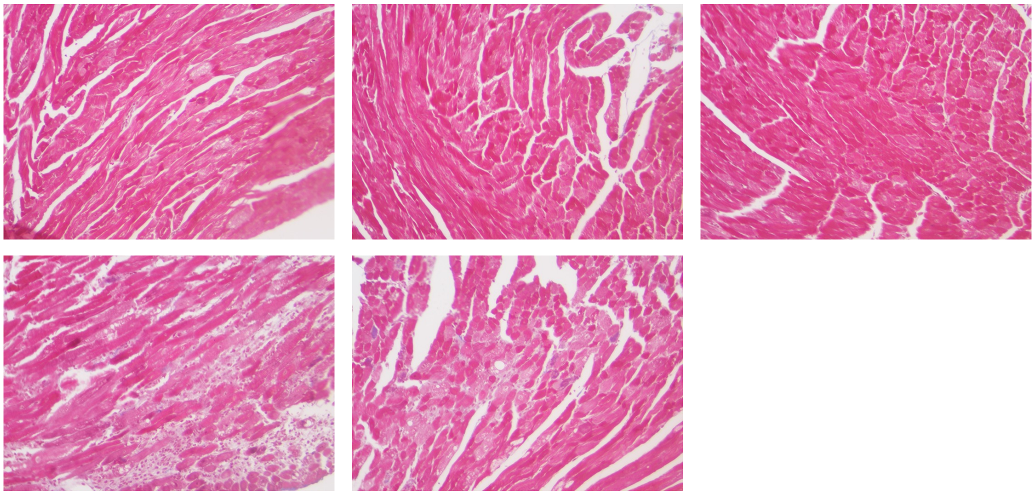

Figure 6Cii Original Trichrome Staining for Fibrosis - dnOGAh Heart 4

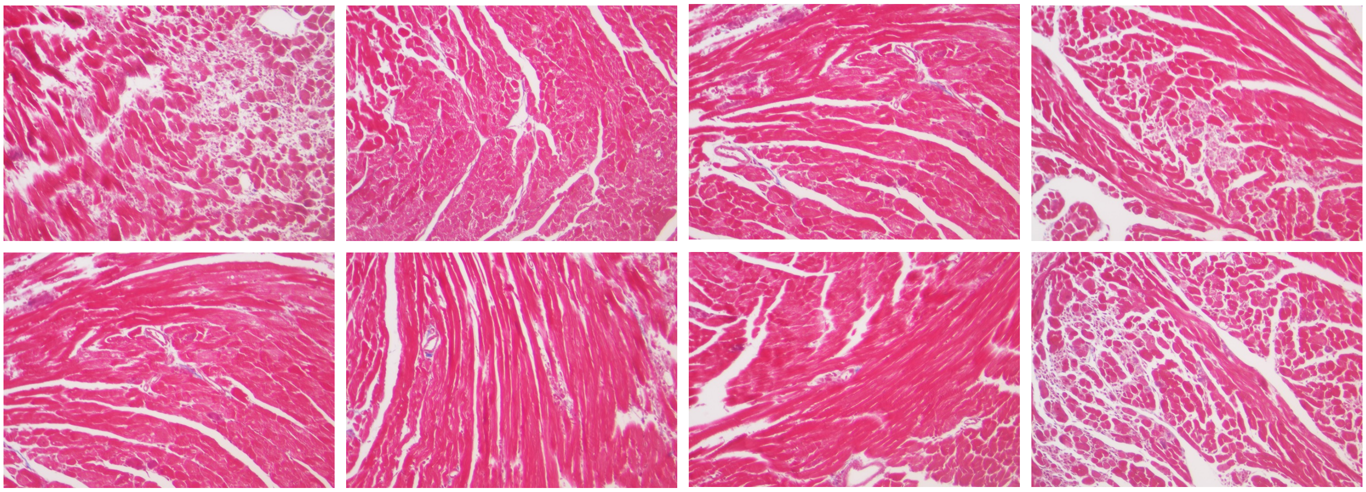

Figure 6Cii Original Trichrome Staining for Fibrosis - dnOGAh Heart 5

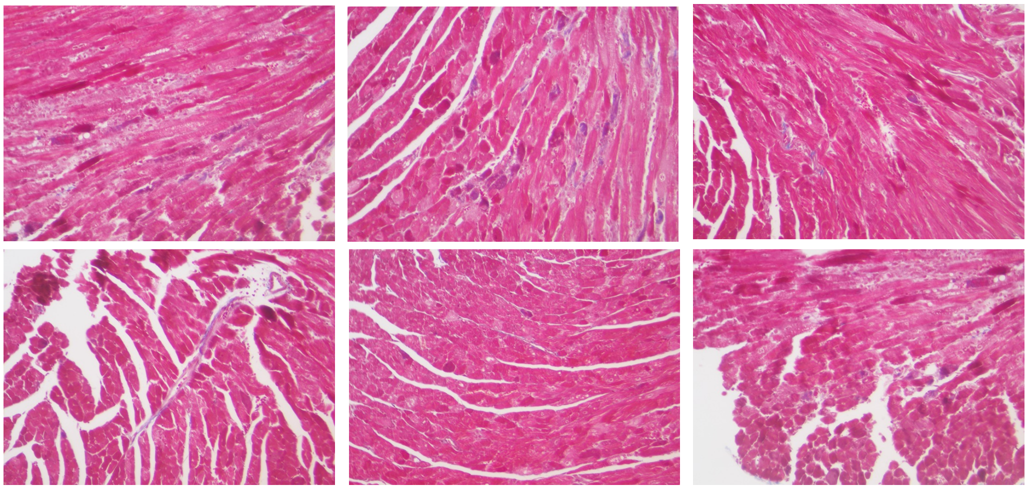

Supplement: Supplementary Figure 2 — Diurnal variations in hepatic gene expression in dnOGA mice. Livers were collected from dnOGAh and littermate CON mice at 4hr intervals over a 24 h period, 2 weeks after doxycycline treatment initiation, followed by RT-PCR analyses. mRNA levels of bmal1 (i), reverba (ii), and dbp (iii) in CON and dnOGAh livers (n = 5–9). ZT0 and ZT24 are identical (the data are double plotted purely for the sake of presentation). Data are presented as mean ± SEM, and have been normalized to the lowest (trough) value in CON hearts. Main effects of time are reported at the top of the figure panels. [file Presentation2.pdf]
