# Supplementary material for: Severe attenuation of circadian clock output in the heart following sustained augmentation of cardiomyocyte protein O-GlcNAcylation
Source: Front Cardiovasc Med. 2025 Jul 17;12:1601407. doi: 10.3389/fcvm.2025.1601407 (PMC12310464; doi:10.3389/fcvm.2025.1601407)
Supplement: Supplementary file 3 [file Datasheet1.pdf]

Parameter	CBK/CON Fold Change	Genotype Main Effect p-value	Time Main Effect p-value	Genotype-Time Interaction p-value
bmal1 mRNA	0.35	<0.0001	<0.0001	<0.0001
clock mRNA	1.57	<0.0001	<0.0001	0.0895
npas2 mRNA	2.57	<0.0001	0.0025	0.9455
reverbα mRNA	0.44	<0.0001	<0.0001	<0.0001
reverbβ mRNA	0.36	<0.0001	<0.0001	0.3441
per1 mRNA	0.61	<0.0001	<0.0001	0.0065
per2 mRNA	0.88	0.1828	<0.0001	0.2503
per3 mRNA	0.45	<0.0001	<0.0001	0.0051
cry1 mRNA	1.94	<0.0001	<0.0001	0.6610
cry2 mRNA	1.16	0.0379	0.1873	0.7535
dbp mRNA	0.36	<0.0001	<0.0001	<0.0001
tef mRNA	0.58	<0.0001	<0.0001	0.0144
e4bp4 mRNA	2.16	<0.0001	0.0006	0.1344
dgat2 mRNA	0.27	<0.0001	<0.0001	<0.0001
nampt mRNA	0.25	<0.0001	0.3117	0.5239
pik3r1 mRNA	0.70	<0.0001	<0.0001	0.7856
rhobtb1 mRNA	0.24	<0.0001	0.0001	0.0148
col1a1 mRNA	1.30	0.0062	0.0017	0.2468
col3a1 mRNA	1.40	<0.0001	0.0001	0.8778
col4a1 mRNA	1.62	<0.0001	0.0002	0.9786
BMAL1 Protein	0.64	<0.0001	<0.0001	0.5394
REVERBα Protein	0.54	<0.0001	<0.0001	<0.0001
PER2 Protein	1.23	0.0389	<0.0001	0.8370
E4BP4 Protein	2.03	<0.0001	0.0148	0.8700
Energy Expenditure	0.98	0.2700	<0.0001	0.2673
Food Intake	0.86	0.1951	0.0009	0.3694
Physical Activity	0.92	0.7053	0.0003	0.3165

Supplementary Table 1. 2-way ANOVA for cardiac mRNA and protein levels, as well as whole body parameters, in CBK and littermate flox control (CON) mice. Tissues were isolated from mice at distinct times of the day, followed by assessment of gene expression by RT-PCR and protein levels by Western Blotting. Whole body parameters were assessed by placement of mice within CLAMS cages. Data are reported as mean ± SEM, for 4-8 mice per experimental group. A 2-way ANOVA calculates time-of-day-independent values (i.e., daily average values) for parameters, which were utilized for CBK/CON fold change determination. p-values for genotype main effects, time main effects, and genotype-time interactions are reported.

Parameter	CON			CBK		
	**Mesor**	**Amplitude**	**Acrophase**	**Mesor**	**Amplitude**	**Acrophase**
bmal1 mRNA	9.81 ± 0.57	10.5 ± 0.80	12.9 ± 0.29	3.41 ± 0.25*	3.13 ± 0.35*	13.4 ± 0.43
clock mRNA	1.51 ± 0.04	0.49 ± 0.06	14.0 ± 0.47	NR	NR	NR
npas2 mRNA	3.05 ± 0.33	2.50 ± 0.47	13.7 ± 0.72	NR	NR	NR
reverbα mRNA	4.37 ± 0.24	4.40 ± 0.35	7.68 ± 0.30	1.94 ± 0.11*	1.78 ± 0.15*	9.02 ± 0.33*
reverbβ mRNA	1.69 ± 0.10	0.75 ± 0.14	11.6 ± 0.70	0.60 ± 0.05*	0.36 ± 0.07*	11.2 ± 0.72
per1 mRNA	2.46 ± 0.12	1.38 ± 0.16	11.8 ± 0.45	1.50 ± 0.09*	0.78 ± 0.13*	12.8 ± 0.63
per2 mRNA	5.25 ± 0.34	4.76 ± 0.49	13.7 ± 0.39	4.63 ± 0.33	3.55 ± 0.47	15.0 ± 0.50
per3 mRNA	2.99 ± 0.16	2.21 ± 0.23	12.8 ± 0.40	1.35 ± 0.10*	1.05 ± 0.15*	14.0 ± 0.53
cry1 mRNA	1.99 ± 0.09	0.95 ± 0.12	7.60 ± 0.50	3.86 ± 0.19*	0.89 ± 0.27	7.54 ± 1.16
cry2 mRNA	1.49 ± 0.07	0.36 ± 0.10	14.6 ± 1.03	NR	NR	NR
dbp mRNA	4.38 ± 0.30	4.32 ± 0.43	11.0 ± 0.38	1.56 ± 0.10*	1.04 ± 0.14*	11.1 ± 0.51
tef mRNA	1.74 ± 0.09	0.75 ± 0.13	15.6 ± 0.67	1.01 ± 0.06*	0.22 ± 0.08*	13.2 ± 1.33
e4bp4 mRNA	2.07 ± 0.10	1.13 ± 0.14	23.1 ± 0.47	NR	NR	NR
dgat2 mRNA	1.27 ± 0.03	0.31 ± 0.05	15.8 ± 0.55	NR	NR	NR
nampt mRNA	NR	NR	NR	NR	NR	NR
pik3r1 mRNA	1.30 ± 0.05	0.30 ± 0.06	15.5 ± 0.82	0.91 ± 0.04*	0.19 ± 0.05	13.8 ± 1.04
rhobtb1 mRNA	1.63 ± 0.13	0.81 ± 0.18	15.1 ± 0.84	NR	NR	NR
BMAL1 Protein	1.30 ± 0.05	0.25 ± 0.07	0.71 ± 1.11	0.83 ± 0.03*	0.18 ± 0.05	1.52 ± 0.95
REVERBα Protein	3.31 ± 0.21	3.16 ± 0.30	10.0 ± 0.36	1.80 ± 0.15*	1.34 ± 0.21*	11.4 ± 0.59
PER2 Protein	1.93 ± 0.14	1.13 ± 0.20	17.3 ± 0.67	2.39 ± 0.18	1.53 ± 0.26	17.9 ± 0.64
E4BP4 Protein	2.06 ± 0.13	0.66 ± 0.18	11.0 ± 1.04	NR	NR	NR
Energy Expenditure	0.40 ± 0.00	0.05 ± 0.01	18.2 ± 0.38	0.39 ± 0.00	0.07 ± 0.01	18.3 ± 0.33
Food Intake	0.21 ± 0.01	0.08 ± 0.02	17.6 ± 0.91	0.18 ± 0.01	0.11 ± 0.02	17.1 ± 0.64
Physical Activity	1661 ± 144	516 ± 204	16.1 ± 1.51	1525 ± 119	895 ± 169	17.1 ± 0.72

Supplementary Table 2. Cosinor analysis for cardiac mRNA and protein levels, as well as whole body parameters, in CBK and littermate flox control (CON) mice. Tissues were isolated from mice at distinct times of the day, followed by assessment of gene expression by RT-PCR and protein levels by Western Blotting. Whole body parameters were assessed by placement of mice within CLAMS cages. Data are reported as mean ± SEM, for 4-8 mice per experimental group. *, $p<0.05$ for CON versus CBK. NR; not rhythmic.

Parameter	dnOGAh/CON Fold Change	Genotype Main Effect p-value	Time Main Effect p-value	Genotype-Time Interaction p-value
bmal1 mRNA	1.26	0.0010	<0.0001	0.8638
clock mRNA	1.14	0.0539	0.0003	0.8418
npas2 mRNA	1.29	0.0309	<0.0001	0.1744
reverbα mRNA	0.97	0.8212	<0.0001	0.6353
reverbβ mRNA	0.75	0.0027	<0.0001	0.5941
per1 mRNA	1.16	0.2174	<0.0001	0.9565
per2 mRNA	1.17	0.0340	<0.0001	0.1454
per3 mRNA	0.59	0.0002	<0.0001	0.4276
cry1 mRNA	0.84	0.0111	<0.0001	0.5275
cry2 mRNA	0.69	0.0010	<0.0001	0.8678
dbp mRNA	0.51	<0.0001	<0.0001	0.0118
tef mRNA	0.73	0.0007	<0.0001	0.6929
e4bp4 mRNA	1.17	0.0439	<0.0001	0.4381
dgat2 mRNA	0.45	<0.0001	0.2052	0.7111
nampt mRNA	0.73	0.0196	0.2243	0.5638
pik3r1 mRNA	0.76	0.0004	0.0377	0.8360
rhobtb1 mRNA	0.71	0.0026	0.0003	0.2446
col1a1 mRNA	14.49	<0.0001	0.2333	0.2163
col3a1 mRNA	17.18	<0.0001	0.0378	0.0513
col4a1 mRNA	2.41	<0.0001	0.1334	0.6774
BMAL1 Protein	1.52	<0.0001	0.0003	0.6165
REVERBα Protein	1.01	0.9163	<0.0001	0.7592
PER2 Protein	2.21	<0.0001	<0.0001	0.0107
E4BP4 Protein	1.53	<0.0001	0.0002	0.2918
Energy Expenditure	0.98	0.7137	<0.0001	0.2793
Food Intake	1.01	0.9095	<0.0001	0.1745
Physical Activity	1.24	0.1456	<0.0001	0.1235

Supplementary Table 3. 2-way ANOVA for cardiac mRNA and protein levels, as well as whole body parameters, in dnOGAh and littermate flox control (CON) mice, 2wks after initiation of doxycycline treatment. Tissues were isolated from mice at distinct times of the day, followed by assessment of gene expression by RT-PCR and protein levels by Western Blotting. Whole body parameters were assessed by placement of mice within CLAMS cages. Data are reported as mean ± SEM, for 5-9 mice per experimental group. A 2-way ANOVA calculates time-of-day-independent values (i.e., daily average values) for parameters, which were utilized for dnOGAh/CON fold change determination. p-values for genotype main effects, time main effects, and genotype-time interactions are reported.

Parameter	CON			dnOGA		
	**Mesor**	**Amplitude**	**Acrophase**	**Mesor**	**Amplitude**	**Acrophase**
bmal1 mRNA	6.20 ± 0.35	6.75 ± 0.49	23.4 ± 0.28	7.91 ± 0.43*	6.29 ± 0.60	23.5 ± 0.37
clock mRNA	1.35 ± 0.06	0.39 ± 0.08	23.1 ± 0.81	NR	NR	NR
npas2 mRNA	2.78 ± 0.22	2.20 ± 0.31	0.13 ± 0.54	3.57 ± 0.30*	3.43 ± 0.43*	0.55 ± 0.48
reverbα mRNA	3.41 ± 0.30	2.80 ± 0.43	7.37 ± 0.57	3.35 ± 0.25	1.90 ± 0.35	7.13 ± 0.70
reverbβ mRNA	1.81 ± 0.10	0.92 ± 0.14	11.1 ± 0.57	1.36 ± 0.11*	0.56 ± 0.15	10.5 ± 1.07
per1 mRNA	2.67 ± 0.23	1.71 ± 0.33	11.2 ± 0.74	3.12 ± 0.24	1.38 ± 0.33	11.5 ± 0.94
per2 mRNA	2.35 ± 0.10	1.48 ± 0.15	12.4 ± 0.38	2.74 ± 0.16*	1.06 ± 0.22	14.1 ± 0.79
per3 mRNA	2.18 ± 0.16	1.40 ± 0.23	12.1 ± 0.63	1.30 ± 0.13*	0.90 ± 0.18	12.8 ± 0.78
cry1 mRNA	2.09 ± 0.09	1.17 ± 0.13	19.5 ± 0.41	1.79 ± 0.09*	0.93 ± 0.13	19.1 ± 0.52
cry2 mRNA	1.53 ± 0.06	0.37 ± 0.09	13.4 ± 0.94	1.07 ± 0.04*	0.24 ± 0.06	14.5 ± 0.86
dbp mRNA	3.84 ± 0.36	3.85 ± 0.51	10.2 ± 0.49	2.01 ± 0.15*	1.75 ± 0.21*	10.4 ± 0.47
tef mRNA	1.86 ± 0.10	0.62 ± 0.13	15.0 ± 0.88	1.37 ± 0.09*	0.49 ± 0.13	13.9 ± 0.98
e4bp4 mRNA	1.90 ± 0.10	1.27 ± 0.15	21.4 ± 0.42	2.25 ± 0.14*	1.02 ± 0.20	21.2 ± 0.75
dgat2 mRNA	1.32 ± 0.07	0.26 ± 0.09	15.8 ± 1.37	NR	NR	NR
nampt mRNA	1.39 ± 0.11	0.46 ± 0.16	17.6 ± 1.34	NR	NR	NR
pik3r1 mRNA	1.21 ± 0.05	0.24 ± 0.07	14.4 ± 1.21	NR	NR	NR
rhobtb1 mRNA	2.02 ± 0.14	0.86 ± 0.19	14.4 ± 0.93	1.43 ± 0.10	0.38 ± 0.14	15.5 ± 1.39
BMAL1 Protein	1.27 ± 0.04	0.28 ± 0.05	23.6 ± 0.72	1.94 ± 0.10*	0.52 ± 0.14	0.22 ± 1.01
REVERBα Protein	6.62 ± 0.51	6.26 ± 0.72	9.31 ± 0.44	6.69 ± 0.54	6.77 ± 0.76	9.68 ± 0.43
PER2 Protein	1.61 ± 0.12	0.61 ± 0.17	16.3 ± 1.05	3.57 ± 0.34*	2.69 ± 0.48*	16.9 ± 0.69
E4BP4 Protein	1.39 ± 0.10	0.35 ± 0.14	23.5 ± 1.50	2.13 ± 0.13*	0.75 ± 0.19	25.2 ± 0.95
Energy Expenditure	0.39 ± 0.00	0.08 ± 0.01	17.0 ± 0.24	0.38 ± 0.01	0.08 ± 0.01	16.9 ± 0.30
Food Intake	0.18 ± 0.01	0.17 ± 0.01	16.2 ± 0.28	0.19 ± 0.01	0.17 ± 0.02	15.8 ± 0.33
Physical Activity	901 ± 43	823 ± 61	17.1 ± 0.29	1117 ± 66*	877 ± 93	16.9 ± 0.40

Supplementary Table 4. Cosinor analysis for cardiac mRNA and protein levels, as well as whole body parameters, in dnOGAh and littermate flox control (CON) mice, 2wks after initiation of doxycycline treatment. Tissues were isolated from mice at distinct times of the day, followed by assessment of gene expression by RT-PCR and protein levels by Western Blotting. Whole body parameters were assessed by placement of mice within CLAMS cages. Data are reported as mean ± SEM, for 5-9 mice per experimental group. *, p<0.05 for CON versus dnOGAh. NR; not rhythmic.

Gene ID	Gene Symbol	Goodness of Fit q-value
ENSMUSG00000090066	1110002E22Rik	0.013091819
ENSMUSG00000099411	2310015D24Rik	0.003754435
ENSMUSG00000031983	2310022B05Rik	0.003214659
ENSMUSG00000045411	2410002F23Rik	0.014692162
ENSMUSG00000099696	2900052N01Rik	0.033071808
ENSMUSG00000071540	3425401B19Rik	0.002231251
ENSMUSG00000086938	4930481A15Rik	0.006085424
ENSMUSG00000086607	4930511M06Rik	0.011575813
ENSMUSG00000046138	9930021J03Rik	0.025495919
ENSMUSG00000029408	Abcb9	0.019243424
ENSMUSG00000029095	Ablim2	0.000376217
ENSMUSG00000042895	Abra	0.022168781
ENSMUSG00000030083	Abtb1	0.013091819
ENSMUSG00000038007	Acer2	0.001869435
ENSMUSG00000034853	Acot11	0.028824062
ENSMUSG00000029580	Actb	0.004475787
ENSMUSG00000062825	Actg1	0.008381805
ENSMUSG00000054693	Adam10	0.019243424
ENSMUSG00000011256	Adam19	0.000200016
ENSMUSG00000006403	Adamts4	0.018806464
ENSMUSG00000025026	Add3	0.044228069
ENSMUSG00000039167	Adgrl4	0.033071808
ENSMUSG00000074207	Adh1	8.32808E-05
ENSMUSG00000039041	Adrm1	0.028824062
ENSMUSG00000033032	Afap1l1	0.001542731
ENSMUSG00000025422	Agap2	0.004475787
ENSMUSG00000049115	Agtr1a	0.008381805
ENSMUSG00000069833	Ahnak	0.001542731
ENSMUSG00000047423	AI837181	0.016916742
ENSMUSG00000018428	Akap1	0.000376217
ENSMUSG00000021057	Akap5	0.001869435
ENSMUSG00000053279	Aldh1a1	4.64985E-05
ENSMUSG00000075232	Amd1	0.001250287
ENSMUSG00000036820	Amdhd2	0.011575813
ENSMUSG00000027889	Ampd2	0.008381805
ENSMUSG00000036977	Anapc10	0.014692162
ENSMUSG00000004105	Angptl2	0.001250287
ENSMUSG00000028989	Angptl7	0.022168781
ENSMUSG00000004849	Ap1s1	0.028824062
ENSMUSG00000044338	Aplnr	0.001542731
ENSMUSG00000050014	Apol10b	0.022168781
ENSMUSG00000004655	Aqp1	0.016916742
ENSMUSG00000028427	Aqp7	0.007203
ENSMUSG00000037999	Arap2	0.001064763
ENSMUSG00000039031	Arhgap18	0.001542731
ENSMUSG00000039831	Arhgap29	0.002686921
ENSMUSG00000025132	Arhgdia	0.033071808
ENSMUSG00000040964	Arhgef10l	0.005252394
ENSMUSG00000059495	Arhgef12	0.019243424
ENSMUSG00000028919	Arhgef19	0.033071808
ENSMUSG00000027599	Armc1	0.033071808
ENSMUSG00000055116	Arntl	2.35E-09
ENSMUSG00000002910	Arrdc2	0.000717948
ENSMUSG00000030996	Art1	0.000252129
ENSMUSG00000070424	Art5	0.011575813
ENSMUSG00000021200	Asb2	0.022168781
ENSMUSG00000028207	Asph	0.014692162
ENSMUSG00000026663	Atf6	0.000126318
ENSMUSG00000029673	Auts2	0.000873619
ENSMUSG00000075010	AW112010	0.025495919
ENSMUSG00000025372	Baiap2	0.001250287
ENSMUSG00000002748	Baz1b	0.006085424

ENSMUSG00000007659	Bcl2l1	0.001869435
ENSMUSG00000000317	Bcl6b	0.003214659
ENSMUSG00000030103	Bhlhe40	0.000717948
ENSMUSG00000030256	Bhlhe41	0.000583768
ENSMUSG00000016758	Bik	0.008381805
ENSMUSG00000024335	Brd2	0.019243424
ENSMUSG00000029544	Cabp1	0.009968119
ENSMUSG00000046447	Camk2n1	0.025495919
ENSMUSG00000024942	Capn1	0.028824062
ENSMUSG00000001794	Capns1	0.013091819
ENSMUSG00000027184	Caprin1	0.009968119
ENSMUSG00000000805	Car4	0.009968119
ENSMUSG00000045954	Cavin2	0.025495919
ENSMUSG00000020074	Ccar1	0.003214659
ENSMUSG00000020925	Ccdc43	0.028824062
ENSMUSG00000095098	Ccdc85b	0.014692162
ENSMUSG00000029617	Ccz1	0.011575813
ENSMUSG00000025351	Cd63	0.025495919
ENSMUSG00000027435	Cd93	0.006085424
ENSMUSG00000027330	Cdc25b	0.014692162
ENSMUSG00000024780	Cdc37l1	0.003754435
ENSMUSG00000020015	Cdk17	0.011575813
ENSMUSG00000021483	Cdk20	0.033071808
ENSMUSG00000023067	Cdkn1a	0.028824062
ENSMUSG00000037443	Cep85	0.014692162
ENSMUSG00000027313	Chac1	0.006085424
ENSMUSG00000020309	Chac2	0.003754435
ENSMUSG00000057133	Chd6	0.019243424
ENSMUSG00000041235	Chd7	0.022168781
ENSMUSG00000000743	Chmp1a	0.028824062
ENSMUSG00000001774	Chordc1	0.000252129
ENSMUSG00000006958	Chrd	0.001250287
ENSMUSG00000038550	Ciart	0.001250287
ENSMUSG00000034157	Cipc	0.002231251
ENSMUSG00000045193	Cirbp	0.000297339
ENSMUSG00000046841	Ckap4	0.003214659
ENSMUSG00000064302	Clasp1	7.05E-08
ENSMUSG00000049550	Clip1	0.014692162
ENSMUSG00000063146	Clip2	0.016916742
ENSMUSG00000029238	Clock	1.08307E-06
ENSMUSG00000015357	Clpx	0.019243424
ENSMUSG00000022037	Clu	0.033071808
ENSMUSG00000031875	Cmtm3	0.011575813
ENSMUSG00000032434	Cmtm6	0.001250287
ENSMUSG00000028339	Col15a1	0.000297339
ENSMUSG00000001506	Col1a1	0.003754435
ENSMUSG00000026043	Col3a1	0.000873619
ENSMUSG00000031502	Col4a1	0.000449642
ENSMUSG00000031503	Col4a2	7.22416E-06
ENSMUSG00000026837	Col5a1	0.028824062
ENSMUSG00000004098	Col5a3	3.6994E-06
ENSMUSG00000001119	Col6a1	0.014692162
ENSMUSG00000020241	Col6a2	0.013091819
ENSMUSG00000057606	Colq	0.009968119
ENSMUSG00000025981	Coq10b	0.002686921
ENSMUSG00000026489	Coq8a	0.003754435
ENSMUSG00000025586	Cpeb1	0.007203
ENSMUSG00000025867	Cplx2	0.028824062
ENSMUSG00000048200	Cracr2b	0.006085424
ENSMUSG00000020038	Cry1	0.000252129
ENSMUSG00000068742	Cry2	0.002231251
ENSMUSG00000032515	Csrnp1	0.016916742
ENSMUSG00000031778	Cx3cl1	0.019243424

ENSMUSG00000052336	Cx3cr1	0.028824062
ENSMUSG00000019590	Cyb561	0.025495919
ENSMUSG00000020810	Cygb	0.000297339
ENSMUSG00000026170	Cyp27a1	0.022168781
ENSMUSG00000028713	Cyp4b1	0.028824062
ENSMUSG00000062563	Cys1	1.38972E-05
ENSMUSG00000034574	Daam1	0.028824062
ENSMUSG00000040260	Daam2	0.033071808
ENSMUSG00000059824	Dbp	1.49841E-06
ENSMUSG00000038506	Dcun1d2	0.044228069
ENSMUSG00000054499	Dedd2	0.016916742
ENSMUSG00000031024	Denn2b	0.003754435
ENSMUSG00000030313	Dennd5b	0.033071808
ENSMUSG00000028974	Dffa	0.014692162
ENSMUSG00000030747	Dgat2	0.033071808
ENSMUSG00000034926	Dhcr24	0.033071808
ENSMUSG00000045414	Dipk2a	0.006085424
ENSMUSG00000040631	Dok4	0.019243424
ENSMUSG00000044716	Dok7	0.022168781
ENSMUSG00000025478	Dpysl4	0.028824062
ENSMUSG00000039982	Dtx4	0.03848718
ENSMUSG00000037887	Dusp8	0.025495919
ENSMUSG00000002409	Dyrk1b	0.007203
ENSMUSG00000028630	Dyrk2	0.006085424
ENSMUSG00000031616	Ednra	0.016916742
ENSMUSG00000035064	Eef2k	0.008381805
ENSMUSG00000036611	Eepd1	0.003214659
ENSMUSG00000026255	Efhd1	0.016916742
ENSMUSG00000040659	Efhd2	0.025495919
ENSMUSG00000031217	Efnb1	0.016916742
ENSMUSG00000020658	Efr3b	0.006085424
ENSMUSG00000024772	Ehd1	0.004475787
ENSMUSG00000031490	Eif4ebp1	0.004475787
ENSMUSG00000028760	Eif4g3	0.009968119
ENSMUSG00000008398	Elk3	0.001250287
ENSMUSG00000027131	Emc4	0.014692162
ENSMUSG00000040811	Eml2	0.033071808
ENSMUSG00000035401	Emsy	0.03848718
ENSMUSG00000026814	Eng	0.028824062
ENSMUSG00000045237	Eola1	0.025495919
ENSMUSG00000026235	Epha4	0.001064763
ENSMUSG00000038776	Ephx1	0.000103518
ENSMUSG00000028343	Erp44	0.007203
ENSMUSG00000032035	Ets1	0.006085424
ENSMUSG00000009079	Ewsr1	0.005252394
ENSMUSG00000028128	F3	0.016916742
ENSMUSG00000025384	Faap100	0.007203
ENSMUSG00000021750	Fam107a	0.003754435
ENSMUSG00000043230	Fam124b	0.028153568
ENSMUSG00000057497	Fam136a	0.044228069
ENSMUSG00000021234	Fam161b	0.011575813
ENSMUSG00000078670	Fam174b	0.033071808
ENSMUSG00000042595	Fam199x	0.016916742
ENSMUSG00000034858	Fam214a	0.006085424
ENSMUSG00000041930	Fam222a	0.000376217
ENSMUSG00000046546	Fam43a	8.32808E-05
ENSMUSG00000028878	Fam76a	0.001542731
ENSMUSG00000027204	Fbn1	8.32808E-05
ENSMUSG00000022358	Fbxo32	0.033071808
ENSMUSG00000047746	Fbxo40	0.014692162
ENSMUSG00000051000	Fhip1a	0.006085424
ENSMUSG00000074971	Fibin	0.013091819
ENSMUSG00000024222	Fkbp5	0.028824062

ENSMUSG00000028414	Fktn	0.013091819
ENSMUSG00000029648	Flt1	0.000103518
ENSMUSG00000040181	Fmo1	0.008381805
ENSMUSG00000040170	Fmo2	0.013091819
ENSMUSG00000003154	Foxj2	0.005252394
ENSMUSG00000048756	Foxo3	0.002231251
ENSMUSG00000074676	Foxs1	0.000449642
ENSMUSG00000056602	Fry	0.006085424
ENSMUSG00000029581	Fscn1	0.001064763
ENSMUSG00000035473	Galm	0.016916742
ENSMUSG00000021944	Gata4	0.014692162
ENSMUSG00000050953	Gja1	3.6994E-06
ENSMUSG00000026473	Glul	0.033071808
ENSMUSG00000113555	Gm10095	0.025495919
ENSMUSG00000086881	Gm13594	0.033071808
ENSMUSG00000084839	Gm14097	0.011575813
ENSMUSG00000085705	Gm16046	0.033071808
ENSMUSG00000091968	Gm17115	0.028153568
ENSMUSG00000093672	Gm20655	0.019243424
ENSMUSG00000097296	Gm26532	0.019243424
ENSMUSG00000098708	Gm27252	0.022168781
ENSMUSG00000109587	Gm31105	0.019243424
ENSMUSG00000112831	Gm35533	0.033071808
ENSMUSG00000092563	Gm3617	0.000717948
ENSMUSG00000104871	Gm42639	0.002231251
ENSMUSG00000109904	Gm45819	0.001250287
ENSMUSG00000114052	Gm48616	0.016916742
ENSMUSG00000025936	Gm4956	0.008381805
ENSMUSG00000027346	Gpcpd1	0.013091819
ENSMUSG00000044197	Gpr146	0.000449642
ENSMUSG00000026930	Gpsm1	0.033071808
ENSMUSG00000001700	Gramd3	0.044228069
ENSMUSG00000035900	Gramd4	0.000449642
ENSMUSG00000026888	Grb14	0.006085424
ENSMUSG00000030098	Grip2	0.014692162
ENSMUSG00000025934	Gsta3	7.05E-08
ENSMUSG00000033318	Gstt2	0.000873619
ENSMUSG00000053835	H2-T24	0.013091819
ENSMUSG00000026313	Hdac4	0.008381805
ENSMUSG00000031770	Herpud1	0.001064763
ENSMUSG00000022528	Hes1	0.016916742
ENSMUSG00000021260	Hhipl1	0.009968119
ENSMUSG00000029328	Hnrnpdl	0.000162335
ENSMUSG00000007617	Homer1	0.000873619
ENSMUSG00000034525	Ice1	0.000126318
ENSMUSG00000000732	Icosl	0.028824062
ENSMUSG00000025491	Ifitm1	0.000717948
ENSMUSG00000033581	Igf2bp2	0.002231251
ENSMUSG00000037679	Inf2	0.044228069
ENSMUSG00000037940	Inpp4b	0.028824062
ENSMUSG00000032599	Ip6k2	0.004475787
ENSMUSG00000030662	Ipo5	0.044228069
ENSMUSG00000060477	Irak2	0.025495919
ENSMUSG00000027111	Itga6	0.019243424
ENSMUSG00000026971	Itgb6	0.000449642
ENSMUSG00000052837	Junb	0.019243424
ENSMUSG00000042099	Kank3	0.005252394
ENSMUSG00000033182	Kbtbd12	0.000162335
ENSMUSG00000038319	Kcnh2	0.002231251
ENSMUSG00000062960	Kdr	1.80897E-05
ENSMUSG00000033863	Klf9	0.008381805
ENSMUSG00000042115	Klhdc8a	5.78314E-05
ENSMUSG00000026308	Klhl30	7.83794E-05

ENSMUSG00000090799	Klhl33	0.005252394
ENSMUSG00000035606	Ky	0.019243424
ENSMUSG00000033499	Larp4b	0.03848718
ENSMUSG00000024063	Lbh	0.014692162
ENSMUSG00000038793	Lefty1	0.016357821
ENSMUSG00000042487	Leo1	5.78314E-05
ENSMUSG00000042793	Lgr6	0.005252394
ENSMUSG00000048332	Lhfp	0.033071808
ENSMUSG00000024395	Lims2	0.016916742
ENSMUSG00000051067	Lingo3	0.002686921
ENSMUSG00000039633	Lonrf1	0.009968119
ENSMUSG00000072568	Lratd2	0.028824062
ENSMUSG00000045045	Lrfn4	0.028824062
ENSMUSG00000032352	Lrrc1	0.001250287
ENSMUSG00000060187	Lrrc10	0.013091819
ENSMUSG00000052316	Lrrc15	0.033071808
ENSMUSG00000007476	Lrrc8a	0.011575813
ENSMUSG00000070639	Lrrc8b	0.016916742
ENSMUSG00000026305	Lrrfip1	0.004475787
ENSMUSG00000032497	Lrrfip2	0.033071808
ENSMUSG00000020863	Luc7l3	0.03848718
ENSMUSG00000079018	Ly6c1	0.000717948
ENSMUSG00000030787	Lyve1	0.000873619
ENSMUSG00000036306	Lzts1	0.025495919
ENSMUSG00000028649	Macf1	0.019243424
ENSMUSG00000028826	Maco1	0.004475787
ENSMUSG00000037306	Man1c1	0.019243424
ENSMUSG00000032295	Man2c1	0.025495919
ENSMUSG00000031812	Map1lc3b	0.028824062
ENSMUSG00000028862	Map3k6	0.044228069
ENSMUSG00000069662	Marcks	0.000200016
ENSMUSG00000003810	Mast2	0.011575813
ENSMUSG00000032135	Mcam	0.025495919
ENSMUSG00000031442	Mcf2l	0.019243424
ENSMUSG00000036718	Micall2	0.03848718
ENSMUSG00000008035	Mid1ip1	0.000126318
ENSMUSG00000026858	Miga2	0.003214659
ENSMUSG00000035158	Mitf	0.013091819
ENSMUSG00000020190	Mknk2	0.004475787
ENSMUSG00000000901	Mmp11	0.003754435
ENSMUSG00000000957	Mmp14	0.007203
ENSMUSG00000031790	Mmp15	0.022168781
ENSMUSG00000005417	Mprip	0.011575813
ENSMUSG00000026566	Mpzl1	0.005252394
ENSMUSG00000040675	Mthfd1l	0.000376217
ENSMUSG00000029009	Mthfr	0.001250287
ENSMUSG00000045636	Mtus1	0.022168781
ENSMUSG00000025141	Myadml2	0.014692162
ENSMUSG00000037169	Mycn	0.000200016
ENSMUSG00000022443	Myh9	0.001869435
ENSMUSG00000038175	Mylip	0.008381805
ENSMUSG00000005982	Naa60	0.013091819
ENSMUSG00000025402	Nab2	0.028824062
ENSMUSG00000009418	Nav1	0.019243424
ENSMUSG00000020647	Ncoa1	0.005252394
ENSMUSG00000021365	Nedd9	0.005252394
ENSMUSG00000056749	Nfil3	3.6994E-06
ENSMUSG00000021025	Nfkbia	0.03848718
ENSMUSG00000039835	Nhsl1	0.025495919
ENSMUSG00000037966	Ninj1	0.007203
ENSMUSG00000020936	Nmt1	0.044228069
ENSMUSG00000026077	Npas2	0.000297339
ENSMUSG00000020889	Nr1d1	9.23E-08

ENSMUSG00000021775	Nr1d2	4.88E-07
ENSMUSG00000021179	Nrde2	0.014692162
ENSMUSG00000020019	Ntn4	0.013091819
ENSMUSG00000109511	Nup62	0.001542731
ENSMUSG00000023068	Nus1	0.013091819
ENSMUSG00000010097	Nxf1	0.008381805
ENSMUSG00000039747	Orai2	0.033071808
ENSMUSG00000038534	Osbpl7	0.025495919
ENSMUSG00000027765	P2ry1	0.019243424
ENSMUSG00000019916	P4ha1	0.014692162
ENSMUSG00000016664	Pacsin2	1.00402E-05
ENSMUSG00000020092	Pald1	0.013091819
ENSMUSG00000035863	Palm	0.000449642
ENSMUSG00000033377	Palmd	0.009968119
ENSMUSG00000021112	Pals1	0.022168781
ENSMUSG00000022685	Parn	0.003214659
ENSMUSG00000020453	Patz1	0.014692162
ENSMUSG00000047617	Paxx	0.025495919
ENSMUSG00000024440	Pcdh12	0.013091819
ENSMUSG00000033623	Pcgf3	0.022168781
ENSMUSG00000024975	Pdcd4	0.03848718
ENSMUSG00000032177	Pde4a	0.008381805
ENSMUSG00000028525	Pde4b	0.028824062
ENSMUSG00000063931	Pepd	0.033071808
ENSMUSG00000020893	Per1	0.000297339
ENSMUSG00000055866	Per2	8.32808E-05
ENSMUSG00000028957	Per3	2.14E-07
ENSMUSG00000026773	Pfkfb3	0.022168781
ENSMUSG00000011752	Pgam1	0.016916742
ENSMUSG00000030729	Pgm2l1	0.003754435
ENSMUSG00000028796	Phc2	0.000449642
ENSMUSG00000048537	Phldb1	0.016916742
ENSMUSG00000034614	Pik3ip1	0.019243424
ENSMUSG00000041417	Pik3r1	0.016916742
ENSMUSG00000035828	Pim3	0.000252129
ENSMUSG00000028221	Pip4p2	0.044228069
ENSMUSG00000050721	Plekho2	0.03848718
ENSMUSG00000024197	Plin3	0.03848718
ENSMUSG00000002831	Plin4	0.001250287
ENSMUSG00000028517	Plpp3	0.005252394
ENSMUSG00000030123	Plxnd1	0.002231251
ENSMUSG00000022803	Popdc2	0.033071808
ENSMUSG00000005514	Por	0.022168781
ENSMUSG00000029246	Ppat	0.014692162
ENSMUSG00000016487	Ppfibp1	0.044228069
ENSMUSG00000039457	Ppl	0.001869435
ENSMUSG00000042717	Ppp1r3a	0.013091819
ENSMUSG00000026626	Ppp2r5a	0.009968119
ENSMUSG00000017843	Ppp2r5c	0.014692162
ENSMUSG00000057637	Prdm2	0.025495919
ENSMUSG00000031145	Prickle3	0.044228069
ENSMUSG00000029513	Prkab1	0.014692162
ENSMUSG00000003526	Prodh	0.002686921
ENSMUSG00000010175	Prox1	0.025495919
ENSMUSG00000024735	Prpf19	0.002686921
ENSMUSG00000015476	Prrt1	0.03848718
ENSMUSG00000017969	Ptgis	0.006085424
ENSMUSG00000022607	Ptk2	0.011575813
ENSMUSG00000059456	Ptk2b	0.007203
ENSMUSG00000059895	Ptp4a3	0.044228069
ENSMUSG00000026384	Ptpn4	0.000583768
ENSMUSG00000020156	Pwwp3a	0.004475787
ENSMUSG00000020674	Pxdn	0.002686921

ENSMUSG00000034910	Pygo1	0.001869435
ENSMUSG00000056515	Rab31	0.008381805
ENSMUSG00000002059	Rab34	0.014692162
ENSMUSG00000064181	Rab3ip	0.028824062
ENSMUSG00000038546	Ranbp9	0.025495919
ENSMUSG00000068798	Rap1a	0.003754435
ENSMUSG00000037896	Rcor1	0.003754435
ENSMUSG00000020275	Rel	0.005252394
ENSMUSG00000002983	Relb	0.013091819
ENSMUSG00000029363	Rfc5	0.03848718
ENSMUSG00000020846	Rflnb	8.32808E-05
ENSMUSG00000017692	Rhbdl3	0.028824062
ENSMUSG00000019944	Rhobtb1	8.32808E-05
ENSMUSG00000046768	Rhoj	0.007203
ENSMUSG00000039960	Rhou	0.044228069
ENSMUSG00000051735	Rinl	0.022168781
ENSMUSG00000020642	Rnf144a	0.011575813
ENSMUSG00000058498	Rnf207	0.005252394
ENSMUSG00000028274	Rngtt	0.033071808
ENSMUSG00000028150	Rorc	0.000717948
ENSMUSG00000027651	Rprd1b	0.016916742
ENSMUSG00000063171	Rps4l	0.03848718
ENSMUSG00000032044	Rpusd4	0.025495919
ENSMUSG00000038387	Rras	0.007203
ENSMUSG00000055723	Rras2	0.025495919
ENSMUSG00000020088	Sar1a	0.022168781
ENSMUSG00000015305	Sash1	0.022168781
ENSMUSG00000042978	Sbk1	0.000873619
ENSMUSG00000027200	Sema6d	0.007203
ENSMUSG00000038264	Sema7a	0.013091819
ENSMUSG00000045827	Serpinb9	0.002686921
ENSMUSG00000037411	Serpine1	0.044228069
ENSMUSG00000027834	Serpini1	0.028824062
ENSMUSG00000038332	Sesn1	0.044228069
ENSMUSG00000025982	Sf3b1	0.03848718
ENSMUSG00000021892	Sh3bp5	0.014692162
ENSMUSG00000044991	Shld1	0.002231251
ENSMUSG00000034908	Sidt2	0.019243424
ENSMUSG00000025138	Sirt7	0.022168781
ENSMUSG00000029321	Slc10a6	0.022168781
ENSMUSG00000017765	Slc12a4	0.000126318
ENSMUSG00000017756	Slc12a7	0.022168781
ENSMUSG00000001918	Slc1a5	0.008381805
ENSMUSG00000028982	Slc25a33	0.001064763
ENSMUSG00000024259	Slc25a46	0.028824062
ENSMUSG00000066150	Slc31a1	0.044228069
ENSMUSG00000066152	Slc31a2	0.011575813
ENSMUSG00000031156	Slc35a2	0.044228069
ENSMUSG00000022094	Slc39a14	0.03848718
ENSMUSG00000030089	Slc41a3	1.80897E-05
ENSMUSG00000027074	Slc43a3	0.009968119
ENSMUSG00000057193	Slc44a2	0.003754435
ENSMUSG00000026435	Slc45a3	0.03848718
ENSMUSG00000029650	Slc46a3	0.004475787
ENSMUSG00000026062	Slc9a2	0.03848718
ENSMUSG00000025790	Slco3a1	0.019243424
ENSMUSG00000025060	Slk	0.044228069
ENSMUSG00000021870	Slmap	0.028824062
ENSMUSG00000075408	Smim41	0.044228069
ENSMUSG00000027488	Snta1	0.016916742
ENSMUSG00000025006	Sorbs1	0.025495919
ENSMUSG00000076431	Sox4	0.013091819
ENSMUSG00000029723	Spacdr	0.008381805

ENSMUSG00000021990	Spata13	0.011575813
ENSMUSG00000037379	Spon2	0.000297339
ENSMUSG00000026532	Spta1	0.007203
ENSMUSG00000057738	Sptan1	0.014692162
ENSMUSG00000015837	Sqstm1	0.014692162
ENSMUSG00000028676	Srsf10	0.014692162
ENSMUSG00000021134	Srsf5	0.001869435
ENSMUSG00000003992	Ssbp2	0.028824062
ENSMUSG00000037926	Ssh2	0.019243424
ENSMUSG00000018167	Stard3	0.000200016
ENSMUSG00000012428	Steap4	0.000873619
ENSMUSG00000063410	Stk24	0.001064763
ENSMUSG00000053963	Stum	0.005252394
ENSMUSG00000011306	Sugp1	0.003754435
ENSMUSG00000016918	Sulf1	0.019243424
ENSMUSG00000030711	Sult1a1	0.009968119
ENSMUSG00000023805	Synj2	0.008381805
ENSMUSG00000050315	Synpo2	0.013091819
ENSMUSG00000030852	Tacc2	0.044228069
ENSMUSG00000031832	Taf1c	0.03848718
ENSMUSG00000038520	Tbc1d17	0.000103518
ENSMUSG00000038517	Tbkbp1	0.03848718
ENSMUSG00000007877	Tcap	2.08244E-06
ENSMUSG00000020034	Tcp11l2	0.014692162
ENSMUSG00000022389	Tef	7.22416E-06
ENSMUSG00000006386	Tek	0.028824062
ENSMUSG00000032265	Tent5a	0.03848718
ENSMUSG00000046694	Tent5b	0.033071808
ENSMUSG00000032411	Tfdp2	0.022168781
ENSMUSG00000022797	Tfrc	0.001064763
ENSMUSG00000074743	Thbd	0.028824062
ENSMUSG00000028145	Them4	0.019243424
ENSMUSG00000058756	Thra	0.014692162
ENSMUSG00000030846	Tial1	0.013091819
ENSMUSG00000020044	Timp3	0.019243424
ENSMUSG00000030317	Timp4	0.019243424
ENSMUSG00000028776	Tinagl1	0.008381805
ENSMUSG00000070462	Tlnrd1	0.000162335
ENSMUSG00000024736	Tmem132a	0.044228069
ENSMUSG00000052485	Tmem171	5.78314E-05
ENSMUSG00000023153	Tmem52	0.011575813
ENSMUSG00000010307	Tmem86a	0.000200016
ENSMUSG00000030306	Tmtc1	0.005252394
ENSMUSG00000021281	Tnfaip2	8.32808E-05
ENSMUSG00000044469	Tnfaip8l1	0.002231251
ENSMUSG00000027692	Tnik	0.014692162
ENSMUSG00000037003	Tns2	0.013091819
ENSMUSG00000048546	Tob2	0.013091819
ENSMUSG00000070544	Top1	8.32808E-05
ENSMUSG00000032501	Trib1	0.011575813
ENSMUSG00000028834	Trim63	0.014692162
ENSMUSG00000040350	Trim7	0.016916742
ENSMUSG00000019487	Trip10	0.002686921
ENSMUSG00000001909	Trmt1	0.006085424
ENSMUSG00000059552	Trp53	0.009968119
ENSMUSG00000068735	Trp53i11	0.001869435
ENSMUSG00000031431	Tsc22d3	0.013091819
ENSMUSG00000037031	Tspan15	0.006085424
ENSMUSG00000027217	Tspan18	0.004475787
ENSMUSG00000025511	Tspan4	2.48992E-05
ENSMUSG00000019505	Ubb	0.016916742
ENSMUSG00000058317	Ube2e2	0.028824062
ENSMUSG00000020794	Ube2g1	0.03848718

ENSMUSG00000023977	Ubr2	0.011575813
ENSMUSG00000046562	Unc119b	0.006085424
ENSMUSG00000032010	Usp2	0.000126318
ENSMUSG00000034993	Vat1	0.044228069
ENSMUSG00000023951	Vegfa	0.025495919
ENSMUSG00000050666	Vstm4	0.044228069
ENSMUSG00000030889	Vwa3a	0.003214659
ENSMUSG00000066357	Wdr6	0.022168781
ENSMUSG00000020257	Wdr82	0.025495919
ENSMUSG00000031016	Wee1	0.000297339
ENSMUSG00000086040	Wipf3	0.000103518
ENSMUSG00000035112	Wnk4	0.003754435
ENSMUSG00000030170	Wnt5b	0.044228069
ENSMUSG00000017677	Wsb1	0.002686921
ENSMUSG00000024066	Xdh	0.000449642
ENSMUSG00000018427	Ypel2	3.41078E-05
ENSMUSG00000066687	Zbtb16	0.000297339
ENSMUSG00000063659	Zbtb18	0.013091819
ENSMUSG00000047473	Zfp30	0.044228069
ENSMUSG00000057894	Zfp329	0.022168781
ENSMUSG00000021127	Zfp36l1	0.022168781
ENSMUSG00000024220	Zfp523	0.007203
ENSMUSG00000022201	Zfr	0.003214659
ENSMUSG00000053080	Zfta	0.044228069
ENSMUSG00000032264	Zw10	0.003214659

Supplementary Table 5. JTK cycle analysis identifies transcripts that oscillate with a periodicity of 24hrs in CON hearts. Tissues were isolated from mice at distinct times of the day, followed by assessment of gene expression by RNAseq. 5 mice per experimental group.

Gene ID	Gene Symbol	Goodness of Fit q-value
ENSMUSG00000031983	2310022B05Rik	0.044912331
ENSMUSG00000006403	Adamts4	0.001404506
ENSMUSG00000053279	Aldh1a1	0.033633529
ENSMUSG00000026687	Aldh9a1	0.029411426
ENSMUSG00000013076	Amotl1	0.009491618
ENSMUSG00000028989	Angptl7	0.009491618
ENSMUSG00000055116	Arntl	2.11054E-05
ENSMUSG00000038539	Atf5	0.044912331
ENSMUSG00000046605	B3gntl1	0.02082025
ENSMUSG00000020385	Clk4	0.039726769
ENSMUSG00000004098	Col5a3	0.008367725
ENSMUSG00000020038	Cry1	2.01484E-05
ENSMUSG00000068742	Cry2	0.02082025
ENSMUSG00000062563	Cys1	0.000127466
ENSMUSG00000059824	Dbp	0.000479002
ENSMUSG00000043671	Dpy19l3	0.044912331
ENSMUSG00000038776	Ephx1	0.011412824
ENSMUSG00000040170	Fmo2	0.011412824
ENSMUSG00000074676	Foxs1	0.000479002
ENSMUSG00000109904	Gm45819	0.033633529
ENSMUSG00000025934	Gsta3	0.000160216
ENSMUSG00000045440	Insm2	0.039726769
ENSMUSG00000042487	Leo1	0.017263847
ENSMUSG00000038046	Mrm3	0.029411426
ENSMUSG00000056749	Nfil3	0.014335374
ENSMUSG00000026077	Npas2	0.004982862
ENSMUSG00000020889	Nr1d1	1.57391E-05
ENSMUSG00000021775	Nr1d2	0.003939203
ENSMUSG00000055866	Per2	0.000421238
ENSMUSG00000028957	Per3	0.000103415
ENSMUSG00000031538	Plat	0.001404506
ENSMUSG00000028600	Podn	0.044912331
ENSMUSG00000022951	Rcan1	3.77963E-05
ENSMUSG00000046768	Rhoj	0.044912331
ENSMUSG00000020642	Rnf144a	0.017263847
ENSMUSG00000037434	Slc30a1	0.029411426
ENSMUSG00000027074	Slc43a3	0.009491618
ENSMUSG00000025790	Slco3a1	0.00310932
ENSMUSG00000037379	Spon2	0.001404506
ENSMUSG00000022389	Tef	0.033633529
ENSMUSG00000027800	Tm4sf1	0.00310932
ENSMUSG00000025511	Tspan4	0.039726769
ENSMUSG00000046562	Unc119b	0.033633529
ENSMUSG00000031016	Wee1	0.029411426

Supplementary Table 6. JTK cycle analysis identifies transcripts that oscillate with a periodicity of 24hrs in dnOGA hearts. Tissues were isolated from mice at distinct times of the day, followed by assessment of gene expression by RNAseq. 5 mice per experimental group.

Gene ID	Gene Symbol	Goodness of Fit q-value
ENSMUSG00000090066	1110002E22Rik	0.013091819
ENSMUSG00000099411	2310015D24Rik	0.003754435
ENSMUSG00000045411	2410002F23Rik	0.014692162
ENSMUSG00000099696	2900052N01Rik	0.033071808
ENSMUSG00000071540	3425401B19Rik	0.002231251
ENSMUSG00000086938	4930481A15Rik	0.006085424
ENSMUSG00000086607	4930511M06Rik	0.011575813
ENSMUSG00000046138	9930021J03Rik	0.025495919
ENSMUSG00000029408	Abcb9	0.019243424
ENSMUSG00000029095	Ablim2	0.000376217
ENSMUSG00000042895	Abra	0.022168781
ENSMUSG00000030083	Abtb1	0.013091819
ENSMUSG00000038007	Acer2	0.001869435
ENSMUSG00000034853	Acot11	0.028824062
ENSMUSG00000029580	Actb	0.004475787
ENSMUSG00000062825	Actg1	0.008381805
ENSMUSG00000054693	Adam10	0.019243424
ENSMUSG00000011256	Adam19	0.000200016
ENSMUSG00000025026	Add3	0.044228069
ENSMUSG00000039167	Adgrl4	0.033071808
ENSMUSG00000074207	Adh1	8.32808E-05
ENSMUSG00000039041	Adrm1	0.028824062
ENSMUSG00000033032	Afap1l1	0.001542731
ENSMUSG00000025422	Agap2	0.004475787
ENSMUSG00000049115	Agtr1a	0.008381805
ENSMUSG00000069833	Ahnak	0.001542731
ENSMUSG00000047423	AI837181	0.016916742
ENSMUSG00000018428	Akap1	0.000376217
ENSMUSG00000021057	Akap5	0.001869435
ENSMUSG00000075232	Amd1	0.001250287
ENSMUSG00000036820	Amdhd2	0.011575813
ENSMUSG00000027889	Ampd2	0.008381805
ENSMUSG00000036977	Anapc10	0.014692162
ENSMUSG00000004105	Angptl2	0.001250287
ENSMUSG00000004849	Ap1s1	0.028824062
ENSMUSG00000044338	Aplnr	0.001542731
ENSMUSG00000050014	Apol10b	0.022168781
ENSMUSG00000004655	Aqp1	0.016916742
ENSMUSG00000028427	Aqp7	0.007203
ENSMUSG00000037999	Arap2	0.001064763
ENSMUSG00000039031	Arhgap18	0.001542731
ENSMUSG00000039831	Arhgap29	0.002686921
ENSMUSG00000025132	Arhgdia	0.033071808
ENSMUSG00000040964	Arhgef10l	0.005252394
ENSMUSG00000059495	Arhgef12	0.019243424
ENSMUSG00000028919	Arhgef19	0.033071808
ENSMUSG00000027599	Armc1	0.033071808
ENSMUSG00000002910	Arrdc2	0.000717948
ENSMUSG00000030996	Art1	0.000252129
ENSMUSG00000070424	Art5	0.011575813
ENSMUSG00000021200	Asb2	0.022168781
ENSMUSG00000028207	Asph	0.014692162
ENSMUSG00000026663	Atf6	0.000126318
ENSMUSG00000029673	Auts2	0.000873619
ENSMUSG00000075010	AW112010	0.025495919
ENSMUSG00000025372	Baiap2	0.001250287
ENSMUSG00000002748	Baz1b	0.006085424
ENSMUSG00000007659	Bcl2l1	0.001869435
ENSMUSG00000000317	Bcl6b	0.003214659
ENSMUSG00000030103	Bhlhe40	0.000717948
ENSMUSG00000030256	Bhlhe41	0.000583768
ENSMUSG00000016758	Bik	0.008381805

ENSMUSG00000024335	Brd2	0.019243424
ENSMUSG00000029544	Cabp1	0.009968119
ENSMUSG00000046447	Camk2n1	0.025495919
ENSMUSG00000024942	Capn1	0.028824062
ENSMUSG00000001794	Capns1	0.013091819
ENSMUSG00000027184	Caprin1	0.009968119
ENSMUSG00000000805	Car4	0.009968119
ENSMUSG00000045954	Cavin2	0.025495919
ENSMUSG00000020074	Ccar1	0.003214659
ENSMUSG00000020925	Ccdc43	0.028824062
ENSMUSG00000095098	Ccdc85b	0.014692162
ENSMUSG00000029617	Ccz1	0.011575813
ENSMUSG00000025351	Cd63	0.025495919
ENSMUSG00000027435	Cd93	0.006085424
ENSMUSG00000027330	Cdc25b	0.014692162
ENSMUSG00000024780	Cdc37l1	0.003754435
ENSMUSG00000020015	Cdk17	0.011575813
ENSMUSG00000021483	Cdk20	0.033071808
ENSMUSG00000023067	Cdkn1a	0.028824062
ENSMUSG00000037443	Cep85	0.014692162
ENSMUSG00000027313	Chac1	0.006085424
ENSMUSG00000020309	Chac2	0.003754435
ENSMUSG00000057133	Chd6	0.019243424
ENSMUSG00000041235	Chd7	0.022168781
ENSMUSG00000000743	Chmp1a	0.028824062
ENSMUSG00000001774	Chordc1	0.000252129
ENSMUSG00000006958	Chrd	0.001250287
ENSMUSG00000038550	Ciart	0.001250287
ENSMUSG00000034157	Cipc	0.002231251
ENSMUSG00000045193	Cirbp	0.000297339
ENSMUSG00000046841	Ckap4	0.003214659
ENSMUSG00000064302	Clasp1	7.05E-08
ENSMUSG00000049550	Clip1	0.014692162
ENSMUSG00000063146	Clip2	0.016916742
ENSMUSG00000029238	Clock	1.08307E-06
ENSMUSG00000015357	Clpx	0.019243424
ENSMUSG00000022037	Clu	0.033071808
ENSMUSG00000031875	Cmtm3	0.011575813
ENSMUSG00000032434	Cmtm6	0.001250287
ENSMUSG00000028339	Col15a1	0.000297339
ENSMUSG00000001506	Col1a1	0.003754435
ENSMUSG00000026043	Col3a1	0.000873619
ENSMUSG00000031502	Col4a1	0.000449642
ENSMUSG00000031503	Col4a2	7.22416E-06
ENSMUSG00000026837	Col5a1	0.028824062
ENSMUSG00000001119	Col6a1	0.014692162
ENSMUSG00000020241	Col6a2	0.013091819
ENSMUSG00000057606	Colq	0.009968119
ENSMUSG00000025981	Coq10b	0.002686921
ENSMUSG00000026489	Coq8a	0.003754435
ENSMUSG00000025586	Cpeb1	0.007203
ENSMUSG00000025867	Cplx2	0.028824062
ENSMUSG00000048200	Cracr2b	0.006085424
ENSMUSG00000032515	Csrnp1	0.016916742
ENSMUSG00000031778	Cx3cl1	0.019243424
ENSMUSG00000052336	Cx3cr1	0.028824062
ENSMUSG00000019590	Cyb561	0.025495919
ENSMUSG00000020810	Cygb	0.000297339
ENSMUSG00000026170	Cyp27a1	0.022168781
ENSMUSG00000028713	Cyp4b1	0.028824062
ENSMUSG00000034574	Daam1	0.028824062
ENSMUSG00000040260	Daam2	0.033071808
ENSMUSG00000038506	Dcun1d2	0.044228069

ENSMUSG00000054499	Dedd2	0.016916742
ENSMUSG00000031024	Denn2b	0.003754435
ENSMUSG00000030313	Dennd5b	0.033071808
ENSMUSG00000028974	Dffa	0.014692162
ENSMUSG00000030747	Dgat2	0.033071808
ENSMUSG00000034926	Dhcr24	0.033071808
ENSMUSG00000045414	Dipk2a	0.006085424
ENSMUSG00000040631	Dok4	0.019243424
ENSMUSG00000044716	Dok7	0.022168781
ENSMUSG00000025478	Dpysl4	0.028824062
ENSMUSG00000039982	Dtx4	0.03848718
ENSMUSG00000037887	Dusp8	0.025495919
ENSMUSG00000002409	Dyrk1b	0.007203
ENSMUSG00000028630	Dyrk2	0.006085424
ENSMUSG00000031616	Ednra	0.016916742
ENSMUSG00000035064	Eef2k	0.008381805
ENSMUSG00000036611	Eepd1	0.003214659
ENSMUSG00000026255	Efhd1	0.016916742
ENSMUSG00000040659	Efhd2	0.025495919
ENSMUSG00000031217	Efnb1	0.016916742
ENSMUSG00000020658	Efr3b	0.006085424
ENSMUSG00000024772	Ehd1	0.004475787
ENSMUSG00000031490	Eif4ebp1	0.004475787
ENSMUSG00000028760	Eif4g3	0.009968119
ENSMUSG00000008398	Elk3	0.001250287
ENSMUSG00000027131	Emc4	0.014692162
ENSMUSG00000040811	Eml2	0.033071808
ENSMUSG00000035401	Emsy	0.03848718
ENSMUSG00000026814	Eng	0.028824062
ENSMUSG00000045237	Eola1	0.025495919
ENSMUSG00000026235	Epha4	0.001064763
ENSMUSG00000028343	Erp44	0.007203
ENSMUSG00000032035	Ets1	0.006085424
ENSMUSG00000009079	Ewsr1	0.005252394
ENSMUSG00000028128	F3	0.016916742
ENSMUSG00000025384	Faap100	0.007203
ENSMUSG00000021750	Fam107a	0.003754435
ENSMUSG00000043230	Fam124b	0.028153568
ENSMUSG00000057497	Fam136a	0.044228069
ENSMUSG00000021234	Fam161b	0.011575813
ENSMUSG00000078670	Fam174b	0.033071808
ENSMUSG00000042595	Fam199x	0.016916742
ENSMUSG00000034858	Fam214a	0.006085424
ENSMUSG00000041930	Fam222a	0.000376217
ENSMUSG00000046546	Fam43a	8.32808E-05
ENSMUSG00000028878	Fam76a	0.001542731
ENSMUSG00000027204	Fbn1	8.32808E-05
ENSMUSG00000022358	Fbxo32	0.033071808
ENSMUSG00000047746	Fbxo40	0.014692162
ENSMUSG00000051000	Fhip1a	0.006085424
ENSMUSG00000074971	Fibin	0.013091819
ENSMUSG00000024222	Fkbp5	0.028824062
ENSMUSG00000028414	Fktn	0.013091819
ENSMUSG00000029648	Flt1	0.000103518
ENSMUSG00000040181	Fmo1	0.008381805
ENSMUSG00000003154	Foxj2	0.005252394
ENSMUSG00000048756	Foxo3	0.002231251
ENSMUSG00000056602	Fry	0.006085424
ENSMUSG00000029581	Fscn1	0.001064763
ENSMUSG00000035473	Galm	0.016916742
ENSMUSG00000021944	Gata4	0.014692162
ENSMUSG00000050953	Gja1	3.6994E-06
ENSMUSG00000026473	Glul	0.033071808

ENSMUSG00000113555	Gm10095	0.025495919
ENSMUSG00000086881	Gm13594	0.033071808
ENSMUSG00000084839	Gm14097	0.011575813
ENSMUSG00000085705	Gm16046	0.033071808
ENSMUSG00000091968	Gm17115	0.028153568
ENSMUSG00000093672	Gm20655	0.019243424
ENSMUSG00000097296	Gm26532	0.019243424
ENSMUSG00000098708	Gm27252	0.022168781
ENSMUSG00000109587	Gm31105	0.019243424
ENSMUSG00000112831	Gm35533	0.033071808
ENSMUSG00000092563	Gm3617	0.000717948
ENSMUSG00000104871	Gm42639	0.002231251
ENSMUSG00000114052	Gm48616	0.016916742
ENSMUSG00000025936	Gm4956	0.008381805
ENSMUSG00000027346	Gpcpd1	0.013091819
ENSMUSG00000044197	Gpr146	0.000449642
ENSMUSG00000026930	Gpsm1	0.033071808
ENSMUSG00000001700	Gramd3	0.044228069
ENSMUSG00000035900	Gramd4	0.000449642
ENSMUSG00000026888	Grb14	0.006085424
ENSMUSG00000030098	Grip2	0.014692162
ENSMUSG00000033318	Gstt2	0.000873619
ENSMUSG00000053835	H2-T24	0.013091819
ENSMUSG00000026313	Hdac4	0.008381805
ENSMUSG00000031770	Herpud1	0.001064763
ENSMUSG00000022528	Hes1	0.016916742
ENSMUSG00000021260	Hhipl1	0.009968119
ENSMUSG00000029328	Hnrnpdl	0.000162335
ENSMUSG00000007617	Homer1	0.000873619
ENSMUSG00000034525	Ice1	0.000126318
ENSMUSG00000000732	Icosl	0.028824062
ENSMUSG00000025491	Ifitm1	0.000717948
ENSMUSG00000033581	Igf2bp2	0.002231251
ENSMUSG00000037679	Inf2	0.044228069
ENSMUSG00000037940	Inpp4b	0.028824062
ENSMUSG00000032599	Ip6k2	0.004475787
ENSMUSG00000030662	Ipo5	0.044228069
ENSMUSG00000060477	Irak2	0.025495919
ENSMUSG00000027111	Itga6	0.019243424
ENSMUSG00000026971	Itgb6	0.000449642
ENSMUSG00000052837	Junb	0.019243424
ENSMUSG00000042099	Kank3	0.005252394
ENSMUSG00000033182	Kbtbd12	0.000162335
ENSMUSG00000038319	Kcnh2	0.002231251
ENSMUSG00000062960	Kdr	1.80897E-05
ENSMUSG00000033863	Klf9	0.008381805
ENSMUSG00000042115	Klhdc8a	5.78314E-05
ENSMUSG00000026308	Klhl30	7.83794E-05
ENSMUSG00000090799	Klhl33	0.005252394
ENSMUSG00000035606	Ky	0.019243424
ENSMUSG00000033499	Larp4b	0.03848718
ENSMUSG00000024063	Lbh	0.014692162
ENSMUSG00000038793	Lefty1	0.016357821
ENSMUSG00000042793	Lgr6	0.005252394
ENSMUSG00000048332	Lhfp	0.033071808
ENSMUSG00000024395	Lims2	0.016916742
ENSMUSG00000051067	Lingo3	0.002686921
ENSMUSG00000039633	Lonrf1	0.009968119
ENSMUSG00000072568	Lratd2	0.028824062
ENSMUSG00000045045	Lrfn4	0.028824062
ENSMUSG00000032352	Lrrc1	0.001250287
ENSMUSG00000060187	Lrrc10	0.013091819
ENSMUSG00000052316	Lrrc15	0.033071808

ENSMUSG00000007476	Lrrc8a	0.011575813
ENSMUSG00000070639	Lrrc8b	0.016916742
ENSMUSG00000026305	Lrrfip1	0.004475787
ENSMUSG00000032497	Lrrfip2	0.033071808
ENSMUSG00000020863	Luc7l3	0.03848718
ENSMUSG00000079018	Ly6c1	0.000717948
ENSMUSG00000030787	Lyve1	0.000873619
ENSMUSG00000036306	Lzts1	0.025495919
ENSMUSG00000028649	Macf1	0.019243424
ENSMUSG00000028826	Maco1	0.004475787
ENSMUSG00000037306	Man1c1	0.019243424
ENSMUSG00000032295	Man2c1	0.025495919
ENSMUSG00000031812	Map1lc3b	0.028824062
ENSMUSG00000028862	Map3k6	0.044228069
ENSMUSG00000069662	Marcks	0.000200016
ENSMUSG00000003810	Mast2	0.011575813
ENSMUSG00000032135	Mcam	0.025495919
ENSMUSG00000031442	Mcf2l	0.019243424
ENSMUSG00000036718	Micall2	0.03848718
ENSMUSG00000008035	Mid1ip1	0.000126318
ENSMUSG00000026858	Miga2	0.003214659
ENSMUSG00000035158	Mitf	0.013091819
ENSMUSG00000020190	Mknk2	0.004475787
ENSMUSG00000000901	Mmp11	0.003754435
ENSMUSG00000000957	Mmp14	0.007203
ENSMUSG00000031790	Mmp15	0.022168781
ENSMUSG00000005417	Mprip	0.011575813
ENSMUSG00000026566	Mpzl1	0.005252394
ENSMUSG00000040675	Mthfd1l	0.000376217
ENSMUSG00000029009	Mthfr	0.001250287
ENSMUSG00000045636	Mtus1	0.022168781
ENSMUSG00000025141	Myadml2	0.014692162
ENSMUSG00000037169	Mycn	0.000200016
ENSMUSG00000022443	Myh9	0.001869435
ENSMUSG00000038175	Mylip	0.008381805
ENSMUSG00000005982	Naa60	0.013091819
ENSMUSG00000025402	Nab2	0.028824062
ENSMUSG00000009418	Nav1	0.019243424
ENSMUSG00000020647	Ncoa1	0.005252394
ENSMUSG00000021365	Nedd9	0.005252394
ENSMUSG00000021025	Nfkbia	0.03848718
ENSMUSG00000039835	Nhsl1	0.025495919
ENSMUSG00000037966	Ninj1	0.007203
ENSMUSG00000020936	Nmt1	0.044228069
ENSMUSG00000021179	Nrde2	0.014692162
ENSMUSG00000020019	Ntn4	0.013091819
ENSMUSG00000109511	Nup62	0.001542731
ENSMUSG00000023068	Nus1	0.013091819
ENSMUSG00000010097	Nxf1	0.008381805
ENSMUSG00000039747	Orai2	0.033071808
ENSMUSG00000038534	Osbpl7	0.025495919
ENSMUSG00000027765	P2ry1	0.019243424
ENSMUSG00000019916	P4ha1	0.014692162
ENSMUSG00000016664	Pacsin2	1.00402E-05
ENSMUSG00000020092	Pald1	0.013091819
ENSMUSG00000035863	Palm	0.000449642
ENSMUSG00000033377	Palmd	0.009968119
ENSMUSG00000021112	Pals1	0.022168781
ENSMUSG00000022685	Parn	0.003214659
ENSMUSG00000020453	Patz1	0.014692162
ENSMUSG00000047617	Paxx	0.025495919
ENSMUSG00000024440	Pcdh12	0.013091819
ENSMUSG00000033623	Pcgf3	0.022168781

ENSMUSG00000024975	Pdcd4	0.03848718
ENSMUSG00000032177	Pde4a	0.008381805
ENSMUSG00000028525	Pde4b	0.028824062
ENSMUSG00000063931	Pepd	0.033071808
ENSMUSG00000020893	Per1	0.000297339
ENSMUSG00000026773	Pfkfb3	0.022168781
ENSMUSG00000011752	Pgam1	0.016916742
ENSMUSG00000030729	Pgm2l1	0.003754435
ENSMUSG00000028796	Phc2	0.000449642
ENSMUSG00000048537	Phldb1	0.016916742
ENSMUSG00000034614	Pik3ip1	0.019243424
ENSMUSG00000041417	Pik3r1	0.016916742
ENSMUSG00000035828	Pim3	0.000252129
ENSMUSG00000028221	Pip4p2	0.044228069
ENSMUSG00000050721	Plekho2	0.03848718
ENSMUSG00000024197	Plin3	0.03848718
ENSMUSG00000002831	Plin4	0.001250287
ENSMUSG00000028517	Plpp3	0.005252394
ENSMUSG00000030123	Plxnd1	0.002231251
ENSMUSG00000022803	Popdc2	0.033071808
ENSMUSG00000005514	Por	0.022168781
ENSMUSG00000029246	Ppat	0.014692162
ENSMUSG00000016487	Ppfibp1	0.044228069
ENSMUSG00000039457	Ppl	0.001869435
ENSMUSG00000042717	Ppp1r3a	0.013091819
ENSMUSG00000026626	Ppp2r5a	0.009968119
ENSMUSG00000017843	Ppp2r5c	0.014692162
ENSMUSG00000057637	Prdm2	0.025495919
ENSMUSG00000031145	Prickle3	0.044228069
ENSMUSG00000029513	Prkab1	0.014692162
ENSMUSG00000003526	Prodh	0.002686921
ENSMUSG00000010175	Prox1	0.025495919
ENSMUSG00000024735	Prpf19	0.002686921
ENSMUSG00000015476	Prrt1	0.03848718
ENSMUSG00000017969	Ptgis	0.006085424
ENSMUSG00000022607	Ptk2	0.011575813
ENSMUSG00000059456	Ptk2b	0.007203
ENSMUSG00000059895	Ptp4a3	0.044228069
ENSMUSG00000026384	Ptpn4	0.000583768
ENSMUSG00000020156	Pwwp3a	0.004475787
ENSMUSG00000020674	Pxdn	0.002686921
ENSMUSG00000034910	Pygo1	0.001869435
ENSMUSG00000056515	Rab31	0.008381805
ENSMUSG00000002059	Rab34	0.014692162
ENSMUSG00000064181	Rab3ip	0.028824062
ENSMUSG00000038546	Ranbp9	0.025495919
ENSMUSG00000068798	Rap1a	0.003754435
ENSMUSG00000037896	Rcor1	0.003754435
ENSMUSG00000020275	Rel	0.005252394
ENSMUSG00000002983	Relb	0.013091819
ENSMUSG00000029363	Rfc5	0.03848718
ENSMUSG00000020846	Rflnb	8.32808E-05
ENSMUSG00000017692	Rhbdl3	0.028824062
ENSMUSG00000019944	Rhobtb1	8.32808E-05
ENSMUSG00000039960	Rhou	0.044228069
ENSMUSG00000051735	Rinl	0.022168781
ENSMUSG00000058498	Rnf207	0.005252394
ENSMUSG00000028274	Rngtt	0.033071808
ENSMUSG00000028150	Rorc	0.000717948
ENSMUSG00000027651	Rprd1b	0.016916742
ENSMUSG00000063171	Rps4l	0.03848718
ENSMUSG00000032044	Rpusd4	0.025495919
ENSMUSG00000038387	Rras	0.007203

ENSMUSG00000055723	Rras2	0.025495919
ENSMUSG00000020088	Sar1a	0.022168781
ENSMUSG00000015305	Sash1	0.022168781
ENSMUSG00000042978	Sbk1	0.000873619
ENSMUSG00000027200	Sema6d	0.007203
ENSMUSG00000038264	Sema7a	0.013091819
ENSMUSG00000045827	Serpinb9	0.002686921
ENSMUSG00000037411	Serpine1	0.044228069
ENSMUSG00000027834	Serpini1	0.028824062
ENSMUSG00000038332	Sesn1	0.044228069
ENSMUSG00000025982	Sf3b1	0.03848718
ENSMUSG00000021892	Sh3bp5	0.014692162
ENSMUSG00000044991	Shld1	0.002231251
ENSMUSG00000034908	Sidt2	0.019243424
ENSMUSG00000025138	Sirt7	0.022168781
ENSMUSG00000029321	Slc10a6	0.022168781
ENSMUSG00000017765	Slc12a4	0.000126318
ENSMUSG00000017756	Slc12a7	0.022168781
ENSMUSG00000001918	Slc1a5	0.008381805
ENSMUSG00000028982	Slc25a33	0.001064763
ENSMUSG00000024259	Slc25a46	0.028824062
ENSMUSG00000066150	Slc31a1	0.044228069
ENSMUSG00000066152	Slc31a2	0.011575813
ENSMUSG00000031156	Slc35a2	0.044228069
ENSMUSG00000022094	Slc39a14	0.03848718
ENSMUSG00000030089	Slc41a3	1.80897E-05
ENSMUSG00000057193	Slc44a2	0.003754435
ENSMUSG00000026435	Slc45a3	0.03848718
ENSMUSG00000029650	Slc46a3	0.004475787
ENSMUSG00000026062	Slc9a2	0.03848718
ENSMUSG00000025060	Slk	0.044228069
ENSMUSG00000021870	Slmap	0.028824062
ENSMUSG00000075408	Smim41	0.044228069
ENSMUSG00000027488	Snta1	0.016916742
ENSMUSG00000025006	Sorbs1	0.025495919
ENSMUSG00000076431	Sox4	0.013091819
ENSMUSG00000029723	Spacdr	0.008381805
ENSMUSG00000021990	Spata13	0.011575813
ENSMUSG00000026532	Spta1	0.007203
ENSMUSG00000057738	Sptan1	0.014692162
ENSMUSG00000015837	Sqstm1	0.014692162
ENSMUSG00000028676	Srsf10	0.014692162
ENSMUSG00000021134	Srsf5	0.001869435
ENSMUSG00000003992	Ssbp2	0.028824062
ENSMUSG00000037926	Ssh2	0.019243424
ENSMUSG00000018167	Stard3	0.000200016
ENSMUSG00000012428	Steap4	0.000873619
ENSMUSG00000063410	Stk24	0.001064763
ENSMUSG00000053963	Stum	0.005252394
ENSMUSG00000011306	Sugp1	0.003754435
ENSMUSG00000016918	Sulf1	0.019243424
ENSMUSG00000030711	Sult1a1	0.009968119
ENSMUSG00000023805	Synj2	0.008381805
ENSMUSG00000050315	Synpo2	0.013091819
ENSMUSG00000030852	Tacc2	0.044228069
ENSMUSG00000031832	Taf1c	0.03848718
ENSMUSG00000038520	Tbc1d17	0.000103518
ENSMUSG00000038517	Tbkbp1	0.03848718
ENSMUSG00000007877	Tcap	2.08244E-06
ENSMUSG00000020034	Tcp11l2	0.014692162
ENSMUSG00000006386	Tek	0.028824062
ENSMUSG00000032265	Tent5a	0.03848718
ENSMUSG00000046694	Tent5b	0.033071808

ENSMUSG00000032411	Tfdp2	0.022168781
ENSMUSG00000022797	Tfrc	0.001064763
ENSMUSG00000074743	Thbd	0.028824062
ENSMUSG00000028145	Them4	0.019243424
ENSMUSG00000058756	Thra	0.014692162
ENSMUSG00000030846	Tial1	0.013091819
ENSMUSG00000020044	Timp3	0.019243424
ENSMUSG00000030317	Timp4	0.019243424
ENSMUSG00000028776	Tinagl1	0.008381805
ENSMUSG00000070462	Tlnrd1	0.000162335
ENSMUSG00000024736	Tmem132a	0.044228069
ENSMUSG00000052485	Tmem171	5.78314E-05
ENSMUSG00000023153	Tmem52	0.011575813
ENSMUSG00000010307	Tmem86a	0.000200016
ENSMUSG00000030306	Tmtc1	0.005252394
ENSMUSG00000021281	Tnfaip2	8.32808E-05
ENSMUSG00000044469	Tnfaip8l1	0.002231251
ENSMUSG00000027692	Tnik	0.014692162
ENSMUSG00000037003	Tns2	0.013091819
ENSMUSG00000048546	Tob2	0.013091819
ENSMUSG00000070544	Top1	8.32808E-05
ENSMUSG00000032501	Trib1	0.011575813
ENSMUSG00000028834	Trim63	0.014692162
ENSMUSG00000040350	Trim7	0.016916742
ENSMUSG00000019487	Trip10	0.002686921
ENSMUSG00000001909	Trmt1	0.006085424
ENSMUSG00000059552	Trp53	0.009968119
ENSMUSG00000068735	Trp53i11	0.001869435
ENSMUSG00000031431	Tsc22d3	0.013091819
ENSMUSG00000037031	Tspan15	0.006085424
ENSMUSG00000027217	Tspan18	0.004475787
ENSMUSG00000019505	Ubb	0.016916742
ENSMUSG00000058317	Ube2e2	0.028824062
ENSMUSG00000020794	Ube2g1	0.03848718
ENSMUSG00000023977	Ubr2	0.011575813
ENSMUSG00000032010	Usp2	0.000126318
ENSMUSG00000034993	Vat1	0.044228069
ENSMUSG00000023951	Vegfa	0.025495919
ENSMUSG00000050666	Vstm4	0.044228069
ENSMUSG00000030889	Vwa3a	0.003214659
ENSMUSG00000066357	Wdr6	0.022168781
ENSMUSG00000020257	Wdr82	0.025495919
ENSMUSG00000086040	Wipf3	0.000103518
ENSMUSG00000035112	Wnk4	0.003754435
ENSMUSG00000030170	Wnt5b	0.044228069
ENSMUSG00000017677	Wsb1	0.002686921
ENSMUSG00000024066	Xdh	0.000449642
ENSMUSG00000018427	Ypel2	3.41078E-05
ENSMUSG00000063659	Zbtb18	0.013091819
ENSMUSG00000047473	Zfp30	0.044228069
ENSMUSG00000057894	Zfp329	0.022168781
ENSMUSG00000021127	Zfp36l1	0.022168781
ENSMUSG00000024220	Zfp523	0.007203
ENSMUSG00000022201	Zfr	0.003214659
ENSMUSG00000053080	Zfta	0.044228069
ENSMUSG00000032264	Zw10	0.003214659

Supplementary Table 7. JTK cycle analysis identifies transcripts that oscillate with a periodicity of 24hrs only in CON hearts. Tissues were isolated from mice at distinct times of the day, followed by assessment of gene expression by RNAseq. 5 mice per experimental group.

Gene ID	Gene Symbol	Goodness of Fit q-value
ENSMUSG00000026687	Aldh9a1	0.029411426
ENSMUSG00000013076	Amotl1	0.009491618
ENSMUSG00000038539	Atf5	0.044912331
ENSMUSG00000046605	B3gntl1	0.02082025
ENSMUSG00000020385	Clk4	0.039726769
ENSMUSG00000043671	Dpy19l3	0.044912331
ENSMUSG00000045440	Insm2	0.039726769
ENSMUSG00000038046	Mrm3	0.029411426
ENSMUSG00000031538	Plat	0.001404506
ENSMUSG00000028600	Podn	0.044912331
ENSMUSG00000022951	Rcan1	3.77963E-05
ENSMUSG00000037434	Slc30a1	0.029411426
ENSMUSG00000027800	Tm4sf1	0.00310932

Supplementary Table 8. JTK cycle analysis identifies transcripts that oscillate with a periodicity of 24hrs only in dnOGAh hearts. Tissues were isolated from mice at distinct times of the day, followed by assessment of gene expression by RNAseq. 5 mice per experimental group.

Gene ID	Gene Symbol	Goodness of Fit q-value (CON)	Goodness of Fit q-value (dnOGA)
ENSMUSG00000020889	Nr1d1	9.23E-08	1.57391E-05
ENSMUSG00000059824	Dbp	1.49841E-06	0.000479002
ENSMUSG00000037379	Spon2	0.000297339	0.001404506
ENSMUSG00000022389	Tef	7.22416E-06	0.033633529
ENSMUSG00000006403	Adamts4	0.018806464	0.001404506
ENSMUSG00000021775	Nr1d2	4.88E-07	0.003939203
ENSMUSG00000066687	Zbtb16	0.000297339	0.025993822
ENSMUSG00000055116	Arntl	2.35E-09	2.11054E-05
ENSMUSG00000042487	Leo1	5.78314E-05	0.017263847
ENSMUSG00000028957	Per3	2.14E-07	0.000103415
ENSMUSG00000068742	Cry2	0.002231251	0.02082025
ENSMUSG00000056749	Nfil3	3.6994E-06	0.014335374
ENSMUSG00000025934	Gsta3	7.05E-08	0.000160216
ENSMUSG00000004098	Col5a3	3.6994E-06	0.008367725
ENSMUSG00000062563	Cys1	1.38972E-05	0.000127466
ENSMUSG00000025511	Tspan4	2.48992E-05	0.039726769
ENSMUSG00000053279	Aldh1a1	4.64985E-05	0.033633529
ENSMUSG00000055866	Per2	8.32808E-05	0.000421238
ENSMUSG00000038776	Ephx1	0.000103518	0.011412824
ENSMUSG00000020038	Cry1	0.000252129	2.01484E-05
ENSMUSG00000026077	Npas2	0.000297339	0.004982862
ENSMUSG00000031016	Wee1	0.000297339	0.029411426
ENSMUSG00000074676	Foxs1	0.000449642	0.000479002
ENSMUSG00000109904	Gm45819	0.001250287	0.033633529
ENSMUSG00000031983	2310022B05Rik	0.003214659	0.044912331
ENSMUSG00000046562	Unc119b	0.006085424	0.033633529
ENSMUSG00000046768	Rhoj	0.007203	0.044912331
ENSMUSG00000027074	Slc43a3	0.009968119	0.009491618
ENSMUSG00000020642	Rnf144a	0.011575813	0.017263847
ENSMUSG00000040170	Fmo2	0.013091819	0.011412824
ENSMUSG00000025790	Slco3a1	0.019243424	0.00310932
ENSMUSG00000028989	Angptl7	0.022168781	0.009491618

Supplementary Table 9. JTK cycle analysis identifies transcripts that oscillate with a periodicity of 24hrs in both CON and dnOGA hearts. Tissues were isolated from mice at distinct times of the day, followed by assessment of gene expression by RNAseq. 5 mice per experimental group.

Gene ID	Gene Symbol	Goodness of Fit q-value (CON)	Goodness of Fit q-value (dnOGAh)	Amplitude (CON)	Amplitude (dnOGAh)	Amplitude Difference (q-value)
ENSMUSG00000090066	1110002E22Rik	0.013091819	1	118.1995403	Not Rhythmic	Not Applicable
ENSMUSG00000099411	2310015D24Rik	0.003754435	1	20.73195216	Not Rhythmic	Not Applicable
ENSMUSG00000045411	2410002F23Rik	0.014692162	0.124032757	55.92643656	Not Rhythmic	Not Applicable
ENSMUSG00000099696	2900052N01Rik	0.033071808	1	6.43978138	Not Rhythmic	Not Applicable
ENSMUSG00000071540	3425401B19Rik	0.002231251	1	305.9303061	Not Rhythmic	Not Applicable
ENSMUSG00000086938	4930481A15Rik	0.006085424	1	122.0299247	Not Rhythmic	Not Applicable
ENSMUSG00000086607	4930511M06Rik	0.011575813	1	9.224265895	Not Rhythmic	Not Applicable
ENSMUSG00000046138	9930021J03Rik	0.025495919	1	75.28613866	Not Rhythmic	Not Applicable
ENSMUSG00000029408	Abcb9	0.019243424	1	53.75069054	Not Rhythmic	Not Applicable
ENSMUSG00000029095	Ablim2	0.000376217	1	64.65136839	Not Rhythmic	Not Applicable
ENSMUSG00000042895	Abra	0.022168781	0.729922185	163.1263121	Not Rhythmic	Not Applicable
ENSMUSG00000030083	Abtb1	0.013091819	1	48.16893049	Not Rhythmic	Not Applicable
ENSMUSG00000038007	Acer2	0.001869435	1	49.43687392	Not Rhythmic	Not Applicable
ENSMUSG00000034853	Acot11	0.028824062	1	55.03433522	Not Rhythmic	Not Applicable
ENSMUSG00000029580	Actb	0.004475787	1	1166.957123	Not Rhythmic	Not Applicable
ENSMUSG00000062825	Actg1	0.008381805	1	486.6837836	Not Rhythmic	Not Applicable
ENSMUSG00000054693	Adam10	0.019243424	1	76.73517904	Not Rhythmic	Not Applicable
ENSMUSG00000011256	Adam19	0.000200016	1	147.4440958	Not Rhythmic	Not Applicable
ENSMUSG00000006403	Adamts4	0.018806464	0.001404506	7.889223276	40.99548143	0.016654883
ENSMUSG00000025026	Add3	0.044228069	1	108.5643958	Not Rhythmic	Not Applicable
ENSMUSG00000039167	Adgrl4	0.033071808	1	138.8605626	Not Rhythmic	Not Applicable
ENSMUSG00000074207	Adh1	8.32808E-05	0.215049924	184.1103253	Not Rhythmic	Not Applicable
ENSMUSG00000039041	Adrm1	0.028824062	1	176.0084626	Not Rhythmic	Not Applicable
ENSMUSG00000033032	Afap1l1	0.001542731	1	218.6699679	Not Rhythmic	Not Applicable
ENSMUSG00000025422	Agap2	0.004475787	0.339122376	29.24543713	Not Rhythmic	Not Applicable
ENSMUSG00000049115	Agtr1a	0.008381805	1	105.9646239	Not Rhythmic	Not Applicable
ENSMUSG00000069833	Ahnak	0.001542731	1	565.597048	Not Rhythmic	Not Applicable
ENSMUSG00000047423	AI837181	0.016916742	1	115.8307829	Not Rhythmic	Not Applicable
ENSMUSG00000018428	Akap1	0.000376217	1	354.3320605	Not Rhythmic	Not Applicable
ENSMUSG00000021057	Akap5	0.001869435	1	38.77232061	Not Rhythmic	Not Applicable
ENSMUSG00000075232	Amd1	0.001250287	0.954282031	310.993804	Not Rhythmic	Not Applicable
ENSMUSG00000036820	Amdhd2	0.011575813	1	38.15662209	Not Rhythmic	Not Applicable
ENSMUSG00000027889	Ampd2	0.008381805	1	34.58797455	Not Rhythmic	Not Applicable

ENSMUSG00000036977	Anapc10	0.014692162	1	37.36143249	Not Rhythmic	Not Applicable
ENSMUSG00000004105	Angptl2	0.001250287	0.194700719	250.9684611	Not Rhythmic	Not Applicable
ENSMUSG00000004849	Ap1s1	0.028824062	1	85.01026202	Not Rhythmic	Not Applicable
ENSMUSG00000044338	Aplnr	0.001542731	0.215049924	269.765411	Not Rhythmic	Not Applicable
ENSMUSG00000050014	Apol10b	0.022168781	1	35.01877057	Not Rhythmic	Not Applicable
ENSMUSG00000004655	Aqp1	0.016916742	1	1563.314447	Not Rhythmic	Not Applicable
ENSMUSG00000028427	Aqp7	0.007203	1	101.0849438	Not Rhythmic	Not Applicable
ENSMUSG00000037999	Arap2	0.001064763	1	56.12962539	Not Rhythmic	Not Applicable
ENSMUSG00000039031	Arhgap18	0.001542731	0.694751079	85.27618899	Not Rhythmic	Not Applicable
ENSMUSG00000039831	Arhgap29	0.002686921	1	125.2385055	Not Rhythmic	Not Applicable
ENSMUSG00000025132	Arhgdia	0.033071808	1	156.1825698	Not Rhythmic	Not Applicable
ENSMUSG00000040964	Arhgef10l	0.005252394	1	177.9098688	Not Rhythmic	Not Applicable
ENSMUSG00000059495	Arhgef12	0.019243424	1	157.7456606	Not Rhythmic	Not Applicable
ENSMUSG00000028919	Arhgef19	0.033071808	1	231.0628193	Not Rhythmic	Not Applicable
ENSMUSG00000027599	Armc1	0.033071808	1	98.03542534	Not Rhythmic	Not Applicable
ENSMUSG00000002910	Arrdc2	0.000717948	0.895546749	134.2907886	Not Rhythmic	Not Applicable
ENSMUSG00000030996	Art1	0.000252129	1	762.2143359	Not Rhythmic	Not Applicable
ENSMUSG00000070424	Art5	0.011575813	1	92.29457168	Not Rhythmic	Not Applicable
ENSMUSG00000021200	Asb2	0.022168781	1	913.8785895	Not Rhythmic	Not Applicable
ENSMUSG00000028207	Asph	0.014692162	1	223.072885	Not Rhythmic	Not Applicable
ENSMUSG00000026663	Atf6	0.000126318	1	300.800561	Not Rhythmic	Not Applicable
ENSMUSG00000029673	Auts2	0.000873619	1	176.9617755	Not Rhythmic	Not Applicable
ENSMUSG00000075010	AW112010	0.025495919	1	54.24163389	Not Rhythmic	Not Applicable
ENSMUSG00000025372	Baiap2	0.001250287	1	89.03864626	Not Rhythmic	Not Applicable
ENSMUSG00000002748	Baz1b	0.006085424	1	111.3940764	Not Rhythmic	Not Applicable
ENSMUSG00000007659	Bcl2l1	0.001869435	1	134.467431	Not Rhythmic	Not Applicable
ENSMUSG00000000317	Bcl6b	0.003214659	1	147.3616769	Not Rhythmic	Not Applicable
ENSMUSG00000030103	Bhlhe40	0.000717948	0.339122376	191.2304925	Not Rhythmic	Not Applicable
ENSMUSG00000030256	Bhlhe41	0.000583768	0.573368658	148.6799321	Not Rhythmic	Not Applicable
ENSMUSG00000016758	Bik	0.008381805	1	44.09724334	Not Rhythmic	Not Applicable
ENSMUSG00000024335	Brd2	0.019243424	1	149.3245089	Not Rhythmic	Not Applicable
ENSMUSG00000029544	Cabp1	0.009968119	0.339122376	31.47203036	Not Rhythmic	Not Applicable
ENSMUSG00000046447	Camk2n1	0.025495919	0.244204264	117.0092688	Not Rhythmic	Not Applicable
ENSMUSG00000024942	Capn1	0.028824062	1	50.05396112	Not Rhythmic	Not Applicable
ENSMUSG00000001794	Capns1	0.013091819	1	437.056509	Not Rhythmic	Not Applicable
ENSMUSG00000027184	Caprin1	0.009968119	1	160.7352729	Not Rhythmic	Not Applicable

ENSMUSG00000000805	Car4	0.009968119	1	171.2493761	Not Rhythmic	Not Applicable
ENSMUSG00000045954	Cavin2	0.025495919	1	220.6699004	Not Rhythmic	Not Applicable
ENSMUSG00000020074	Ccar1	0.003214659	0.694751079	362.8184384	Not Rhythmic	Not Applicable
ENSMUSG00000020925	Ccdc43	0.028824062	1	52.34260682	Not Rhythmic	Not Applicable
ENSMUSG00000095098	Ccdc85b	0.014692162	1	243.3402459	Not Rhythmic	Not Applicable
ENSMUSG00000029617	Ccz1	0.011575813	1	69.52208408	Not Rhythmic	Not Applicable
ENSMUSG00000025351	Cd63	0.025495919	1	331.3683357	Not Rhythmic	Not Applicable
ENSMUSG00000027435	Cd93	0.006085424	1	267.1769058	Not Rhythmic	Not Applicable
ENSMUSG00000027330	Cdc25b	0.014692162	0.448935165	18.76167369	Not Rhythmic	Not Applicable
ENSMUSG00000024780	Cdc37l1	0.003754435	1	192.7182201	Not Rhythmic	Not Applicable
ENSMUSG00000020015	Cdk17	0.011575813	1	42.17316629	Not Rhythmic	Not Applicable
ENSMUSG00000021483	Cdk20	0.033071808	1	19.38063135	Not Rhythmic	Not Applicable
ENSMUSG00000023067	Cdkn1a	0.028824062	1	172.7477634	Not Rhythmic	Not Applicable
ENSMUSG00000037443	Cep85	0.014692162	1	217.2427064	Not Rhythmic	Not Applicable
ENSMUSG00000027313	Chac1	0.006085424	1	33.43661676	Not Rhythmic	Not Applicable
ENSMUSG00000020309	Chac2	0.003754435	1	59.69744702	Not Rhythmic	Not Applicable
ENSMUSG00000057133	Chd6	0.019243424	1	184.833159	Not Rhythmic	Not Applicable
ENSMUSG00000041235	Chd7	0.022168781	1	143.2513603	Not Rhythmic	Not Applicable
ENSMUSG00000000743	Chmp1a	0.028824062	1	122.8841745	Not Rhythmic	Not Applicable
ENSMUSG00000001774	Chordc1	0.000252129	1	134.9390439	Not Rhythmic	Not Applicable
ENSMUSG00000006958	Chrd	0.001250287	1	28.73668858	Not Rhythmic	Not Applicable
ENSMUSG00000038550	Ciart	0.001250287	0.073875181	129.7866555	Not Rhythmic	Not Applicable
ENSMUSG00000034157	Cipc	0.002231251	0.617578436	149.0380757	Not Rhythmic	Not Applicable
ENSMUSG00000045193	Cirbp	0.000297339	1	357.0849294	Not Rhythmic	Not Applicable
ENSMUSG00000046841	Ckap4	0.003214659	1	167.5743289	Not Rhythmic	Not Applicable
ENSMUSG00000064302	Clasp1	7.05E-08	0.573368658	1039.884954	Not Rhythmic	Not Applicable
ENSMUSG00000049550	Clip1	0.014692162	1	385.5319899	Not Rhythmic	Not Applicable
ENSMUSG00000063146	Clip2	0.016916742	0.694751079	41.66068561	Not Rhythmic	Not Applicable
ENSMUSG00000029238	Clock	1.08307E-06	0.124032757	217.5789263	Not Rhythmic	Not Applicable
ENSMUSG00000015357	Clpx	0.019243424	1	184.3844971	Not Rhythmic	Not Applicable
ENSMUSG00000022037	Clu	0.033071808	1	1736.191929	Not Rhythmic	Not Applicable
ENSMUSG00000031875	Cmtm3	0.011575813	1	75.8976885	Not Rhythmic	Not Applicable
ENSMUSG00000032434	Cmtm6	0.001250287	1	107.751553	Not Rhythmic	Not Applicable
ENSMUSG00000028339	Col15a1	0.000297339	1	362.4870166	Not Rhythmic	Not Applicable
ENSMUSG00000001506	Col1a1	0.003754435	1	468.699287	Not Rhythmic	Not Applicable
ENSMUSG00000026043	Col3a1	0.000873619	1	951.4080056	Not Rhythmic	Not Applicable

ENSMUSG00000031502	Col4a1	0.000449642	1	1303.658233	Not Rhythmic	Not Applicable
ENSMUSG00000031503	Col4a2	7.22416E-06	1	1192.176159	Not Rhythmic	Not Applicable
ENSMUSG00000026837	Col5a1	0.028824062	1	95.64119565	Not Rhythmic	Not Applicable
ENSMUSG00000001119	Col6a1	0.014692162	1	233.3610558	Not Rhythmic	Not Applicable
ENSMUSG00000020241	Col6a2	0.013091819	1	261.6483816	Not Rhythmic	Not Applicable
ENSMUSG00000057606	Colq	0.009968119	1	91.64844713	Not Rhythmic	Not Applicable
ENSMUSG00000025981	Coq10b	0.002686921	1	233.046304	Not Rhythmic	Not Applicable
ENSMUSG00000026489	Coq8a	0.003754435	1	1243.629412	Not Rhythmic	Not Applicable
ENSMUSG00000025586	Cpeb1	0.007203	1	71.79955783	Not Rhythmic	Not Applicable
ENSMUSG00000025867	Cplx2	0.028824062	1	16.9761572	Not Rhythmic	Not Applicable
ENSMUSG00000048200	Cracr2b	0.006085424	1	69.84284008	Not Rhythmic	Not Applicable
ENSMUSG00000032515	Csrnp1	0.016916742	1	66.5615342	Not Rhythmic	Not Applicable
ENSMUSG00000031778	Cx3cl1	0.019243424	1	41.31143068	Not Rhythmic	Not Applicable
ENSMUSG00000052336	Cx3cr1	0.028824062	1	23.62698236	Not Rhythmic	Not Applicable
ENSMUSG00000019590	Cyb561	0.025495919	1	80.4931526	Not Rhythmic	Not Applicable
ENSMUSG00000020810	Cygb	0.000297339	1	406.3570748	Not Rhythmic	Not Applicable
ENSMUSG00000026170	Cyp27a1	0.022168781	1	79.25421678	Not Rhythmic	Not Applicable
ENSMUSG00000028713	Cyp4b1	0.028824062	1	118.5117766	Not Rhythmic	Not Applicable
ENSMUSG00000034574	Daam1	0.028824062	1	131.0005928	Not Rhythmic	Not Applicable
ENSMUSG00000040260	Daam2	0.033071808	1	34.71042256	Not Rhythmic	Not Applicable

Supplementary Table 10. JTK cycle analysis identifies transcripts that oscillate with a periodicity of 24hrs in CON hearts, for which oscillations are either abolished or significantly attenuated in dnOGAh heart. Tissues were isolated from mice at distinct times of the day, followed by assessment of gene expression by RNAseq. 5 mice per experimental group.
